# Prenylated Isoflavones Originating from East African Medicinal Plants Selectively Modulate the α‑Glucosidase from *Saccharomyces cerevisiae*

**DOI:** 10.1021/acs.jnatprod.6c00411

**Published:** 2026-05-08

**Authors:** Kai Lüersen, Friederike Neuber, Eric Sperlich, Alexandra Kelling, Sarah von Chamier-Gliszczinski, Julia Greese, Taye B. Demissie, Bernd Schmidt, Gerald Rimbach, Vaderament-Alexe Nchiozem-Ngnitedem

**Affiliations:** † Institute of Human Nutrition and Food Science, 9179 University of Kiel, Kiel D-24118, Germany; ‡ 26583 University of Potsdam, Institut für Chemie, Karl-Liebknecht-Strasse 24-25, Potsdam-Golm, Potsdam D-14476, Germany; § Department of Chemistry, Faculty of Science, University of Botswana, Gaborone, 00704, Botswana

## Abstract

Nine naturally occurring prenylated isoflavones and three nonnatural analogues were synthesized for the first time. The 12 newly synthesized and 11 previously synthesized isoflavones were tested for their activity as modulators of *Saccharomyces cerevisiae* α-glucosidase. Out of the 23 prenylated isoflavones tested in total, 4′,7-dihydroxy-3′,5′-diprenylisoflavone (**26**) and 8-prenyldaidzein (**28**), both secondary plant metabolites originally isolated from *Psoralea corylifolia*, were found to be the most potent inhibitors of *S. cerevisiae* α-glucosidase, with IC_50_ values of 7.8 ± 2.3 μM and 14.6 ± 5.1 μM, respectively. Kinetic analysis revealed that **26** acts through a noncompetitive mechanism and **28** through an uncompetitive mechanism of inhibition. Surprisingly, a few of the compounds tested, in particular isoflavones with a 6,7- and 3′,4′-dioxy substitution pattern, were found to be activators of *S. cerevisiae* α-glucosidase. The most notable effects in this regard were observed for predurallone (**21c**), a secondary plant metabolite originally isolated from *Millettia dura*, and its nonnatural analogue 7-methylpredurallone (**22c**). At a concentration of 50 μM, both compounds enhanced the activity of *S. cerevisiae* α-glucosidase by 53.7 ± 8.8% and 41.5 ± 8.2%, respectively. To the best of our knowledge, these are the first examples of small molecule activators of this enzyme.

## Graphical abstract

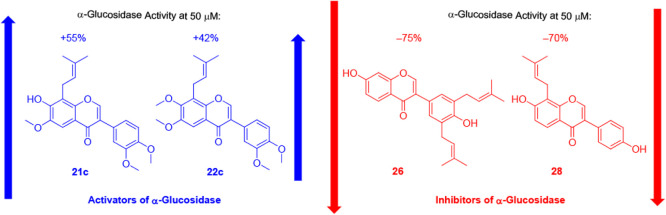

α-Glucosidase and α-amylase play a key role in the digestive system by catalyzing the hydrolytic cleavage of dietary starch, sucrose and related poly-, oligo- and disaccharides into monomeric sugars. This is crucial, because only free glucose (and other monosaccharides) can be transported across the intestinal epithelium into the bloodstream. Inhibition of these glycolytic enzymes has been recognized as a way of controlling the postprandial blood glucose level, and is hence an important therapeutic concept for the treatment of hyperglycemia associated with type-2 diabetes mellitus.^1^ Elevated blood glucose levels due to insufficient insulin production by β-cells and/or insulin resistance in liver, muscle and other tissues are a significant contributor to long-term complications. These include cardiovascular diseases, retinopathy, nephropathy, neuropathy, and increased risk of premature mortality.^2^ Important synthetic α-glucosidase inhibitors that are currently in clinical use are acarbose,^3^ miglitol^4^ and voglibose^5^ (Figure 1). While miglitol and voglibose are selective inhibitors of α-glucosidase, acarbose is a dual inhibitor of α-glucosidase and α-amylase. Hypoglycemic effects have also been found for several nutritional plants and extracts of these plants.^1,6^ For instance, a health promoting effect of *Morus australis*, the Chinese mulberry, has been known for quite some time^7^ and is inter alia attributed to the blood glucose lowering effect of its phytochemical constituents, in particular 1-deoxynojirimycin.^8,9^ Investigations into hypoglycemic activities of plant extracts and isolated secondary plant metabolites is relevant for several reasons. For one, natural products can be structural blueprints for active pharmaceutical ingredients (API), as for example 1-deoxynojirimycin (natural product) and miglitol (API) (Figure 1).

**Figure 1:**
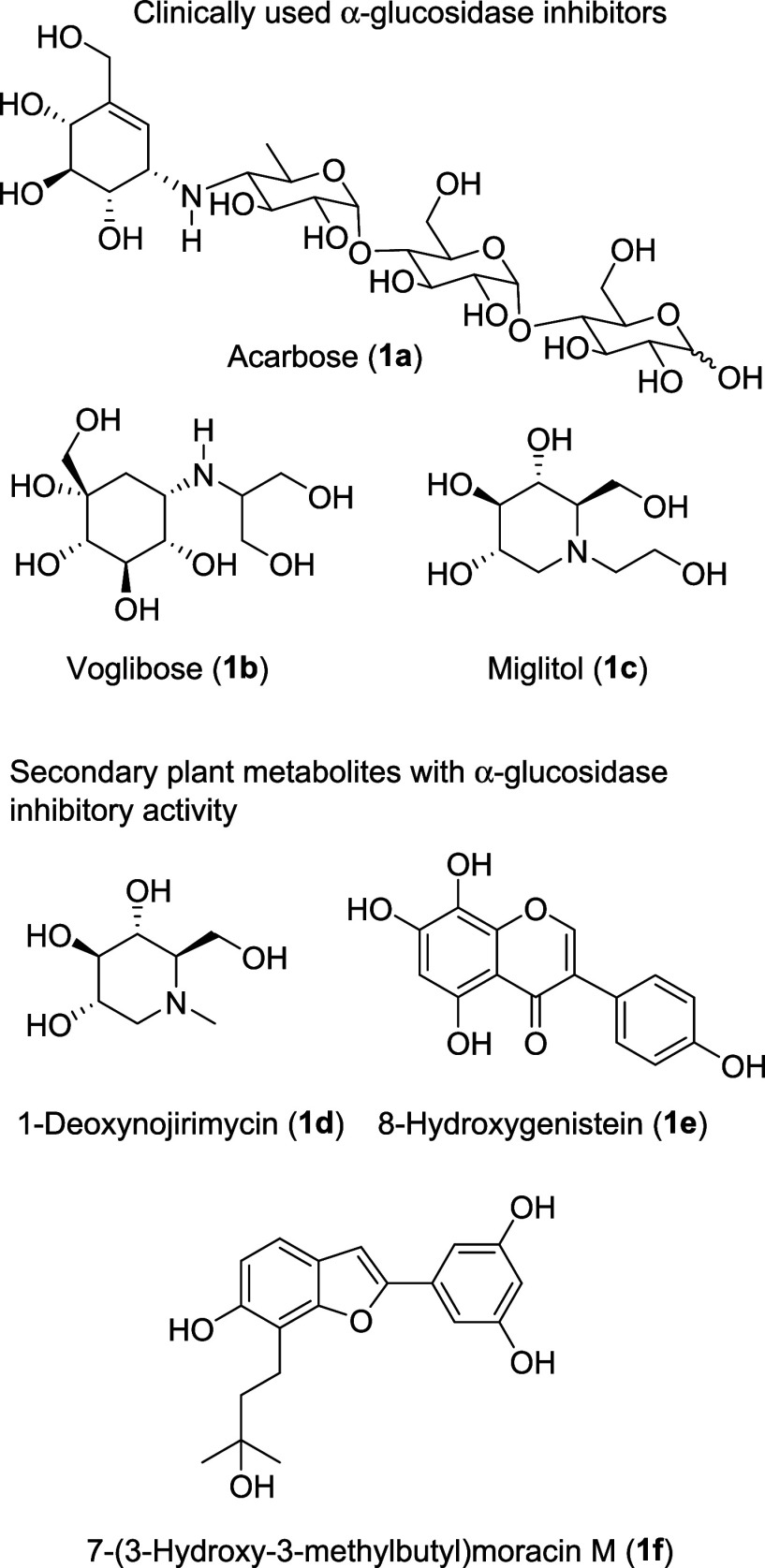
Clinically used inhibitors of α-glucosidase (top) and secondary plant metabolites with proven inhibitory activity toward α-glucosidase (bottom).

A second reason lies in the increasing prevalence of diabetes mellitus in many African countries, where the disease is often treated by traditional health practitioners with herbal remedies due to limited access to or lack of trust in Western medicine.^10^ For example, leaf extracts of the African mulberry *Morus mesozygia* Stapf are widely used in traditional medicine in many regions of Africa for the treatment of diabetes mellitus.^11^ In a phytochemical study on *M. mesozygia* Stapf some of us have recently identified prenylated benzofuran-type polyphenols as secondary metabolites with notable inhibitory activity against α-glucosidase (e.g., 7-(3-hydroxy-3-methylbutyl)­moracin M (Figure 1): IC_50_ 16.9 μM). This example shows how the traditional use of herbal remedies can be corroborated by scientific means.^12^ The use of medicinal plants for managing diabetes mellitus based on indigenous knowledge in Africa has been the subject of numerous reviews.^10,13–20^ Plants of the *Fabaceae* family represent the largest source of antidiabetic natural products, and with regard to chemical structure terpenes (33%) and flavonoids and chalcones (combined 28%)^21^ are the two largest classes of plant metabolites with proven antidiabetic activity.^6^ It was, for example, shown by two of us that soy extract enriched in hydroxy isoflavones (e.g., 8-hydroxygenistein, Figure 1) by fermentation shows a 6-fold increase of α-glucosidase inhibitory activity compared to the positive control acarbose.^22^ Most investigations into the antidiabetic potential of isoflavones focus on very few compounds, such as genistein, daidzein and formononetin present in nutritional plants such as soybeans.^23^ Structurally more complex isoflavones, in particular prenylated isoflavones, have attracted considerably less attention with regard to their hypoglycemic activity, although many of these compounds have been isolated^24^ and investigated for other bioactivities.^25–27^ Examples of α-glucosidase inhibitors with a prenylated isoflavone structure are retamasin H, isolated from *Retama raetam*,^28^ millewanin G, isolated from *Masclura tricuspidate*,^29^ and 6-chloromaximaisoflavone J, a synthetic analogue of maximaisoflavone J (a natural product from plants of the genus *Millettia*)^30^ (Figure 2).

**Figure 2:**
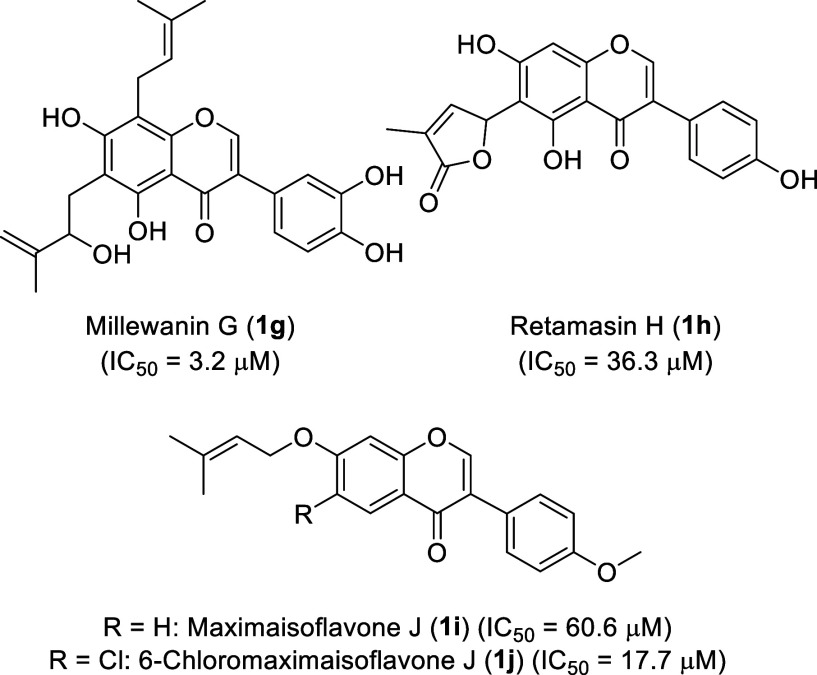
Prenylated isoflavones with inhibitory activity against α-glucosidase.

Over the past few years, two of us have developed synthetic routes to prenylated isoflavones,^31–34^ mainly found in African medicinal plants, with the aim to overcome the supply problem: very often only small amounts of material are available by isolation from the natural source, which hampers the biological evaluation.^35^ In addition, purification of natural products and quantitative separation from other metabolites can be very difficult, which may limit the reliability of bioactivity data for compounds obtained from natural sources. Chemical synthesis of the secondary metabolites can help to resolve both the supply and the reliability problem.

Herein, we report our results on the first syntheses of 12 prenylated isoflavones that were mainly isolated from African medicinal plants of the genus *Millettia*,^36–43^ and their evaluation as antidiabetic agents, together with the evaluation of 11 natural and nonnatural isoflavones that were previously synthesized by us. Although plants of the genus *Millettia* play an outstanding role in African traditional medicine, they are apparently not widely used traditionally for the treatment of diabetes.^44^ They are, however, a rich source of prenylated isoflavone metabolites, in particular with a 6,7-dioxygenation pattern, which have so far not been investigated as inhibitors of glycoside hydrolases.

## Results and Discussion

### Synthesis of 8-Prenyl-6,7-dioxygenated Isoflavones from *Millettia* sp. and Nonnatural Analogues

Numerous methods and strategies for the total synthesis of isoflavonoids are available, and their application has recently been comprehensively reviewed by Selepe et al.^25,45^ From our former work in this field,^31,34,35^ we found the implementation of a Pd-catalyzed cross coupling reaction with boronic acids^46–48^ as a key step for introducing the B ring particularly useful, because structural variation within the B-ring can easily be realized by using different B-ring coupling partners and a common building block for the chromone part (rings A and C). Conveniently, the latter is introduced as an iodochromone **2**, and the former as a boronic acid **3** (Scheme 1).

**Scheme 1:**
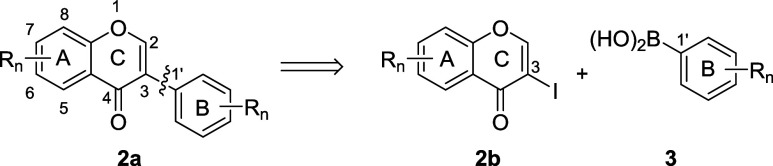
Pd-Catalyzed Coupling for the Synthesis of Isoflavones **1** and Numbering Scheme

For the synthesis of 8-prenyl-6,7-dioxygenated isoflavones we chose a MOM-protected 3-iodochromone **12** as the electrophilic coupling partner for the Pd-catalyzed cross coupling reaction. Precursor **12** was synthesized from isovanillin (**4**) in eight steps and 34% overall yield (Scheme 2). Isovanillin (**4**) was first converted to phenol **5** by a Se-catalyzed oxidation-alkaline hydrolysis sequence.^49,50^ The acetophenone derivative **6** was obtained from **5** by Lewis-acid mediated acetylation.^51^ In the next step, the phenol group *para* to the acetyl group in **6** underwent selective *O*-allylation under basic conditions to yield allyl ether **7**,^52^ which underwent a sigmatropic rearrangement upon microwave irradiation at 250 °C to furnish the pentasubstituted benzene **8**. The rearrangement of allyl ether **7** to allylbenzene **8** had previously been described in the literature using conventional heating in an oil bath.^52^ Protection of **8** as a methoxymethyl-(MOM)-ether was accomplished regioselectively at the *para*-OH group to give **9**, which was next reacted with dimethylformamide-dimethylacetal (DMF-DMA), again under microwave conditions, to give enamine **10**. Enamine **10** was cyclized to 3-iodochromone **11** with iodine under basic conditions, and eventually the prenyl group at C-8 was introduced by cross metathesis^53^ using 2-methyl-2-butene and second-generation olefin metathesis catalyst **A**.^54^

**Scheme 2:**
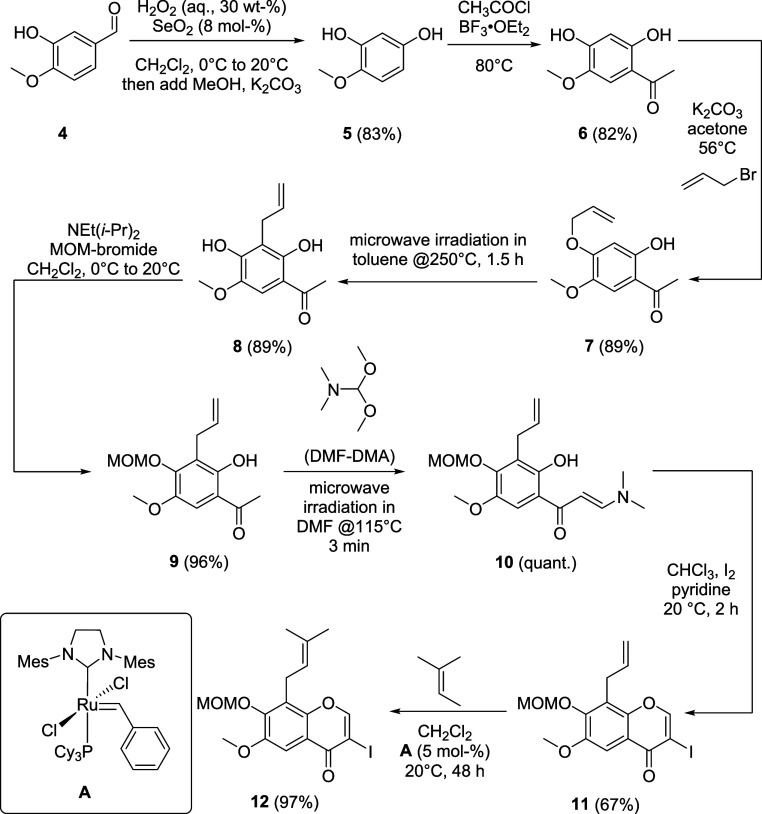
Synthesis of Iodochromone **12**

Six different boronic acids **3a**–**f** were used as coupling partners. Boronic acids **3a**–**d** (Table 1) are commercially available and were purchased, **3e** and **3f** were synthesized as outlined in Scheme 3 and Scheme 4. Starting from veratraldehyde (**13**), the aryl bromide **16** was obtained in three steps.^55–57^ Boronic acid **3e** ^58^ was synthesized from **16** by bromine–lithium exchange, borylation and finally acidic hydrolysis of the boronate.

**Table 1:**
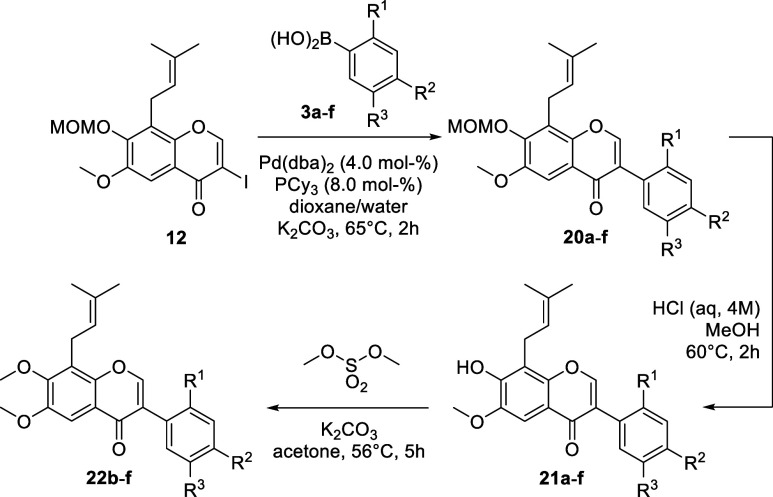
Synthesis of Isoflavones **20**, **21** and **22**

Entry	3	R^1^	R^2^	R^3^	20 (yield, %)	21 (yield, %)	22 (yield, %)
1	**3a**	H	OH	H	**20a** (88)	**21a** (83)	–^a^
2	**3b**	H	OCH_3_	H	**20b** (77)	**21b** (93)	**22b** (98)
3	**3c**	H	OCH_3_	OCH_3_	**20c** (84)	**21c** (84)	**22c** (90)
4	**3d**	H	–OCH_2_O–		**20d** (81)	**21d** (97)	**22d** (94)
5	**3e**	OCH_3_	OCH_3_	OCH_3_	**20e** (52)	**21e** (88)	**22e** (quant.)
6	**3f**	OCH_3_	–OCH_2_O–		**20f** (54)	**21f** (98)	**22f** (93)

a Not undertaken due to unselective methylation; dual methylation would furnish **22b**.

**Scheme 3:**
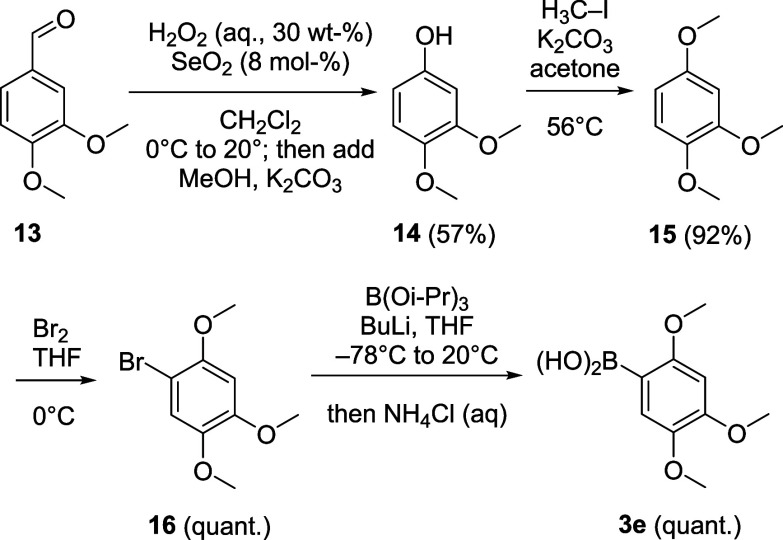
Synthesis of Boronic Acid **3e**

**Scheme 4:**
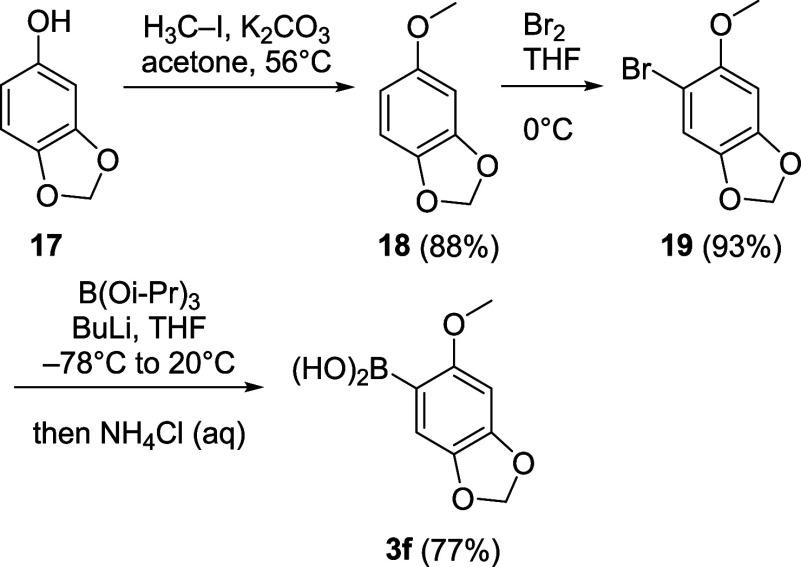
Synthesis of Boronic Acid **3f**

For the synthesis of **3f**, sesamol (**17**) was used as a starting material. Methylation and bromination following a modified literature procedure furnished **19**.^59^ Bromine–lithium exchange and borylation/hydrolysis then gave boronic acid **3f**.

Pd-catalyzed cross coupling of 3-iodochromone **12** with boronic acids **3a**–**f** was accomplished using a catalyst system consisting of Pd­(dba)_2_ and tricyclohexylphosphine as activating ligand^31,60,61^ and furnished MOM-protected isoflavones **20a**–**f**. Cleavage of the methoxymethyl ether was achieved with hydrochloric acid and gave the 7-hydroxyisoflavones **21**. The analogous 7-methoxyisoflavones **22b**–**f** were obtained by reacting **21b**–**f** with dimethylsulfate (Table 1).

Some bioactive isoflavones possess a prenyloxy substituent, as for example the *Millettia*-metabolite maximaisoflavone J and its 6-chloro derivative (Figure 2). As an example of a prenyl ether, we therefore synthesized compound **23** by base mediated prenylation of **21d** (Scheme 5).

**Scheme 5:**
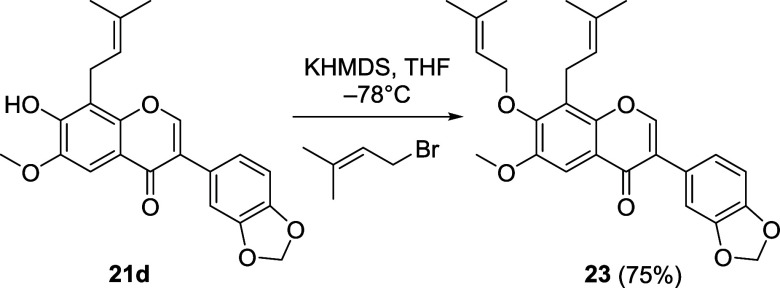
Synthesis of *O*-Prenylated Isoflavone **23**

For the example of tetramethyl ether **22c**, which is the methyl ether of the natural product predurallone, the molecular structure was unambiguously corroborated by single crystal X-ray structure analysis (Figure 3).

**Figure 3:**
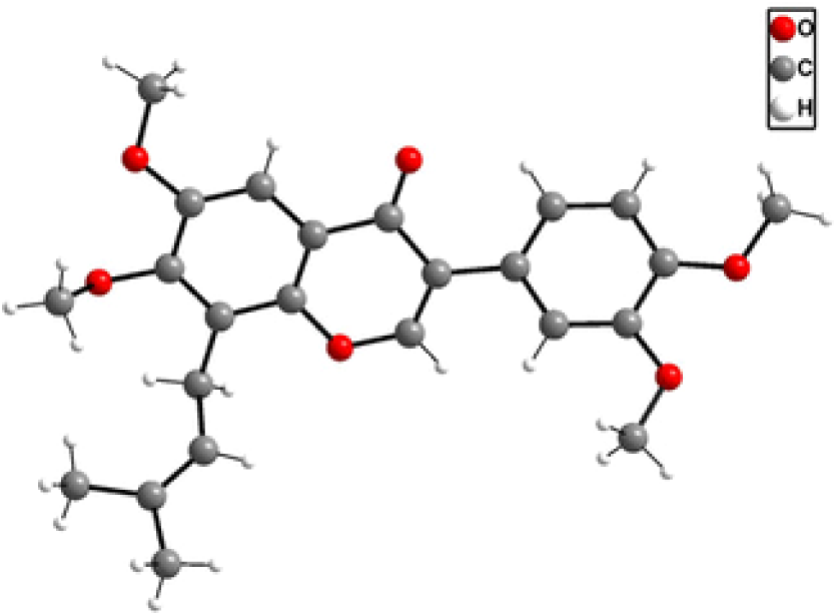
Single crystal X-ray structure of **22c**.

Structures of all compounds tested for their antihyperglycemic activity in this study are shown below, including isoflavones **21a**–**f**, **22b**–**f** and **23**, which had not been previously synthesized.
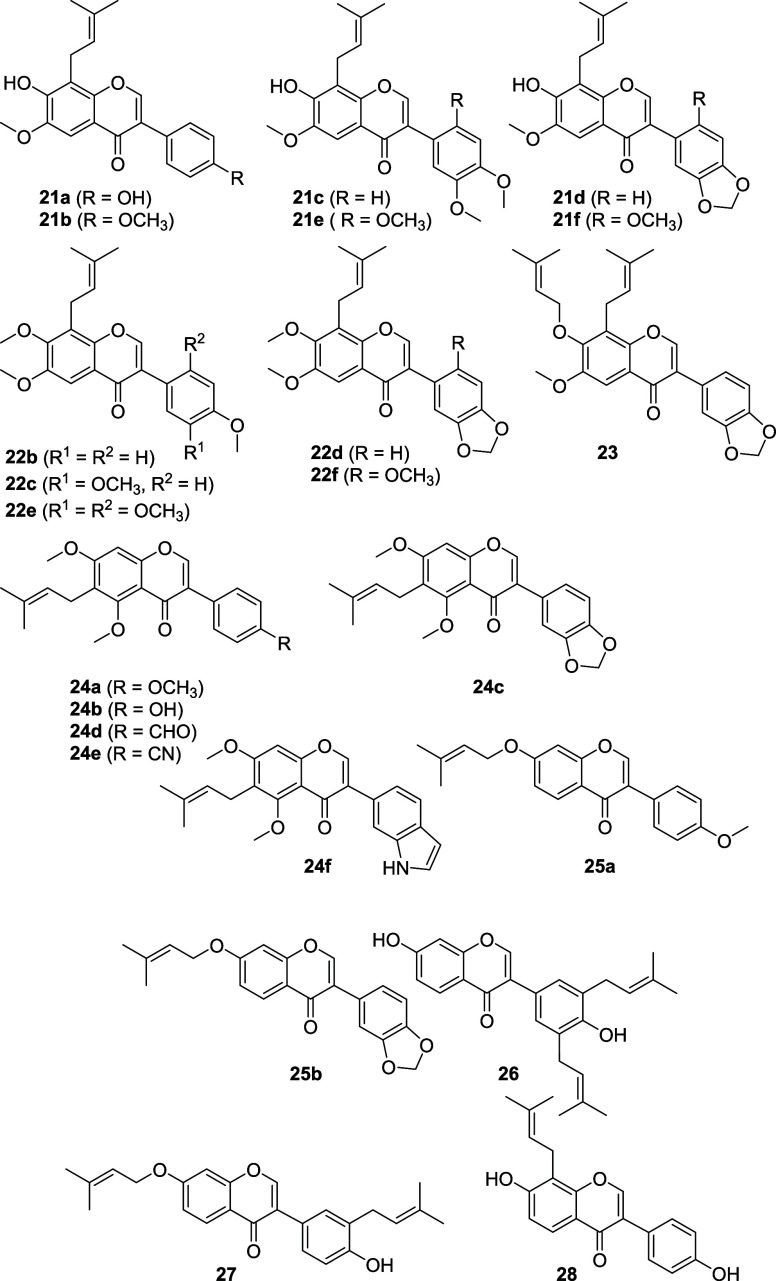


The *Ficus carica* metabolite ficucaricone D (**24a**) and its nonnatural analogues **24b**–**f**,^34^ the *Millettia dura* metabolites maximaisoflavone J (**25a**) and maximaisoflavone B (**25b**),^31^ and the *Psoralea corylifolia* metabolites 4′,7-dihydroxy-3′,5′-diprenylisoflavone (**26**), 7-*O*-isoprenylneobavaisoflavone (**27**) and 8-prenyldaidzein (**28**) were previously synthesized by us.^35^ All compounds **21a**–**f**, **22b**–**f** and **23** have not been synthesized previously, and among these are the natural products millesianin H (**21b**),^62^ predurallone (**21c**),^63^ predurmillone (**21d**),^62,64^ millesianin I^62^ (also published as pachyloisoflavone A,^65^ **21e**), millesianin D (**21f**),^62,66,67^ pachyvone A (**22b**),^68^ pachyvone B (**22d**),^68,69^ placoisoflavone A^70^ (also published as pachyvone C,^68^ **22e**), and 8-prenylmilldurone (**22f**).^68,71^ Isoflavones **21a**, **22c** and **23** are new compounds that have not (or not yet) been reported as natural products. A tabular overview of the natural products synthesized in the course of this study, the natural sources they were isolated from, and reported bioactivities is given in the Supporting Information (Table S3).

### The Prenylated Isoflavones **26** and **28** Are Potent Inhibitors of *Saccharomyces cerevisiae* α-Glucosidase

Screening of the 23 isoflavone compounds set at a concentration of 50 μM against the α-glucosidase from baker’s yeast revealed distinct modulatory effects (Figure 4A). Nineteen compounds altered enzyme activity by less than 25%, and were, therefore, not further examined. However, compounds **26** and **28** emerged as potent inhibitors of *S. cerevisiae* α-glucosidase, decreasing enzymatic activity by 76.4 ± 10.7% and 69.2 ± 21.3%, respectively. For comparison, the reference inhibitor acarbose when administered at 300 μM reduced α-glucosidase activity by about 40%. The inhibitory effect of compound **26** and **28** on *S. cerevisiae* α-glucosidase appears to be specific, as neither of them, nor any of the other 21 isoflavone compounds tested at 50 μM, appreciably affected porcine pancreatic α-amylase activity (Figure 4A). Consistent with results by Zulfiqar et al.,^72^ the reference inhibitor acarbose at 50 μM reduced α-amylase enzyme by approximately 95%.

**Figure 4:**
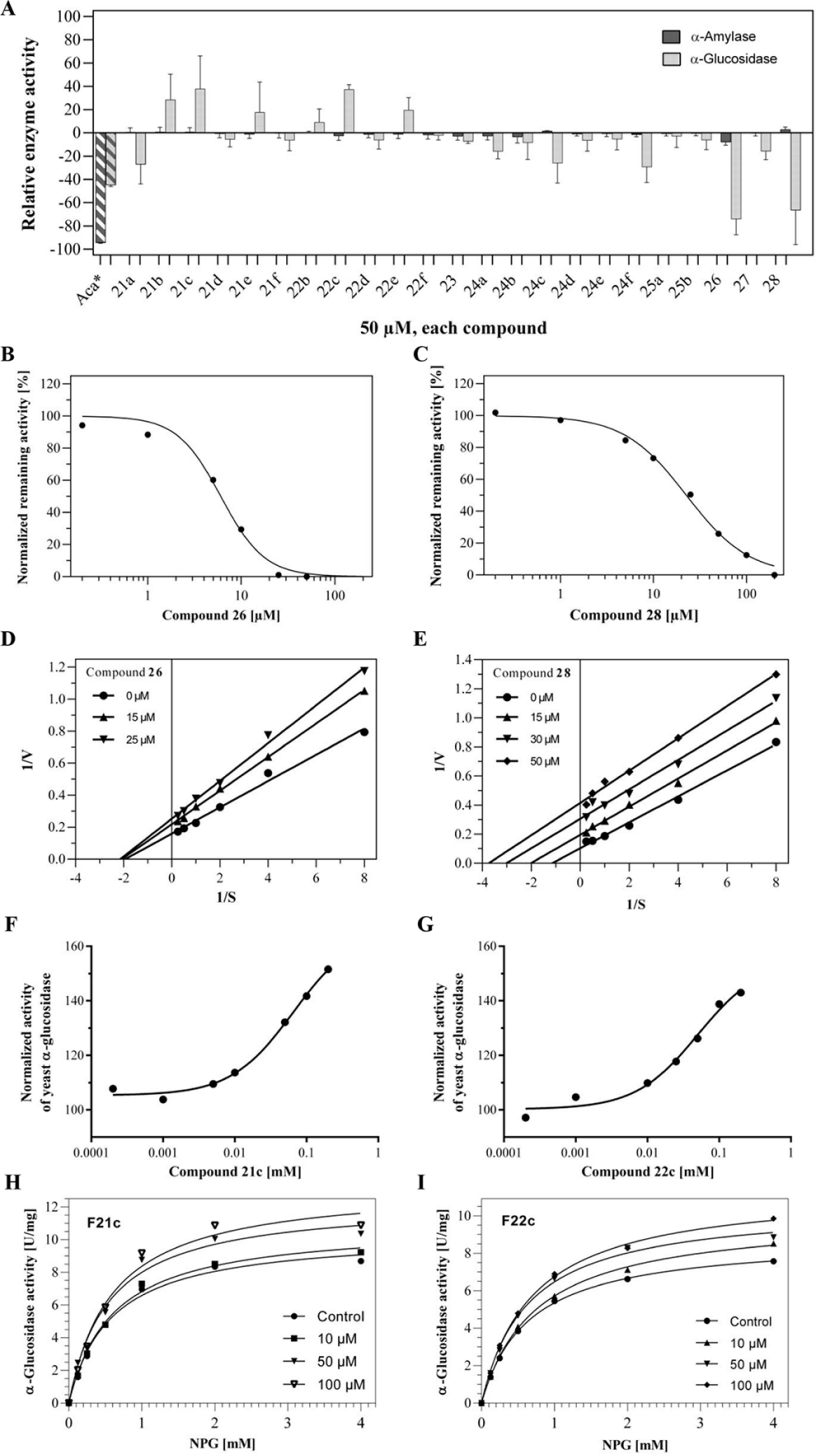
Prenylated isoflavones **21c**, **22c**, **26**, and **28** are potent modulators of *Saccharomyces cerevisiae* α-glucosidase. (A) Prenylated isoflavones were added to both standard α-amylase and α-glucosidase assays, with each test compound having a final concentration of 50 μM. The impact on the activity of the *S. cerevisiae* enzyme was determined relative to a solvent control containing 10% DMSO and expressed as a percentage. Negative values indicate inhibition, positive values indicate activation of enzyme activity. Acarbose (Aca*) served as positive control and was added to the α-amylase and α-glucosidase assay at 50 μM and 300 μM, respectively. Data represent the mean ± SD of three independent determinations. The IC_50_ values of (B) compound **26** and (C) compound **28** were determined at a substrate concentration of 10 mM 4-NPG and, as indicated, increasing concentrations of the respective prenylated isoflavone. To assess the mode of inhibition and *K*_i_ values through Lineweaver–Burk plots, the influences of different concentrations of (D) compound **26** and (E) compound **28** on *S. cerevisiae* α-glucosidase activity at increasing 4-NPG substrate concentrations (0–4 mM) were tested. The activation curves for (F) compound **21c** and (G) compound **22c** were generated in a similar manner as in (B,C). The impact of (H) compound **21c** and (I) compound **22c** on the Michaelis–Menten kinetics of *S. cerevisiae* α-glucosidase activity was determined by using concentrations of the substrate 4-NPG ranging from 0 to 4 mM. The corresponding *V*_max_ and *K*_m_ values are presented in Table S2. All experiments were conducted three times and exemplary IC_50_, Lineweaver–Burk and Michaelis–Menten curves are displayed.

We next investigated the dose-dependence of α-glucosidase inhibition by the two prenylated isoflavones **26** and **28** and determined IC_50_ values of 7.8 ± 2.3 μM and 14.6 ± 5.1 μM, respectively (Figure 4B,C).

### Compounds **26** and **28** Are Fast-Binding Inhibitors of *S. cerevisiae* α-Glucosidase

To assess whether the inhibitory efficacy of compounds **26** and **28** could be enhanced by prolonged enzyme–inhibitor interaction in the absence of substrate, the preincubation period in the α-glucosidase assay was extended from 5 min to 15, 30, 45, and 60 min. As depicted in Figure 5, the specific activity of the *S. cerevisiae* α-glucosidase decreased with increasing preincubation time even in the absence of inhibitors. However, as indicated by the normalized specific activities, for both compounds, the rate of inhibition was not affected by any of the preincubation period tested, indicating that both compounds **26** and **28** represent fast-binding inhibitors.

**Figure 5:**
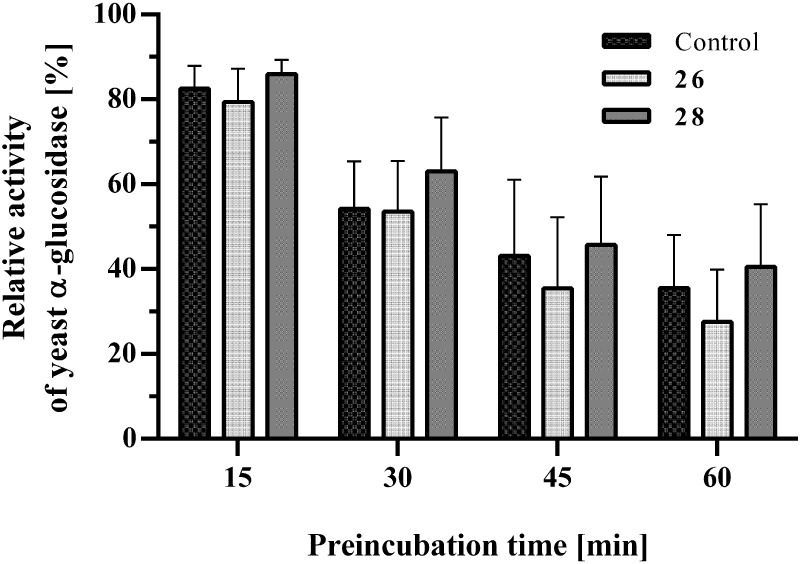
An extended preincubation period did not affect the inhibitor efficacy of compound **26** and **28** on *S. cerevisiae* α-glucosidase. Prior to the start of the standard α-glucosidase assay, different preincubation times ranging from 5 to 60 min were applied, during which the inhibitor and the yeast enzyme could interact in the absence of substrate. For each treatment, the enzyme activity determined with 5 min preincubation was set to 100%. Based on this, the relative changes in α-glucosidase activity due to extended preincubation times were calculated separately for each treatment group. No significant differences in relative enzyme activity, expressed as a percentage, were observed between the treatment groups (*n* = 3; two-way ANOVA with Dunnett’s posthoc test).

### Compounds **26** and **28** Are Noncompetitive and Uncompetitive Inhibitors of *S. cerevisiae* α-Glucosidase, Respectively

We next determined the mode of inhibition and the kinetic constant *K*_i_ for the compounds **26** and **28**. As indicated by the Lineweaver–Burk plot in Figure 4D, compound **26** acts as a noncompetitive inhibitor of *S. cerevisiae* α-glucosidase with a *K*_i_ value of 23.4 ± 3.1 μM. In contrast, compound **28** was found to inhibit the baker’s yeast enzyme uncompetitively with a *K*_i_ value of 41.2 ± 4.2 μM (Figure 4E). Given that both compounds share an identical isoflavone scaffold, the observed differences in inhibitory behavior are most likely attributable to the distinct positioning of their prenyl substituents. Such positional variation may influence (i) the compounds’ binding orientation, (ii) change interactions within the allosteric binding site, or (iii) lead to binding at a different site within the enzyme, thereby altering their overall inhibitory profiles. This is in line with two recent structure-inhibitory activity relationship studies on prenylated isoflavones isolated from the immature fruits of the Chinese mulberry *M. tricuspidate*.^29,73^ Both highlighted the critical role of the type and position of prenyl and hydroxy groups attached to the isoflavonoid skeleton with respect to *S. cerevisiae* α-glucosidase inhibition. Among the compounds studied by Jo et al.,^73^ macluraisoflavone I, characterized by a 2-hydroperoxy-3-methylbut-3-enyl group and 3′,4′-dihydroxy groups (B ring) emerged as the most potent inhibitor. However, kinetic parameters such as the type of inhibition, IC_50_ or *K*_i_ values were not determined. Cudracusisoflavone L, erysenegalensein E and millewanin G showed strong α-glucosidase inhibition of *S. cerevisiae* α-glucosidase with IC_50_ values below 10.0 μM.^29^ Here, a free 7-hydroxy group at ring A and hydroxylated linear prenyl group were suggested to enhance the inhibitory activity. Of note, the structure of the fourth effective prenylated isoflavone gancaonin M (IC_50_ value below 10 μM) exhibits structural similarity to compound **28**, however, containing a 4′-methoxy instead of a 4′-hydroxy group attached to ring B and a second 5-hydroxy group at ring A. This constellation is also found in the prenylated isoflavones 6,8-diprenylgenistein isolated from licorice *Glycyrrhiza uralensis* (IC_50_ value of 16.3 μM)^74^ and isolupalbigenin, isolated from Trifoliate jewel vine *Millettia pachycarpa* Benth (IC_50_ value of 11.3 μM for *S. cerevisiae* α-glucosidase inhibition).^75^

### The Prenylated Isoflavones **21c** and **22c** Substantially Enhance the Activity of *Saccharomyces cerevisiae* α-Glucosidase

The screening presented in Figure 4A also identified two prenylated isoflavones, compounds **21c** and **22c**, which activated *S. cerevisiae* α-glucosidase, enhancing its activity by 53.7 ± 8.8% and 41.5 ± 8.2%, respectively, when administered at a concentration of 50 μM. The activation by both compounds was dose-dependent (Figure 4F,G) and did not reach saturation up to 200 μM (further increase of the activator concentration was limited due to insolubility). The activator effect of compound **21c** and **22c** on *S. cerevisiae* α-glucosidase appears to be specific, as none of the 23 isoflavone compounds tested at 50 μM appreciably stimulate porcine pancreatic α-amylase activity (Figure 4A).

Further kinetic analyses revealed that compounds **21c** and **22c** increased the velocity of the enzyme reaction without altering the substrate affinity of the enzyme (indicated by the constant values of the apparent Michaelis constant *K*_m_) (Figure 4H). This points to a noncompetitive activation mechanism, where the activator may bind both the free enzyme and the enzyme–substrate complex.^76^

The structures of the isoflavone compounds **21c** and **22c** are characterized by a prenyl group at position 8 of ring A and two methoxy groups at ring B. Remarkably, small alterations of the substituents at ring B including the addition of a third methoxy group to ring B (compound **21e**, **22e**), the omission of one of the two methoxy group (compounds **21b**, **22b**) or the exchange of the adjacent methoxy groups by a methylenedioxy group (compound **21d**, **22d**), mitigated or abolished the activating effect on *S. cerevisiae* α-glucosidase. Therefore, we conclude that the two methoxy groups at ring B are crucial for the observed bioactivity of compound **21c** and **22c**.

Enzyme activation, whether essential or allosteric, for example by metal ions or physiological metabolites, is common in natural biochemical processes.^77,78^ Small-molecule activators, either synthetic or natural, can similarly enhance enzyme activity, serving as chemical biology tools that mimic gain-of-function mutations and may have therapeutic potential. Prominent examples for the latter are activators of glucokinase such as dorzagliatin which is intended for the treatment of type 2 diabetes.^79,80^ The underlying mode of action is based on the activation of glucokinase which leads to enhanced insulin secretion by pancreatic β-cells and improves hepatic glucose utilization. Together, this results in lowered blood–glucose concentrations and increased glucose tolerance.^79,80^ Nevertheless, pharmacological reports on small-molecule activators of enzyme activity are rare, as most drug screenings aim at enzyme inhibitors.^78^ Furthermore, identifying activators can also be challenging because enzymes are generally considered to be optimally adapted to their function in the course of evolution. In fact, to the best of our knowledge, activators of α-glucosidase activity have not been described previously.

α-Glucosidases from microorganisms, including yeasts, play an important role in biotechnology and food technology. They are, e.g., involved in fermentation processes, food processing and the production of prebiotics or low-calorie sweeteners such as isomaltose oligosaccharides.^81,82^ It would therefore be interesting to investigate whether small-molecule activators of α-glucosidases such as compound **21c** and **22c** can be used to accelerate such processes. Prior to this, however, it must be clarified whether the two prenylated isoflavone compounds increase the activity of α-glucosidases from other yeasts, molds and microorganisms in a similar way to the *S. cerevisiae* enzyme.^81^ Many α-glucosidases can perform both hydrolysis and transglucosylation reactions.^83^ In the synthesis of nonfermentable oligo-isomaltose, yeast α-glucosidase transfers glucose residues to other carbohydrate substrates, forming α-1,6-glucoside bonds. To date it is unclear, whether compounds **21c** and **22c** can also improve this catalytic step. In summary, we suggest that the use of prenylated isoflavones with α-glucosidase activator activity may have great potential in the aforementioned areas of application.

### Molecular Docking Simulations for α-Glucosidase Inhibitors **26** and **28**

Molecular docking simulations were performed with the most active inhibitors **26** and **28** to understand their interactions with the key amino acids of the proteins (Table 2 and Figure 6). With inhibition constants (*K*_i_) of 0.047 and 0.293 μM, respectively, compounds **26** and **28** demonstrated the strongest inhibitory activity against α-glucosidase Mal32 (PDB: AFP38158F1). A similar trend was also observed against another protein of α-glucosidase (PDB: 3AJ7), with *K*_i_ values of 0.727 μM and 0.893 μM, respectively. In both compounds, multiple favorable interactions with important active site residues supported their high binding affinities. Compound **26** has the lowest binding energy of −10.00 kcal/mol whereas compound **28** has −8.91 kcal/mol against α-glucosidase Mal32. Moreover, **26** fits within the pocket of the enzyme by forming hydrogen bonds of α-glucosidase Mal32 with Glu276, Ser308, and Thr307 in addition to π-interactions and multiple aromatic residues (Figure 6A,B). Similarly, compound **28** interacts with Val277 through hydrogen bond and with Phe300, Phe177, Tyr71, and Leu218 amino acids through π-sigma or π-alkyl interactions (Figure 6C,D). Overall, irrespective of the proteins, compound **26** demonstrated the strongest inhibitory effects, outperforming compound **28**, which is monoprenylated. This finding is consistent with the in vitro α-glucosidase activity of the tested compounds.

**Table 2:** Molecular Docking Scores and the Corresponding Prominent Residual Amino Acid Interactions of **26** and **28** against Two α-Glucosidase Proteins (PDB: AFP38158F1 and 3AJ7)

			Types of interactions		
Compound	Inhibition constant, *K*_i_ (μM)	Lowest binding energy (kcal/mol)	H-Bonding	π-Sigma/π-Alkyl	van der Waals
α-Glucosidase Mal32 (PDB: AFP38158F1)					
**26**	0.047	–10.00	Glu276, Ser308, Thr307	Phe300, Ala278, His279, His245	Arg312, Asp349
**28**	0.293	–8.91	Val277	Phe300, Phe177, Tyr71, Leu218	Glu276, Gly217, His245, Asp214
α-Glucosidase (PDB: 3AJ7)					
**26**	0.727	–9.37	Tyr158, Thr306, Tyr347	Glu411, Phe178, Phe303, Phe314	Arg442, Asp352
**28**	0.893	–8.25	His280, Asp307, Thr310, Ser241	Asp242, Phe303	Arg315, Ser240

**Figure 6:**
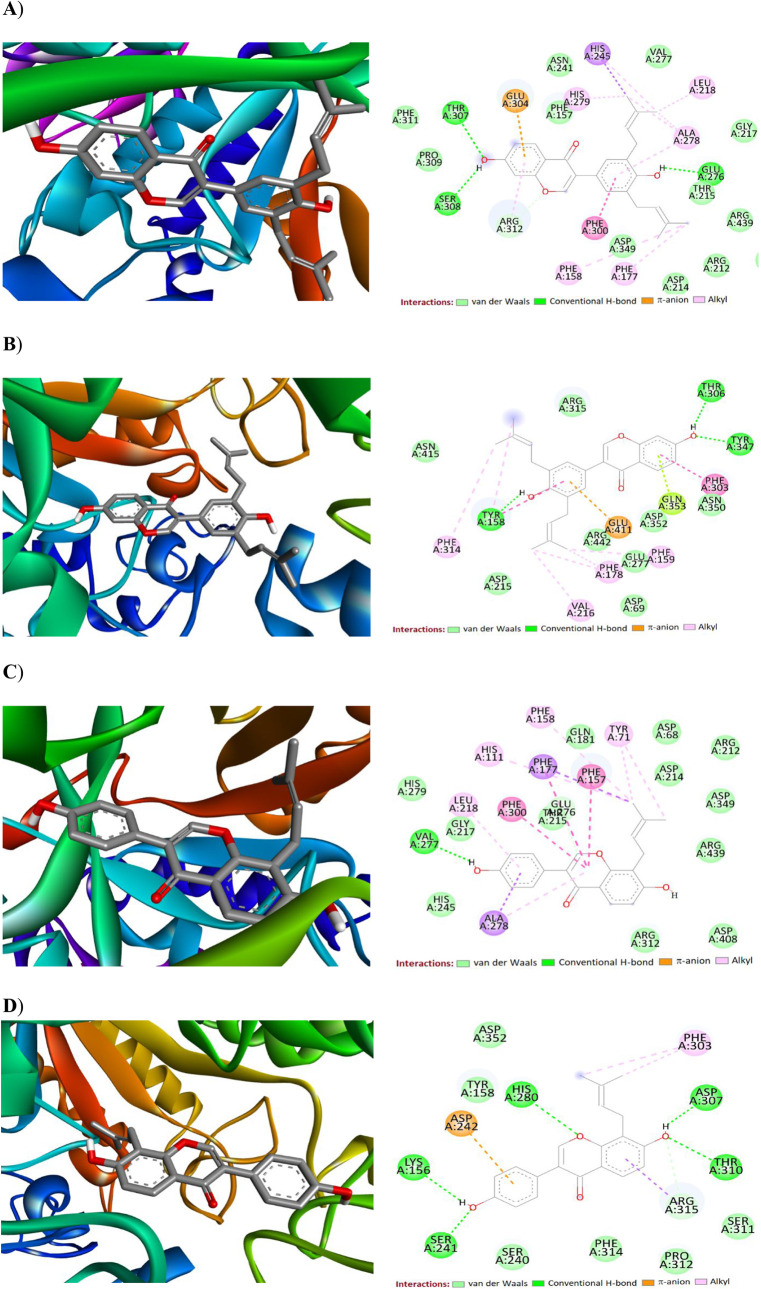
2D and 3D interactions of compound **26** in the active site of α-glucosidase Mal32 (PDB: AFP38158F1) (A) and α-glucosidase (PDB: 3AJ7) (B). 2D and 3D interactions of compound **28** in the active site of α-glucosidase Mal32 (PDB: AFP38158F1) (C) and α-glucosidase (PDB: 3AJ7) (D).

In summary, 23 prenylated isoflavones were synthesized and evaluated as modulators of α-amylase from porcine pancreas and α-glucosidase from *Saccharomyces cerevisiae*. Out of these, 15 compounds are secondary metabolites isolated from traditionally used African medicinal plants of the genus *Millettia*, or from the Chinese medicinal plant *Psoralea corylifolia*, or from the nutritional plant *Ficus carica* (common Figure tree). Eight of the compounds tested are nonnatural analogues. All compounds were obtained by chemical synthesis, which efficiently allowed to overcome the supply problem and provide sufficient quantities of material for a comprehensive evaluation of the antidiabetic properties. The two *Psoralea corylifolia* metabolites, 4′,7-dihydroxy-3′,5′-diprenylisoflavone (**26**) and 8-prenyldaidzein (**28**), were found to be potent and fast-binding inhibitors of *S. cerevisiae* α-glucosidase, with IC_50_ values of 7.8 μM and 14.6 μM, respectively. Kinetic measurements revealed that **26** and **28** act through different mechanisms: while 4′,7-dihydroxy-3′,5′-diprenylisoflavone (**26**) is a noncompetitive inhibitor, 8-prenyldaidzein (**28**) inhibits *S. cerevisiae* α-glucosidase uncompetitively. Molecular docking simulations support that **26** and **28** bind at different sites of the enzyme. Neither **26** nor **28**, nor any of the other 21 compounds tested, affected the porcine pancreatic α-amylase notably, indicating that **26** and **28** are selective α-glucosidase inhibitors. Remarkably, two of the compounds evaluated show a significant activation of *S. cerevisiae* α-glucosidase: predurallone (**21c**), a metabolite from *Millettia dura*, and its nonnatural analogue 7-*O*-methylpredurallone (**22c**) increase the activity of α-glucosidase when administered at a concentration of 50 μM by 54% and 42%, respectively. Kinetic investigations point at a noncompetitive activation mechanism with the isoflavones binding to both the free enzyme and the enzyme–substrate complex. In future studies we plan to evaluate whether results obtained in this study can be transferred to human α-glucosidase.

## Experimental Section

### General Experimental Procedures

All syntheses were conducted in dry reaction vessels under an atmosphere of dry nitrogen. Solvents were purified by standard procedures. Unless otherwise stated, reaction mixtures were heated with silicon oil baths. Microwave reactions were carried out in an Anton Paar Monowave 300 or Anton Paar Monowave 400 reactor (Monowave, maximum power 850 W, temperature control by IR sensor, vial volume 20 mL). All NMR spectra were recorded with a Bruker NEO 400 instrument from Bruker Biospin GmbH, Ettlingen, Germany. ^1^H NMR spectra were obtained at 400 MHz in CDCl_3_ with residual CHCl_3_ (δ = 7.26 ppm) as an internal reference. ^13^C­{^1^H} NMR spectra were recorded at 100 MHz in CDCl_3_ with CDCl_3_ (δ = 77.1 ppm) as an internal reference. Whenever the solubility of the sample was insufficient in CDCl_3_, it was replaced by either MeOH-*d*_4_ (CD_2_HOD as a calibrant for ^1^H NMR spectroscopy, δ = 3.31; CD_3_OD as a calibrant for ^13^C­{^1^H} NMR spectroscopy, δ = 49.0) or DMSO-*d*_6_ (DMSO-*d*_5_ as a calibrant for ^1^H NMR spectroscopy, δ = 2.50; DMSO-*d*_6_ as a calibrant for ^13^C­{^1^H} NMR spectroscopy, δ = 39.5). All signal assignments are based on 2D-NMR experiments COSY, HSQC, HMBC or NOESY. Whenever complex fine splitting of the individual lines of a signal due to long-range coupling was observed, the descriptor “m” was added to the multiplicity descriptor. The numbering scheme used for signal assignments is shown in Scheme 1. IR spectra were recorded as ATR-FTIR spectra using a PerkinElmer UATR TWO FT-IR spectrometer. Low- and high-resolution mass spectra were obtained by ESI-TOF using a Waters Micromass (Manchester, UK) instrument. For the chromatographic purification of compounds, the dry column vacuum chromatography (DCVC) method was used as described in the literature.^84^

### General Procedure for the Pd-Catalyzed Coupling of Iodochromone **12** and Boronic Acids **3**

Iodochromone **12** (430 mg, 1.00 mmol) was dissolved in dioxane (7 mL). Water (3 mL) and K_2_CO_3_ (415.0 mg, 3.00 mmol) were added, followed by the respective boronic acid **3** (1.50 mmol). The reaction mixture was stirred at ambient temperature for 10 min. PCy_3_ (22.4 mg, 0.08 mmol, 8.0 mol %) and Pd­(dba)_2_ (23.0 mg, 0.04 mmol, 4.0 mol %) were added, and the mixture was heated at 65 °C for 2 h. After complete consumption of the iodochromone **12** (TLC) the mixture was cooled to ambient temperature. A satd. aq. solution of NH_4_Cl (10 mL) was added, and the mixture was filtered. The solution was diluted with water (50 mL), and extracted with EtOAc (40 mL). The aqueous layer was separated and further extracted with EtOAc (3 times, 40 mL each). The combined organic layers were dried with anhydrous MgSO_4_, filtered, and evaporated in vacuo. The residue was purified by column chromatography on silica, using petroleum ether/EtOAc mixtures as eluents as specified for the individual compounds, to yield the MOM-protected isoflavones **20a**–**f**.

#### 3-(4-Hydroxyphenyl)-6-methoxy-7-(methoxymethoxy)-8-(3-methylbut-2-en-1-yl)-4*H*-chromen-4-one (**20a**)

Following the general procedure, compound **12** (215 mg, 0.50 mmol) and 4-hydroxyphenylboronic acid (**3a**, 103 mg, 0.75 mmol) were converted to isoflavone **20a** (176 mg, 0.44 mmol, 88%). Eluent for chromatography: petroleum ether/EtOAc (3:1 (v/v)): brown oil; IR (ATR) *ν̃* 3320 (s), 2926 (w), 1514 (m), 1461 (s), 1427 (s), 1281 (m), 1226 (m), 1071 (m), 839 (w); ^1^H NMR (400 MHz, CDCl_3_) δ 8.02 (s, 1H), 7.60 (s, 1H), 7.35 (d, *J* = 8.6 Hz, 2H), 6.82 (d, *J* = 8.6, 2H), 6.53 (s­(br.), 1H), 5.27–5.19 (m, 3H), 3.93 (s, 3H), 3.66 (d, *J* = 7.0, 2H), 3.61 (s, 3H), 1.83 (s, 3H), 1.69 (s, 3H); ^13^C­{^1^H} NMR (100 MHz, CDCl_3_) δ 176.9, 156.4, 152.8, 150.7, 150.4, 149.3, 133.0, 130.4, 125.1, 124.5, 123.9, 121.5, 120.9, 116.0, 103.9, 99.3, 57.9, 56.2, 25.9, 23.4, 18.1; HREIMS *m*/*z* 396.1586 [M^+^] (calcd for C_23_H_24_O_6_, 396.1573).

#### 6-Methoxy-7-(methoxymethoxy)-3-(4-methoxyphenyl)-8-(3-methylbut-2-en-1-yl)-4*H*-chromen-4-one (**20b**)

Following the general procedure, compound **12** (430 mg, 1.00 mmol) and 4-methoxyphenylboronic acid (**3b**, 228 mg, 1.50 mmol) were converted to isoflavone **20b** (318 mg, 0.77 mmol, 77%); eluent for chromatography: petroleum ether/EtOAc (5:1 (v/v)): yellow oil; IR (ATR) *ν̃* 2929 (w), 1639 (s), 1607 (m), 1512 (s), 1460 (s), 1427 (s), 1246 (m), 1032 (m), 952 (w); ^1^H NMR (400 MHz, CDCl_3_) δ 8.01 (s, 1H), 7.60 (s, 1H), 7.52 (d, *J* = 8.7 Hz, 2H), 6.98 (d, *J* = 8.7 Hz, 2H), 5.25 (tm, *J* = 7.2 Hz, 1H), 5.23 (s, 2H), 3.94 (s, 3H), 3.85 (s, 3H), 3.65 (d, *J* = 7.0, 2H), 3.61 (s, 3H), 1.84 (s, 3H), 1.69 (s, 3H); ^13^C­{^1^H} NMR (100 MHz, CDCl_3_) δ 176.2, 159.7, 152.4, 150.6, 150.2, 149.0, 132.9, 130.2, 125.1, 124.6, 124.1, 121.6, 121.1, 114.1, 104.0, 99.3, 57.9, 56.2, 55.5, 25.9, 23.4, 18.1; HREIMS *m*/*z* 410.1725 [M^+^] (calcd for C_24_H_26_O_6_, 410.1729).

#### 3-(3,4-Dimethoxyphenyl)-6-methoxy-7-(methoxymethoxy)-8-(3-methylbut-2-en-1-yl)-4*H*-chromen-4-one (**20c**)

Following the general procedure, compound **12** (430 mg, 1.00 mmol) and 3,4-dimethoxyphenylboronic acid (**3c**, 273 mg, 1.50 mmol) were converted to isoflavone **20c** (370 mg, 0.84 mmol, 84%); eluent for chromatography: petroleum ether/EtOAc (3:1 (v/v)): yellow oil; IR (ATR) *ν̃* 2930 (w), 1637 (s), 1601 (m), 1515 (s), 1460 (s), 1427 (s), 1261 (m), 1169 (m), 1029 (m), 959 (w); ^1^H NMR (400 MHz, CDCl_3_) δ 8.05 (s, 1H), 7.60 (s, 1H), 7.25 (d, *J* = 2.0 Hz, 1H), 7.07 (dd, *J* = 8.3, 2.0 Hz, 1H), 6.93 (d, *J* = 8.3 Hz, 1H), 5.28–5.19 (m, 3H), 3.94 (s, 3H), 3.93 (s, 3H), 3.92 (s, 3H), 3.66 (d, *J* = 7.0, 2H), 3.61 (s, 3H), 1.84 (s, 3H), 1.69 (s, 3H); ^13^C­{^1^H} NMR (100 MHz, CDCl_3_) δ 176.2, 152.6, 150.6, 150.2, 149.2, 149.1, 148.9, 132.9, 125.1, 125.0, 124.1, 121.5, 121.1, 121.1, 112.6, 111.3, 103.9, 99.3, 57.9, 56.2, 56.1, 56.1, 25.9, 23.4, 18.1; HREIMS *m*/*z* 440.1830 [M^+^] (calcd for C_25_H_28_O_7_, 440.1835).

#### 3-(Benzo­[*d*]­[1,3]­dioxol-5-yl)-6-methoxy-7-(methoxymethoxy)-8-(3-methylbut-2-en-1-yl)-4*H*-chromen-4-one (**20d**)

Following the general procedure, compound **12** (430 mg, 1.00 mmol) and benzo­[*d*]­[1,3]­dioxol-5-ylboronic acid (**3d**, 249 mg, 1.50 mmol) were converted to isoflavone **20d** (344 mg, 0.81 mmol, 81%); eluent for chromatography: petroleum ether/EtOAc (6:1 (v/v)): white solid; IR (ATR) *ν̃* 2919 (w), 1637 (s), 1599 (m), 1460 (m), 1426 (s), 1232 (m), 1158 (w), 1033 (m), 916 (w); ^1^H NMR (400 MHz, CDCl_3_) δ 8.00 (s, 1H), 7.59 (s, 1H), 7.12 (d, *J* = 1.7 Hz, 1H), 6.99 (dd, *J* = 8.0, 1.7 Hz, 1H), 6.87 (d, *J* = 8.0 Hz, 1H), 5.99 (s, 2H), 5.27–5.19 (m, 3H), 3.94 (s, 3H), 3.65 (d, *J* = 7.0, 2H), 3.61 (s, 3H), 1.83 (s, 3H), 1.69 (s, 3H); ^13^C­{^1^H} NMR (100 MHz, CDCl_3_) δ 176.0, 152.6, 150.6, 150.2, 149.1, 147.8, 147.7, 132.9, 126.0, 125.1, 124.2, 122.5, 121.5, 121.0, 109.9, 108.5, 104.0, 101.3, 99.3, 57.9, 56.2, 25.9, 23.4, 18.1; HREIMS *m*/*z* 424.1525 [M^+^] (calcd for C_24_H_24_O_7_, 424.1522).

#### 6-Methoxy-7-(methoxymethoxy)-8-(3-methylbut-2-en-1-yl)-3-(2,4,5-trimethoxyphenyl)-4*H*-chromen-4-one (**20e**)

Following the general procedure, compound **12** (430 mg, 1.00 mmol) and (2,4,5-trimethoxyphenyl)­boronic acid (**3e**, 212 mg, 1.00 mmol) were converted to isoflavone **20e** (247 mg, 0.52 mmol, 52%); eluent for chromatography: petroleum ether/EtOAc (4:1 (v/v)): yellow amorphous solid; ^1^H NMR (400 MHz, CDCl_3_) δ 8.04 (s, 1H), 7.59 (s, 1H), 6.95 (s, 1H), 6.63 (s, 1H), 5.25 (tm, *J* = 6.8 Hz, 1H), 5.22 (s, 2H), 3.93 (s, 3H), 3.93 (s, 3H), 3.85 (s, 3H), 3.78 (s, 3H), 3.65 (d, *J* = 6.8 Hz, 2H), 3.60 (s, 3H), 1.83 (s, 3H), 1.69 (s, 3H); ^13^C­{^1^H} NMR (100 MHz, CDCl_3_) δ 176.1, 154.6, 154.6, 152.0, 150.5, 150.2, 149.9, 149.0, 143.2, 132.8, 125.1, 121.6, 121.1, 115.4, 112.5, 104.0, 99.3, 98.4, 57.9, 57.1, 56.7, 56.3, 56.2, 25.9, 23.4, 18.1; IR (ATR) *ν̃* 2934 (w), 1637 (m), 1603 (m), 1512 (m), 1460 (s), 1427 (s), 1315 (m), 1202 (s), 1147 (s), 1029 (s), 938 (w); HRESIMS *m*/*z* 471.2003 [M + H]^+^ (calcd for C_26_H_31_O_8_, 471.2019).

#### 6-Methoxy-3-(6-methoxybenzo­[*d*]­[1,3]­dioxol-5-yl)-7-(methoxymethoxy)-8-(3-methylbut-2-en-1-yl)-4*H*-chromen-4-one (**20f**)

Following the general procedure, compound **12** (430 mg, 1.00 mmol) and (6-methoxybenzo­[*d*]­[1,3]­dioxol-5-yl)­boronic acid (**3f**, 196 mg, 1.00 mmol) were converted to isoflavone **20f** (247 mg, 0.54 mmol, 54%); eluent for chromatography: petroleum ether/EtOAc (4:1 (v/v)): yellow amorphous solid; ^1^H NMR (400 MHz, CDCl_3_) δ 7.97 (s, 1H), 7.57 (s, 1H), 6.82 (s, 1H), 6.63 (s, 1H), 5.96 (s, 2H), 5.26 (tm, *J* = 6.9 Hz, 1H), 5.22 (s, 2H), 3.93 (s, 3H), 3.74 (s, 3H), 3.65 (d, *J* = 6.9 Hz, 2H), 3.60 (s, 3H), 1.83 (s, 3H), 1.69 (s, 3H); ^13^C­{^1^H} NMR (100 MHz, CDCl_3_) δ 176.0, 154.3, 153.2, 150.5, 150.2, 148.9, 148.5, 141.3, 132.8, 125.1, 121.6, 121.6, 121.0, 113.2, 111.3, 104.1, 101.5, 99.3, 95.6, 57.9, 57.0, 56.2, 25.9, 23.4, 18.1; IR (ATR) *ν̃* 2925 (w), 1639 (m), 1461 (s), 1425 (s), 1325 (m), 1192 (s), 1168 (m), 1034 (m), 930 (m); HRESIMS *m*/*z* 455.1700 [M + H]^+^ (calcd for C_25_H_27_O_8_, 455.1706).

### General Procedure for the Acid-Catalyzed Cleavage of MOM-Ethers **20**

To a solution of the appropriate MOM-protected isoflavone **20** (0.50 mmol) in MeOH or THF (10 mL), as specified for the individual compounds, HCl (aq., 4 M, 1.5 mmol, 0.375 mL) was added, and the mixture was stirred at 60 °C for 2 h. The mixture was cooled to room temperature and H_2_O (50 mL) was added. The aqueous phase was extracted with EtOAc (3 times, 30 mL each), and the combined organic extracts were dried with anhydrous MgSO_4_, filtered, and evaporated in vacuo. The residue was purified by column chromatography on silica, as specified for the individual compounds, to yield the deprotected isoflavones **21a**–**f**.

#### 7-Hydroxy-3-(4-hydroxyphenyl)-6-methoxy-8-(3-methylbut-2-en-1-yl)-4*H*-chromen-4-one (**21a**)

Following the general procedure, compound **20a** (165 mg, 0.42 mmol) in MeOH (8 mL) was converted to isoflavone **21a** (122 mg, 0.35 mmol, 83%); eluent for chromatography: hexanes/EtOAc (1:1 (v/v)): white powder; IR (ATR) *ν̃* 3290 (s), 2926 (w), 1597 (m), 1513 (m), 1466 (s), 1432 (s), 1286 (m), 1226 (m), 1080 (w), 834 (w); ^1^H NMR (400 MHz, CDCl_3_) δ 8.00 (s, 1H), 7.56 (s, 1H), 7.34 (d, *J* = 8.6 Hz, 2H), 6.82 (d, *J* = 8.6, 2H), 6.43 (s, 1H), 5.27 (tm, *J* = 7.2 Hz, 1H), 3.97 (s, 3H), 3.59 (d, *J* = 7.1, 2H), 1.83 (s, 3H), 1.70 (s, 3H); ^13^C­{^1^H} NMR (100 MHz, CDCl_3_) δ 177.0, 156.4, 152.6, 151.3, 149.0, 145.4, 133.2, 130.5, 124.5, 123.9, 121.0, 117.5, 116.1, 115.8, 102.3, 56.5, 25.9, 22.5, 18.0; HREIMS *m*/*z* 352.1305 [M^+^] (calcd for C_21_H_20_O_5_, 352.1311).

#### 7-Hydroxy-6-methoxy-3-(4-methoxyphenyl)-8-(3-methylbut-2-en-1-yl)-4*H*-chromen-4-one (**21b**)

Following the general procedure, compound **20b** (309 mg, 0.75 mmol) in MeOH (15 mL) was converted to isoflavone **21b** (259 mg, 0.71 mmol, 93%): colorless amorphous solid; eluent for chromatography: petroleum ether/EtOAc (3:1 (v/v)): IR (ATR) *ν̃* 3278 (w), 2928 (w), 1632 (m), 1603 (s), 1512 (m), 1465 (s), 1432 (s), 1290 (m), 1229 (m), 1178 (m), 1033 (w), 831 (w); ^1^H NMR (400 MHz, CDCl_3_) δ 7.99 (s, 1H), 7.55 (s, 1H), 7.52 (d, *J* = 8.7 Hz, 2H), 6.97 (d, *J* = 8.7 Hz, 2H), 6.38 (s, 1H), 5.27 (tm, *J* = 7.2 Hz, 1H), 3.99 (s, 3H), 3.84 (s, 3H), 3.59 (d, *J* = 7.2, 2H), 1.84 (s, 3H), 1.70 (s, 3H); ^13^C­{^1^H} NMR (100 MHz, CDCl_3_) δ 176.1, 159.6, 152.1, 151.0, 148.7, 145.2, 133.1, 130.3, 124.8, 124.0, 121.1, 117.7, 115.7, 114.1, 102.4, 56.5, 55.5, 25.9, 22.5, 18.0; HREIMS *m*/*z* 366.1473 [M^+^] (calcd for C_22_H_22_O_5_, 366.1467). All analytical data match those previously reported for the natural product millesianin H.^62^

#### 3-(3,4-Dimethoxyphenyl)-7-hydroxy-6-methoxy-8-(3-methylbut-2-en-1-yl)-4*H*-chromen-4-one (**21c**)

Following the general procedure, compound **20c** (358 mg, 0.81 mmol) in THF (15 mL) was converted to isoflavone **21c** (271 mg, 0.68 mmol, 84%); eluent for chromatography: hexanes/EtOAc (2:1 (v/v)): colorless solid; IR (ATR) *ν̃* 3300 (w), 2931 (s), 1631 (m), 1604 (m), 1515 (m), 1465 (s), 1433 (s), 1250 (s), 1209 (m), 1227 (w), 849 (w); ^1^H NMR (400 MHz, CDCl_3_) δ 8.03 (s, 1H), 7.56 (s, 1H), 7.25 (d, *J* = 2.1 Hz, 1H), 7.07 (dd, *J* = 8.3, 2.1 Hz, 1H), 6.93 (d, *J* = 8.3 Hz, 1H), 6.38 (s, 1H), 5.27 (tm, *J* = 7.2 Hz, 1H), 3.99 (s, 3H), 3.93 (s, 3H), 3.91 (s, 3H), 3.59 (d, *J* = 7.1, 2H), 1.84 (s, 3H), 1.70 (s, 3H); ^13^C­{^1^H} NMR (100 MHz, CDCl_3_) δ 176.2, 152.3, 151.0, 149.1, 148.9, 148.8, 145.2, 133.1, 125.2, 124.0, 121.1, 121.1, 117.7, 115.8, 112.7, 111.3, 102.3, 56.5, 56.1, 56.1, 25.9, 22.5, 18.0; HREIMS *m*/*z* 396.1586 [M^+^] (calcd for C_23_H_24_O_6_, 396.1573). All analytical data match those previously reported for the natural product predurallone.^63^

#### 3-(Benzo­[*d*]­[1,3]­dioxol-5-yl)-7-hydroxy-6-methoxy-8-(3-methylbut-2-en-1-yl)-4*H*-chromen-4-one (**21d**)

Following the general procedure, compound **20d** (322 mg, 0.76 mmol) in THF (15 mL) was converted to isoflavone **21d** (285 mg, 0.75 mmol, 97%); eluent for chromatography: hexanes/EtOAc (3:1 (v/v)): white powder; IR (ATR) *ν̃* 3315 (w), 2923 (w), 1633 (m), 1605 (m), 1466 (s), 1432 (s), 1291 (m), 1238 (s), 1037 (m), 862 (w); ^1^H NMR (400 MHz, CDCl_3_) δ 7.98 (s, 1H), 7.54 (s, 1H), 7.12 (d, *J* = 1.7 Hz, 1H), 6.99 (dd, *J* = 8.0, 1.7 Hz, 1H), 6.87 (d, *J* = 8.0 Hz, 1H), 6.39 (s, 1H), 5.99 (s, 2H), 5.27 (tm, *J* = 7.1 Hz, 1H), 4.00 (s, 3H), 3.58 (d, *J* = 7.1, 2H), 1.83 (s, 3H), 1.70 (s, 3H); ^13^C­{^1^H} NMR (100 MHz, CDCl_3_) δ 176.0, 152.3, 151.0, 148.8, 147.8, 147.6, 145.2, 133.1, 126.2, 124.1, 122.5, 121.1, 117.7, 115.8, 109.9, 108.5, 102.4, 101.3, 56.5, 25.9, 22.5, 18.0; HREIMS *m*/*z* 380.1273 [M^+^] (calcd for C_22_H_20_O_6_, 380.1260). All analytical data match those previously reported for the natural product predurmillone.^64^ No ^13^C NMR data have been reported in the isolation study.

#### 7-Hydroxy-6-methoxy-8-(3-methylbut-2-en-1-yl)-3-(2,4,5-trimethoxyphenyl)-4*H*-chromen-4-one (**21e**)

Following the general procedure, compound **20e** (233 mg, 0.50 mmol) in THF (10 mL) was converted to isoflavone **21e** (186 mg, 0.44 mmol, 88%); eluent for chromatography: hexanes/EtOAc (1:2 (v/v)): white amorphous solid; IR (ATR) *ν̃* 3357 (w), 2936 (w), 1633 (m), 1606 (m), 1512 (m), 1464 (s), 1434 (m), 1322 (m), 1204 (s), 1148 (m), 1031 (m); ^1^H NMR (400 MHz, CDCl_3_) δ 8.02 (s, 1H), 7.55 (s, 1H), 6.96 (s, 1H), 6.63 (s, 1H), 6.38 (s­(br), 1H), 5.28 (tm, *J* = 6.2 Hz, 1H), 3.98 (s, 3H), 3.93 (s, 3H), 3.85 (s, 3H), 3.79 (s, 3H), 3.59 (d, *J* = 6.2 Hz, 2H), 1.83 (s, 3H), 1.70 (s, 3H); ^13^C­{^1^H} NMR (100 MHz, CDCl_3_) δ 176.1, 154.3, 152.0, 151.0, 149.8, 148.6, 145.1, 143.2, 133.0, 121.1, 121.0, 117.7, 115.7, 115.4, 112.7, 102.4, 98.4, 57.1, 56.7, 56.5, 56.3, 25.9, 22.5, 18.0; HRESIMS *m*/*z* 427.1767 [M + H]^+^ (calcd for C_24_H_27_O_7_, 427.1757). All analytical data match those previously reported for the natural product millesianin I^62^ (pachyloisoflavone A^65^).

#### 7-Hydroxy-6-methoxy-3-(6-methoxybenzo­[*d*]­[1,3]­dioxol-5-yl)-8-(3-methylbut-2-en-1-yl)-4*H*-chromen-4-one (**21f**)

Following the general procedure, compound **20f** (247 mg, 0.54 mmol) in THF (10 mL) was converted to isoflavone **21f** (217 mg, 0.53 mmol, 98%); eluent for chromatography: hexanes/EtOAc (2:1 (v/v)): white amorphous solid; IR (ATR) *ν̃* 3462 (m), 2912 (w), 1640 (m), 1610 (m), 1465 (s), 1432 (s), 1191 (s), 1105 (w), 1033 (m); ^1^H NMR (400 MHz, CDCl_3_) δ 7.95 (s, 1H), 7.53 (s, 1H), 6.83 (s, 1H), 6.63 (s, 1H), 6.38 (s­(br), 1H), 5.95 (s, 2H), 5.28 (tm, *J* = 7.1 Hz, 1H), 3.98 (s, 3H), 3.74 (s, 3H), 3.58 (d, *J* = 7.1 Hz, 2H), 1.83 (s, 3H), 1.70 (s, 3H); ^13^C­{^1^H} NMR (100 MHz, CDCl_3_) δ 176.0, 154.0, 153.2, 151.0, 148.6, 148.5, 145.1, 141.3, 133.0, 121.4, 121.1, 117.7, 115.7, 113.4, 111.4, 102.5, 101.5, 95.6, 57.1, 56.5, 25.9, 22.5, 18.0; HRESIMS *m*/*z* 411.1458 [M + H]^+^ (calcd for C_23_H_23_O_7_, 411.1444). All analytical data match those previously reported for the natural product millesianin D.^62,66,67^

### General Procedure for the *O*-Methylation of Isoflavones **21**

To a refluxing solution (56 °C) of isoflavones **21** (0.50 mmol) was added K_2_CO_3_ (138 mg, 1.00 mmol) in acetone or DMF (5 mL), as indicated for the individual example. Dimethyl sulfate (63 mg, 0.50 mmol) was added, and the mixture was heated at 56 °C for 5 h. It was then cooled to ambient temperature, filtered, and evaporated in vacuo. The residue was purified by column chromatography on silica, as specified for the individual compounds, to yield the 7-methoxyisoflavones **22b**–**f**.

#### 6,7-Dimethoxy-3-(4-methoxyphenyl)-8-(3-methylbut-2-en-1-yl)-4*H*-chromen-4-one (**22b**)

Following the general procedure, compound **21b** (157 mg, 0.43 mmol) in acetone (5 mL) was converted to isoflavone **22b** (161 mg, 0.42 mmol, 98%); eluent for chromatography: petroleum ether/EtOAc (2:1 (v/v)): yellow oil; IR (ATR) *ν̃* 2932 (w), 1638 (s), 1601 (m), 1512 (s), 1461 (s), 1425 (s), 1287 (m), 1227 (m), 1178 (m), 1053 (w), 832 (w); ^1^H NMR (400 MHz, CDCl_3_) δ 8.02 (s, 1H), 7.59 (s, 1H), 7.52 (d, *J* = 8.8 Hz, 2H), 6.98 (d, *J* = 8.8 Hz, 2H), 5.21 (tm, *J* = 7.0 Hz, 1H), 3.96 (s, 3H), 3.93 (s, 3H), 3.85 (s, 3H), 3.59 (d, *J* = 7.0, 2H), 1.84 (s, 3H), 1.69 (s, 3H); ^13^C­{^1^H} NMR (100 MHz, CDCl_3_) δ 176.2, 159.7, 152.4, 152.0, 151.2, 150.1, 132.8, 130.2, 124.8, 124.6, 124.1, 121.7, 120.9, 114.1, 104.0, 61.3, 56.2, 55.5, 25.9, 23.1, 18.0; HREIMS *m*/*z* 380.1620 [M^+^] (calcd for C_23_H_24_O_5_, 380.1624). All analytical data match those previously reported for the natural product pachyvone A.^68^

#### 3-(3,4-Dimethoxyphenyl)-6,7-dimethoxy-8-(3-methylbut-2-en-1-yl)-4*H*-chromen-4-one (**22c**)

Following the general procedure, compound **21c** (169 mg, 0.43 mmol) in acetone (5 mL) was converted to isoflavone **22c** (160 mg, 0.39 mmol, 90%); eluent for chromatography: hexanes/EtOAc (2:1 (v/v)): colorless crystals, mp 95–96 °C; IR (ATR) *ν̃* 2932 (m), 1636 (s), 1600 (m), 1515 (s), 1461 (s), 1425 (s), 1262 (m), 1027 (w); ^1^H NMR (400 MHz, CDCl_3_) δ 8.05 (s, 1H), 7.60 (s, 1H), 7.25 (d, *J* = 2.1 Hz, 1H), 7.07 (dd, *J* = 8.3, 2.1 Hz, 1H), 6.93 (d, *J* = 8.3 Hz, 1H), 5.21 (tm, *J* = 7.1 Hz, 1H), 3.96 (s, 3H), 3.93 (s, 3H), 3.93 (s, 3H), 3.92 (s, 3H), 3.59 (d, *J* = 7.1, 2H), 1.84 (s, 3H), 1.69 (s, 3H); ^13^C­{^1^H} NMR (100 MHz, CDCl_3_) δ 176.3, 152.6, 152.1, 151.3, 150.1, 149.2, 148.9, 132.8, 125.0, 124.8, 124.0, 121.6, 121.1, 120.9, 112.6, 111.3, 103.9, 61.3, 56.2, 56.1, 56.1, 25.9, 23.1, 18.0; HREIMS *m*/*z* 410.1733 [M^+^] (calcd for C_24_H_26_O_6_, 410.1729).

#### 3-(Benzo­[*d*]­[1,3]­dioxol-5-yl)-6,7-dimethoxy-8-(3-methylbut-2-en-1-yl)-4*H*-chromen-4-one (**22d**)

Following the general procedure, compound **21d** (180 mg, 0.47 mmol) in DMF (5 mL) was converted to isoflavone **22d** (175 mg, 0.44 mmol, 94%); eluent for chromatography: hexanes/EtOAc (6:1 (v/v)): yellow solid; IR (ATR) *ν̃* 2928 (w), 1635 (s), 1599 (m), 1460 (m), 1423 (s), 1285 (m), 1231 (s), 1037 (m), 810 (w); ^1^H NMR (400 MHz, CDCl_3_) δ 8.00 (s, 1H), 7.58 (s, 1H), 7.12 (d, *J* = 1.7 Hz, 1H), 6.99 (dd, *J* = 8.0, 1.7 Hz, 1H), 6.87 (d, *J* = 8.0 Hz, 1H), 5.99 (s, 2H), 5.20 (tm, *J* = 7.0 Hz, 1H), 3.96 (s, 3H), 3.93 (s, 3H), 3.59 (d, *J* = 7.0, 2H), 1.83 (s, 3H), 1.69 (s, 3H); ^13^C­{^1^H} NMR (100 MHz, CDCl_3_) δ 176.1, 152.6, 152.1, 151.3, 150.1, 147.8, 147.7, 132.8, 126.1, 124.8, 124.2, 122.5, 121.6, 120.8, 109.9, 108.5, 104.0, 101.3, 61.3, 56.2, 25.9, 23.1, 18.0; HREIMS *m*/*z* 394.1416 [M^+^] (calcd for C_23_H_22_O_6_, 394.1416). All analytical data match those previously reported for the natural product pachyvone B.^68,69^

#### 6,7-Dimethoxy-8-(3-methylbut-2-en-1-yl)-3-(2,4,5-trimethoxyphenyl)-4*H*-chromen-4-one (**22e**)

Following the general procedure, compound **21e** (123 mg, 0.29 mmol) in DMF (5 mL) was converted to isoflavone **22e** (128 mg, 0.29 mmol, quant.); eluent for chromatography: hexanes/EtOAc (1:1 (v/v)): white powder; IR (ATR) *ν̃* 2934 (w), 1638 (m), 1601 (m), 1512 (m), 1459 (s), 1425 (s), 1379 (w), 1315 (m), 1280 (w), 1203 (s), 1148 (m), 1031 (m); ^1^H NMR (400 MHz, CDCl_3_) δ 8.05 (s, 1H), 7.59 (s, 1H), 6.95 (s, 1H), 6.63 (s, 1H), 5.22 (tm, *J* = 6.9 Hz, 1H), 3.95 (s, 3H), 3.93 (s, 3H), 3.92 (s, 3H), 3.85 (s, 3H), 3.78 (s, 3H), 3.59 (d, *J* = 6.9 Hz, 2H), 1.83 (s, 3H), 1.69 (s, 3H); ^13^C­{^1^H} NMR (100 MHz, CDCl_3_) δ 176.2, 154.6, 152.0, 152.0, 151.1, 150.1, 149.9, 143.2, 132.7, 124.8, 121.7, 121.1, 120.9, 115.4, 112.6, 104.0, 98.4, 61.2, 57.0, 56.7, 56.3, 56.2, 25.9, 23.1, 18.0; HRESIMS *m*/*z* 441.1909 [M + H]^+^ (calcd for C_25_H_29_O_7_, 441.1913). All analytical data match those previously reported for the natural product pachyvone C^68^ (placoisoflavone A^70^).

#### 6,7-Dimethoxy-3-(6-methoxybenzo­[*d*]­[1,3]­dioxol-5-yl)-8-(3-methylbut-2-en-1-yl)-4*H*-chromen-4-one (**22f**)

Following the general procedure, compound **21f** (123 mg, 0.30 mmol) in DMF (5 mL) was converted to isoflavone **22f** (118 mg, 0.28 mmol, 93%); eluent for chromatography: hexanes/EtOAc (9:1 (v/v)): off white amorphous solid; IR (ATR) *ν̃* 2925 (s), 1635 (s), 1599 (m), 1505 (s), 1456 (s), 1421 (s), 1327 (m), 1263 (m), 1192 (s), 1170 (m); ^1^H NMR (400 MHz, CDCl_3_) δ 7.97 (s, 1H), 7.57 (s, 1H), 6.82 (s, 1H), 6.63 (s, 1H), 5.96 (s, 2H), 5.22 (tm, d, *J* = 7.0 Hz, 1H), 3.95 (s, 3H), 3.92 (s, 3H), 3.74 (s, 3H), 3.59 (d, *J* = 7.0 Hz, 2H), 1.83 (s, 3H), 1.69 (s, 3H); ^13^C­{^1^H} NMR (100 MHz, CDCl_3_) δ 176.0, 154.3, 153.2, 151.9, 151.1, 150.1, 148.5, 141.3, 132.7, 124.8, 121.7, 121.5, 120.9, 113.2, 111.3, 104.1, 101.5, 95.6, 61.2, 57.0, 56.2, 25.9, 23.1, 18.0; HRESIMS *m*/*z* 425.1606 [M + H]^+^ (calcd for C_24_H_25_O_7_, 425.1600). All analytical data match those previously reported for the natural product 8-prenylmilldurone.^68,71^

#### 3-(Benzo­[*d*]­[1,3]­dioxol-5-yl)-6-methoxy-8-(3-methylbut-2-en-1-yl)-7-((3-methylbut-2-en-1-yl)­oxy)-4*H*-chromen-4-one (**23**)

To a solution of KHMDS (200 mg, 1.00 mmol) in THF (10 mL) at −78 °C was added **21d** (210 mg, 0.60 mmol) in THF (10 mL) over 25 min. The mixture was stirred for 15 min. Prenyl bromide (127 μL, 1.00 mmol) in dry THF (2 mL) was then added over 12 min. The temperature was maintained at −78 °C for 4 h. The mixture was then allowed to warm up to ambient temperature, and stirred for 12 h. The reaction was quenched with aq. HCl (1 M, 6 mL) and H_2_O (50 mL) was added. The aqueous phase was extracted with EtOAc (3 times, 30 mL each), the combined organic layers were washed with brine and dried with anhydrous MgSO_4_, filtered, evaporated in vacuo, and purified by column chromatography using petroleum ether/EtOAc to yield **23** (185 mg, 0.41 mmol, 75%): yellow oil; IR (ATR) *ν̃* 2912 (w), 1636 (m), 1460 (s), 1425 (s), 1285 (m), 1225 (m), 1037 (m), 810 (w); ^1^H NMR (400 MHz, CDCl_3_) δ 8.00 (s, 1H), 7.57 (s, 1H), 7.12 (d, *J* = 1.7 Hz, 1H), 6.99 (dd, *J* = 8.0, 1.7 Hz, 1H), 6.87 (d, *J* = 8.0 Hz, 1H), 5.99 (s, 2H), 5.54 (tm, *J* = 7.3 Hz, 1H), 5.20 (tm, *J* = 7.0 Hz, 1H), 4.63 (d, *J* = 7.3 Hz, 2H), 3.96 (s, 3H), 3.59 (d, *J* = 7.0, 2H), 1.82 (s, 3H), 1.77 (s, 3H), 1.70 (s, 3H), 1.69 (s, 3H); ^13^C­{^1^H} NMR (100 MHz, CDCl_3_) δ 176.1, 152.6, 151.4, 151.0, 150.2, 147.8, 147.7, 139.0, 132.6, 126.1, 125.2, 124.2, 122.5, 121.8, 120.7, 120.3, 109.9, 108.5, 103.7, 101.3, 70.0, 56.2, 26.0, 25.9, 23.3, 18.1, 18.0; HREIMS *m*/*z* 449.1954 [M + H]^+^ (calcd for C_27_H_29_O_6_, 449.1964).

### X-ray Crystallographic Data

Compound **22c** crystallized from hexanes-EtOAc solution as colorless blocks. Crystallographic data were collected at 293 K on a Stoe imaging plate diffraction system Stadivari, using Mo Kα radiation (λ = 0.71073 Å). The data correction was performed using the program X-Area.^85^ The structure was solved by direct methods and refined against *F* ^2^ on all data by full-matrix least-squares using the SHELX suite of programs.^86,87^ All nonhydrogen atoms were refined anisotropically; the hydrogen atoms were placed on calculated positions. The crystal structure was visualized with Mercury.^88^

The crystallographic data can be obtained free of charge via http://www.ccdc.cam.ac.uk/structures/ or from The Cambridge Crystallographic Data Center, 12 Union Road, Cambridge, CB2 1EZ, UK; fax (+44)­1223-336-033; or via e-mail: deposit@ccdc.cam.ac; deposit number: CCDC 2435363.

Data for compound **22c**: C_24_H_26_O_6_, *M* = 410.45 g/mol, 0.400 × 0.467 × 0.500 mm^3^, triclinic, space group *P*1̅ (no. 2), *a* = 8.2455(16) Å, *b* = 11.884(2) Å, *c* = 12.351(3) Å, α = 103.88(3)°, β = 108.84(3)°, γ = 99.35(3)°, *V* = 1073.4(4) Å^3^, *Z* = 2, *D*_c_ = 1.270 g/cm^3^, 65585 reflections measured (6.26° ≤ 2θ ≤ 66.02°), 7656 unique (*R*_int_ = 0.0306, *R*_sigma_ = 0.0172), which were used in all calculations. The final *R*_1_ was 0.0493 (*I* > 2σ­(*I*)) and *wR*_2_ was 0.1588 (all data).

### In Vitro α-Amylase Assay Using the Enzyme from Porcine Pancreas

The activity of porcine pancreatic α-amylase (Sigma-Aldrich, Darmstadt, Germany, #A3176) was determined spectrophotometrically using the chromogenic substrate 2-chloro-4-nitrophenyl-d-maltotrioside (CNPG3; Carl Roth, Karlsruhe, Germany, #2C2X.3) as described by Zulfiqar et al.^72^ Prenylated isoflavones were first dissolved in DMSO to prepare 500 μM stock solutions. For each assay, a mixture of 20 μL of the test compound or 20 μL DMSO (solvent control), 30 μL H_2_O and 100 μL enzyme solution (5 U/mL α-amylase in 20 mM sodium phosphate buffer, 200 mM NaCl, pH 6.8) was preincubated for 10 min at 37 °C, before adding 50 μL of 2 mM CNPG3 in 20 mM sodium phosphate buffer. Enzyme activity was monitored by measuring changes in absorbance at 405 nm, corresponding to the release of *p*-nitrophenol as the reaction product. Absorbance readings were recorded at 1 min intervals over a 10 min period at 37 °C using a plate reader (Varioskan LUX, Thermo Fisher Scientific, Darmstadt, Germany). 50 μM Acarbose (LKT Laboratories, St. Paul, MN, USA, #A0802) served as positive control. Enzyme inhibition was calculated from blank-corrected absorbance values by using the following equation:

$$\mathrm{Inhibition}\left(\%\right)=\frac{\left[\left(\mathrm{AbC}-{\mathrm{AbC}}_{\mathrm{blank}}\right)-\left(\mathrm{AbS}-{\mathrm{AbS}}_{\mathrm{blank}}\right)\right]}{\left(\mathrm{AbC}-{\mathrm{AbC}}_{\mathrm{blank}}\right)}\times 100$$

AbC, absorbance of the control; AbS, absorbance of the sample.

### In Vitro α-Glucosidase Assay Using the *Saccharomyces cerevisiae* Enzyme

The activity of α-glucosidase (MAL12, MAL32) from baker’s yeast (Sigma-Aldrich, #G5003) was determined using *p*-nitrophenyl-α-d-glucopyranoside (4-NPG; Sigma-Aldrich, #N1377) as substrate.^89^ 15 μL of the test compounds dissolved in DMSO (final concentration as indicated) or 15 μL DMSO (solvent control) were pipetted to 105 μL sodium phosphate buffer (pH 6.8; NaPi) and 15 μL enzyme solution (0.5 U/mL in NaPi). Acarbose (300 μM) served as positive control. If not stated otherwise, the mixtures were preincubated for 5 min at 37 °C, before the reactions were initiated by the addition of 15 μL prewarmed (37 °C) 4-NPG, which has a final concentration of 1 mM in the standard assay. After 15 min at 37 °C, reactions were terminated with 50 μL 2 M Na_2_CO_3_, and the release of *p*-nitrophenol (*t*_15_ values) was measured at 405 nm in 96-well microplates using a multimode microplate reader. Baseline absorbance (*t*_0_) was determined in parallel reactions by adding Na_2_CO_3_ prior to substrate addition. Enzyme activity was calculated from the difference between *t*_15_ and *t*_0_ absorbance values.

Specific enzyme activity was calculated using the molar extinction coefficient for *p*-nitrophenol (ε = 18 cm^–1^·mM^–1^). IC_50_ values and activation curves were determined in the presence of 1 mM 4-NPG and with prenylated isoflavone concentrations ranging from 0 to 200 μM. To assess the kinetic parameters *K*_m_ and *V*_max_, substrate concentrations range from 0–4 mM 4-NPG. For determination of *K*_i_ values, selected isoprenylated isoflavones were tested at concentrations ranging from 0 to 50 μM. All kinetic analyses were performed using GraphPad Prism software.

### Molecular Docking Studies

The molecular docking calculations were performed using AutoDock 4.2.6 Vina software^90^ against proteins of α-glucosidase Mal32 (PDB: AFP38158F1) and α-glucosidase (PDB: 3AJ7). We followed the same methodologies as in previous related studies.^91,92^ The MGL 1.5.6 program was used to remove the cocrystallized substrate and water molecules from the receptor. Polar hydrogens and Kollman charges were added to the cleaned protein. After merging nonpolar hydrogen atoms, Gasteiger partial atomic charges were assigned to all atoms. The grid box for the α-glucosidase (PDB: 3AJ7) was constructed using 80, 80, and 80 pointing in the *x*, *y*, and *z* directions, respectively, with a grid point spacing of 0.375 Å. The center grid box was set at 21.342, −0.264, and 18.691 Å for the *x*, *y*, and *z* centers, respectively. On the other hand, for the α-glucosidase Mal32 (PDB: AFP38158F1) it was constructed using 46, 46, and 56 pointing in the *x*, *y*, and *z* directions, respectively, with a grid point spacing of 0.375 Å, and the center grid box was set at −11.631, 7.280, and 2.476 Å for the *x*, *y*, and *z* centers, respectively. To ensure that the lowest energy conformer is used for the visualization, hundred alternative conformations for each molecule with an adaptive whole method search was employed using the Lamarckian genetic algorithm (LGA) in AutoDock Vina. The Discovery Studio program was used to visualize the interaction of the compound with the lowest binding free energy with the amino acids of the protein.

## Supplementary Material



## Data Availability

Primary NMR FID files for compounds **3e**, **3f**, **5**–**12**, **14**–**16**, **18**, **19**, **20a**–**f**, **21a**–**f**, **22b**–**f**, and **23** (ZIP) are available via the Zenodo data repository at 10.5281/zenodo.17791931.

## Notes

Chemical synthesis procedures and analytical data for compounds **3e**, **3f**, **5**–**12**, **14**–**16**, **18**, **19**; enzyme kinetic parameters for **21c** and **22c**; copies of ^1^H and ^13^C­{^1^H} NMR spectra of all compounds; NMR-data comparison tables for natural products **21b**–**f**, **22b**, **22d**–**f**; crystallographic details and refinement data for **22c** (PDF)
