# Supplementary material for: Prenylated Isoflavones Originating from East African Medicinal Plants Selectively Modulate the α‑Glucosidase from Saccharomyces cerevisiae
Source: J Nat Prod. 2026 May 8;89(5):1610–24. doi: 10.1021/acs.jnatprod.6c00411 (PMC13200251; doi:10.1021/acs.jnatprod.6c00411)
Supplement: Supplementary file 1 [file np6c00411_si_001.pdf]

Supporting Information

Prenylated Isoflavones originating from East African Medicinal Plants Selectively Modulate the α-Glucosidase from *Saccharomyces cerevisiae*

Kai Lüersen[‡], Friederike Neuber[‡], Eric Sperlich,[†] Alexandra Kelling[†], Sarah von Chamier-Gliszczinski[†], Julia Greese[†], Taye B. Demissie[§], Bernd Schmidt[†], Gerald Rimbach[‡], and Vaderament-Alexe Nchiozem-Ngnitedem[†*]*

[‡]Institute of Human Nutrition and Food Science, University of Kiel, D-24118 Kiel, Germany

[†]University of Potsdam, Institut für Chemie, Karl-Liebknecht-Strasse 24-25, D-14476 Potsdam-Golm, Germany

[§]Department of Chemistry, Faculty of Science, University of Botswana, Gaborone, Botswana

*e-mail: nchiozem-ngnitedem@uni-potsdam.de (Vaderament-Alexe Nchiozem-Ngnitedem); luersen@foodsci.uni-kiel.de (Kai Lüersen)

Contents:

**A**	**Single crystal X-ray structure analysis of compound 22c**	**S2**
**B**	**Kinetic parameters for activator compounds 21c and 22c and modulatory effects of isoflavones on α-glucosidase and α-amylase**	**S7**
**C**	**Structure Block: Isoflavones with inhibitory activity of *S. cerevisiae* α-glucosidase**	**S8**
**D**	**Chemical Syntheses of 3-Iodochromone 12 and boronic acids 3**	**S9**
**E**	**Copies of NMR-spectra and NMR-data for comparison**	**S16**
**F**	**References**	**S96**

A Single crystal X-ray structure analyses of compound 22c

1 General details of X-ray structure analysis

The crystal structure was determined by single crystal structure analysis. Suitable single crystals were selected using a Leica M205C light microscope and separated with oil. X-ray crystal structure analysis was performed on a Stadivari diffractometer (Stoe) with monochromated Mo-*K*α radiation (λ = 0.71073 Å). The data correction was performed using the program X-Area.[1] The structure was solved by direct methods and refined against F^2 on all data by full-matrix least-squares using the SHELX suite of programs.[2] All non-hydrogen atoms were refined anisotropically; the hydrogen atoms were placed on calculated positions. **Table S1** was created using FinalCif.[3] The crystal structure was visualized with Mercury.[4] The data (**22c**: CCDC 2435363) can be obtained free of charge from the Cambridge Crystallographic Data Centre, http://www.ccdc.cam.ac.uk.

2 Crystallographic Data:

Table S1. Crystal data and details of structure refinement for **22c**.

Compound	**22c**
CCDC number	2435363
Empirical formula	$C_{24}H_{26}O_6$
Formula weight	410.45
Temperature [K]	293
Crystal system	triclinic
Space group (number)	$P\overline{1}$ (2)
a [Å]	8.2455(16)
b [Å]	11.884(2)
c [Å]	12.351(3)
α [°]	103.88(3)
β [°]	108.84(3)
γ [°]	99.35(3)
Volume [Å^3]	1073.4(4)
Z	2
ρ_{calc} [gcm^{-3}]	1.270
μ [mm^{-1}]	0.090
F(000)	436
Crystal size [mm^3]	0.400×0.467×0.500
Crystal colour	colorless
Crystal shape	block
Radiation	MoK_α (λ=0.71073 Å)
2θ range [°]	6.26 to 66.02 (0.65 Å)
Index ranges	$-12 \leq h \leq 12$<br>$-18 \leq k \leq 17$<br>$-18 \leq l \leq 18$
Reflections collected	65585
Independent reflections	7656<br>R_{int} = 0.0306<br>R_{sigma} = 0.0172
Completeness to θ = 25.242°	99.7 %
Data / Restraints / Parameters	7656 / 0 / 277
Goodness-of-fit on F^2	1.122
Final R indexes [$I \geq 2\sigma(I)$]	R_1 = 0.0493<br>wR_2 = 0.1588
Final R indexes [all data]	R_1 = 0.0742<br>wR_2 = 0.1735
Largest peak/hole [eÅ^{-3}]	0.27/−0.20

3 Visualization of the crystal structure and molecular structure for compound 22c

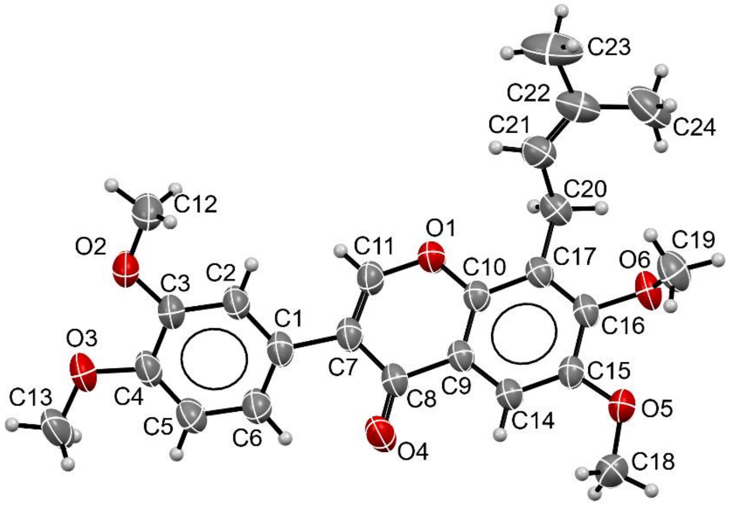

Figure S1: Molecular structure with atom labeling of compound **22c**. Displacement ellipsoids are shown at the 50% probability level.

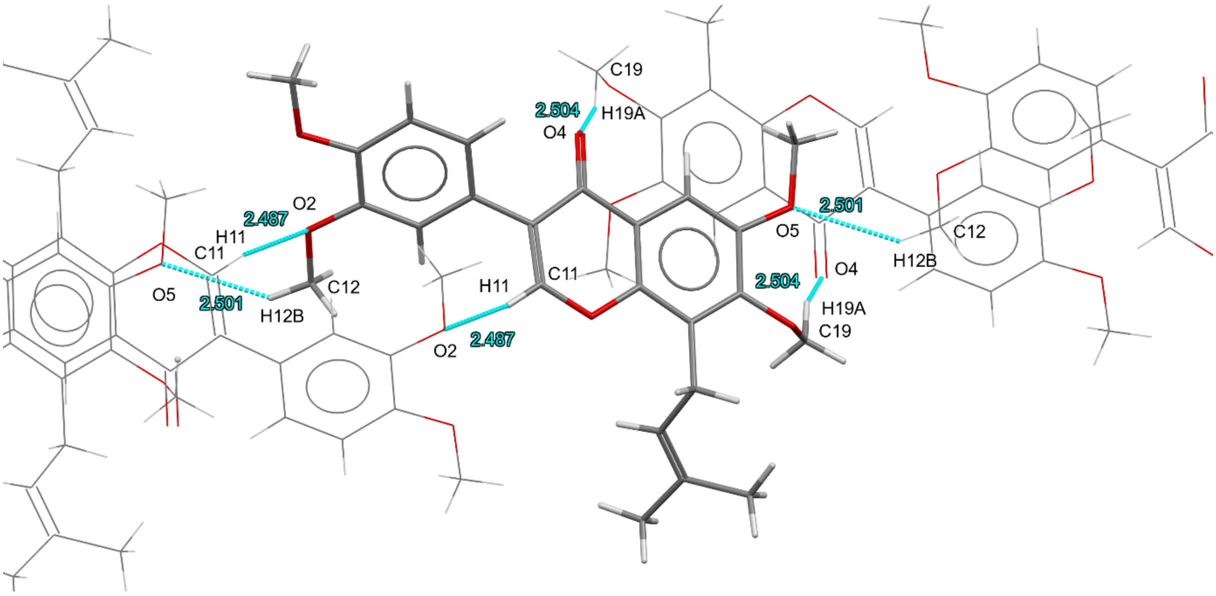

Figure S2: All C-H···O hydrogen bonds in **22c** with A-H distances up to 2.6 Å (blue dashed lines) and D-H-A angles of at least 120°.

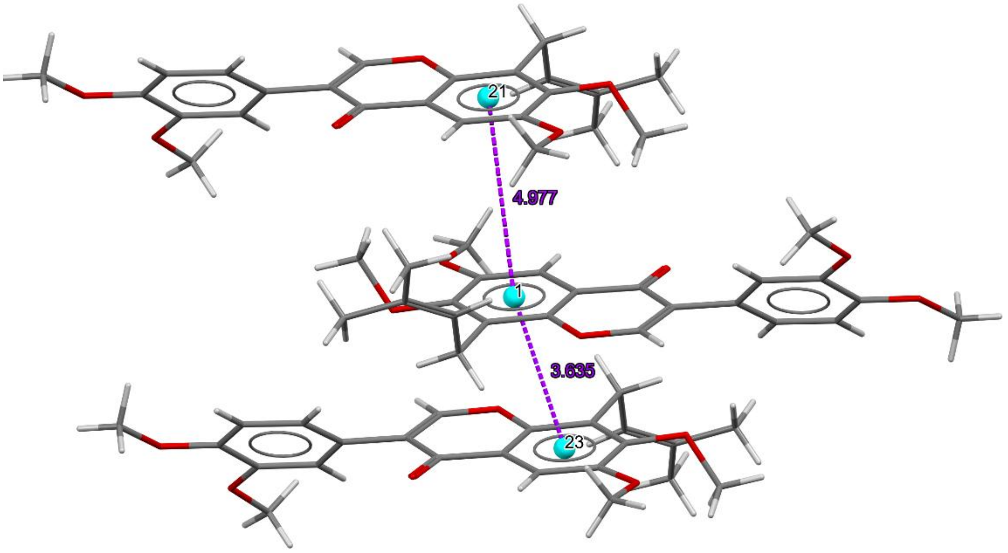

Figure S3: Strong parallel-displaced stacking interactions (purple dotted lines) between the molecules in **22c**.

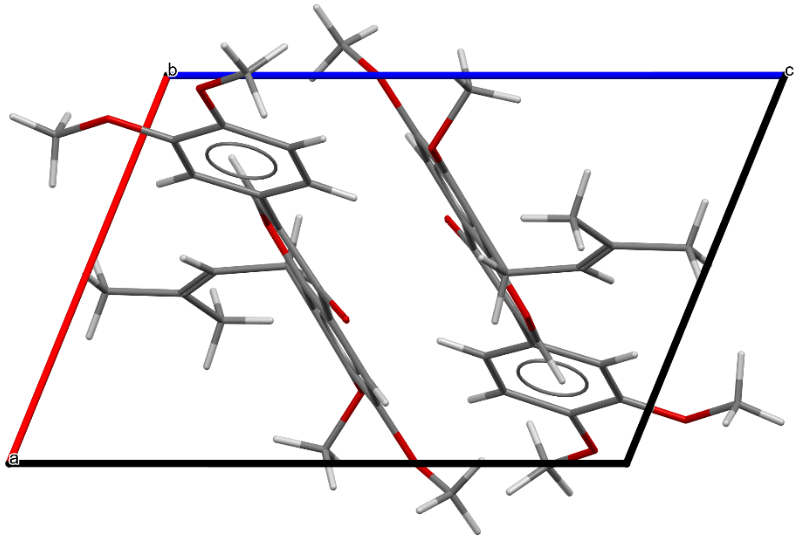

Figure S4: Cell view of compound **22c** looking along the crystallographic b axis.

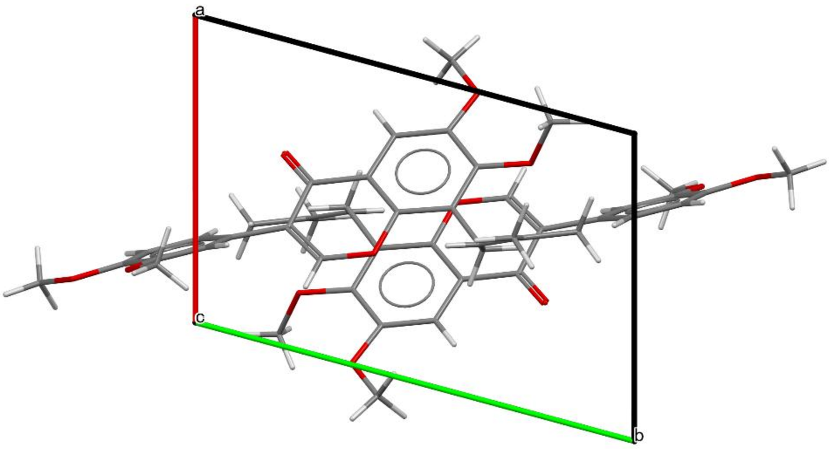

Figure S5: Cell view of compound **22c** looking along the crystallographic c axis.

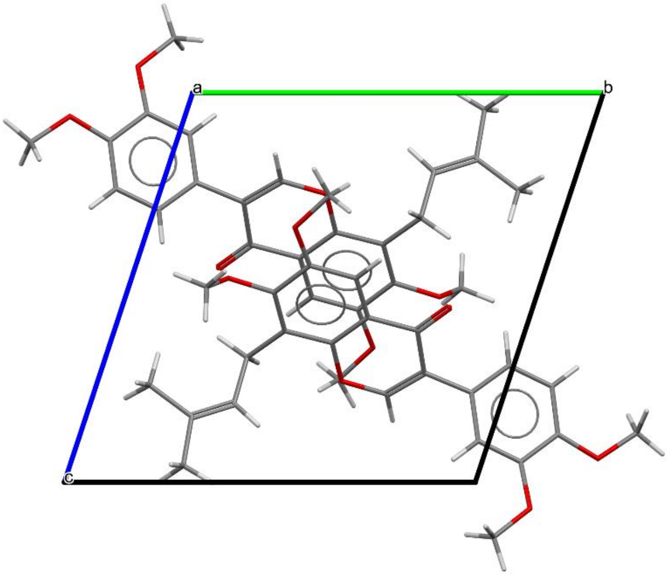

Figure S6: Cell view of compound **22c** looking along the crystallographic a axis.

B Kinetic parameters for activator compounds 21c and 22c and modulatory effects of isoflavones on α-glucosidase and α-amylase

Table S2. Compound **21c** and **22c** increased the velocity of the *S. cerevisiae* α-glucosidase reaction without altering the substrate affinity of the enzyme. Michaelis-Menten kinetics were determined in the presence of 0-100 µM of the activator compounds **21c** and **22c** (see also **Figure 5H**, **I**). Data are the mean ± SD of three independent experiments.

**Activator**	**21c**		**22c**	
	V_{max} [U/mg]	K_m [mM]	V_{max} [U/mg]	K_m [mM]
**Control**	9.3 ± 0.5	0.62 ± 0.04	10.3 ± 0.3	0.60 ± 0.01
**10 µM**	10.1 ± 0.4	0.69 ± 0.09	10.8 ± 0.6	0.61 ± 0.05
**50 µM**	10.8 ± 0.7	0.62 ± 0.06	12.2 ± 0.1	0.59 ± 0.04
**100 µM**	11.9 ± 0.4	0.70 ± 0.02	12.8 ± 0.5	0.57 ± 0.02

Table S3. The modulatory effects of prenylated isoflavones on α-glucosidase and α-amylase. The impact of prenylated isoflavones (each tested at 50 µM) on the enzyme activity of the α-glucosidase from *Saccharomyces cerevisiae* and porcine pancreatic α-amylase was calculated as percentage relative to the corresponding control enzyme activity, with negative values indicating inhibition and positive values activation. The reference inhibitor acarbose was added at 50 µM and 500 µM in the α-amylose and α-glucosidase assay, respectively. All data are presented as mean ± SD of three independent determinations.

	**Relative α-glucosidase activity (% Modulation)**	**Relative α-amylase activity (% Modulation)**
**Acarbose**	-44.7 ± 1.1	-94.5 ± 0.3
**21a**	-27.1 ± 13.6	0.4 ± 3.2
**21b**	28.3 ± 18.2	0.7 ± 3.3
**21c**	37.8 ± 23.2	0.7 ± 3.1
**21d**	-5.4 ± 5.4	-0.6 ± 3.0
**21e**	17.6 ± 21.3	-1.1 ± 3.0
**21f**	-6.3 ± 7.3	-0.3 ± 3.4
**22b**	9.1 ± 9.4	0.1 ± 0.9
**22c**	37.1 ± 3.5	-2.5 ± 3.2
**22d**	-6.3 ± 6.3	-1.3 ± 2.4
**22e**	19.4 ± 8.7	-1.2 ± 3.1
**22f**	-2.1 ± 3.3	-1.6 ± 3.0
**23**	-7.3 ± 1.4	-2.8 ± 2.9
**24a**	-15.9 ± 5.3	-2.7 ± 2.9
**24b**	-29.5 ± 31.2	-3.3 ± 4.3
**24c**	-25.9 ± 14.0	1.6 ± 0.2
**24d**	-6.5 ± 7.5	-1.0 ± 1.3
**24e**	-5.3 ± 7.7	-0.7 ± 2.1
**24f**	-29.3 ± 10.9	-1.4 ± 1.6
**25a**	-3,0 ± 7.8	-0.5 ± 1.7
**25b**	-6.2 ± 6.8	-0.5 ± 1.7
**26**	-73.9 ± 11.2	-7.8 ± 2.3
**27**	-15.6 ± 6.0	-0.3 ± 2.0
**28**	-66.5 ± 24.0	2.8 ± 1.8

C Isoflavones with inhibitory activity of *S. cerevisiae* α-glucosidase previously reported in the literature (illustration for comparative discussion of inhibitory activity of compounds 26 and 28)

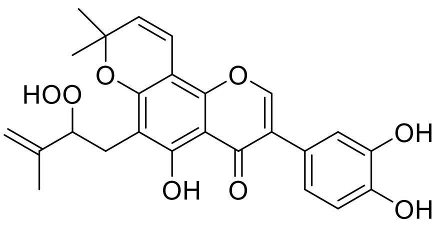

Macluraisoflavone I

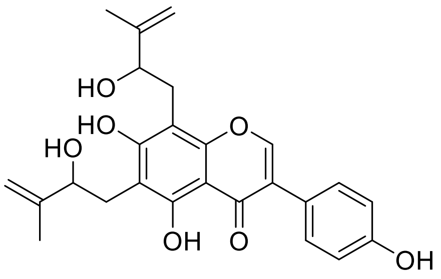

Cudracusisoflavone L

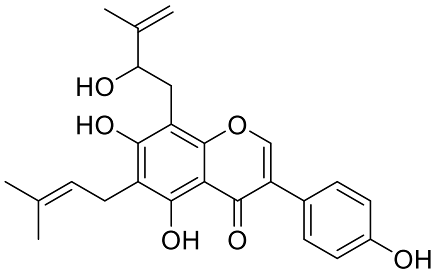

Erysenegalensein E

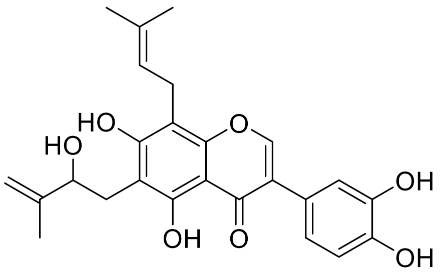

Millewanin G

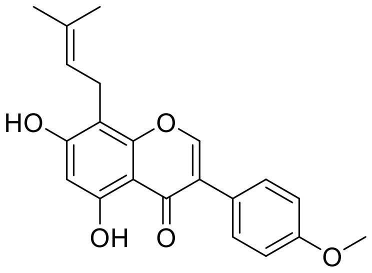

Gancaonin M

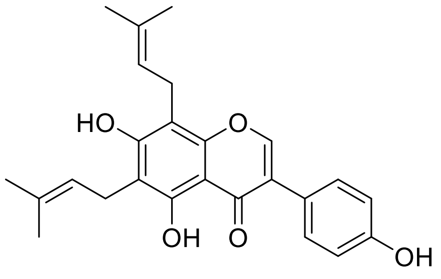

6,8-Diprenylgenistein

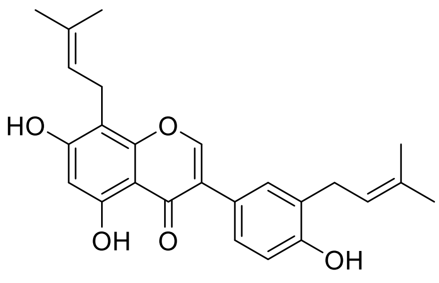

Isolupalbigenin

D Chemical Syntheses of 3-Iodochromone 12 and boronic acids 3.

General Experimental Procedures. All syntheses were conducted in dry reaction vessels under an atmosphere of dry nitrogen. Solvents were purified by standard procedures. Unless otherwise stated, reaction mixtures were heated with silicon oil baths. Microwave reactions were carried out in an Anton-Paar-monowave 300 or Anton-Paar-monowave 400 reactor (monowave, maximum power 850 W, temperature control by IR-sensor, vial volume 20 mL). All NMR spectra were recorded with a Bruker NEO-400 instrument from Bruker Biospin GmbH, Ettlingen, Germany. ^{1}H NMR spectra were obtained at 400 MHz in $CDCl_3$ with residual $C\mathit{H}Cl_3$ (δ = 7.26 ppm) as an internal reference. ^{13}C{^{1}H} NMR spectra were recorded at 100 MHz in $CDCl_3$ with $CDCl_3$ (δ = 77.1 ppm) as an internal reference. Whenever the solubility of the sample was insufficient in $CDCl_3$, it was replaced by either MeOH-d_4 (CD_2HOD as a calibrant for ^{1}H NMR spectroscopy, δ = 3.31; CD_3OD as a calibrant for ^{13}C{^{1}H} NMR spectroscopy, δ = 49.0) or DMSO-d_6 (DMSO-d_5 as a calibrant for ^{1}H NMR spectroscopy, δ = 2.50; DMSO-d_6 as a calibrant for ^{13}C{^{1}H} NMR spectroscopy, δ = 39.5). All signal assignments are based on 2D-NMR experiments COSY, HSQC, HMBC or NOESY. Whenever complex fine splitting of the individual lines of a signal due to long-range coupling was observed, the descriptor "m" was added to the multiplicity descriptor. The numbering scheme used for signal assignments is shown in scheme 1. IR spectra were recorded as ATR-FTIR spectra using a Perkin-Elmer UART TWO FT-IR spectrometer. Low- and high-resolution mass spectra were obtained by ESI-TOF using a Waters Micromass (Manchester, UK) instrument. For the chromatographic purification of compounds, the dry column vacuum chromatography (DCVC) method was used as described in the literature.[5]

4-Methoxybenzene-1,3-diol (5).[6] Isovanilline (**9**, 6.00 g, 39.4 mmol) was added over a period of 0.25 h to a well stirred mixture of H_2O_2 (aq., 30%, 8.90 mL, 86.7 mmol), and SeO_2 (350 mg, 3.10 mmol) in CH_2Cl_2 (100 mL) at 0 °C. Afterwards, the mixture was warmed to ambient temperature and stirred for 18 h, filtered, and washed with H_2O (100 mL). The organic phase

was separated, and the aqueous layer was further extracted with CH_2Cl_2 (3 times, 40 mL each). The combined organic layers were washed consecutively with 100 mL (each) of a 10% solution of $NaHSO_3$ and brine, and then evaporated to give a brown oil. The crude oil was dissolved in MeOH (30 mL), and aq. K_2CO_3 solution (25 mL, 15%) was added. The mixture was stirred at ambient temperature for 1 h. The solvent was evaporated, and H_2O (50 mL) was added. The mixture was extracted with EtOAc (3 times, 40 mL each). The aqueous phase was treated with aqueous HCl (1 M, 2 mL), and then extracted with EtOAc (twice, 40 mL each). The combined EtOAc layers were washed with brine (200 mL) and dried with anhydrous $MgSO_4$. The solution was filtered and evaporated *in vacuo* to yield compound **5** (4.60 g, 32.8 mmol, 83%), which was used in the next step without further purification: brown oil; IR (ATR) $\tilde{\nu}$ 3350 (s), 1606 (w), 1509 (s), 1218 (m), 1021 (w), 967 (w); ^{1}H NMR (400 MHz, $CDCl_3$) δ 6.71 (d, J = 8.6 Hz, 1H), 6.49 (d, J = 2.9 Hz, 1H), 6.31 (dd, J = 8.6, 2.9 Hz, 1H), 5.70 (s, 1H), 4.81 (s, 1H), 3.83 (s, 3H); ^{13}C{^{1}H} NMR (100 MHz, $CDCl_3$) δ 150.4, 146.6, 141.1, 111.9, 106.0, 103.1, 56.7; HREIMS m/z 140.0469 [M$^+$] (calcd for $C_7H_8O_3$, 140.0468).

1-(2,4-Dihydroxy-5-methoxyphenyl)ethan-1-one (6).[7] $BF_3{\bullet}OEt_2$ (1.20 mL, 10.0 mmol) was added to a mixture of **5** (1.40 g, 10.0 mmol), and acetyl chloride (1.40 mL, 20.0 mmol) at 0 °C. The mixture was then heated to reflux at 80 °C for 3 h. After cooling to ambient temperature, the yellow residue was poured onto icewater (50 mL). A saturated aq. solution of $NaHCO_3$ (50 mL) was added, and the mixture was extracted with EtOAc (3 times, 50 mL each). The combined EtOAc layers were washed with brine (150 mL) and dried over anhydrous $MgSO_4$. The solution was filtered and evaporated *in vacuo*. The residue was purified by column chromatography on silica, using petroleum ether/EtOAc mixture (4 : 1 (v/v)) as eluent, to yield compound **6** (1.50 g, 8.2 mmol, 82%): colourless powder; IR (ATR) $\tilde{\nu}$ 3189 (s), 1508 (s), 1279 (s), 1222 (m), 1160 (s), 843 (w); ^{1}H NMR (400 MHz, $CDCl_3$) δ 12.58 (s, 1H), 7.04 (s, 1H), 6.51 (s, 1H), 6.33 (s, 1H), 3.90 (s, 3H), 2.55 (s, 3H); ^{13}C{^{1}H} NMR (100 MHz, $CDCl_3$) δ 202.1,

160.4, 153.9, 139.8, 112.2, 110.8, 103.6, 56.6, 26.5; HREIMS *m/z* 182.0589 [M^+] (calcd for $C_9H_{10}O_4$, 182.0579).

1-(4-(Allyloxy)-2-hydroxy-5-methoxyphenyl)ethan-1-one (7). To a solution of **6** (1.65 g, 9.1 mmol) in acetone (65 mL) was added K_2CO_3 (1.30 g, 9.1 mmol) and the mixture was stirred at ambient temperature for 20 min. A solution of allyl bromide (786 µL, 9.1 mmol) in acetone (1.5 mL) was added dropwise, and the mixture was stirred at ambient temperature for 0.5 h. It was then heated to reflux at 56 °C for 7 h, cooled to ambient temperature, filtered, and evaporated in vacuo. The residue was purified by column chromatography on silica, using petroleum ether/EtOAc mixture (6 : 1 (v/v)) as eluent, to yield compound **7** (1.80 g, 8.1 mmol, 89%): yellow oil; IR (ATR) $\tilde{\nu}$ 2932 (w), 1618 (s), 1503 (s), 1329 (m), 1256 (s), 1167 (s), 1060 (w), 954 (w); ^{1}H NMR (400 MHz, acetone-d_6) δ 12.64 (s, 1H), 7.32 (s, 1H), 6.48 (s, 1H), 6.10 (ddt, J = 17.3, 10.5, 5.2 Hz, 1H), 5.47 (dq, J = 17.3, 1.8 Hz, 1H), 5.30 (dq, J = 10.5, 1.8 Hz, 1H), 4.67 (dt, J = 5.2, 1.8 Hz, 2H), 3.82 (s, 3H), 2.58 (s, 3H); ^{13}C{^{1}H} NMR (100 MHz, acetone-d_6) δ 203.7, 160.7, 157.0, 143.2, 133.8, 118.2, 114.1, 112.6, 102.2, 70.0, 57.0, 26.6; HREIMS *m/z* 222.0889 [M^+] (calcd for $C_{12}H_{14}O_4$, 222.0892).

1-(3-Allyl-2,4-dihydroxy-5-methoxyphenyl)ethan-1-one (8). Compound **7** (1.80 g, 8.1 mmol) was dissolved in toluene (8 mL) and the solution was placed in a vessel suited for microwave irradiation. The vessel was sealed, and the solution was irradiated at 250 °C for 1.5 h. After cooling to ambient temperature, the solvent was evaporated, and the residue was purified by column chromatography on silica, using petroleum ether/EtOAc mixture (8 : 1 (v/v)) as eluent, to yield compound **8** (1.60 g, 7.2 mmol, 89%): white solid; IR (ATR) $\tilde{\nu}$ 3338 (s), 1620 (w), 1490 (s), 1331 (m), 1286 (m), 1225 (s), 1095 (w), 807 (w); ^{1}H NMR (400 MHz, acetone-d_6) δ 13.04 (s, 1H), 7.28 (s, 1H), 5.93 (ddt, J = 17.2, 10.0, 6.4 Hz, 1H), 5.01 (dq, J = 17.2, 1.8 Hz, 1H), 4.90 (dq, J = 10.0, 1.8 Hz, 1H), 3.87 (s, 3H), 3.40 (dt, J = 6.4, 1.8 Hz, 2H), 2.57 (s, 3H); ^{13}C{^{1}H} NMR (100 MHz, acetone-d_6) δ 203.8, 159.1, 153.4, 141.2, 136.7, 115.1, 114.6,

112.0, 110.9, 57.0, 27.6, 26.6; HRESIMS *m/z* 223.0972 $[M+H]^+$ (calcd for $C_{12}H_{15}O_4$, 223.0970).

1-(3-Allyl-2-hydroxy-5-methoxy-4-(methoxymethoxy)phenyl)ethan-1-one (9). NEt(*i*-Pr)$_2$ (2.60 mL, 15.0 mmol) was added dropwise at 0 °C to a solution of compound **8** (2.23 g, 10.0 mmol) in dry CH_2Cl_2 (100 mL). The mixture was stirred at 0 °C for 20 min. MOM-bromide (1.00 mL, 12.5 mmol) was added dropwise at the same temperature, and the mixture was stirred at 0 °C for 20 min. It was allowed to warm to ambient temperature, and stirring was continued for 16 h. A satd. aq. solution of NH_4Cl (10 mL) and H_2O (100 mL) were added, and the CH_2Cl_2 phase was separated. The aqueous layer was extracted with EtOAc (twice, 50 mL each). The combined organic layers were dried with anhydrous $MgSO_4$, filtered, and evaporated *in vacuo*. The crude residue was purified by column chromatography on silica, using petroleum ether/EtOAc mixture (6 : 1 (v/v)) as eluent, to yield compound **9** (2.56 g, 9.6 mmol, 96%): yellow oil; IR (ATR) $\tilde{\nu}$ 2935 (w), 1628 (s), 1464 (m), 1368 (m), 1329 (m), 1274 (s), 1159 (s), 1034 (m), 922 (m); ^{1}H NMR (400 MHz, $CDCl_3$) δ 12.59 (s, 1H), 7.07 (s, 1H), 5.99 (ddt, *J* = 17.2, 10.0, 6.1 Hz, 1H), 5.22 (s, 2H), 5.04 (dq, *J* = 17.2, 1.7 Hz, 1H), 5.00 (dq, *J* = 10.2, 1.7 Hz, 1H), 3.83 (s, 3H), 3.57 (s, 3H), 3.51 (dt, *J* = 6.1, 1.6 Hz, 2H), 2.58 (s, 3H); $^{13}C\{^1H\}$ NMR (100 MHz, $CDCl_3$) δ 203.0, 157.4, 152.0, 144.7, 135.9, 122.9, 115.2, 114.7, 111.2, 99.1, 57.8, 56.7, 27.9, 26.8; HREIMS *m/z* 266.1158 $[M^+]$ (calcd for $C_{14}H_{18}O_5$, 266.1154).

(*E*)-1-(3-Allyl-2-hydroxy-5-methoxy-4-(methoxymethoxy)phenyl)-3-(dimethylamino)-prop-2-en-1-one (10). Compound **9** (2.60 g, 9.8 mmol) was dissolved in dry and degassed DMF (7.0 mL), and dimethylformamide-dimethylacetal (2.00 mL, 14.7 mmol) was added. The solution was transferred into a vessel suited for microwave irradiation, sealed, and irradiated at 115 °C for 3 min. The solution was cooled to ambient temperature, H_2O (100 mL) was added, and the mixture was extracted with EtOAc (3 × 75 mL). The combined organic layers were dried with anhydrous $MgSO_4$, filtered, and evaporated *in vacuo*. The residue was purified by column chromatography on silica, using petroleum ether/EtOAc mixture (2 : 1 (v/v)) to yield

compound **10** (3.20 g, 10.0 mmol, quant.): yellow oil; IR (ATR) $\tilde{\nu}$ 2929 (w), 1626 (s), 1541 (m), 1367 (w), 1265 (m), 1155 (w), 958 (w); ^{1}H NMR (400 MHz, $CDCl_3$) δ 14.14 (s, 1H), 7.86 (d, *J* = 12.1 Hz, 1H), 7.08 (s, 1H), 6.04 (ddt, *J* = 17.2, 10.0, 6.0 Hz, 1H), 5.62 (d, *J* = 12.1 Hz, 1H), 5.17 (s, 2H), 5.04 (dq, *J* = 17.1, 1.8 Hz, 1H), 4.99 (dq, *J* = 10.1, 1.8 Hz, 1H), 3.82 (s, 3H), 3.57 (s, 3H), 3.51 (dt, *J* = 6.0, 1.7 Hz, 2H), 3.17 (s(br.), 3H), 2.97 (s(br.), 3H); ^{13}C{^{1}H} NMR (100 MHz, $CDCl_3$) δ 190.9, 157.6, 154.6, 150.1, 144.2, 136.5, 122.6, 115.2, 114.8, 110.4, 99.1, 90.1, 57.7, 57.1, 45.5, 37.5, 28.1; HREIMS *m*/*z* 321.1581 [M^+] (calcd for $C_{17}H_{23}NO_5$, 321.1576).

8-Allyl-3-iodo-6-methoxy-7-(methoxymethoxy)-4*H*-chromen-4-one (11). Compound **10** (3.20 g, 10.0 mmol) was dissolved in $CHCl_3$ (35 mL). Pyridine (1.20 mL, 15.0 mmol) and I_2 (5.10 g, 20.0 mmol) were added. The solution was stirred at ambient temperature for 2 h. The reaction was quenched by addition of a satd. aq. solution of $Na_2S_2O_3$ (40 mL). The mixture was stirred for an additional 5 min, and H_2O (150 mL) was added. The aqueous layer was extracted with CH_2Cl_2 (3 times, 100 mL each). The combined organic layers were dried with anhydrous $MgSO_4$, filtered, and evaporated *in vacuo*. The residue was purified by column chromatography on silica, using petroleum ether/EtOAc mixture (7 : 1 (v/v)) as eluent, to yield compound **11** (2.70 g, 6.7 mmol, 67%): brown oil; IR (ATR) $\tilde{\nu}$ 2945 (w), 1638 (s), 1607 (m), 1461 (s), 1425 (s), 1315 (m), 1195 (m), 1022 (m), 887 (w); ^{1}H NMR (400 MHz, $CDCl_3$) δ 8.29 (s, 1H), 7.53 (s, 1H), 5.95 (ddt, *J* = 16.6, 10.1, 6.0 Hz, 1H), 5.22 (s, 2H), 5.03 (dq, *J* = 10.1, 1.8 Hz, 1H), 5.00 (dq, *J* = 16.9, 1.8 Hz, 1H), 3.93 (s, 3H), 3.66 (dt, *J* = 6.0, 1.7 Hz, 2H), 3.59 (s, 3H); ^{13}C{^{1}H} NMR (100 MHz, $CDCl_3$) δ 173.1, 157.6, 151.0, 150.2, 149.8, 135.0, 123.2, 118.3, 116.1, 104.6, 99.3, 86.2, 57.9, 56.3, 28.3; HREIMS *m*/*z* 401.9969 [M^+] (calcd for $C_{15}H_{15}{}^{127}IO_5$, 401.9964).

3-Iodo-6-methoxy-7-(methoxymethoxy)-8-(3-methylbut-2-en-1-yl)-4*H*-chromen-4-one (12). Compound **11** (2.70 g, 6.7 mmol) was dissolved in CH_2Cl_2 (38 mL). 2-Methyl-2-butene (6.37 mL, 60.3 mmol) and Ru-catalyst **A** (284 mg, 0.34 mmol, 5.0 mol%) were added, and the mixture was stirred at ambient temperature under an atmosphere of dry nitrogen for 48 h. The

solvent was evaporated *in vacuo* and the residue purified by column chromatography on silica, using petroleum ether/EtOAc mixture (5 : 1 (v/v)) as eluent, to yield compound **12** (2.80 g, 6.5 mmol, 97%): brown oil; IR (ATR) $\tilde{\nu}$ 2924 (w), 1639 (s), 1459 (s), 1424 (s), 1314 (s), 1072 (m), 927 (m); ^{1}H NMR (400 MHz, $CDCl_3$) δ 8.30 (s, 1H), 7.50 (s, 1H), 5.22 (s, 2H), 5.17 (tm, *J* = 7.0 Hz, 1H), 3.93 (s, 3H), 3.61 (d, *J* = 7.0, 2H), 3.59 (s, 3H), 1.80 (s, 3H), 1.67 (s, 3H); $^{13}C\{^1H\}$ NMR (100 MHz, $CDCl_3$) δ 173.1, 157.5, 151.0, 150.3, 149.5, 133.1, 125.2, 121.2, 118.4, 104.1, 99.3, 86.2, 57.9, 56.3, 25.8, 23.5, 18.1; HREIMS *m*/*z* 430.0283 [M$^+$] (calcd for $C_{17}H_{19}{}^{127}IO_5$, 430.0277).

3,4-Dimethoxyphenol (**14**).[8] Following the procedure for the synthesis of compound **5**, veratraldehyde (**13**, 6.60 g, 39.7 mmol) was converted to **14** (3.50 g, 22.7 mmol, 57%). Unreacted starting material **13** (1.50 g, 9.0 mmol, 23%) was recovered by chromatography on silica, using petroleum ether/EtOAc (8 : 1 (v/v)) as eluent: colorless oil; ^{1}H NMR (400 MHz, $CDCl_3$) δ 6.72 (d, *J* = 8.6 Hz, 1H), 6.46 (d, *J* = 2.8 Hz, 1H), 6.35 (dd, *J* = 8.6, 2.8 Hz, 1H), 3.81 (s, 3H), 3.79 (s, 3H); $^{13}C\{^1H\}$ NMR (100 MHz, $CDCl_3$) δ 150.3, 150.0, 143.2, 112.6, 106.0, 100.8, 56.7, 55.9; IR (ATR) $\tilde{\nu}$ 3422 (s), 1607 (m), 1507 (s), 1476 (m), 1289 (m), 1221 (s), 1126 (s), 1022 (m), 948 (m); HRESIMS *m*/*z* 155.0701 [M+H]$^+$ (calcd for $C_8H_{11}O_3$, 155.0708).

1,2,4-Trimethoxybenzene (**15**).[9] To a solution of **14** (1.10 g, 7.1 mmol) in acetone (47 mL) was added K_2CO_3 (2.9 g, 21.3 mmol) and the mixture was stirred at ambient temperature for 20 min. A solution of iodomethane (884 µL, 14.2 mmol) in acetone (1 mL) was added dropwise, and the mixture was stirred at ambient temperature for 0.5 h. After refluxing at 56 °C for 7 h, the solution was cooled to ambient temperature, filtered, and evaporated *in vacuo*. The residue was purified by column chromatography on silica, using petroleum ether/EtOAc mixture (9 : 1 (v/v)) as eluent, to yield **15** (1.10 g, 6.5 mmol, 92%): colorless oil; IR (ATR) $\tilde{\nu}$ 2937 (w), 1596 (w), 1508 (s), 1205 (s), 1155 (m), 1137 (m), 1023 (m); ^{1}H NMR (400 MHz, $CDCl_3$) δ 6.78 (d, *J* = 8.7 Hz, 1H), 6.51 (d, *J* = 2.8 Hz, 1H), 6.39 (dd, *J* = 8.7, 2.8 Hz, 1H), 3.85 (s, 3H), 3.83 (s,

3H), 3.77 (s, 3H); $^{13}C\{^1H\}$ NMR (100 MHz, $CDCl_3$) δ 154.4, 150.0, 143.6, 112.0, 103.0, 100.5, 56.6, 55.9, 55.8; HRESIMS *m/z* 169.0866 $[M+H]^+$ (calcd for $C_9H_{13}O_3$, 169.0865).

1-Bromo-2,4,5-trimethoxybenzene (16).[10] To a solution of **15** (1.00 g, 6.0 mmol) in THF (85 mL), was added bromine (307 μL, 6.0 mmol) at 0 °C. The mixture was stirred at 0 °C for 10 min. A satd. aq. solution of Na_2CO_3 (100 mL) and Et_2O (50 mL) were added, and the organic phase was separated. The aqueous layer was further extracted with Et_2O (twice, 50 mL each). The combined organic layers were washed with brine, dried with anhydrous $MgSO_4$, filtered, and evaporated *in vacuo*. The crude residue was purified by column chromatography on silica, using petroleum ether/EtOAc mixture (6 : 1 (v/v)) as eluent, to yield **16** (1.50 g, 6.1 mmol, quant.): reddish oil; IR (ATR) $\tilde{\nu}$ 2852 (w), 1505 (s), 1437 (m), 1377 (m), 1024 (s), 1165 (m), 1022 (s), 838 (m); ^1H NMR (400 MHz, $CDCl_3$) δ 7.03 (s, 1H), 6.56 (s, 1H), 3.88 (s, 3H), 3.86 (s, 3H), 3.82 (s, 3H); $^{13}C\{^1H\}$ NMR (100 MHz, $CDCl_3$) δ 150.4, 149.2, 143.9, 116.6, 101.2, 99.0, 57.4, 56.8, 56.4. No $[M^+]$ peak could be observed with either EI or ESI ionization mode.

(2,4,5-Trimethoxyphenyl)boronic acid (3e).[11] To a solution of **16** (543 mg, 2.20 mmol) in THF (12 mL), was added B(O*i*-Pr)$_3$ (1.00 mL, 4.40 mmol) dropwise under N_2 atmosphere. The solution was cooled to –78 °C. BuLi (2.5 M in hexanes, 1.30 mL, 3.30 mmol) was added over 12 min. Afterwards, the solution was stirred at –78 °C for 1 h, warmed to ambient temperature, and stirred for an additional 3 h. The reaction was quenched with aq. NH_4Cl (30 mL), and the mixture was extracted with EtOAc (3 times, 25 mL each). The combined organic layers were washed with brine and dried with anhydrous $MgSO_4$, filtered, and evaporated *in vacuo* to yield **3e** (488 mg, 2.30 mmol, quant.): off-white powder; IR (ATR) $\tilde{\nu}$ 3365 (s), 2932 (m), 1510 (m), 1466 (m), 1378 (s), 1270 (m), 1207 (s), 1048 (m), 758 (m); ^1H NMR (400 MHz, $CDCl_3$) δ 7.32 (s, 1H), 6.51 (s, 1H), 3.92 (s, 3H), 3.90 (s, 3H), 3.88 (s, 3H); $^{13}C\{^1H\}$ NMR (100 MHz, $CDCl_3$) δ 160.1, 152.6, 143.5, 118.4, 96.1, 56.4, 56.3, 56.0, C-B signal not observed. No $[M^+]$ peak could be observed with either EI or ESI ionization mode.

5-Methoxybenzo[*d*][1,3]dioxole (**18**).[12] Following the procedure for the synthesis of compound **15**, sesamol (1.000 g, 7.20 mmol) was converted to **18** (958 mg, 6.30 mmol, 88%). Eluent for chromatography: petroleum ether/EtOAc (9 : 1 (v/v)): colorless oil; IR (ATR) $\tilde{\nu}$ 2900 (w), 1485 (s), 1194 (s), 1174 (s), 1131 (m), 1024 (m), 937 (w); ^{1}H NMR (400 MHz, $CDCl_3$) δ 6.71 (d, J = 8.5 Hz, 1H), 6.49 (d, J = 2.5 Hz, 1H), 6.32 (dd, J = 8.5, 2.5 Hz, 1H), 5.91 (s, 2H), 3.75 (s, 3H); ^{13}C{^{1}H} NMR (100 MHz, $CDCl_3$) δ 155.3, 148.4, 141.7, 108.0, 104.8, 101.3, 97.6, 56.1; HRESIMS m/z 153.0551 $[M+H]^+$ (calcd for $C_8H_9O_3$, 153.0552).

5-Bromo-6-methoxybenzo[*d*][1,3]dioxole (**19**).[12] Following the procedure for the synthesis of compound **16**, compound **18** (913 mg, 6.00 mmol) was converted to **19** (1.30 g, 5.60 mmol, 93%). Eluent for chromatography: petroleum ether/EtOAc (9 : 1 (v/v)): yellowish solid; IR (ATR) $\tilde{\nu}$ 2902 (w), 1486 (s), 1388 (m), 1268 (s), 1174 (s), 1112 (m), 1031 (s), 925 (m); ^{1}H NMR (400 MHz, $CDCl_3$) δ 6.99 (s, 1H), 6.56 (s, 1H), 5.94 (s, 2H), 3.82 (s, 3H); ^{13}C{^{1}H} NMR (100 MHz, $CDCl_3$) δ 151.3, 147.9, 142.0, 112.8, 101.9, 101.6, 96.1, 57.3. No $[M^+]$ peak could be observed with either EI or ESI ionization mode.

(6-Methoxybenzo[*d*][1,3]dioxol-5-yl)boronic acid (**3f**). Following the procedure for the synthesis of **3d**, compound **19** (500 mg, 2.20 mmol) was converted to **3f** (324 mg, 1.70 mmol, 77%): white powder; IR (ATR) $\tilde{\nu}$ 3332 (s), 1617 (m), 1426 (s), 1403 (s), 1326 (m), 1259 (m), 1199 (m), 1028 (s), 926 (m); ^{1}H NMR (400 MHz, DMSO-d_6) δ 7.53 (s, 2H), 7.04 (s, 1H), 6.77 (s, 1H), 5.97 (s, 2H), 3.78 (s, 3H); ^{13}C{^{1}H} NMR (100 MHz, DMSO-d_6) δ 160.3, 150.2, 140.8, 113.4, 101.1, 94.6, 56.3, C-B signal not observed. No $[M^+]$ peak could be observed with either EI or ESI ionization mode.

E Copies of NMR-spectra and NMR-data for comparison

**No**	page	**No**	page	**No**	page
**5**	S18	**3e**	S40	**21b**	S65
**6**	S20	**18**	S42	**21c**	S67
**7**	S22	**19**	S44	**21d**	S69
**8**	S24	**3f**	S46	**21e**	S74
**9**	S26	**20a**	S49	**21f**	S76
**10**	S28	**20b**	S51	**22b**	S78
**11**	S30	**20c**	S53	**22c**	S80
**12**	S32	**20d**	S57	**22d**	S82
**14**	S34	**20e**	S59	**22e**	S84
**15**	S36	**20f**	S61	**22f**	S86
**16**	S38	**21a**	S63	**23**	S88
**Table S4** and Structures of natural products			S90		
**Table S5**			S91		
**Table S6**			S92		
**Table S7**			S93		
**Table S8**			S94		
**Table S9**			S95		

Figure S7: ^{1}H NMR (400 MHz, $CDCl_3$) of **5**

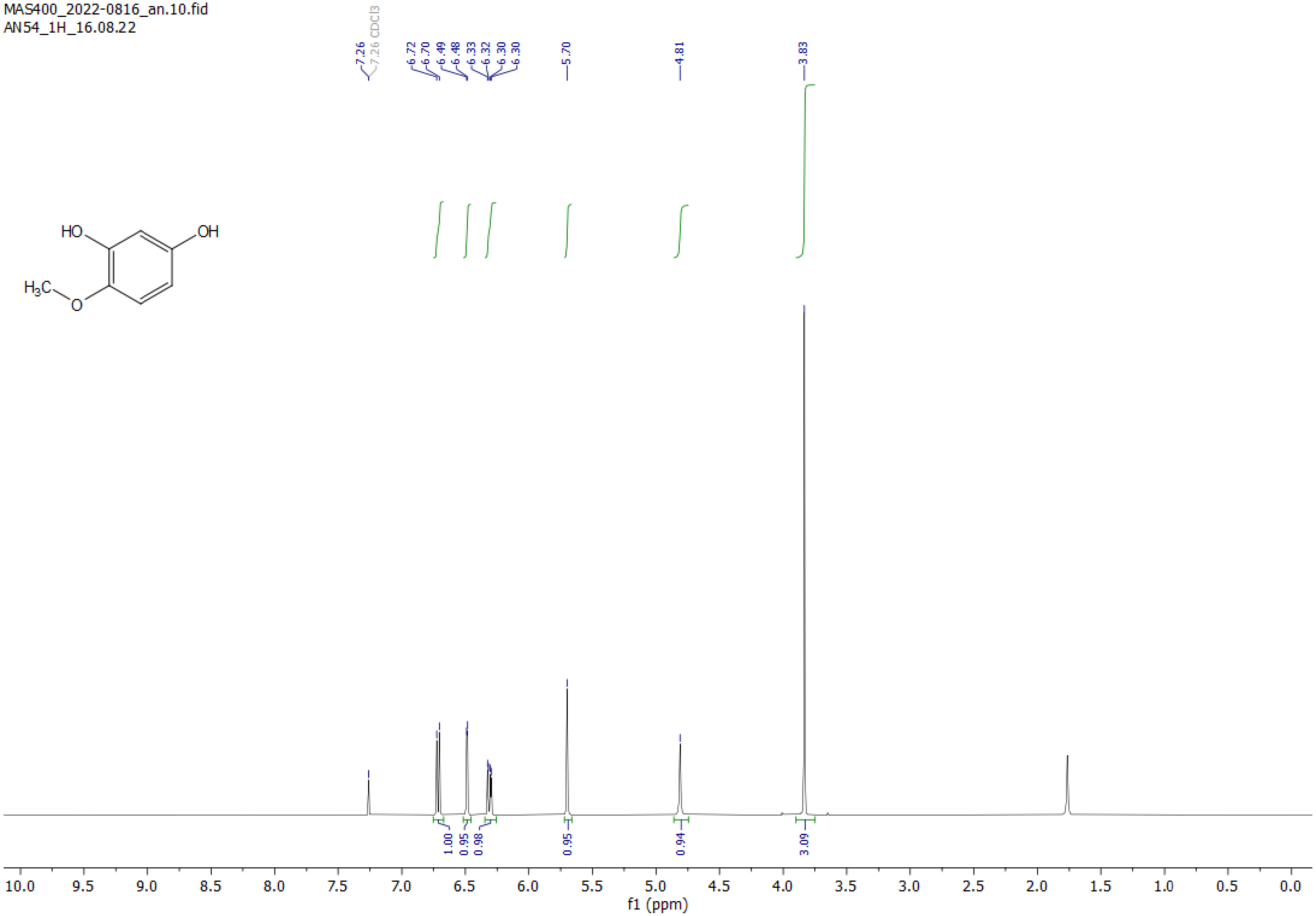

Figure S8: $^{13}C\{^1H\}$ NMR (101 MHz, $CDCl_3$) of **5**

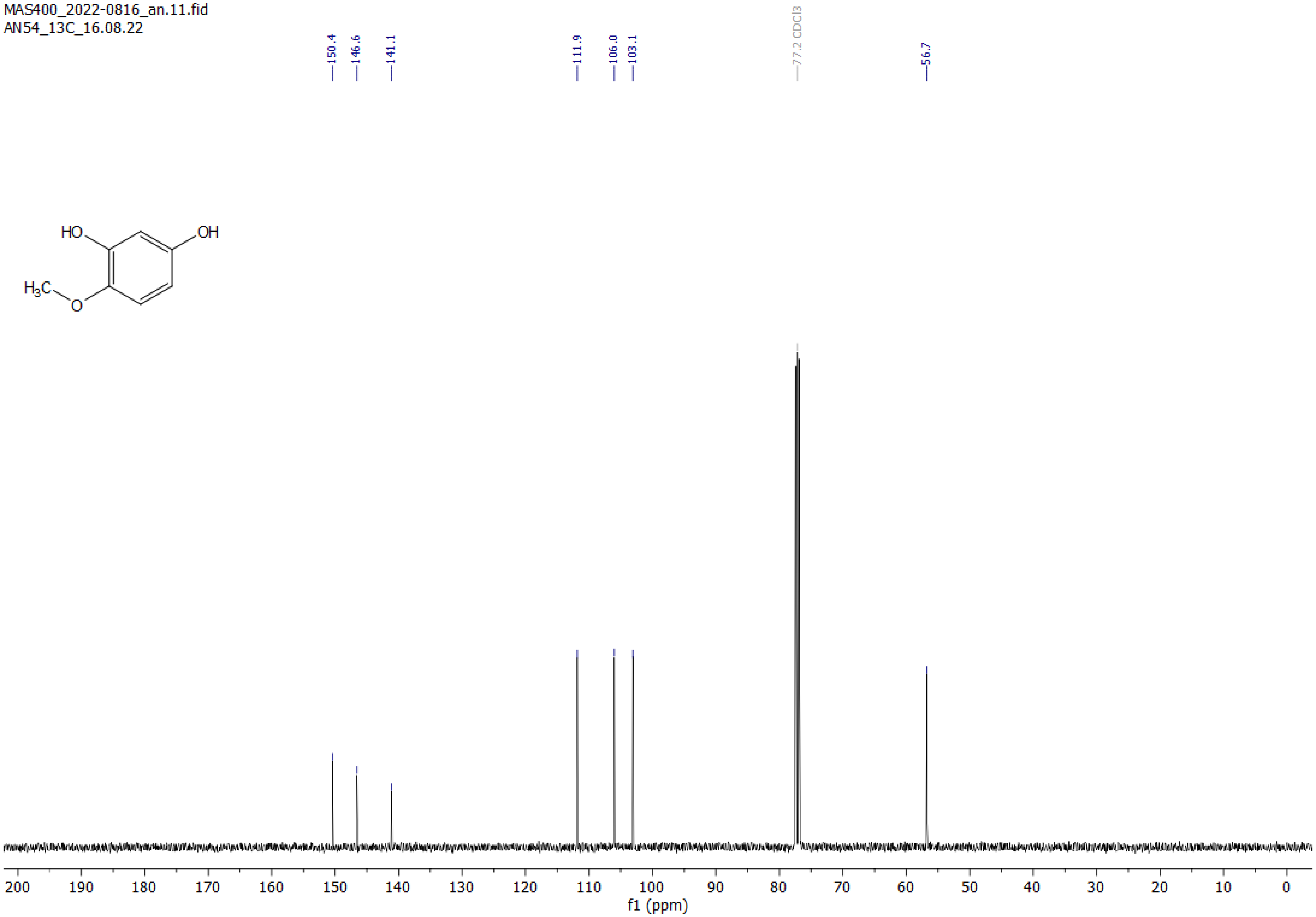

Figure S9: ^{1}H NMR (400 MHz, $CDCl_3$) of **6**

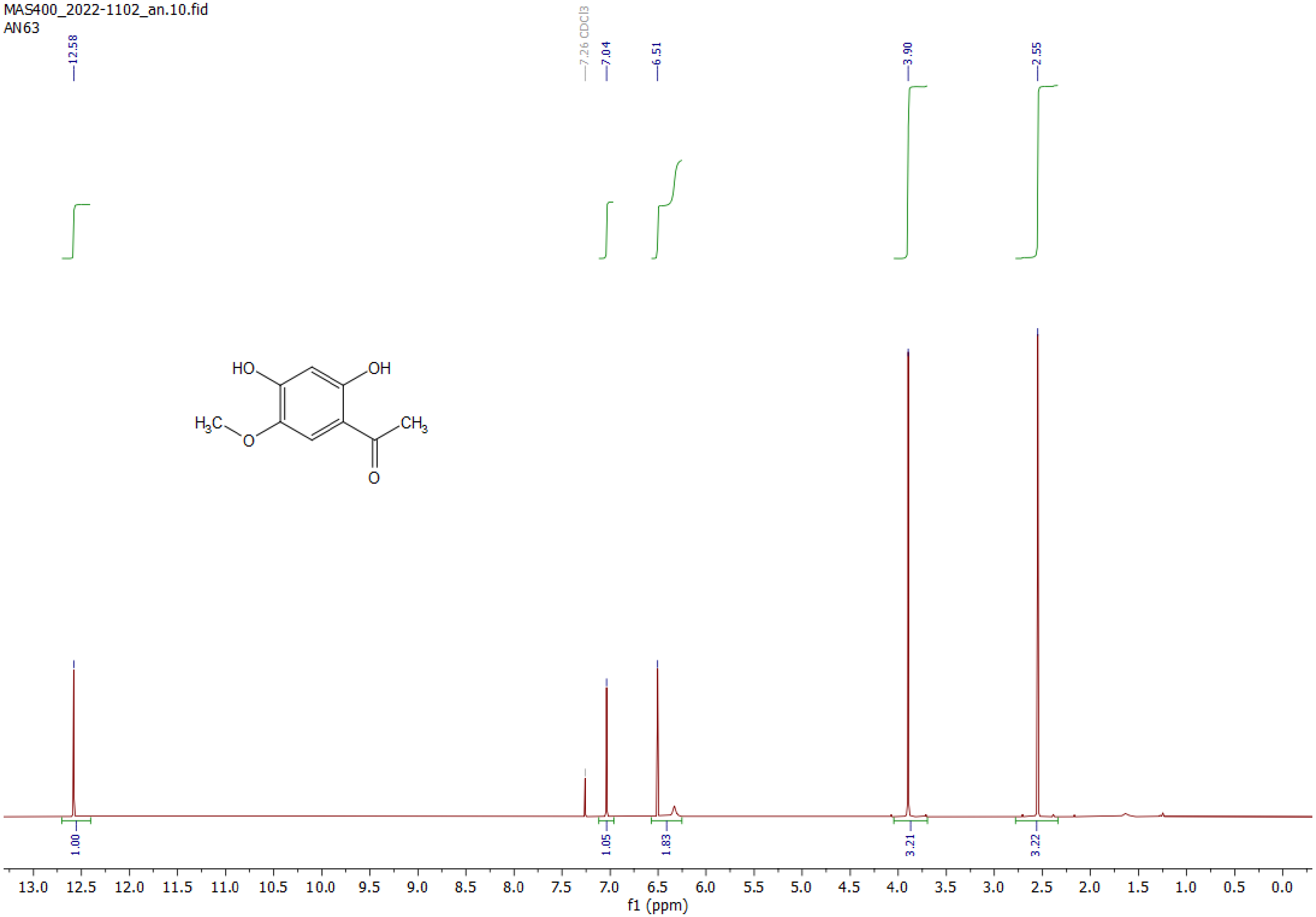

Figure S10: $^{13}C\{^{1}H\}$ NMR (101 MHz, $CDCl_3$) of **6**

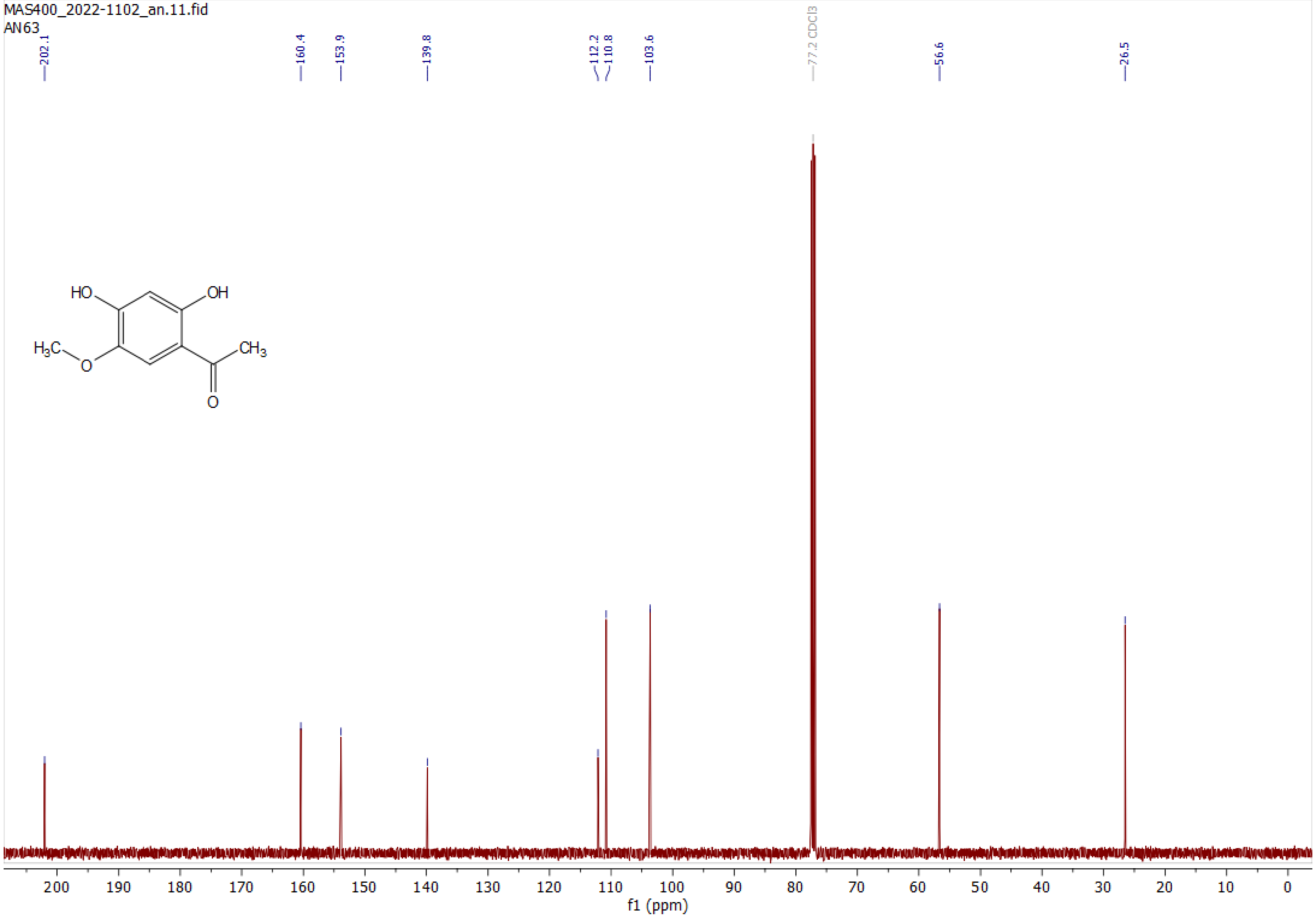

Figure S11 ^{1}H NMR (400 MHz, acetone-d_6) of **7**

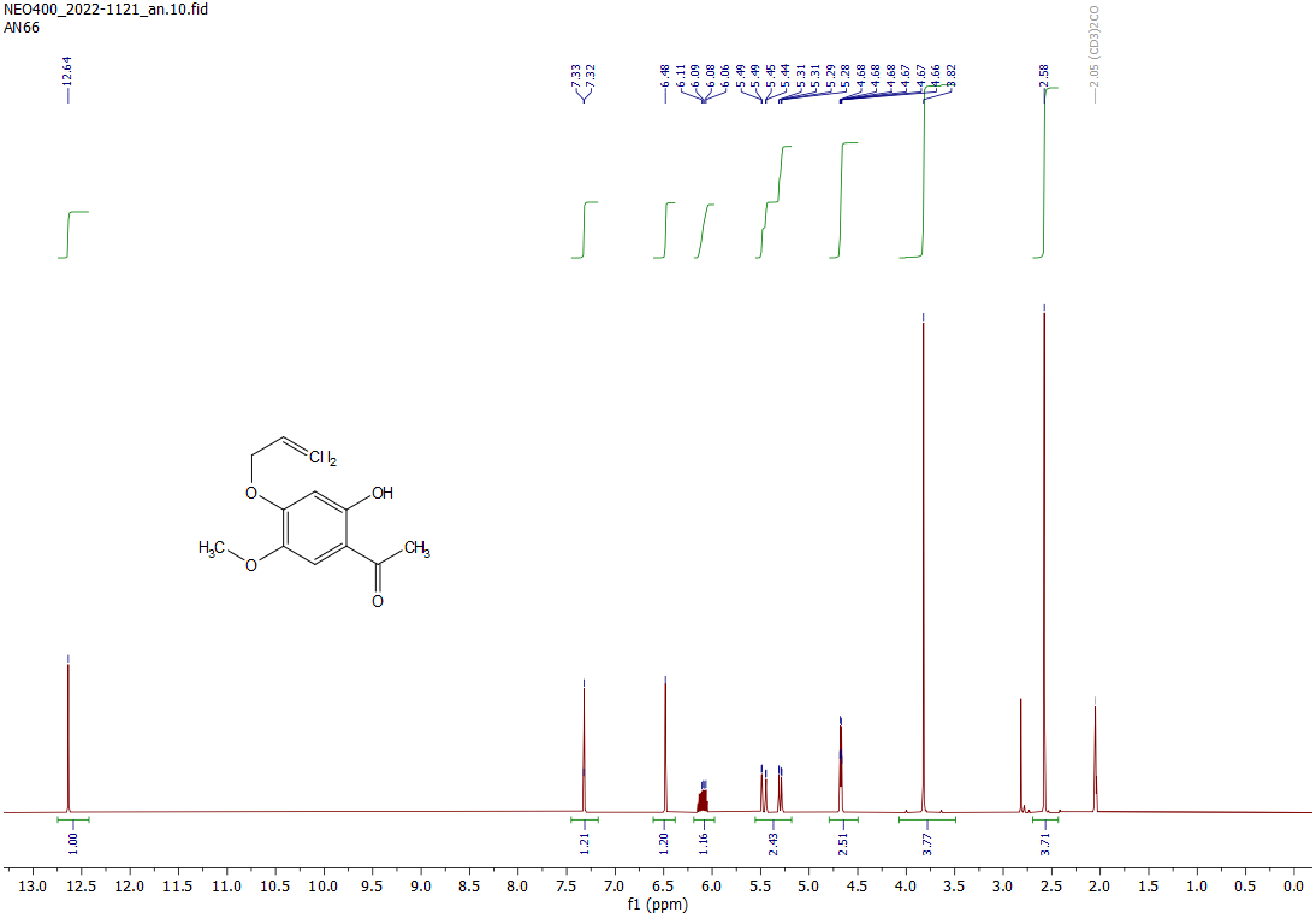

Figure S12: $^{13}C\{^1H\}$ NMR (101 MHz, acetone-d_6) of **7**

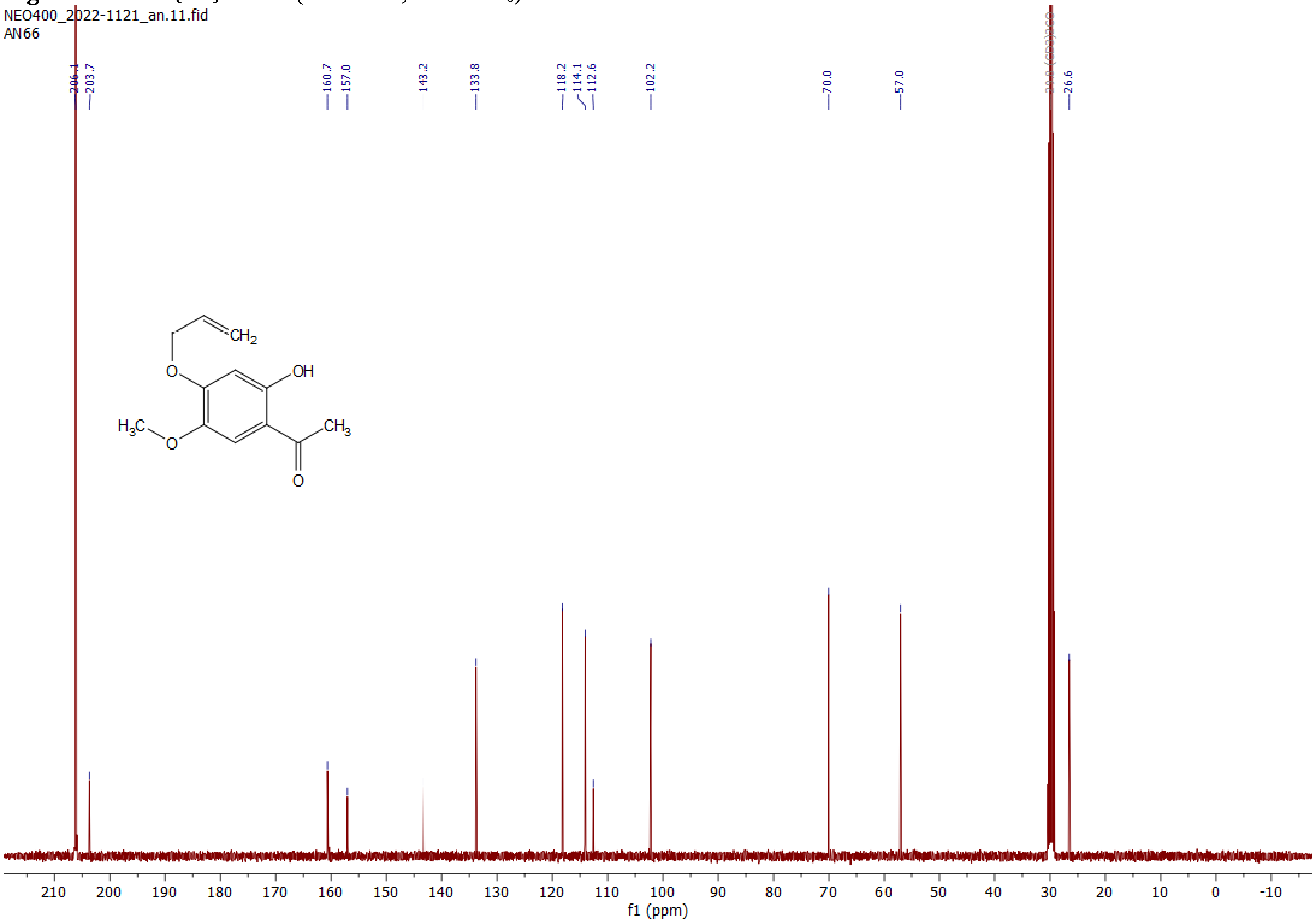

Figure S13: ^{1}H NMR (400 MHz, acetone-d_6) of **8**

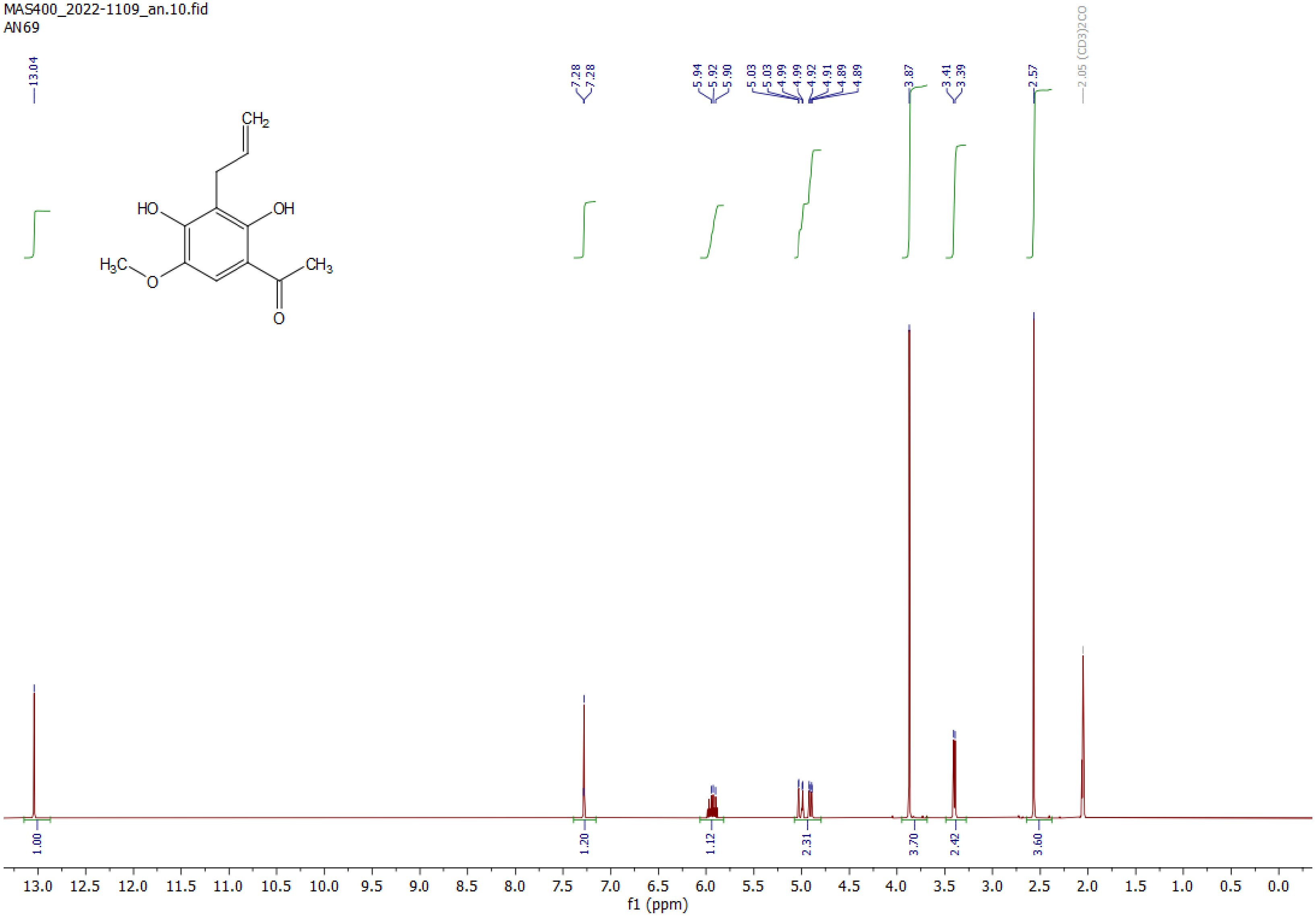

Figure S14: $^{13}C\{^{1}H\}$ NMR (101 MHz, acetone-d_6) of **8**

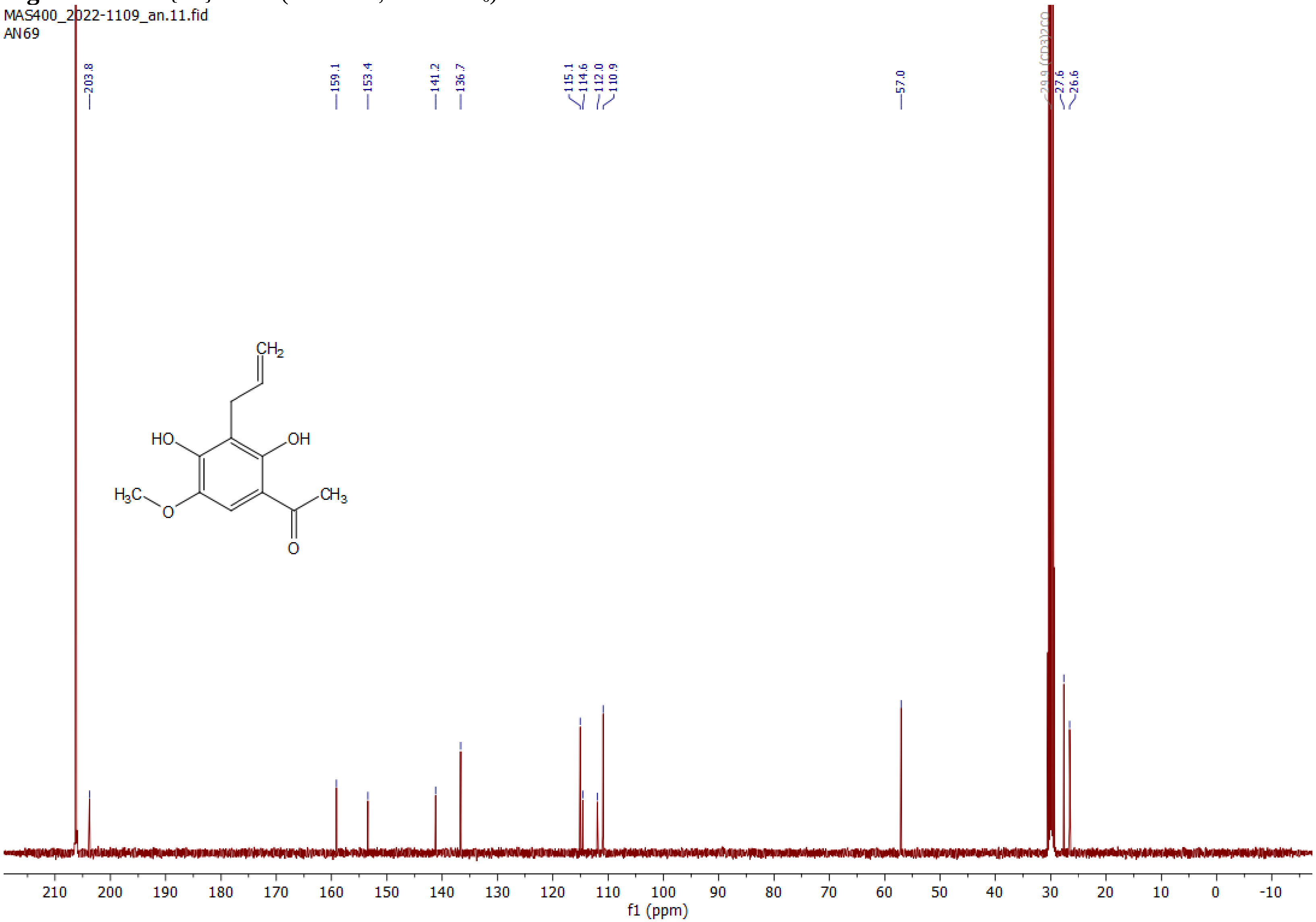

Figure S15: ^{1}H NMR (400 MHz, $CDCl_3$) of **9**

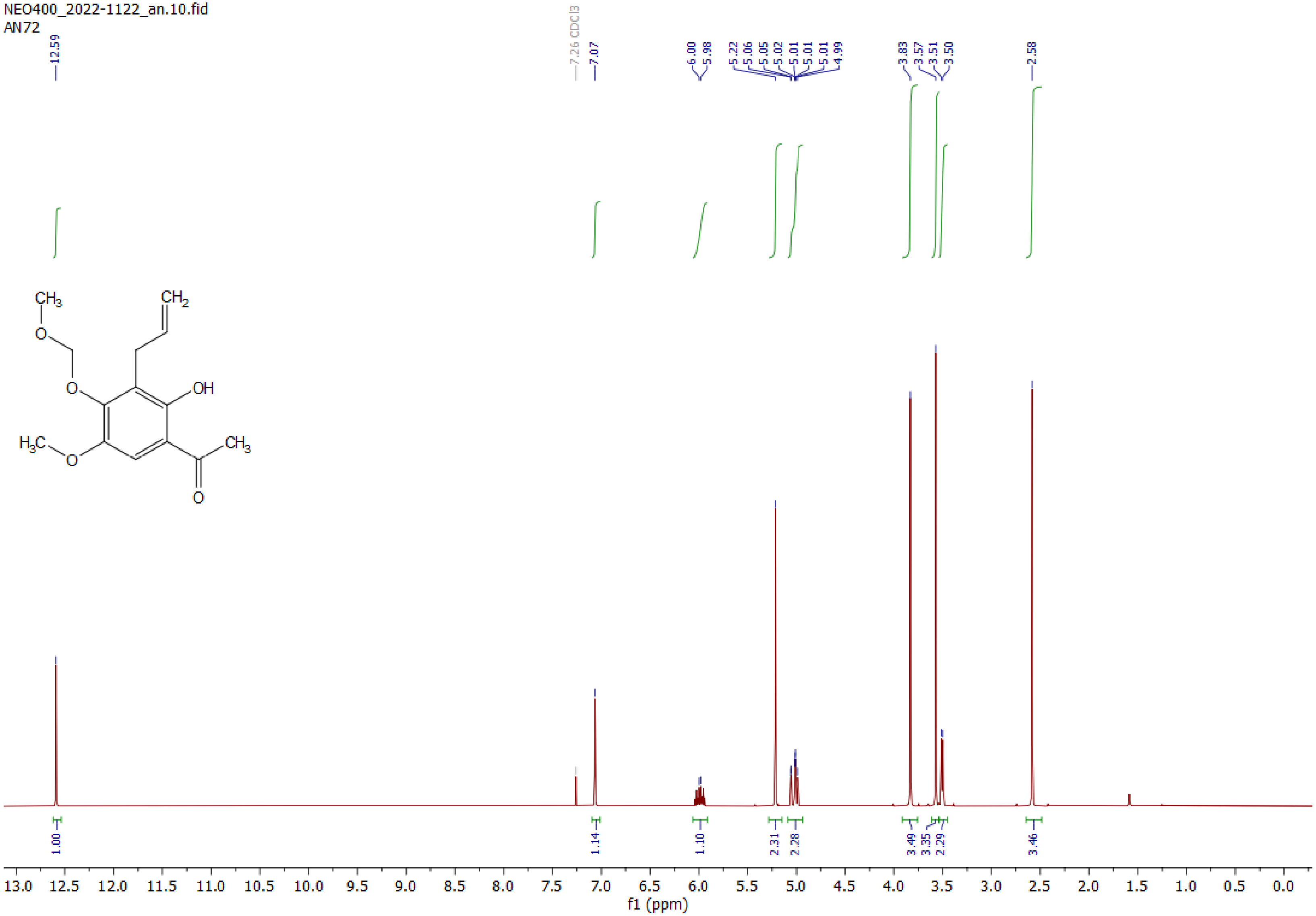

Figure S16: $^{13}C\{^{1}H\}$ NMR (101 MHz, $CDCl_3$) of **9**

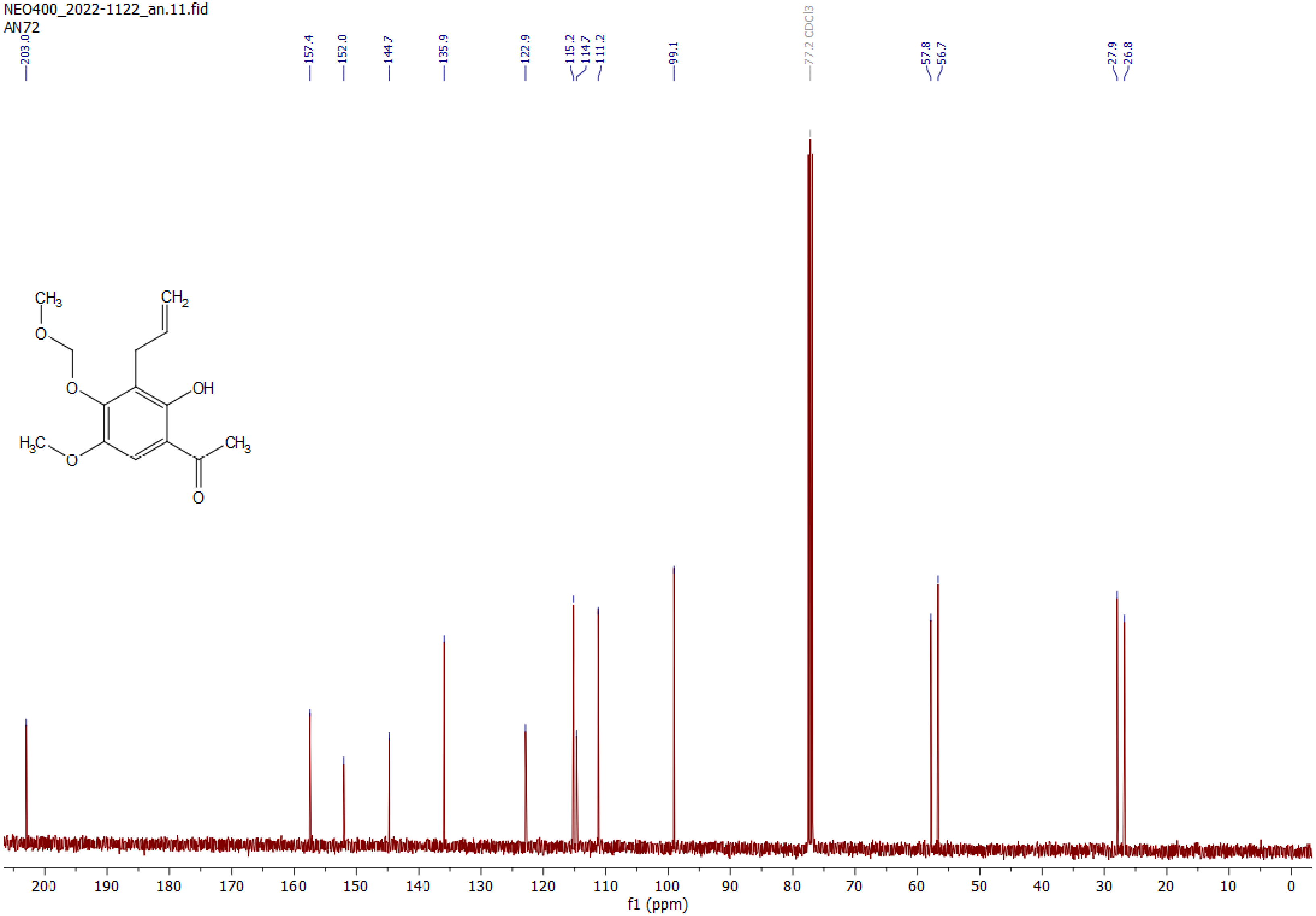

Figure S17: ^{1}H NMR (400 MHz, $CDCl_3$) of **10**

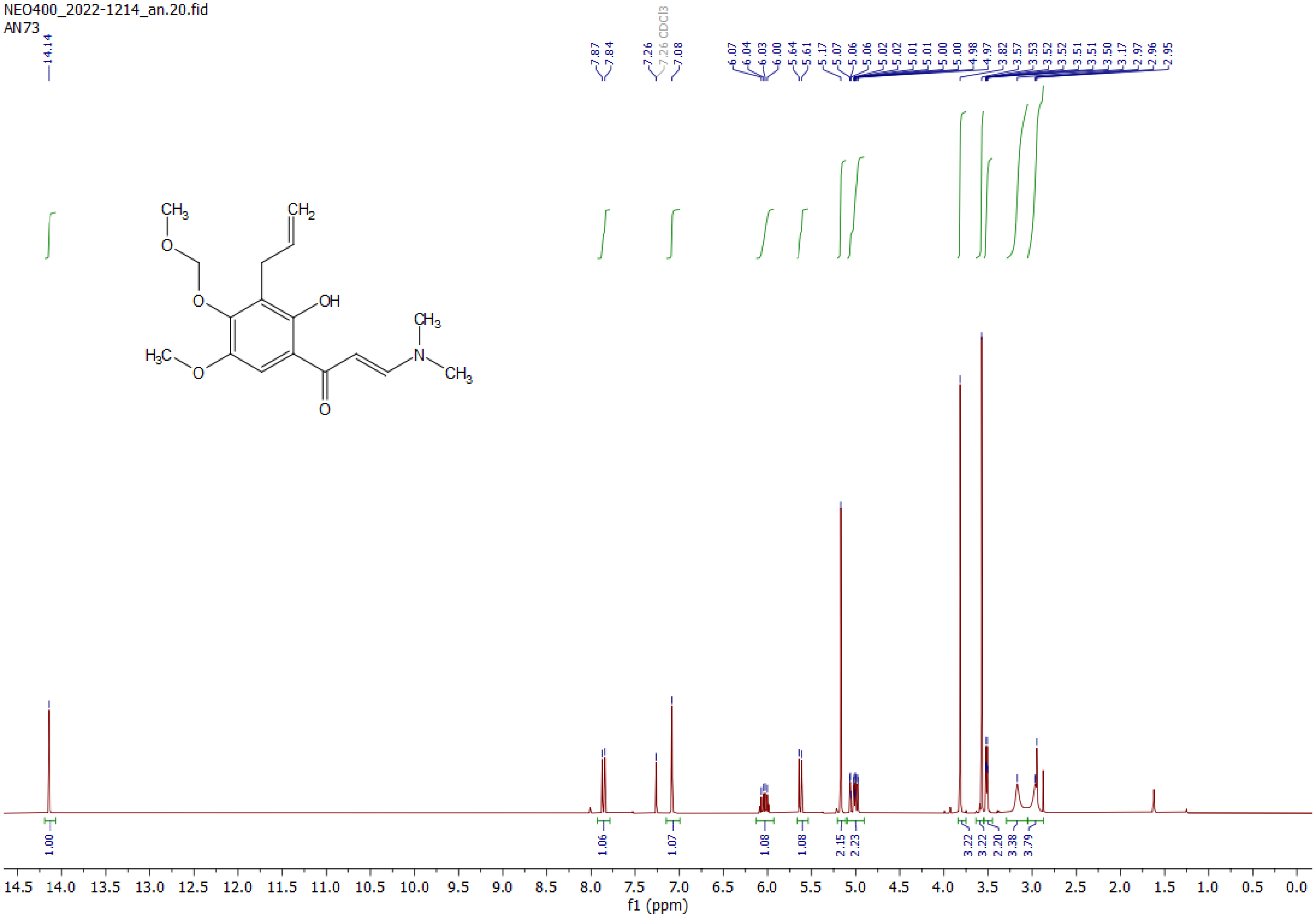

Figure S18: $^{13}C\{^1H\}$ NMR (101 MHz, $CDCl_3$) of **10**

NEO400_2022-1214_an.21.fid
AN73

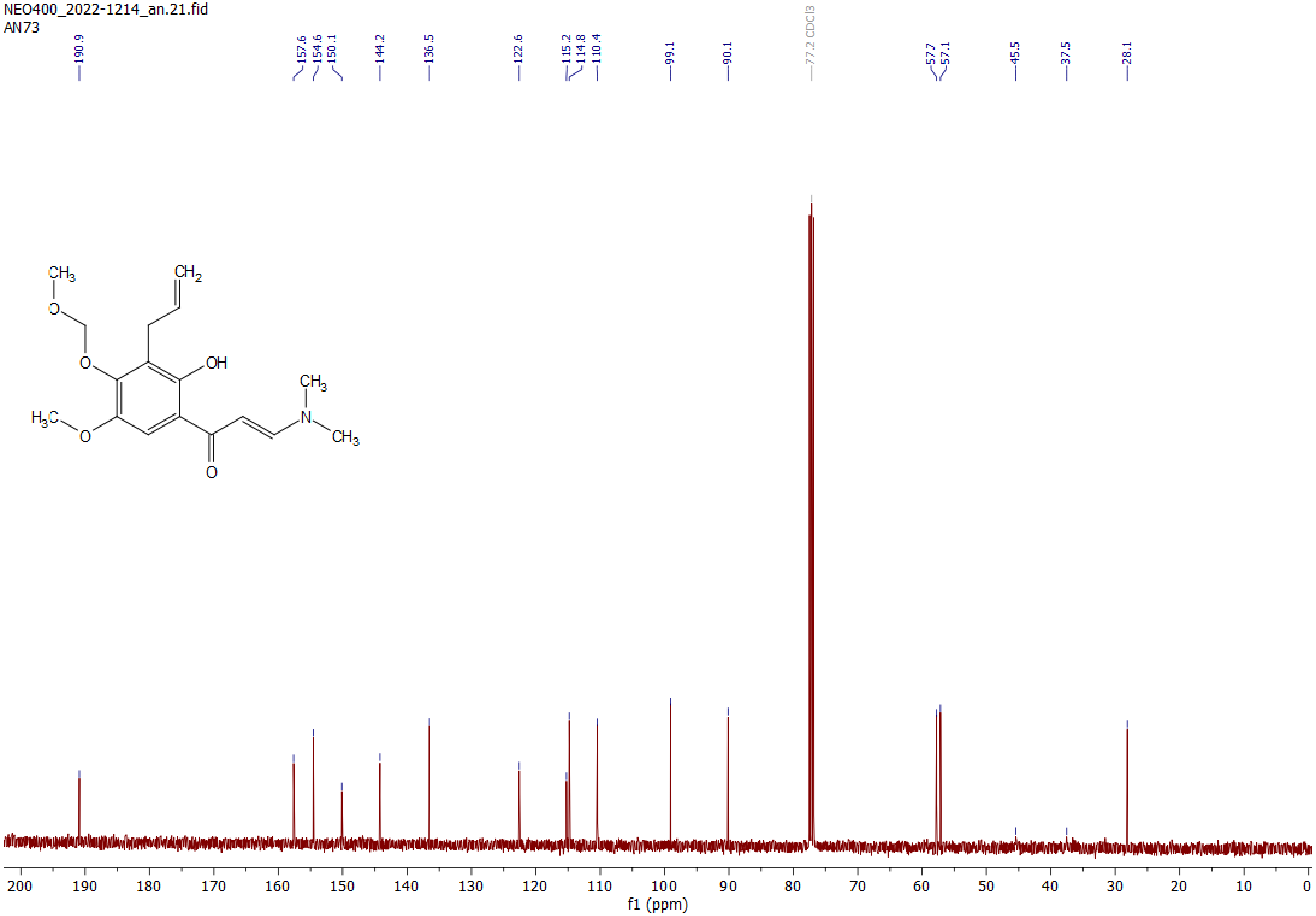

Figure S19: ^{1}H NMR (400 MHz, $CDCl_3$) of **11**

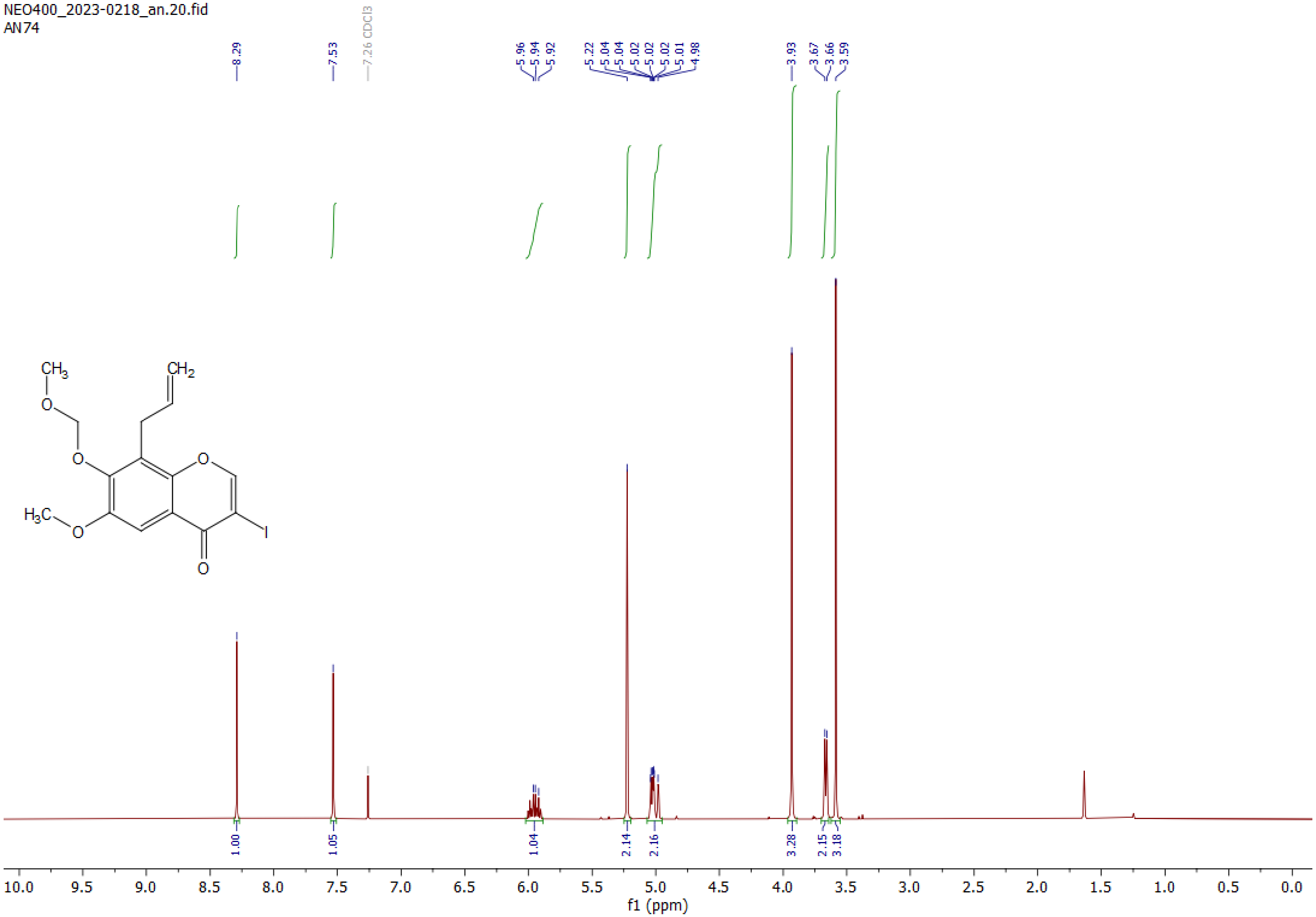

Figure S20: $^{13}C\{^{1}H\}$ NMR (101 MHz, $CDCl_3$) of **11**

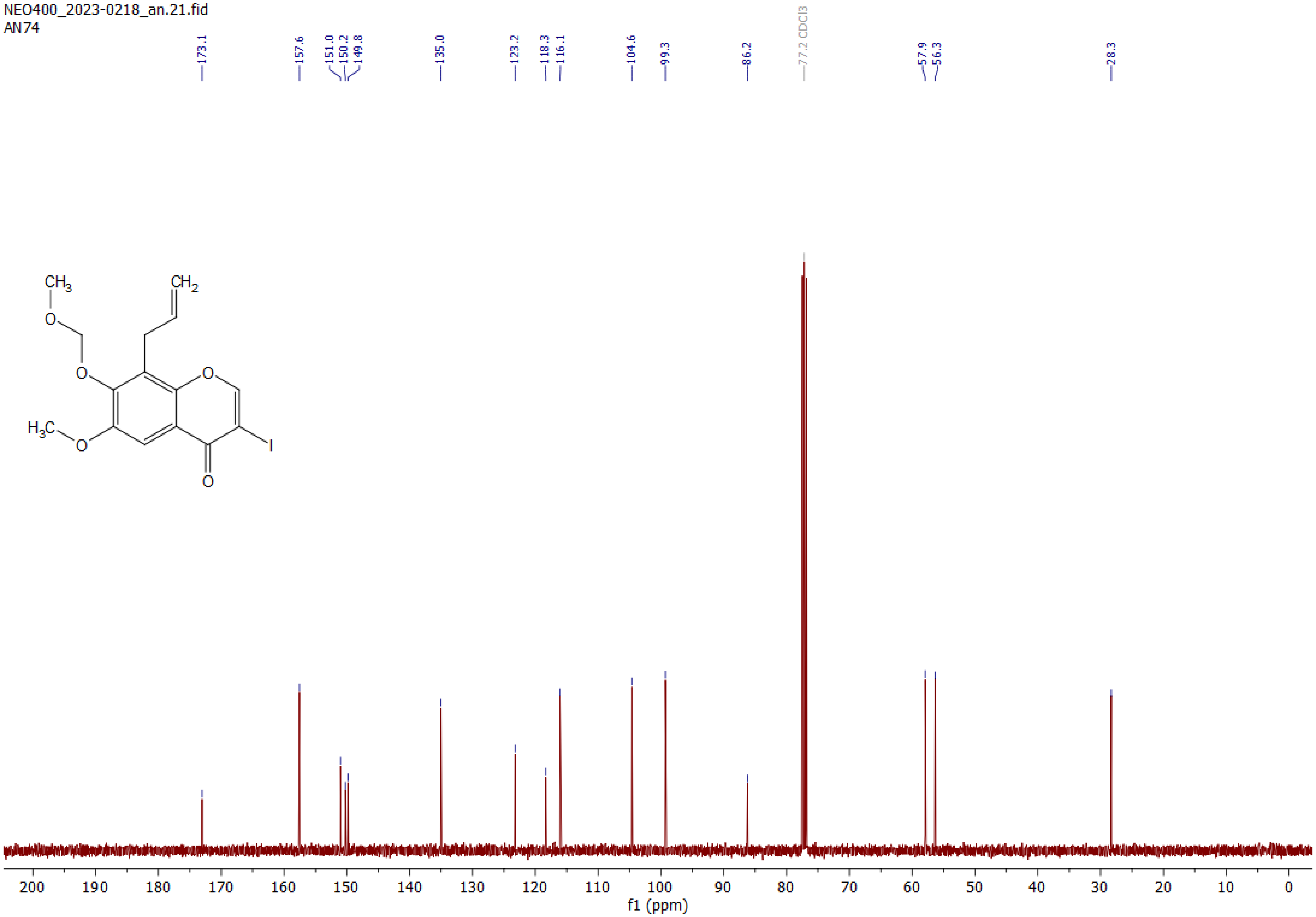

Figure S21: ^{1}H NMR (400 MHz, $CDCl_3$) of **12**

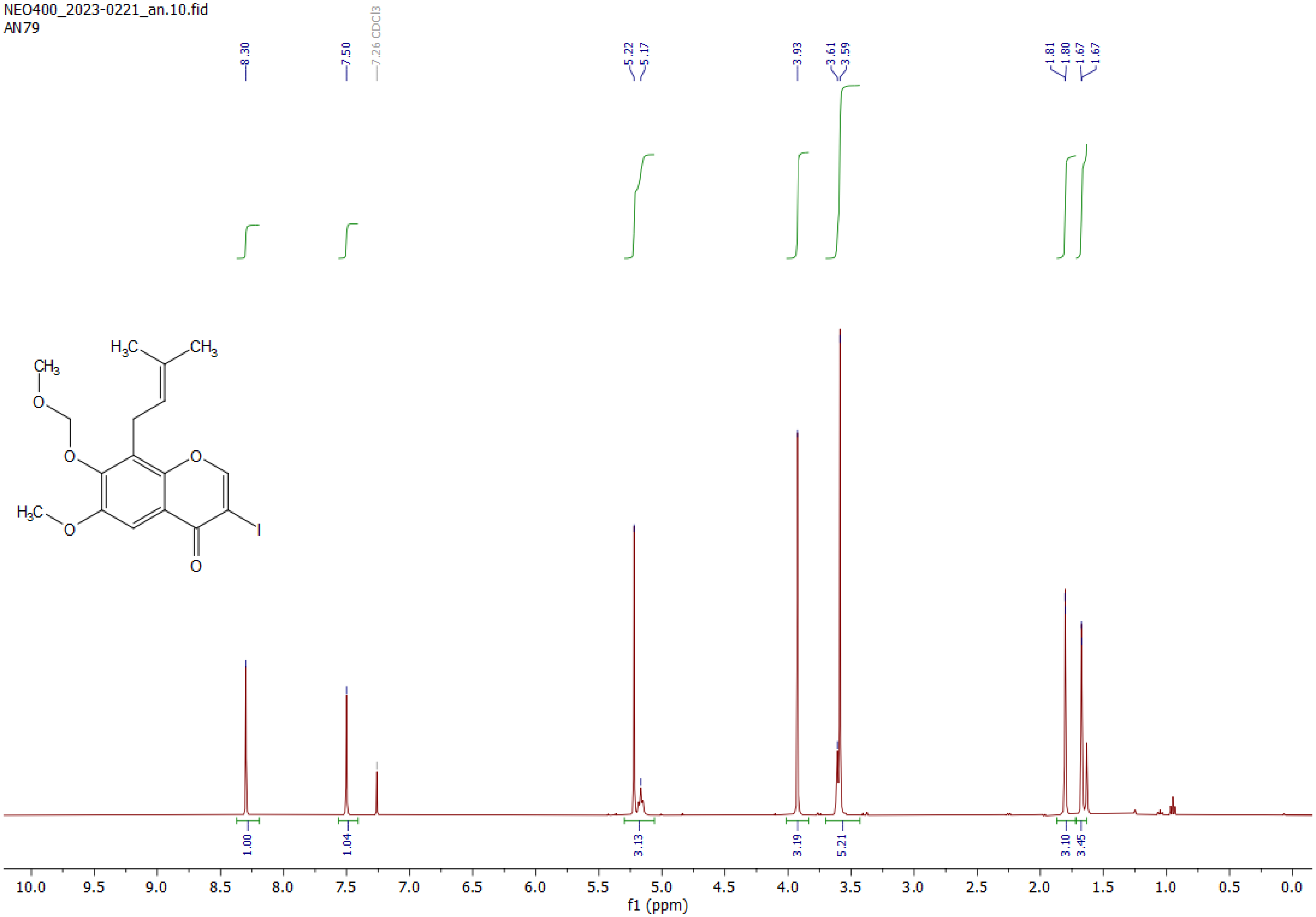

Figure S22: $^{13}C\{^1H\}$ NMR (101 MHz, $CDCl_3$) of **12**

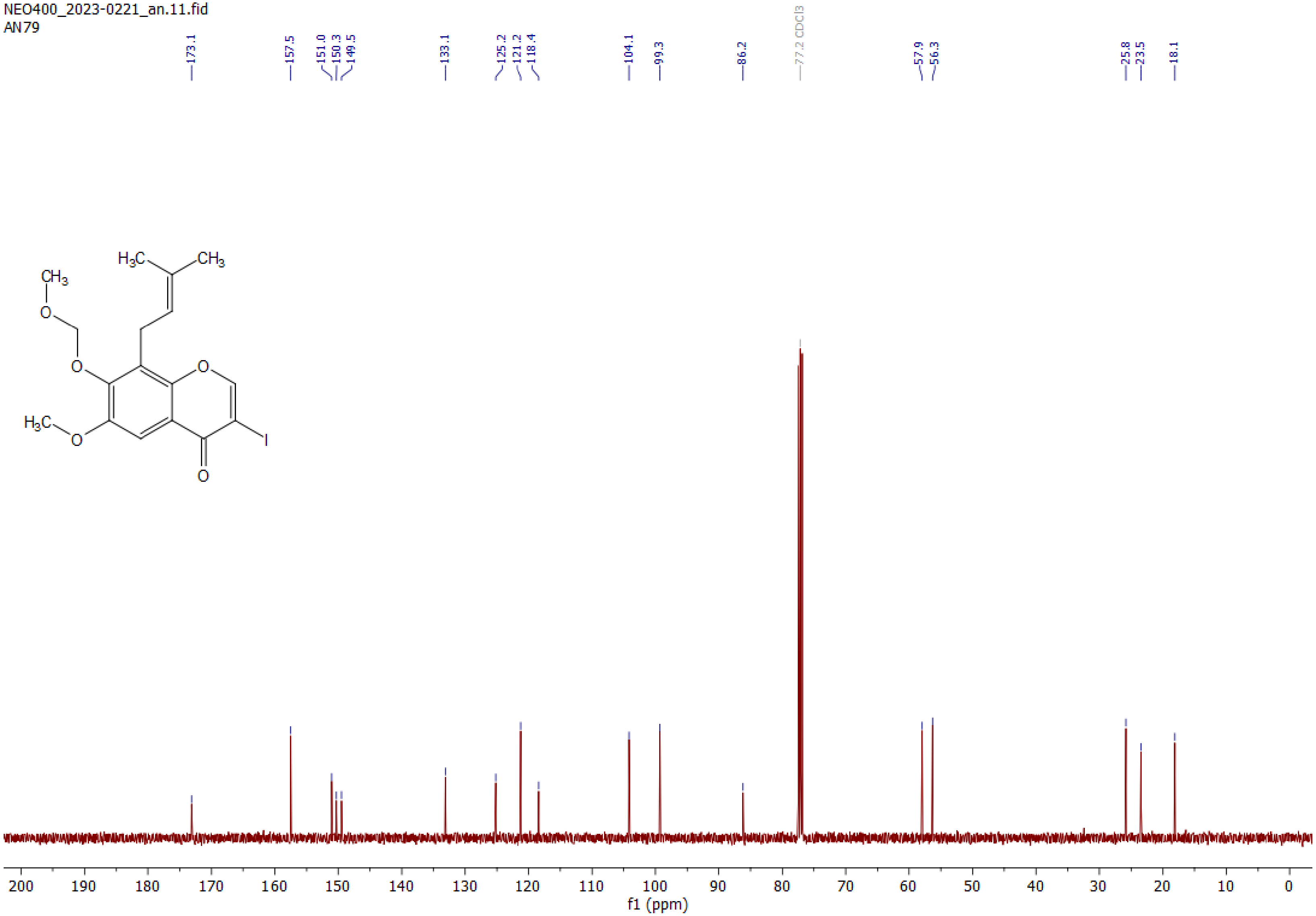

Figure S23: ^{1}H NMR (400 MHz, $CDCl_3$) of **14**

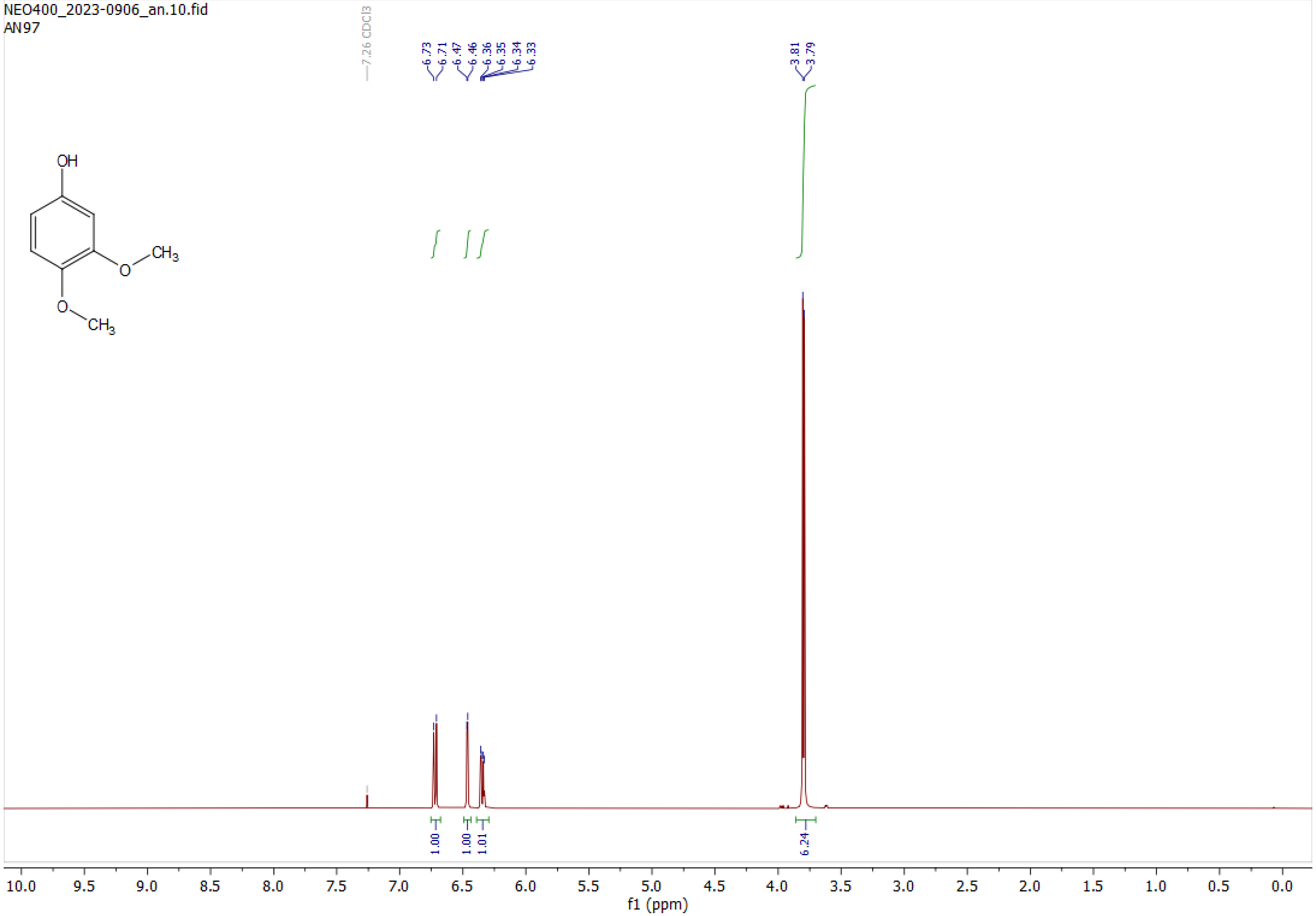

Figure S24: $^{13}C\{^{1}H\}$ NMR (101 MHz, $CDCl_3$) of **14**

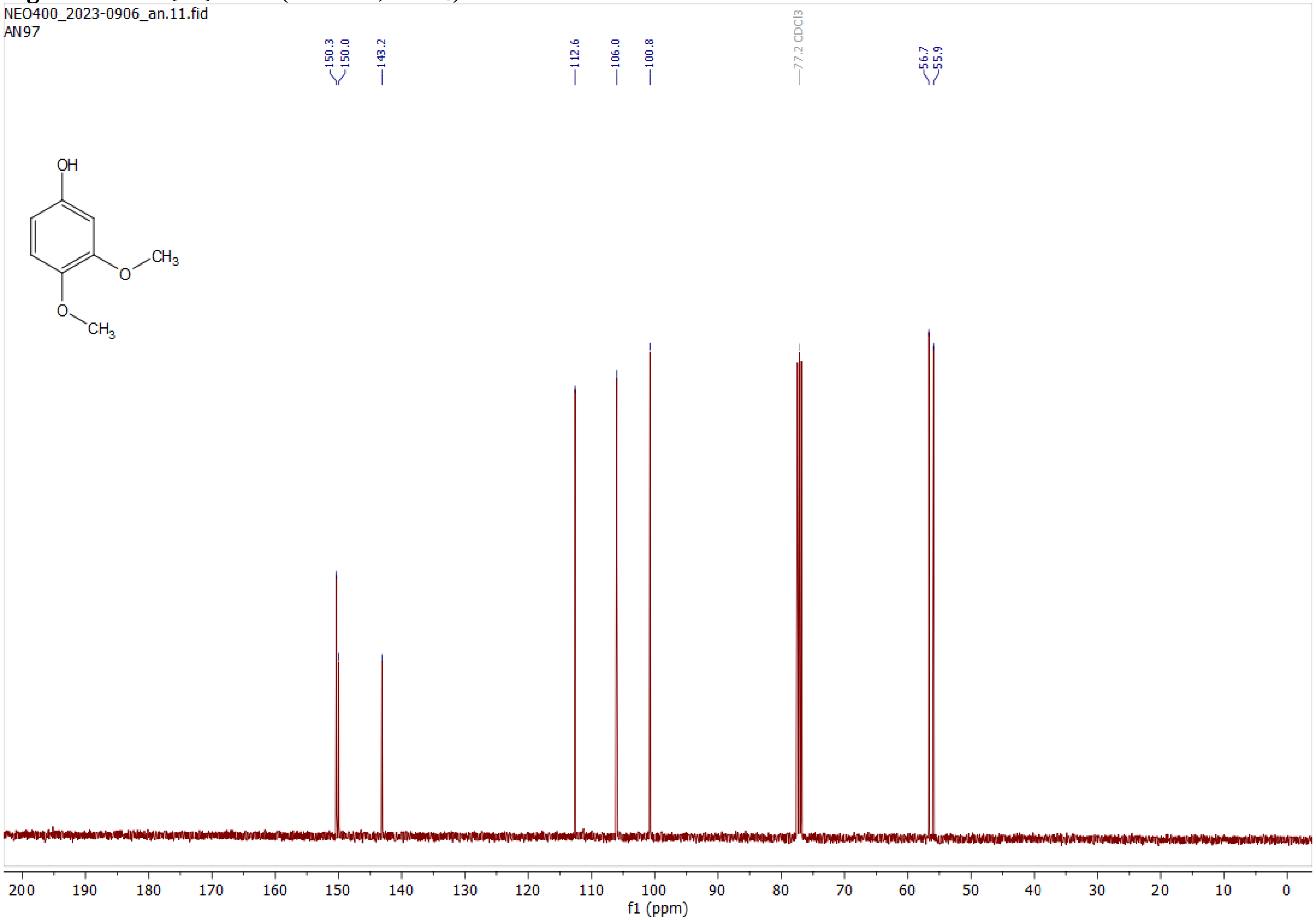

Figure S25: ^{1}H NMR (400 MHz, $CDCl_3$) of **15**

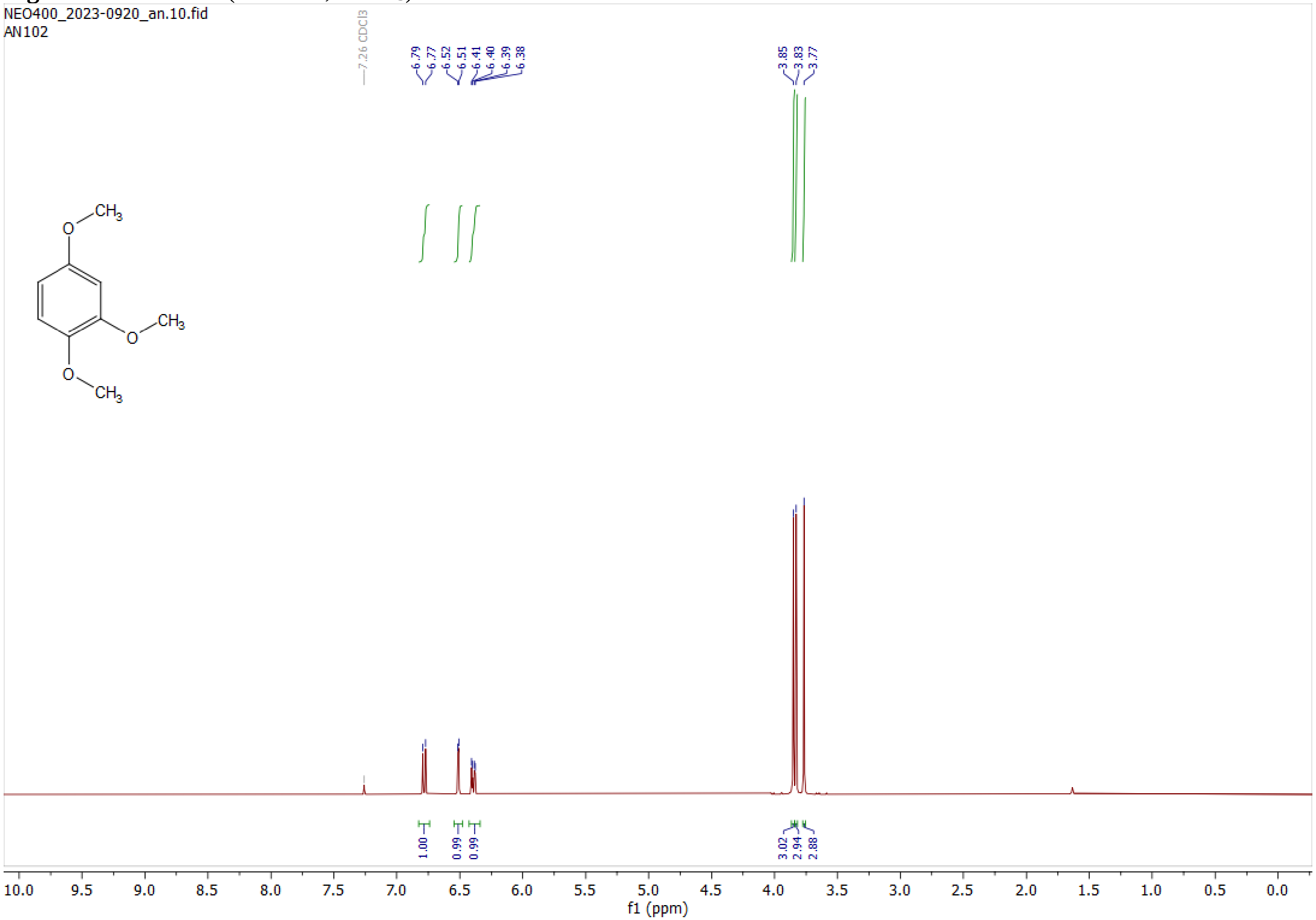

Figure S26: $^{13}C\{^1H\}$ NMR (101 MHz, $CDCl_3$) of **15**

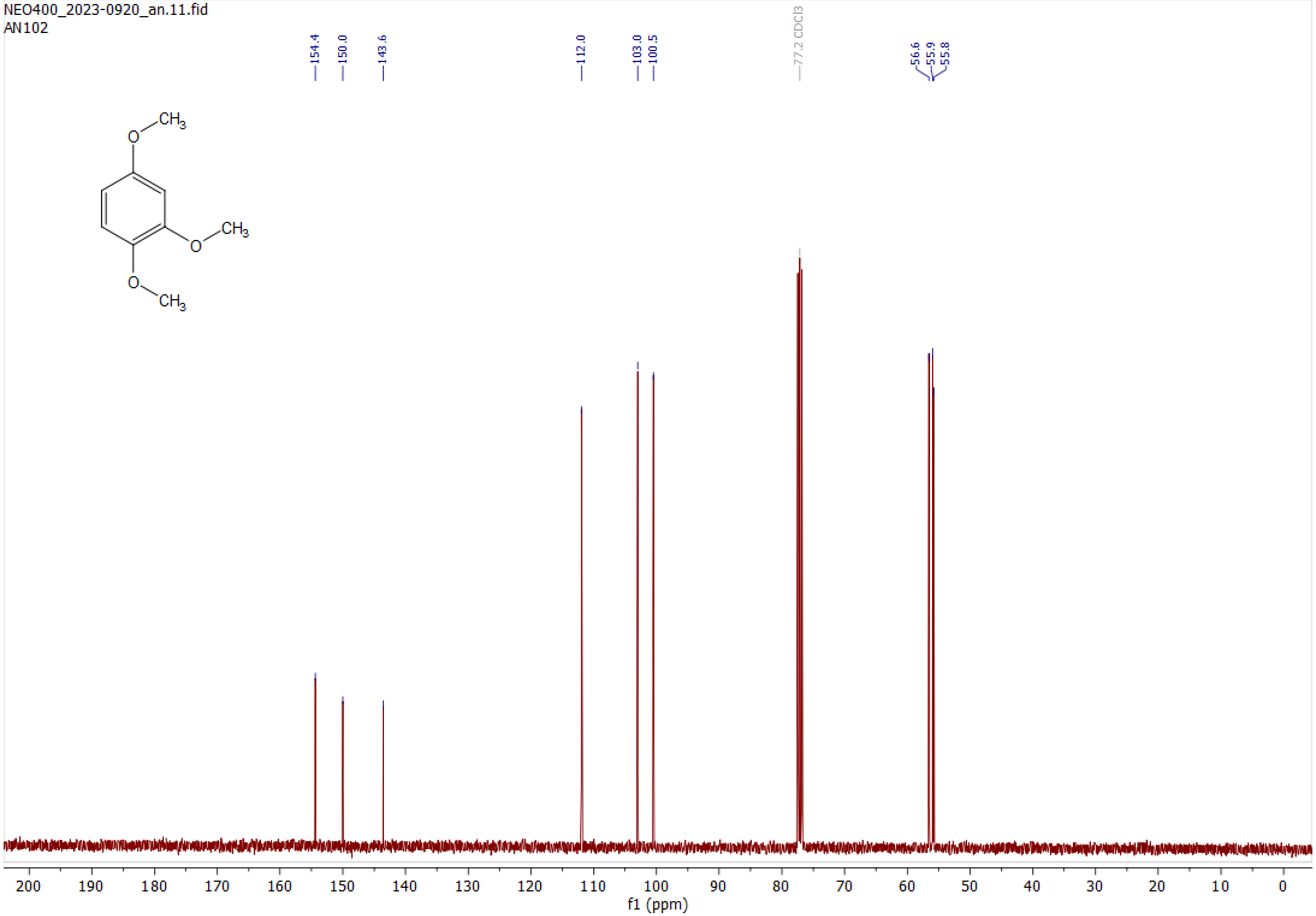

Figure S27: ^{1}H NMR (400 MHz, $CDCl_3$) of **16**

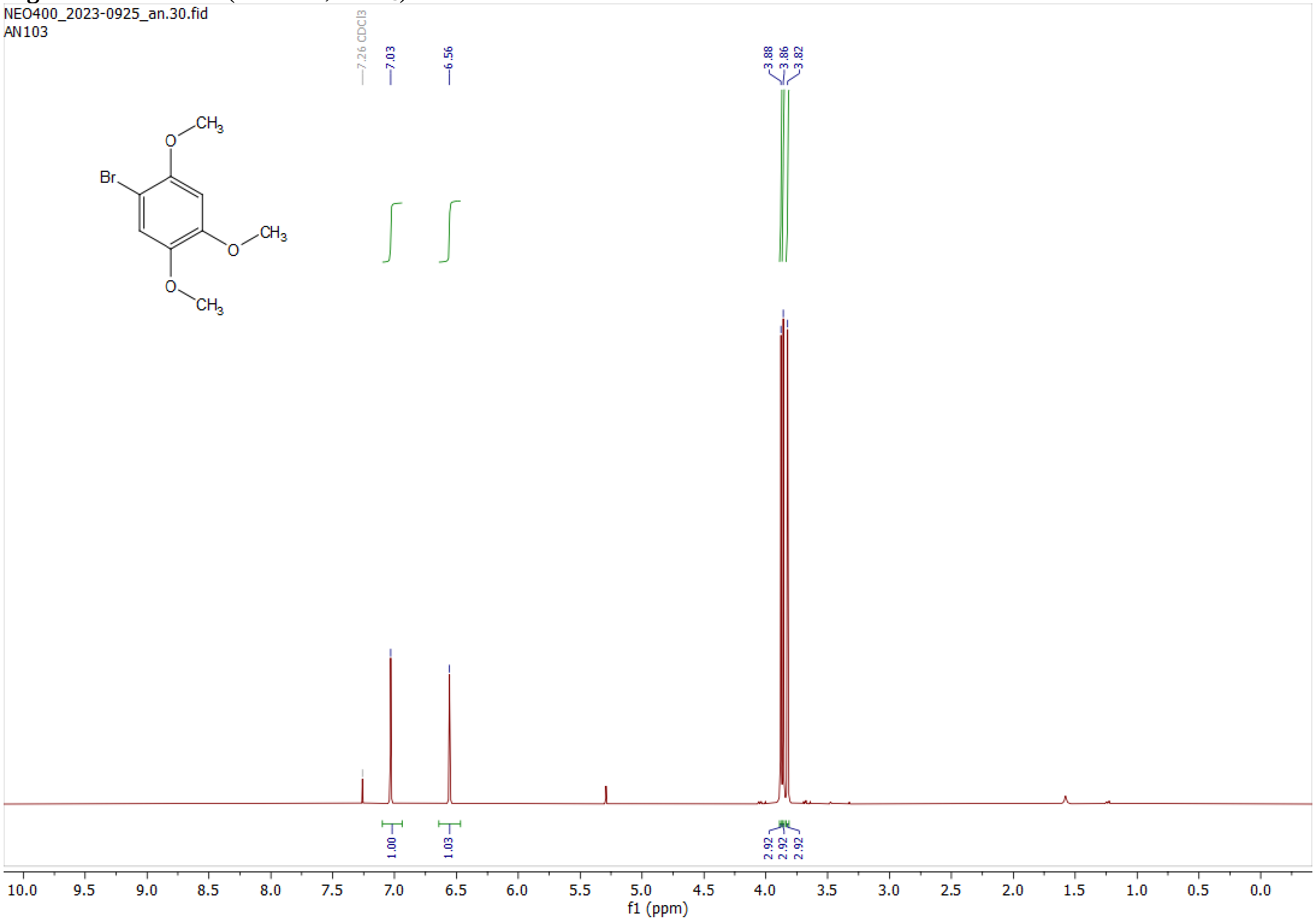

Figure S28: $^{13}C\{^1H\}$ NMR (101 MHz, $CDCl_3$) of **16**

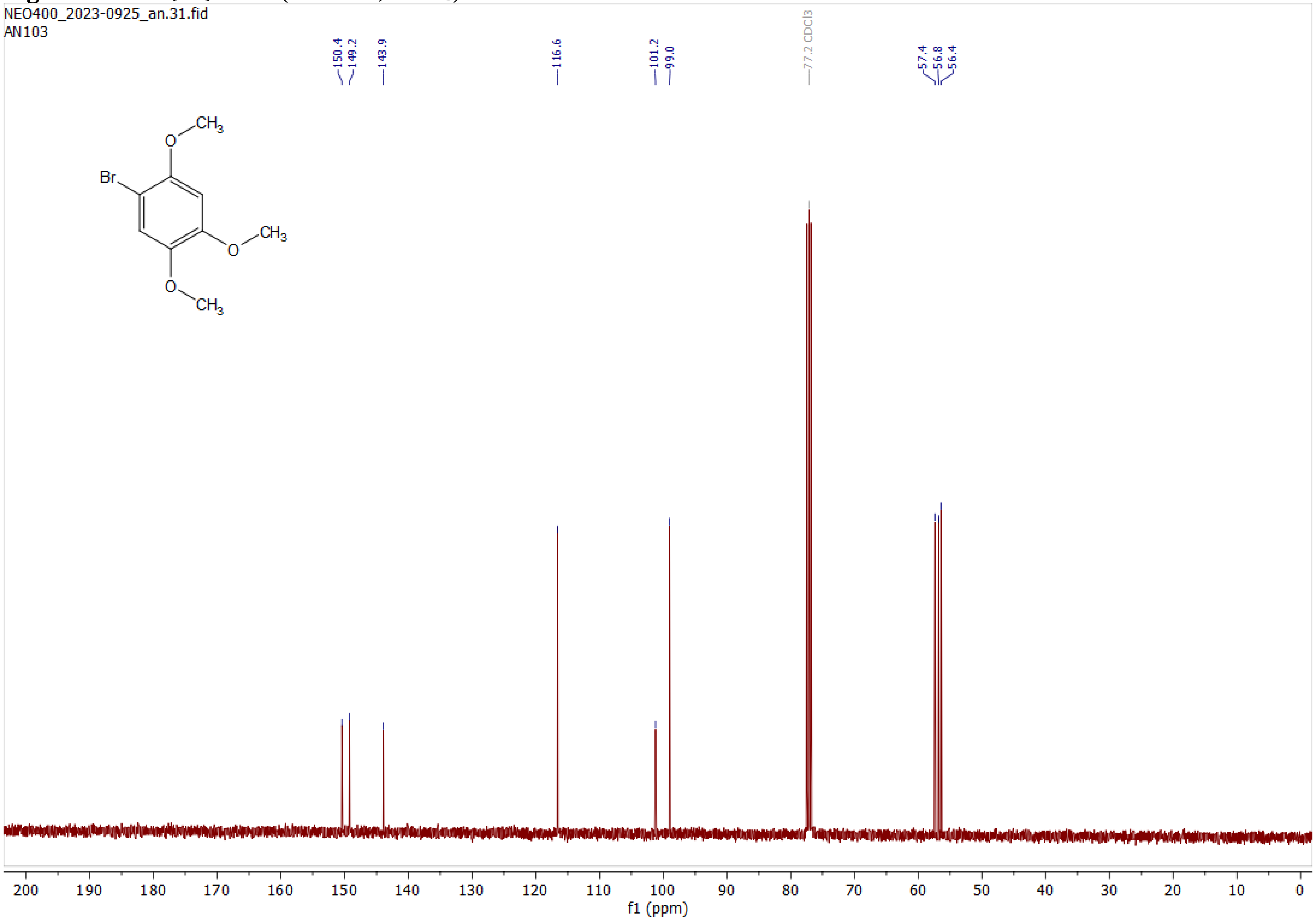

Figure S29: ^{1}H NMR (400 MHz, $CDCl_3$) of **3e**

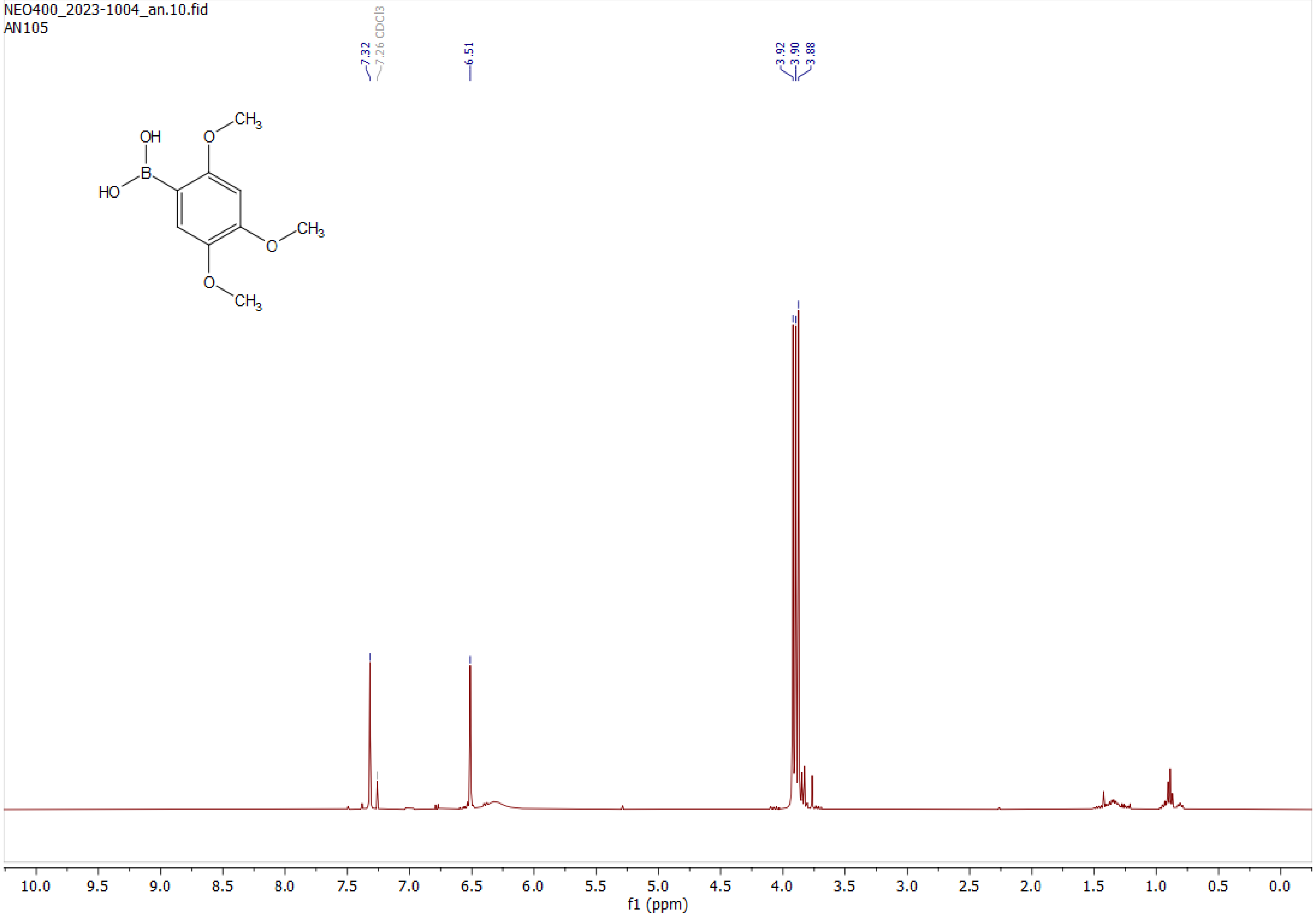

Figure S30: $^{13}C\{^{1}H\}$ NMR (101 MHz, $CDCl_3$) of **3e**

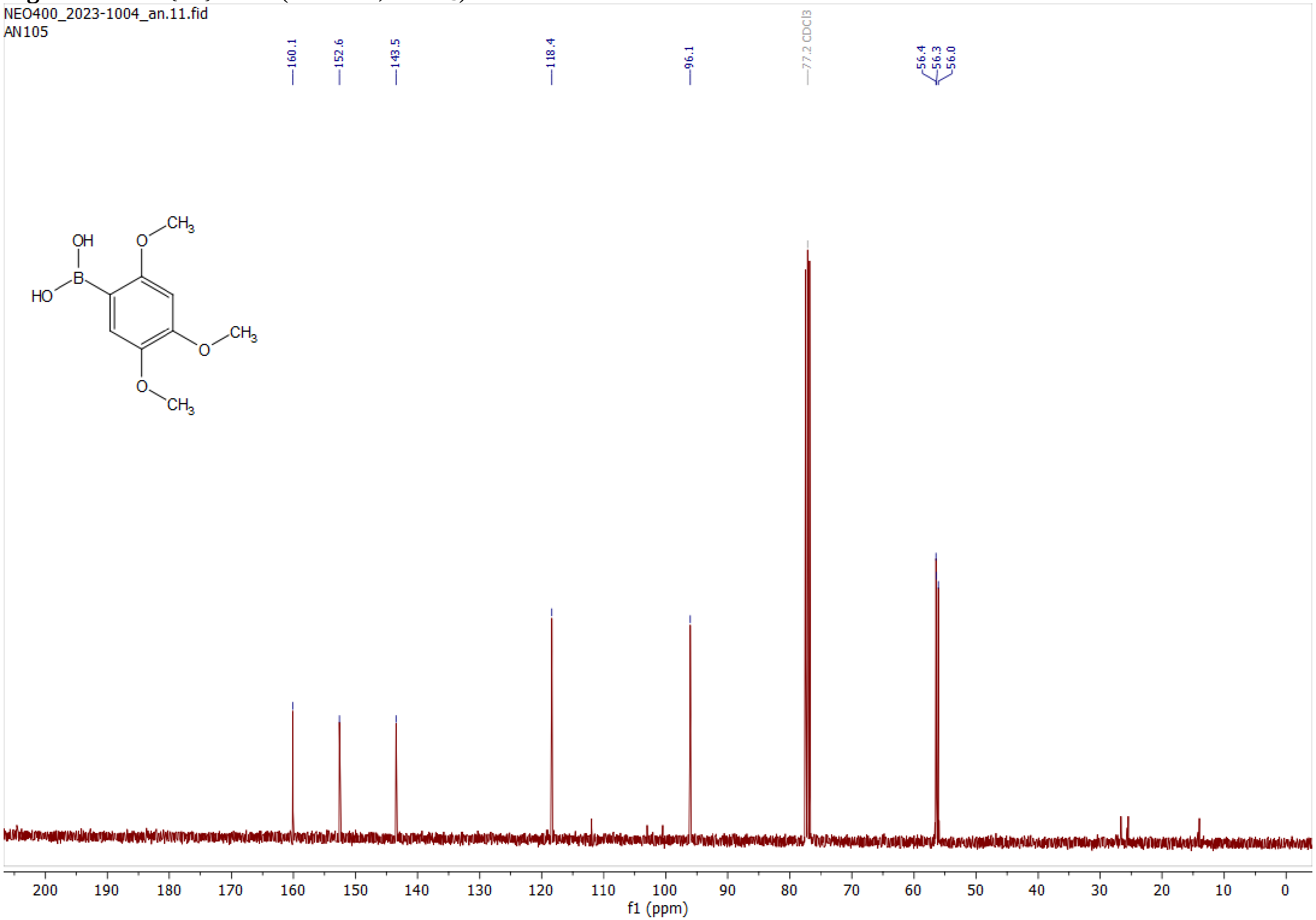

Figure S31: ^{1}H NMR (400 MHz, $CDCl_3$) of **18**

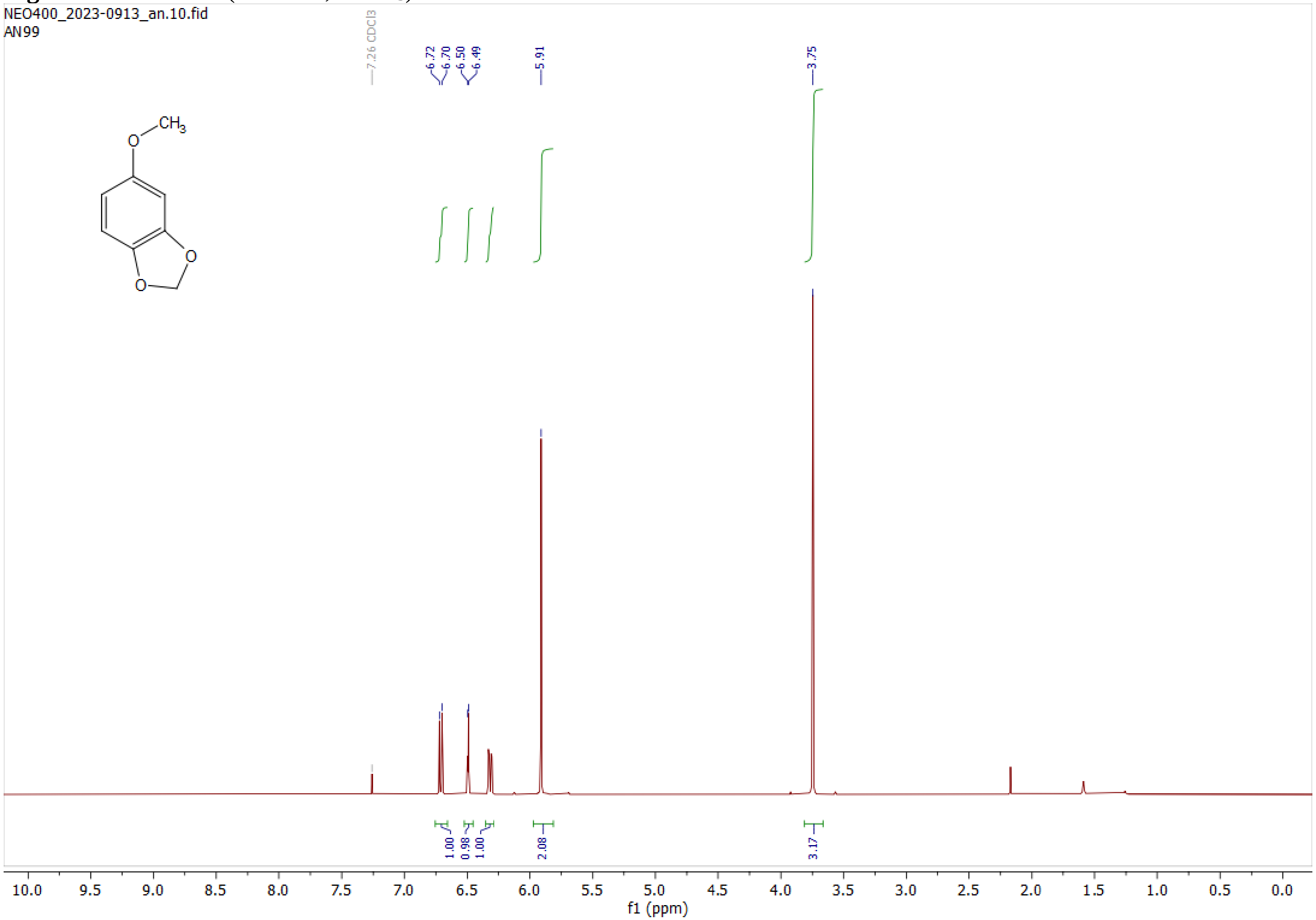

Figure S32: $^{13}C\{^1H\}$ NMR (101 MHz, $CDCl_3$) of **18**

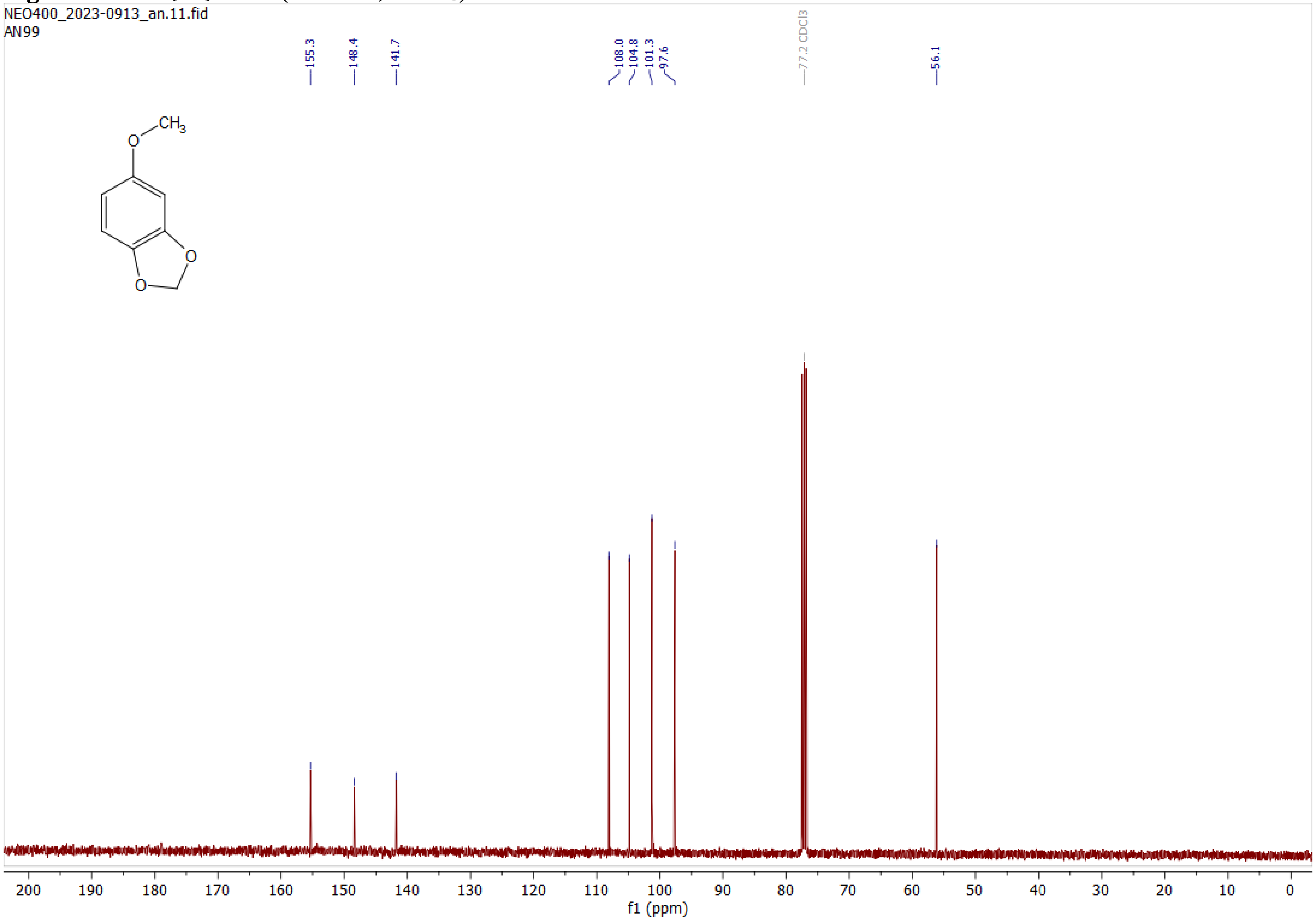

Figure S33: ^{1}H NMR (400 MHz, $CDCl_3$) of **19**

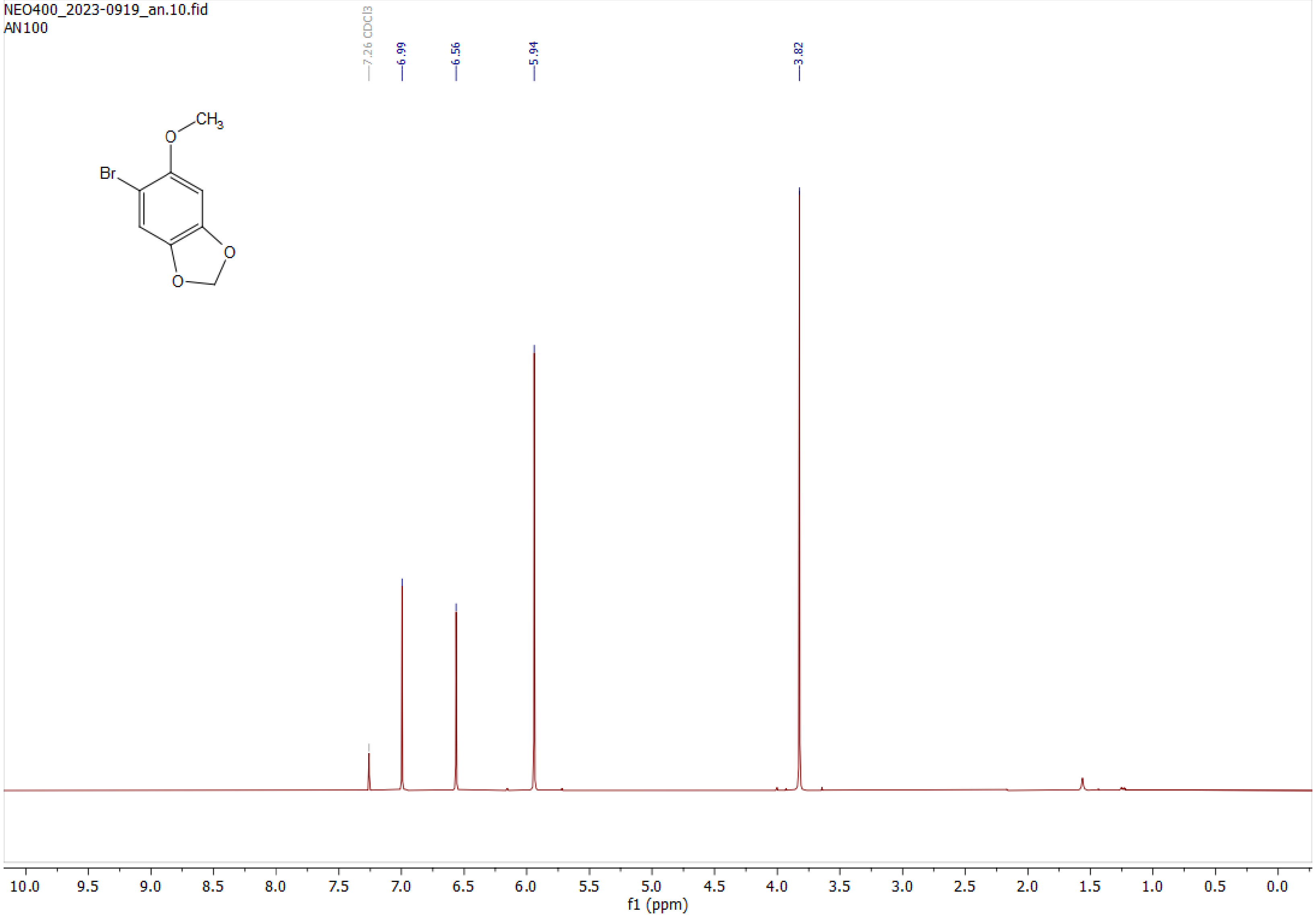

Figure S34: $^{13}C\{^{1}H\}$ NMR (101 MHz, $CDCl_3$) of **19**

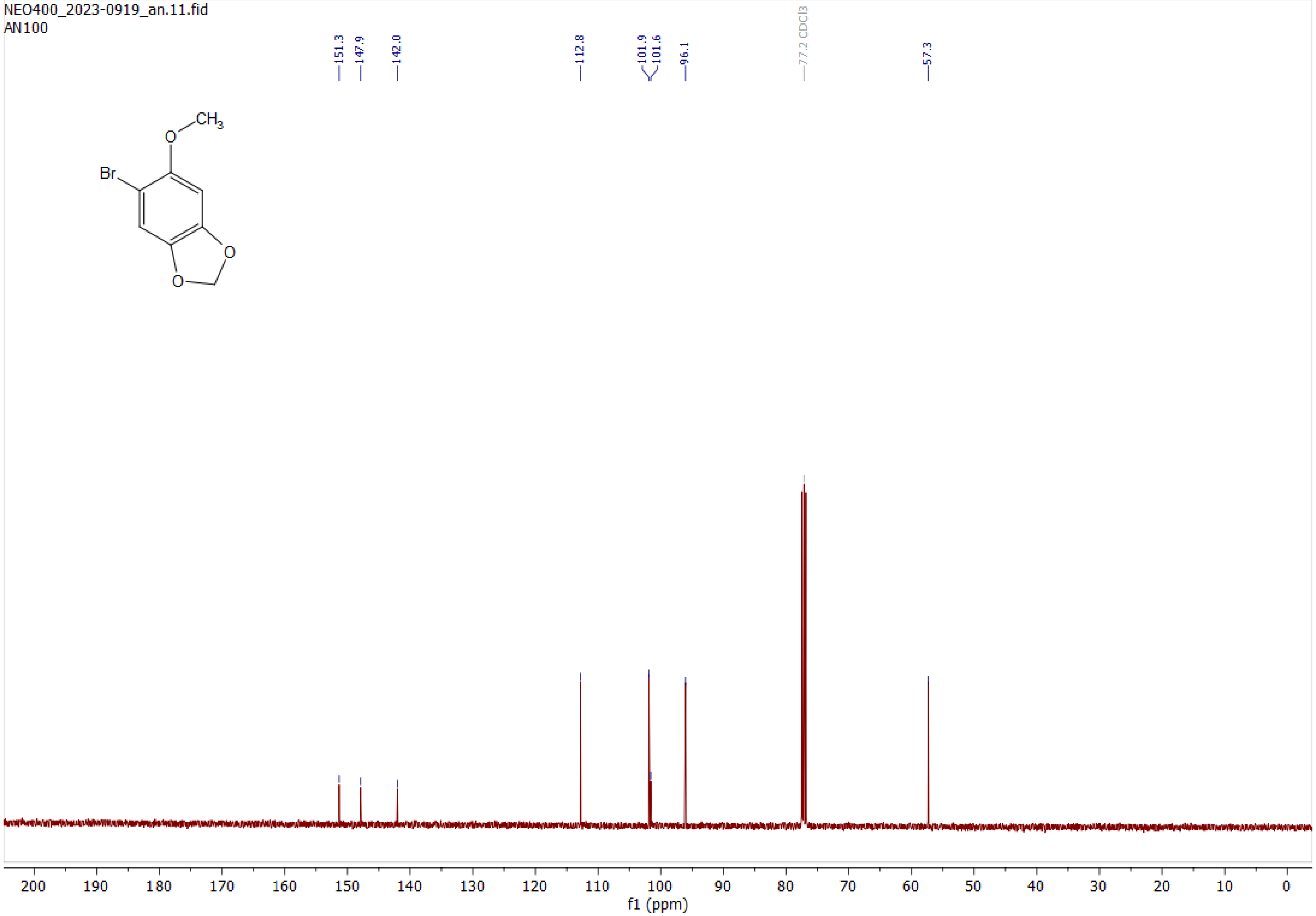

Figure S35: ^{1}H NMR (128 MHz, DMSO-d_6) of **3f**

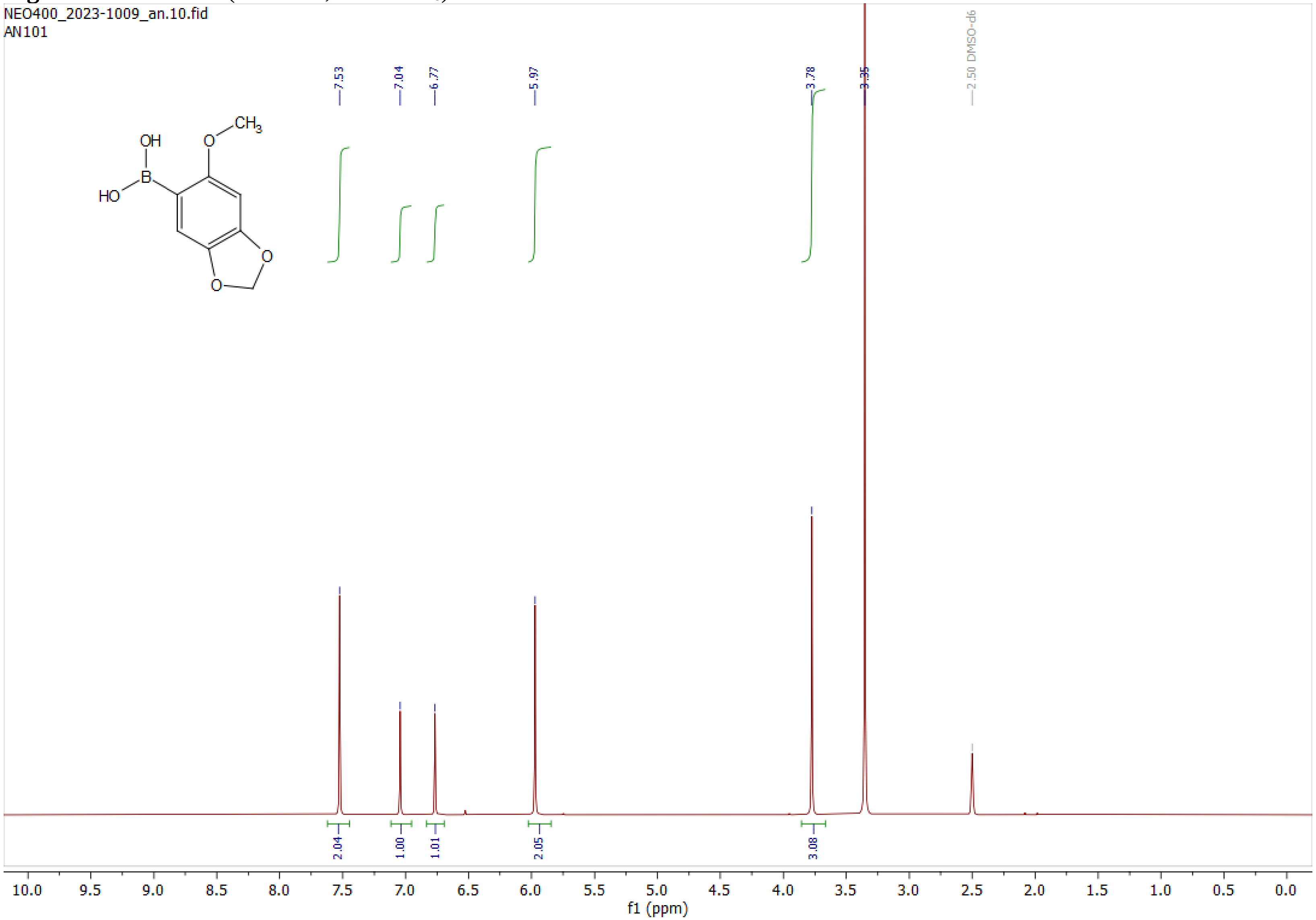

Figure S36: $^{13}C\{^{1}H\}$ NMR (101 MHz, DMSO-d_6) of **3f**

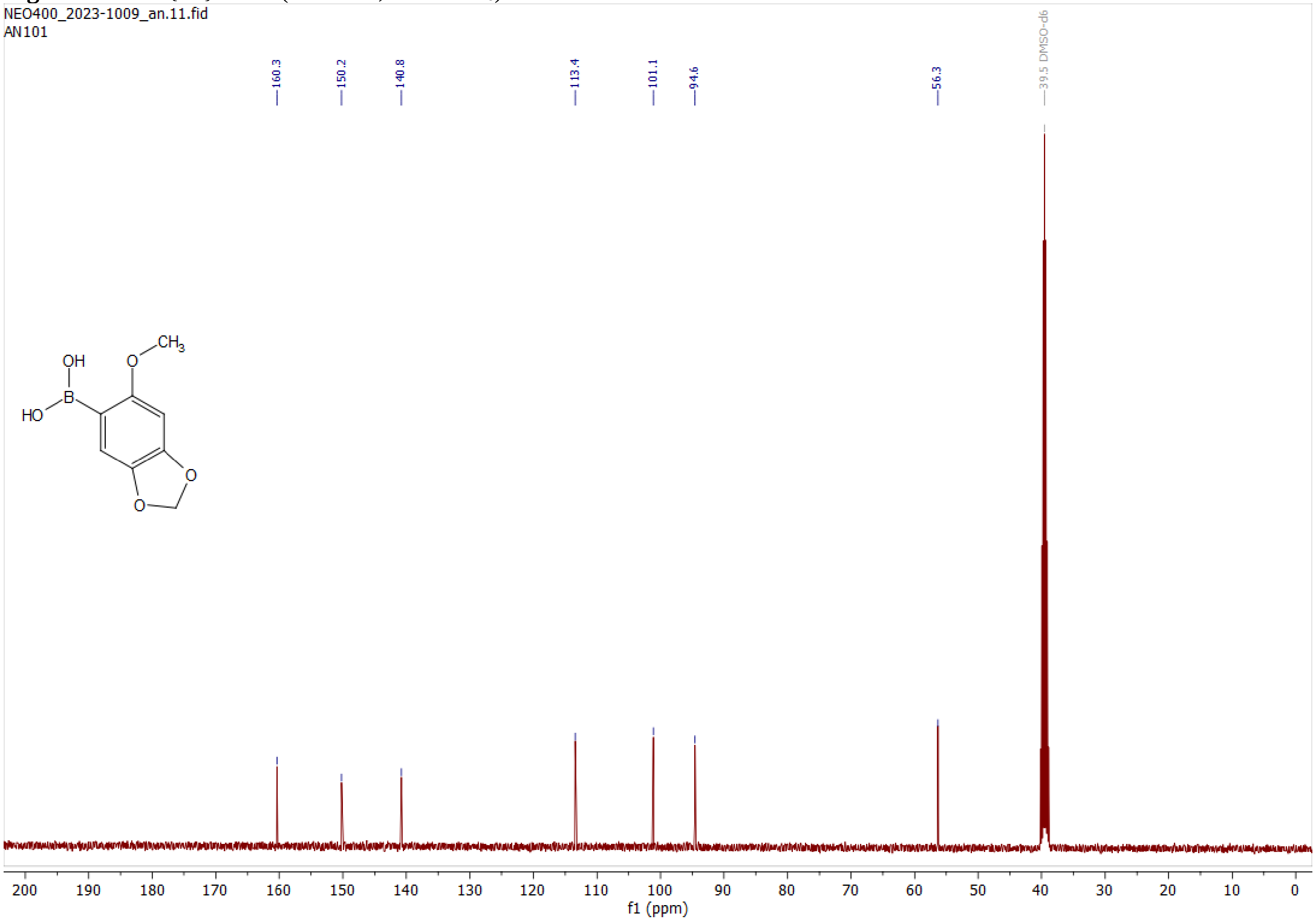

Figure S37: $^{11}B\{^{1}H\}$ NMR (101 MHz, DMSO-d_6) of **3f**

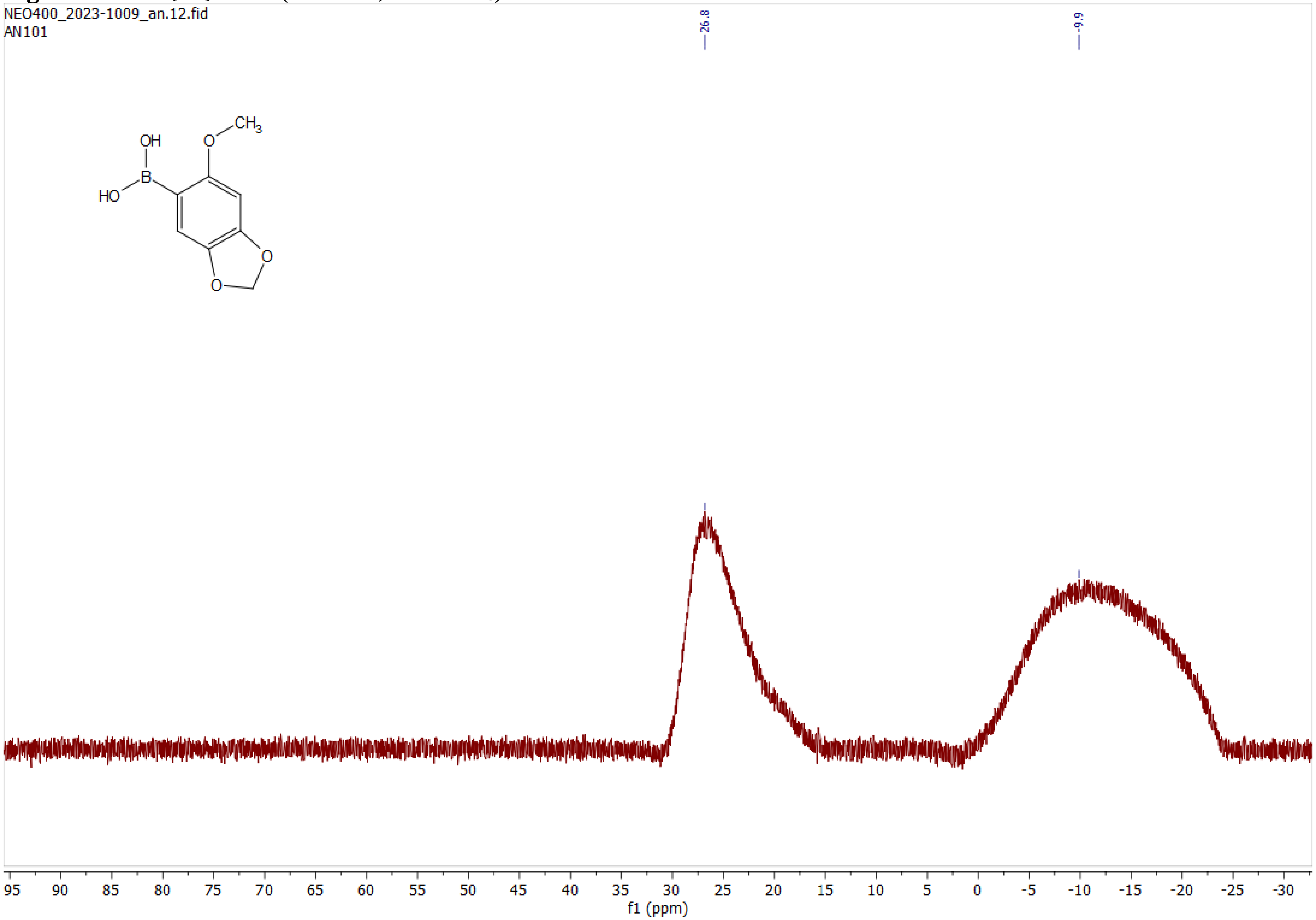

Figure S38: ^{1}H NMR (400 MHz, $CDCl_3$) of **20a**

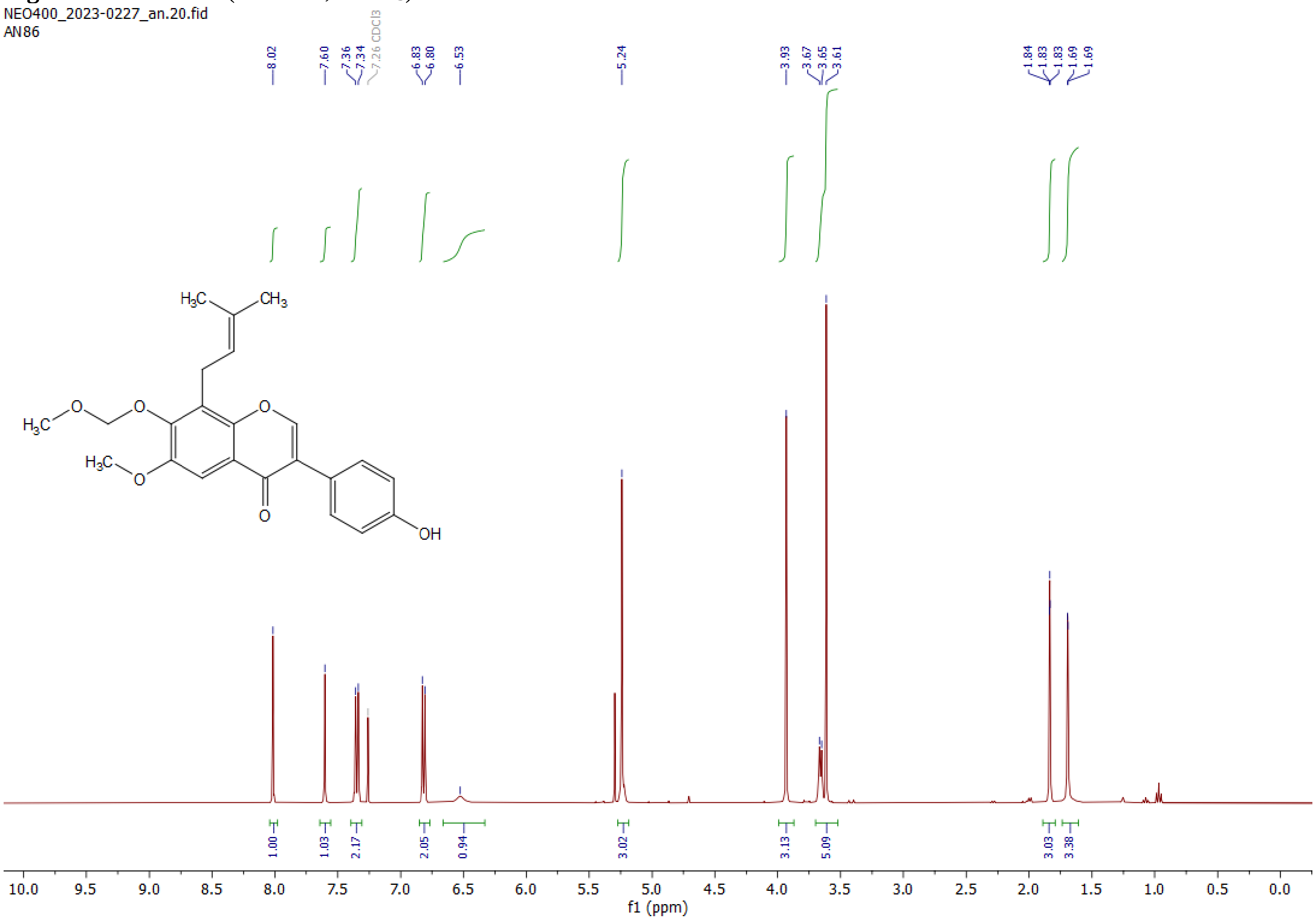

Figure S39: $^{13}C\{^{1}H\}$ NMR (101 MHz, $CDCl_3$) of **20a**

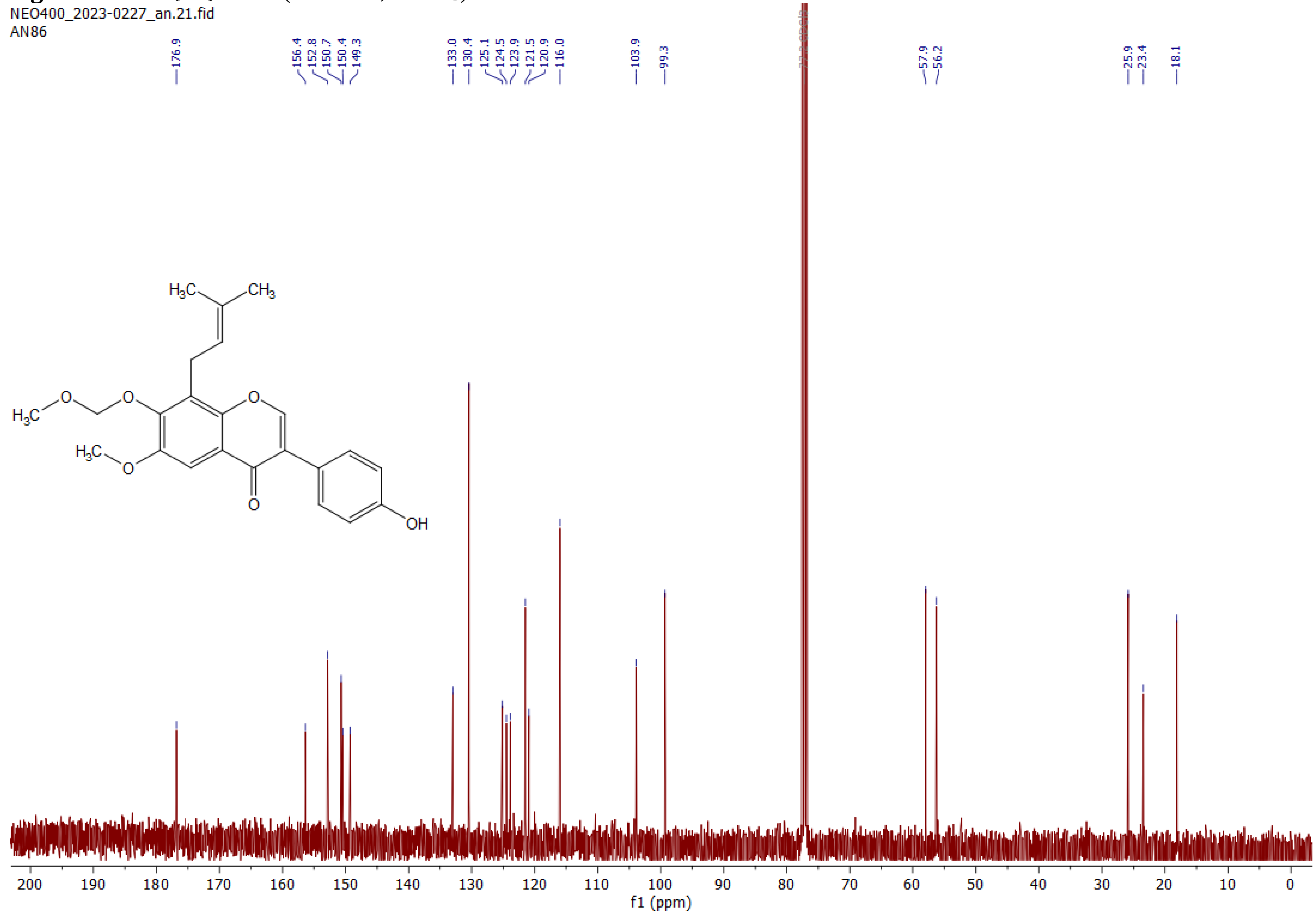

Figure S40: ^{1}H NMR (400 MHz, $CDCl_3$) of **20b**

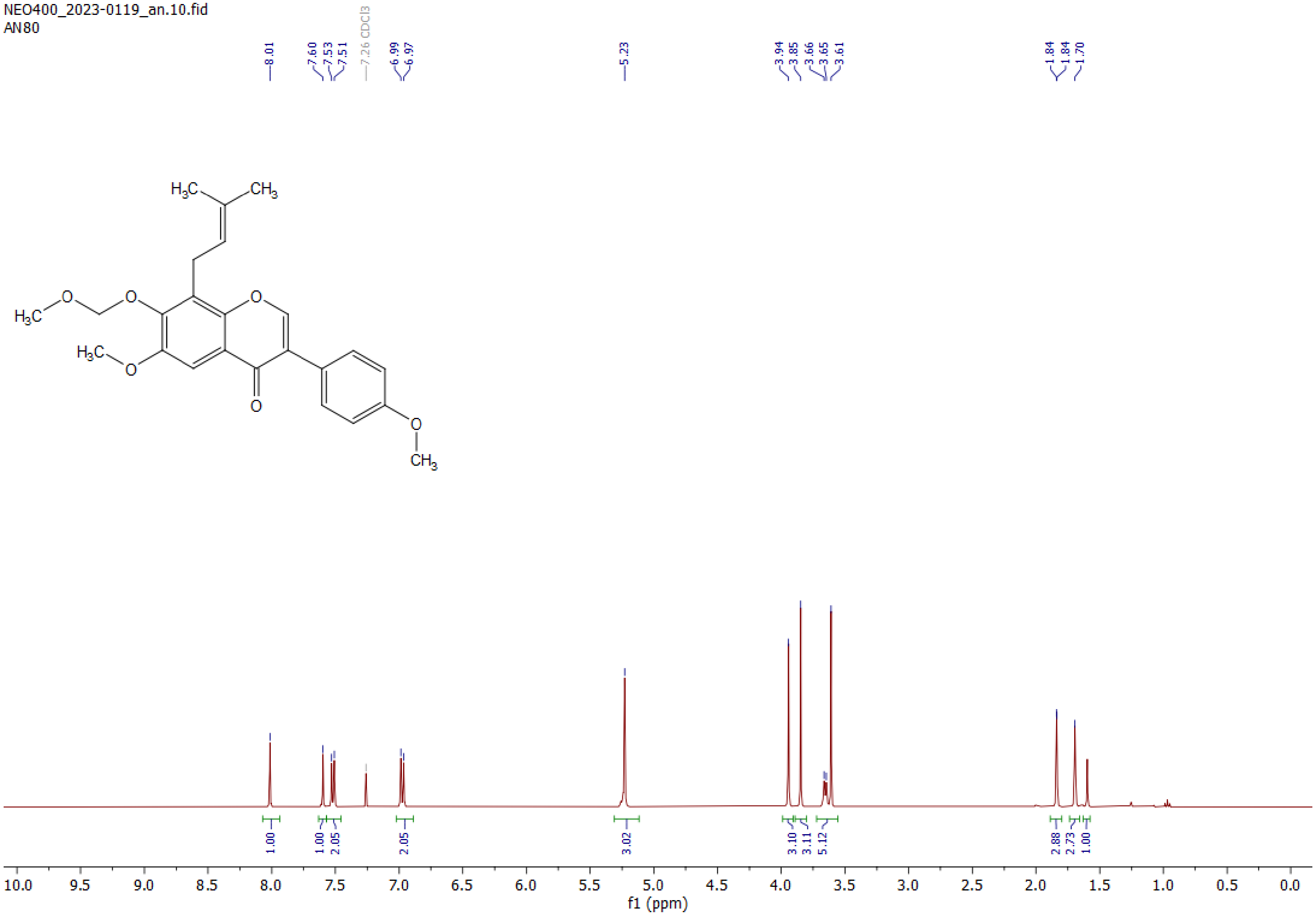

Figure S41: $^{13}C\{^{1}H\}$ NMR (101 MHz, $CDCl_3$) of **20b**

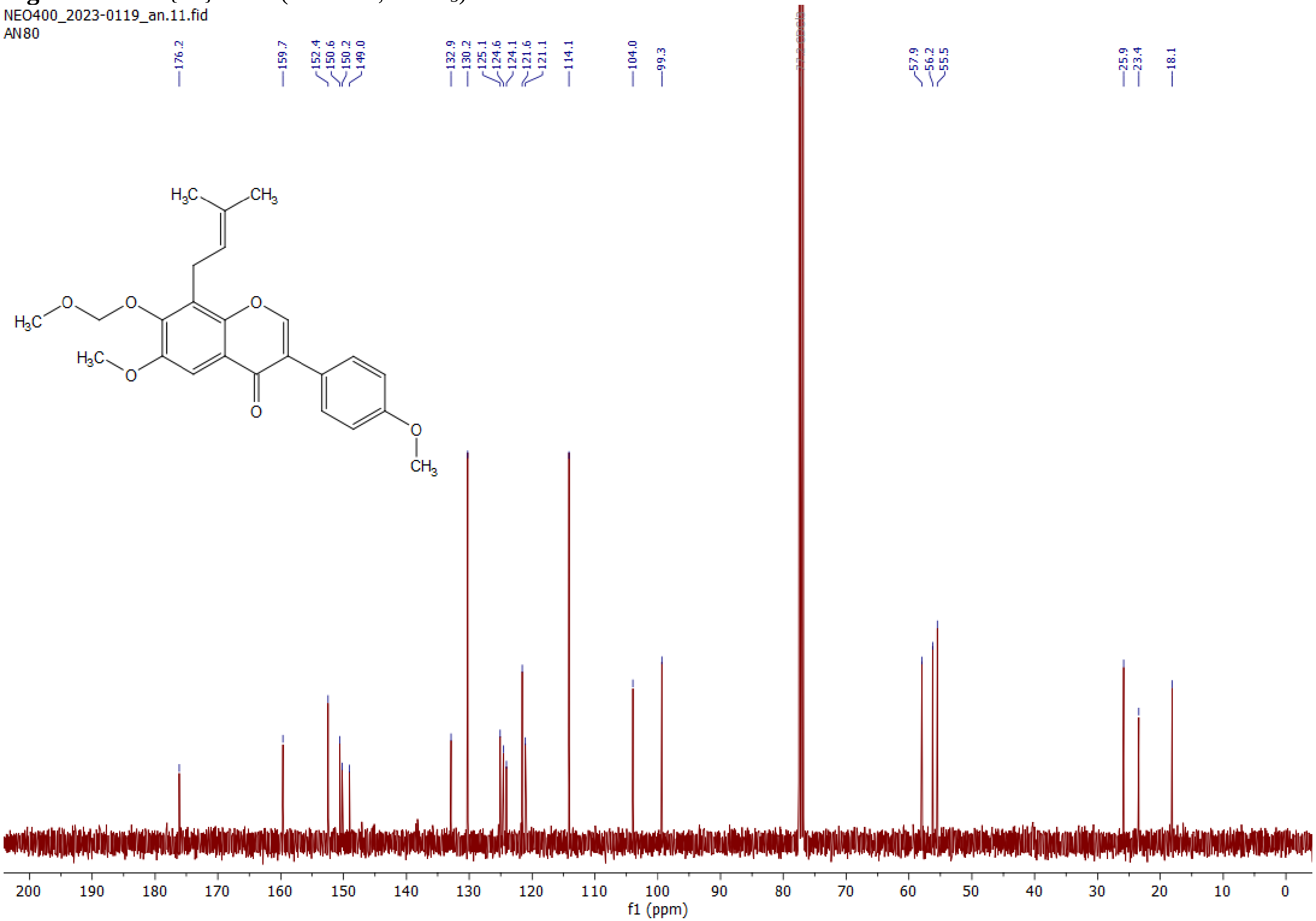

Figure S42: ^{1}H NMR (400 MHz, $CDCl_3$) of **20c**

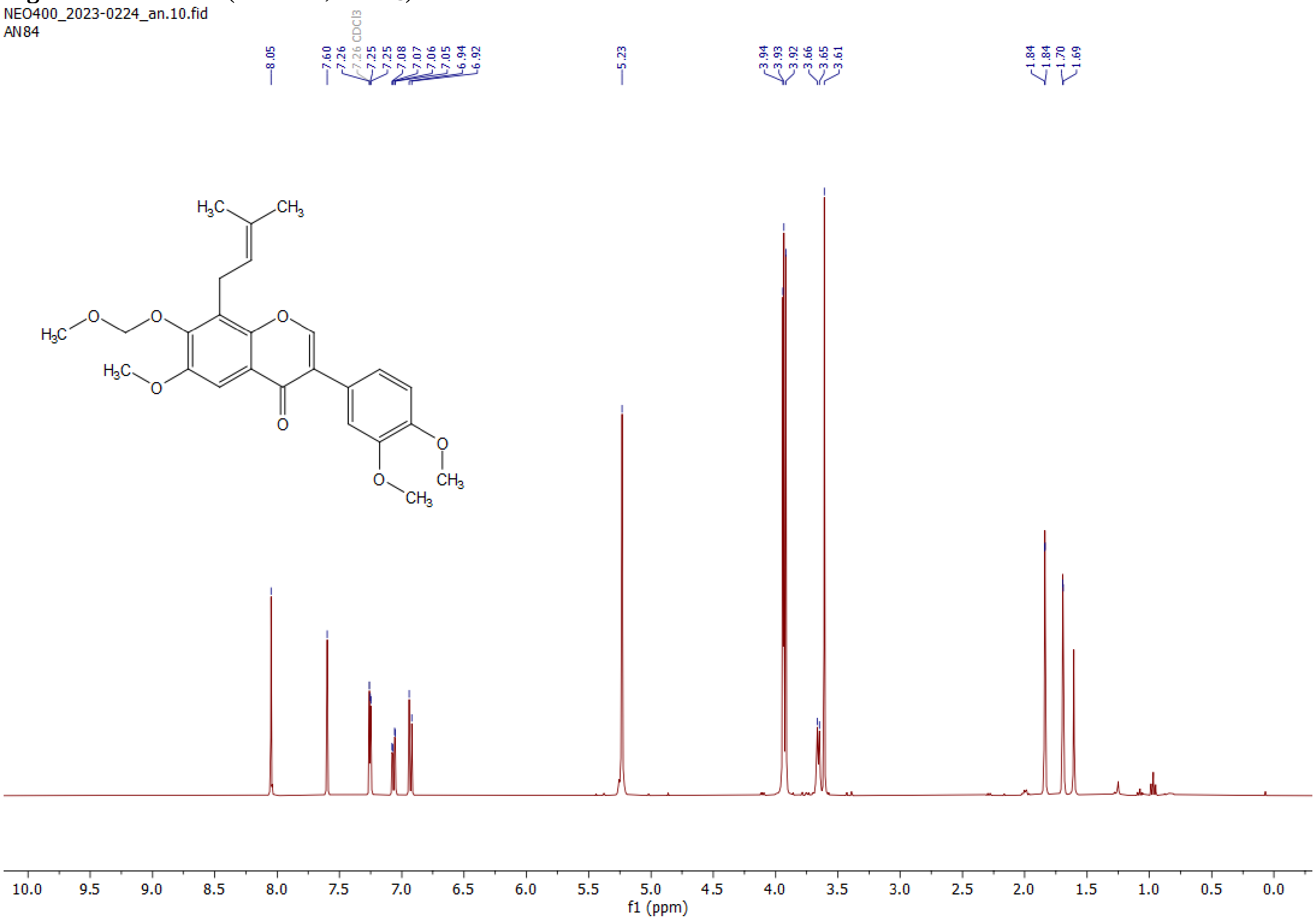

Figure S43: $^{13}C\{^{1}H\}$ NMR (101 MHz, $CDCl_3$) of **20c**

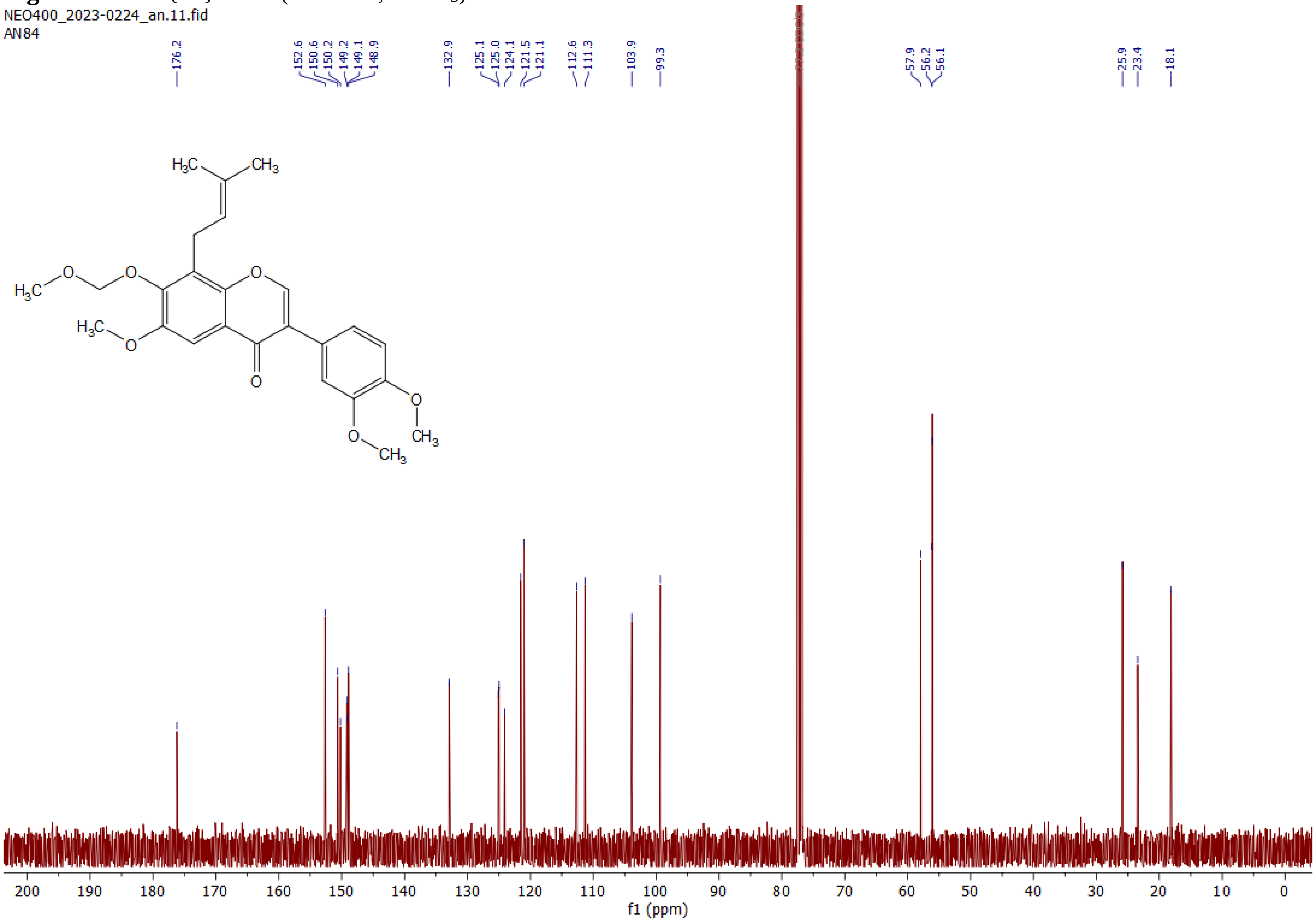

Figure S44: HSQC (400/101 MHz, $CDCl_3$) of **20c**

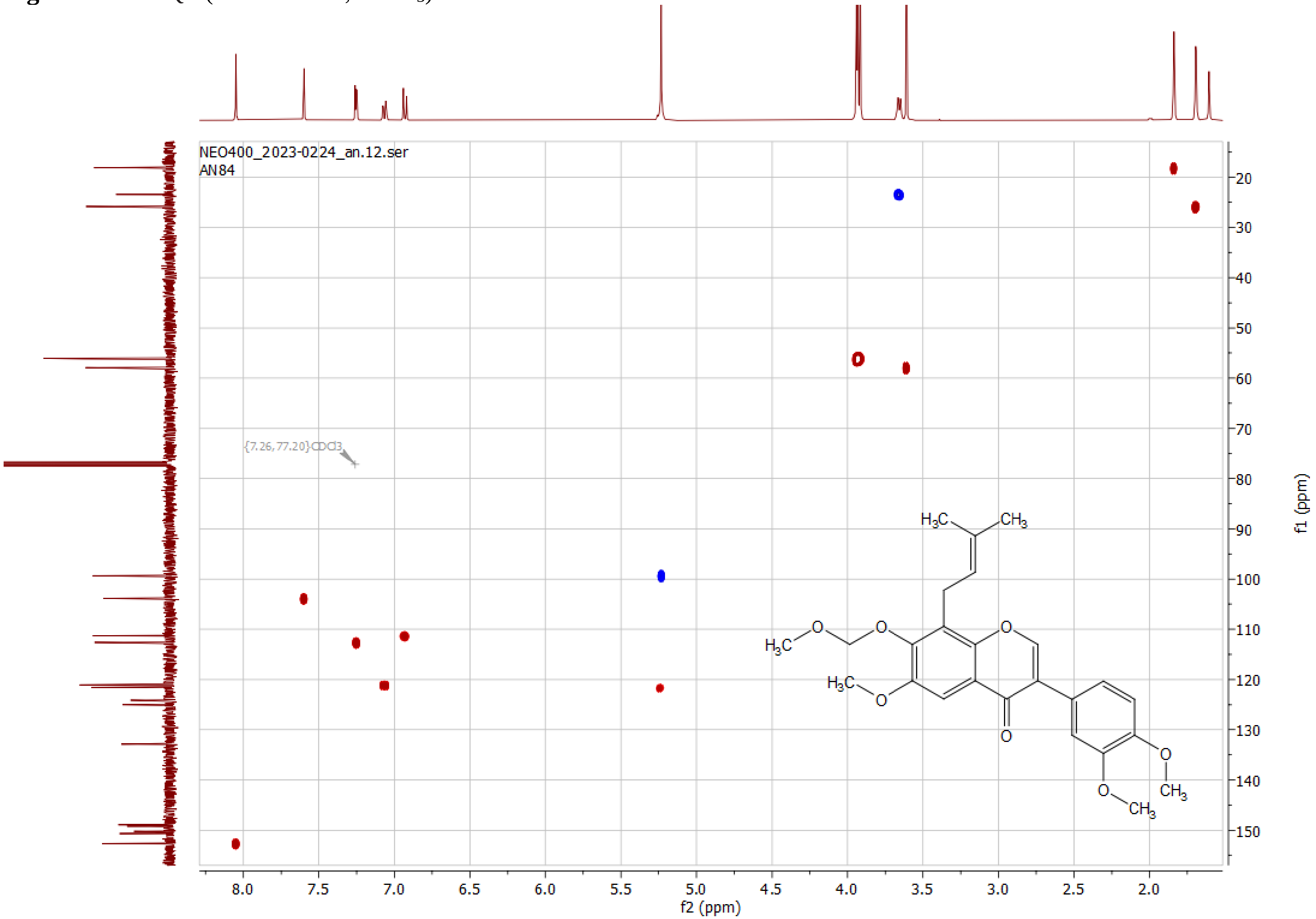

Figure S45: HMBC (400/101 MHz, $CDCl_3$) of **20c**

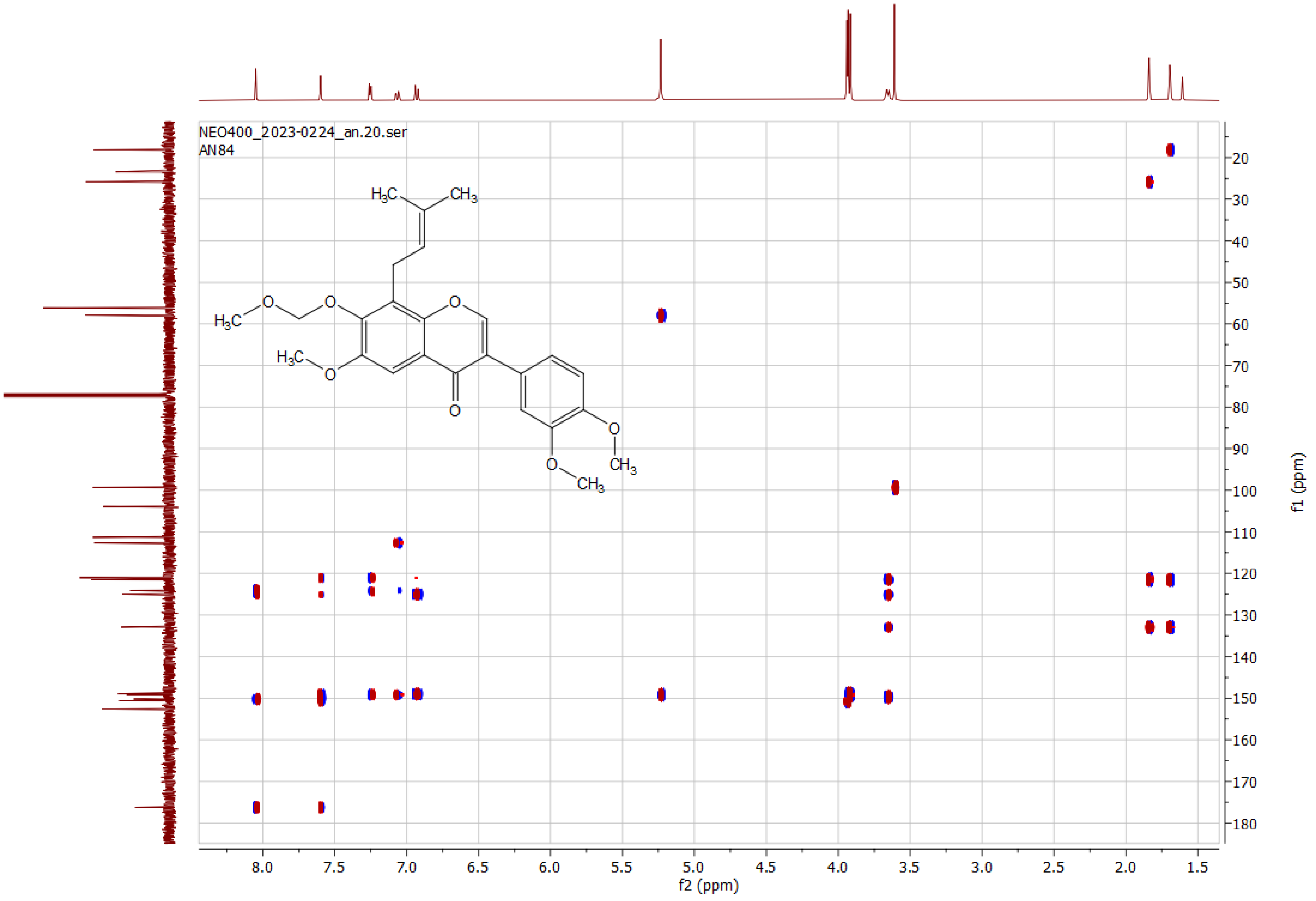

Figure S46: ^{1}H NMR (400 MHz, $CDCl_3$) of **20d**

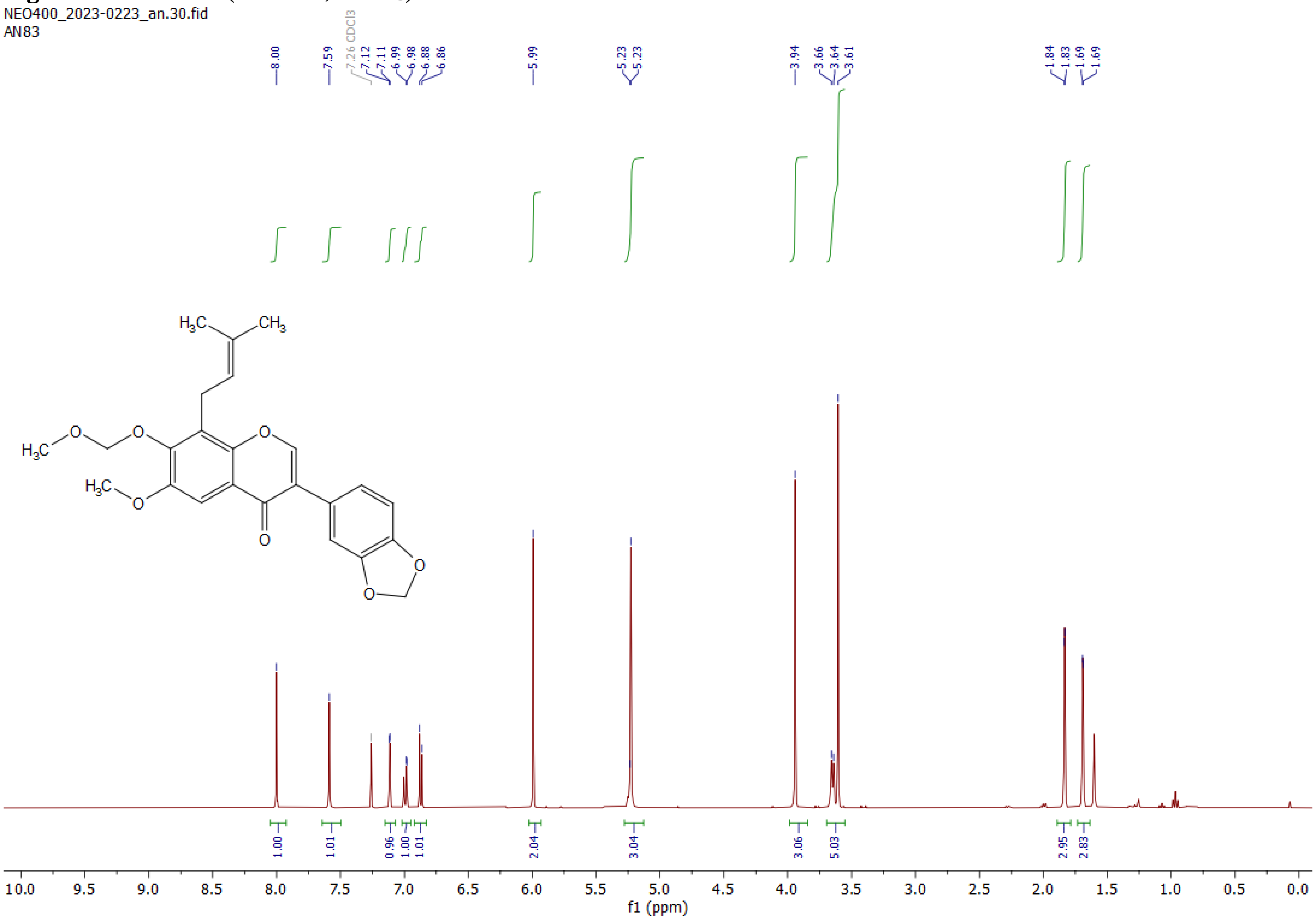

Figure S47: $^{13}C\{^1H\}$ NMR (101 MHz, $CDCl_3$) of **20d**

NEO400_2023-0223_an.31.fid
AN83

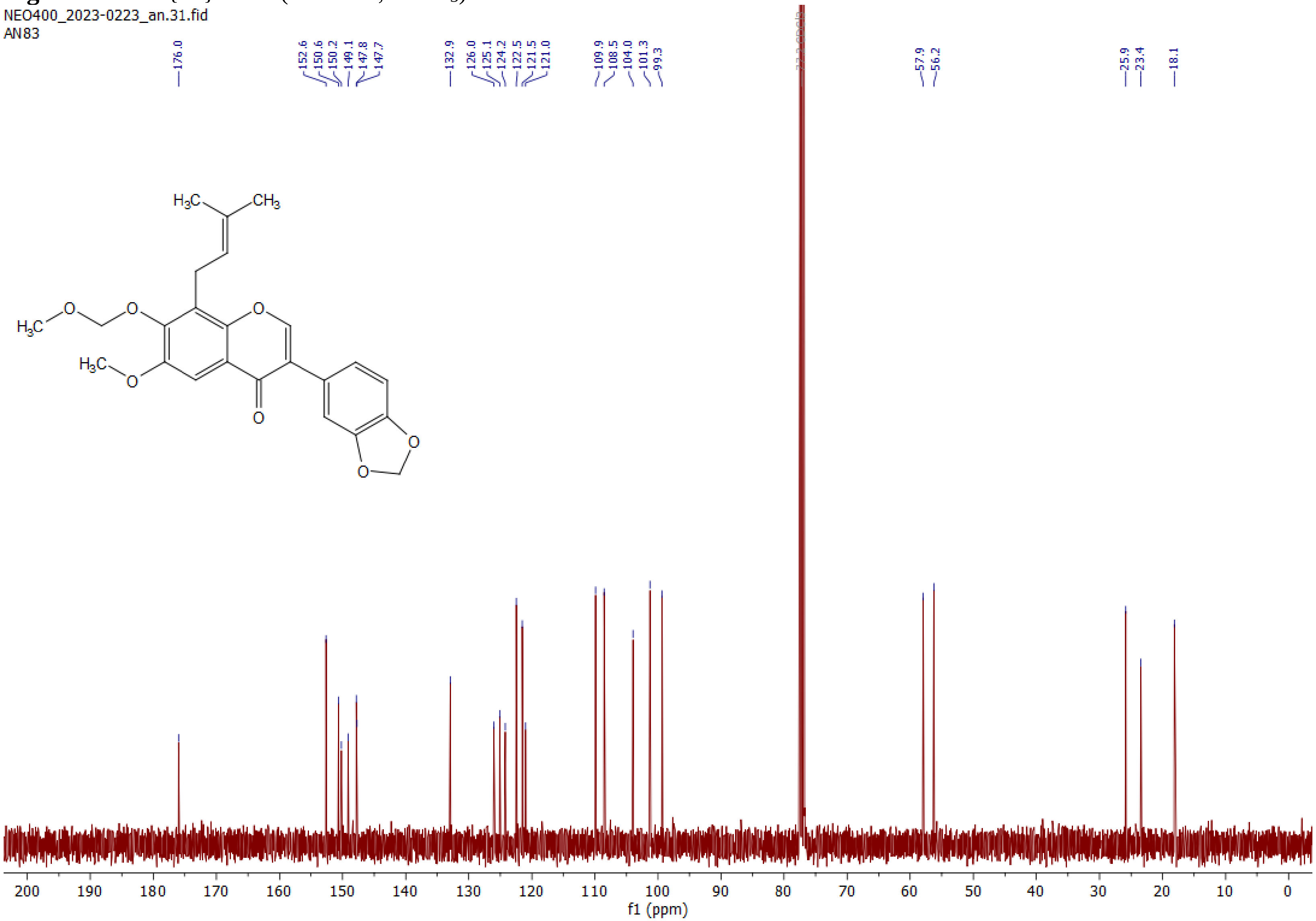

Figure S48: ^{1}H NMR (400 MHz, $CDCl_3$) of **20e**

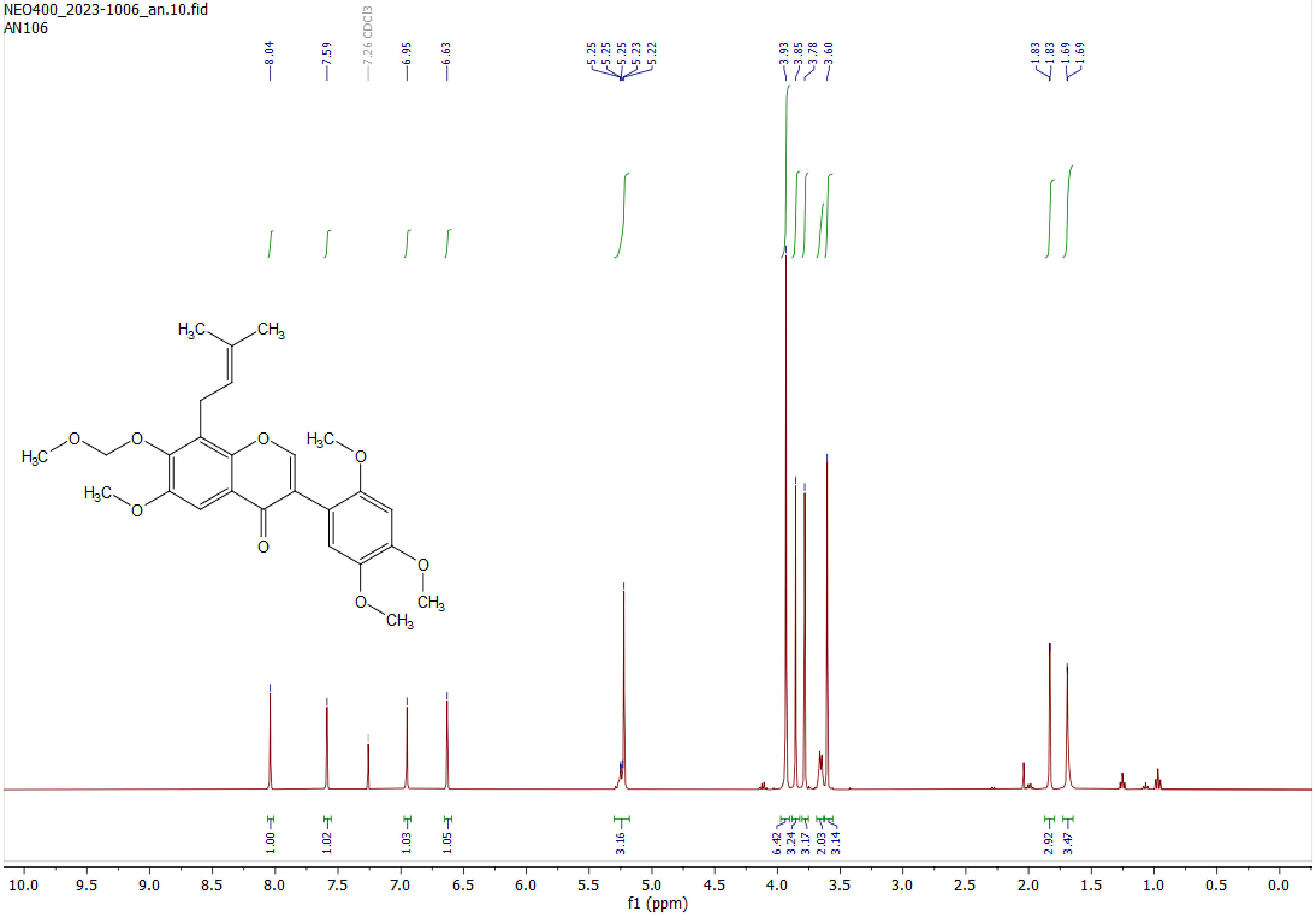

Figure S49: $^{13}C\{^{1}H\}$ NMR (101 MHz, $CDCl_3$) of **20e**

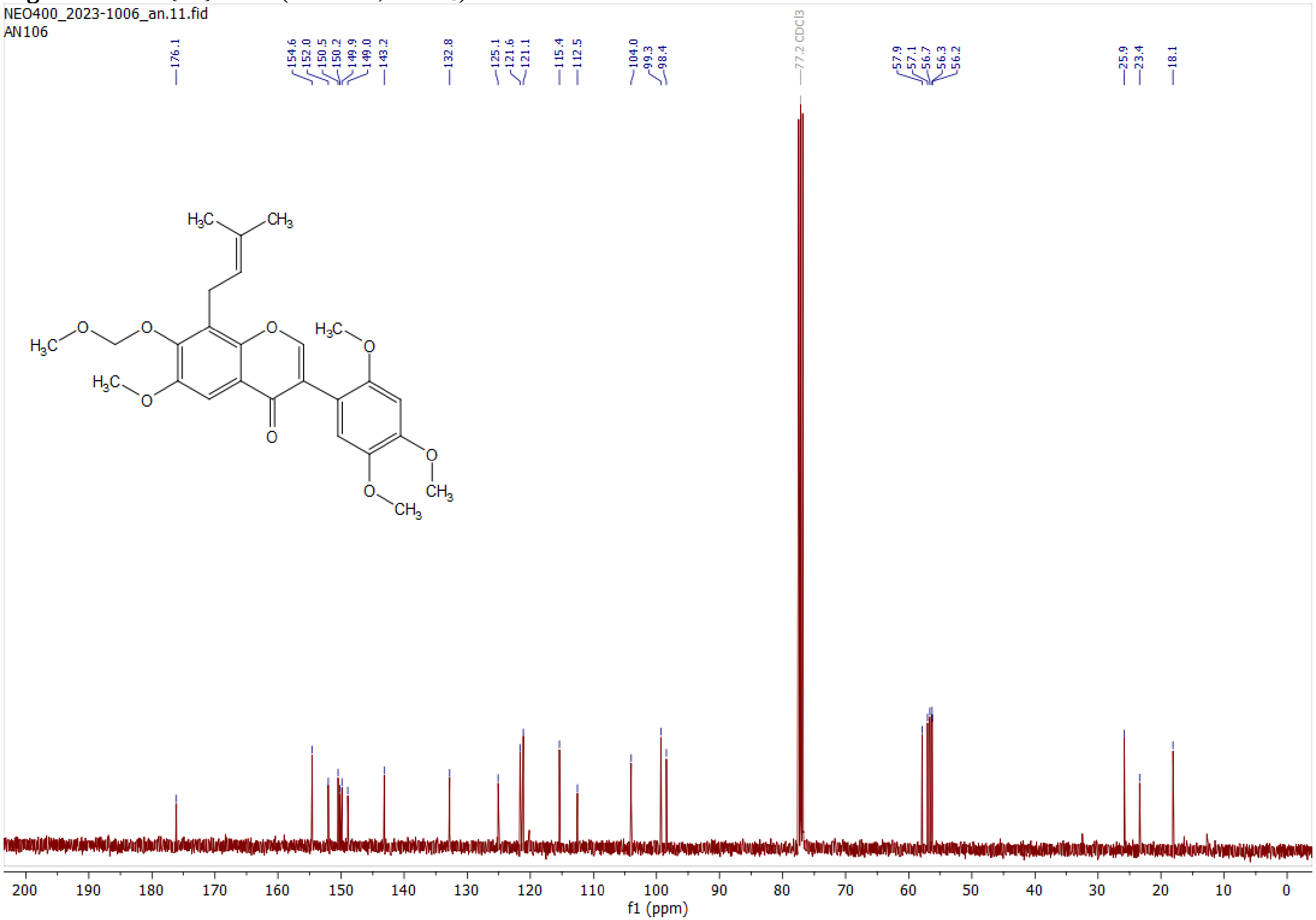

Figure S50: ^{1}H NMR (400 MHz, $CDCl_3$) of **20f**

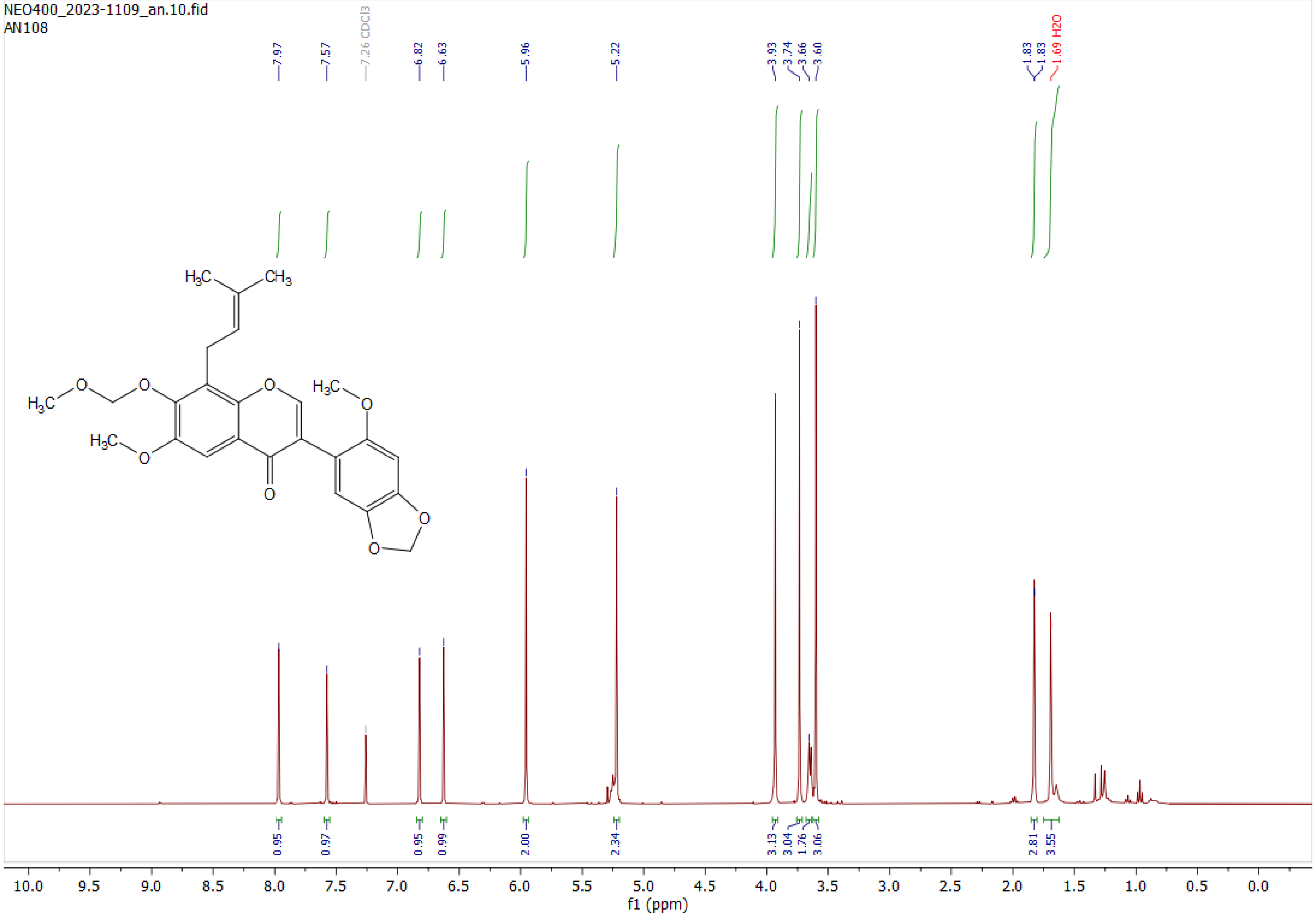

Figure S51: $^{13}C\{^{1}H\}$ NMR (101 MHz, $CDCl_3$) of **20f**

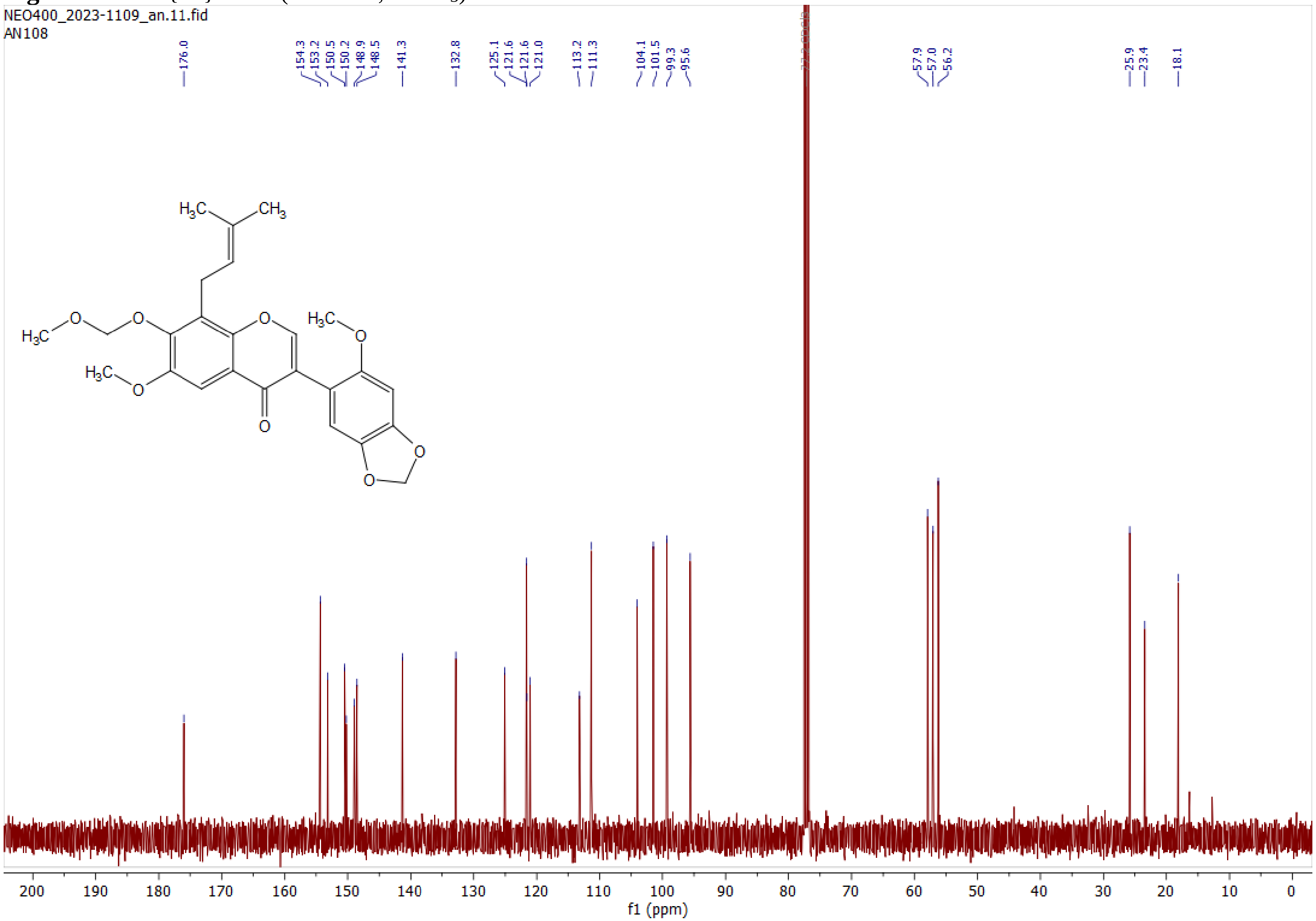

Figure S52: ^{1}H NMR (400 MHz, $CDCl_3$) of **21a**

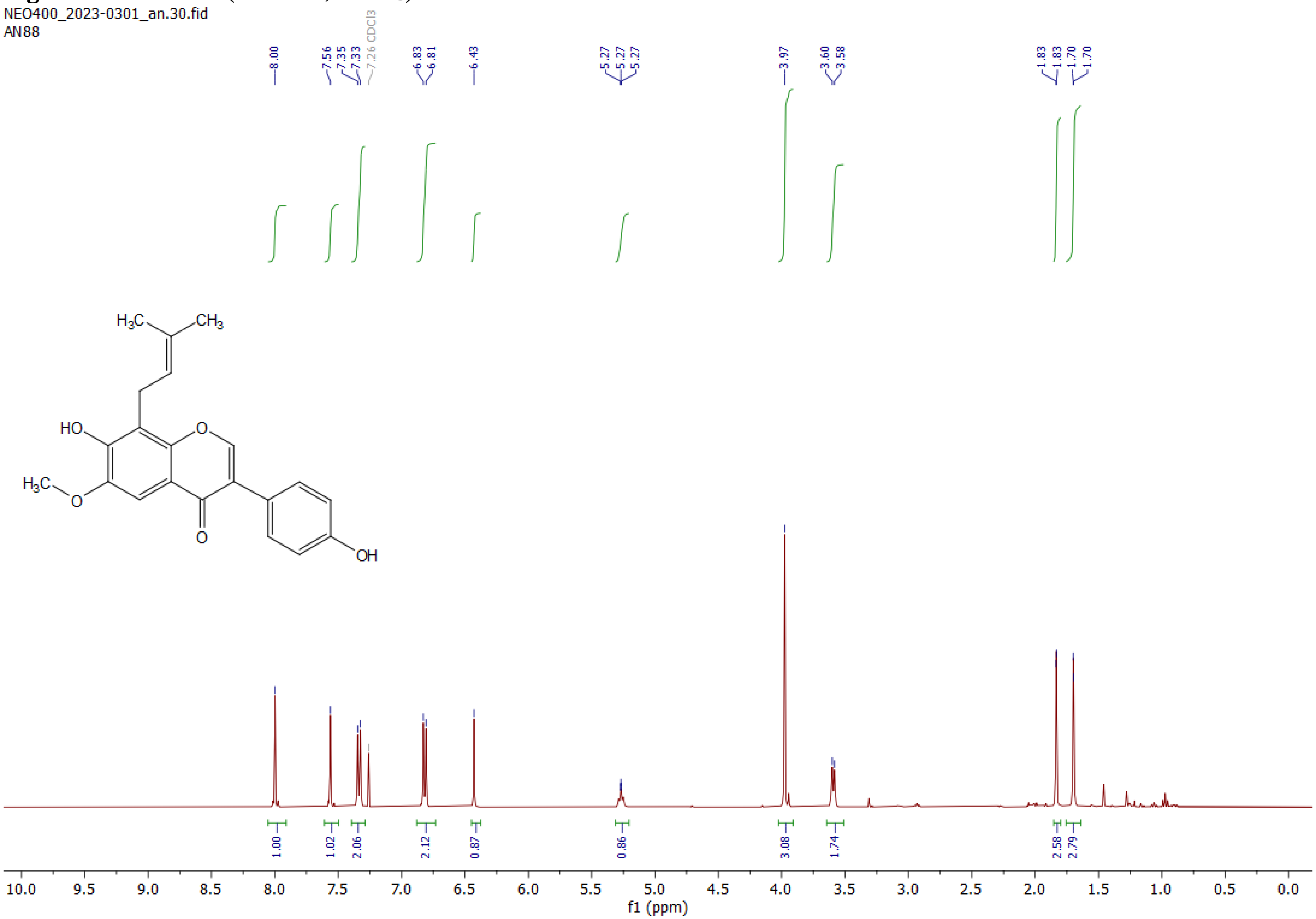

Figure S53: $^{13}C\{^{1}H\}$ NMR (101 MHz, $CDCl_3$) of **21a**

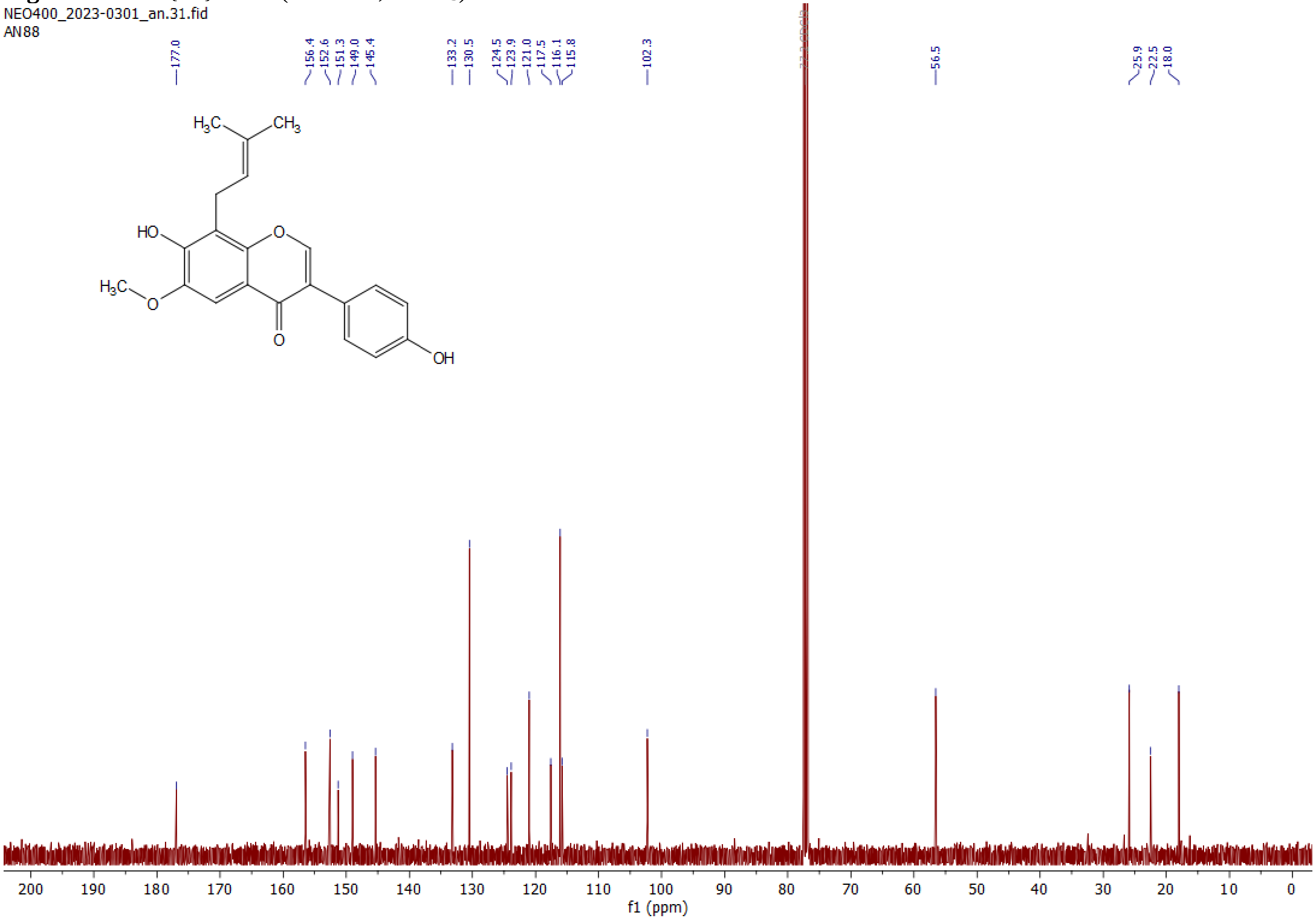

Figure S54: ^{1}H NMR (400 MHz, $CDCl_3$) of **21b**

NEO400_2023-0208_an.10.fid
AN81

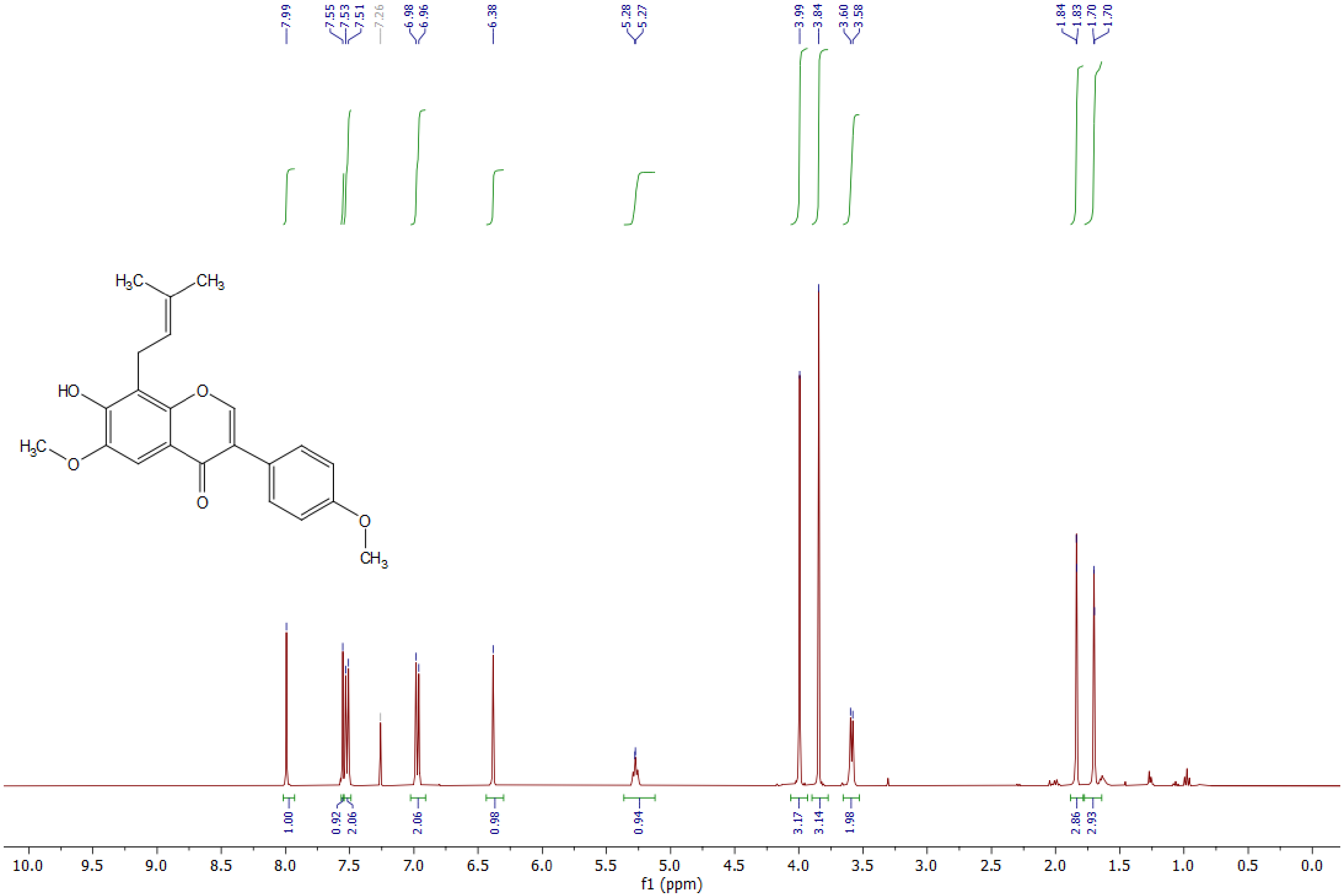

Figure S55: $^{13}C\{^{1}H\}$ NMR (101 MHz, $CDCl_3$) of **21b**

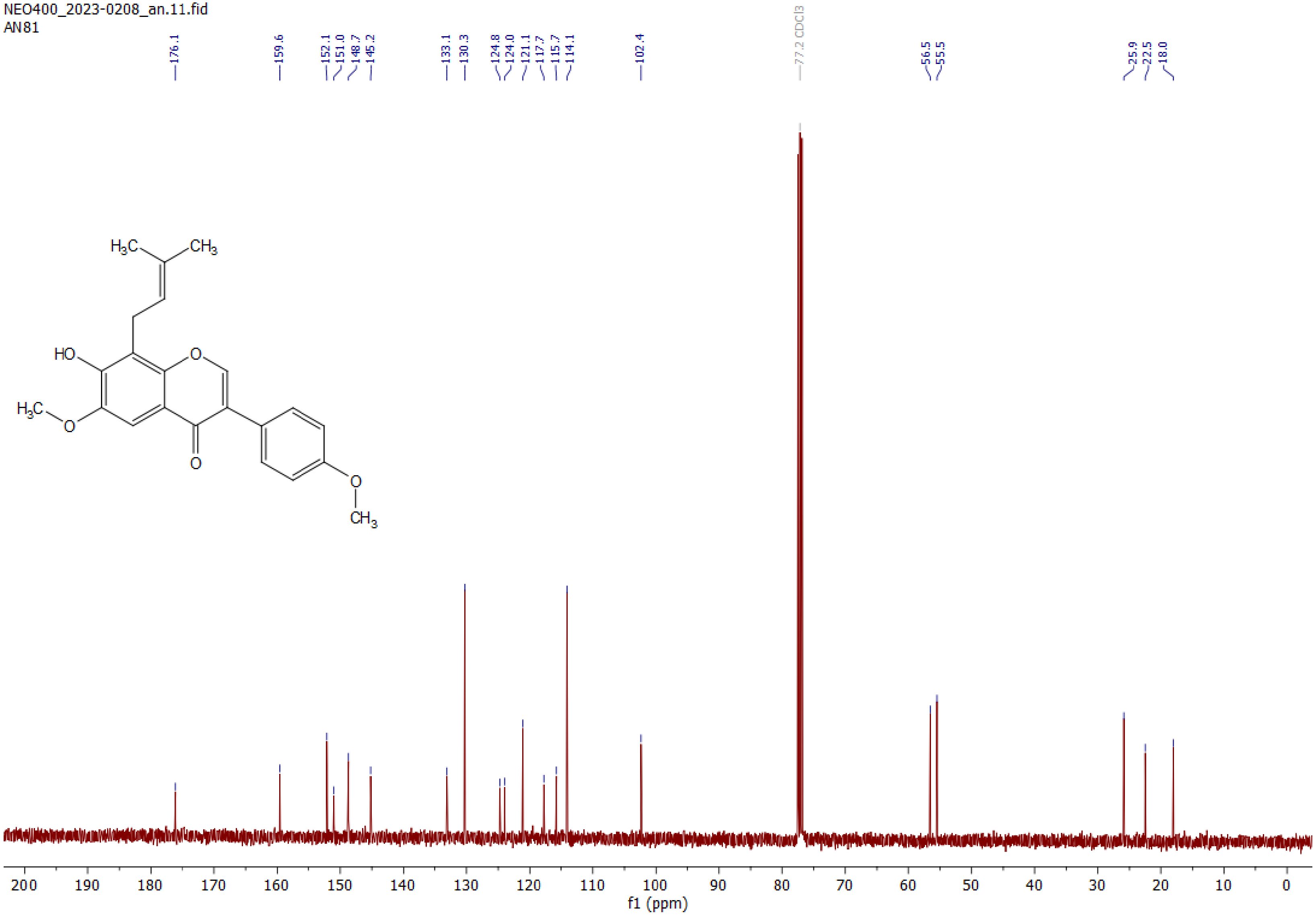

Figure S56: ^{1}H NMR (400 MHz, $CDCl_3$) of **21c**

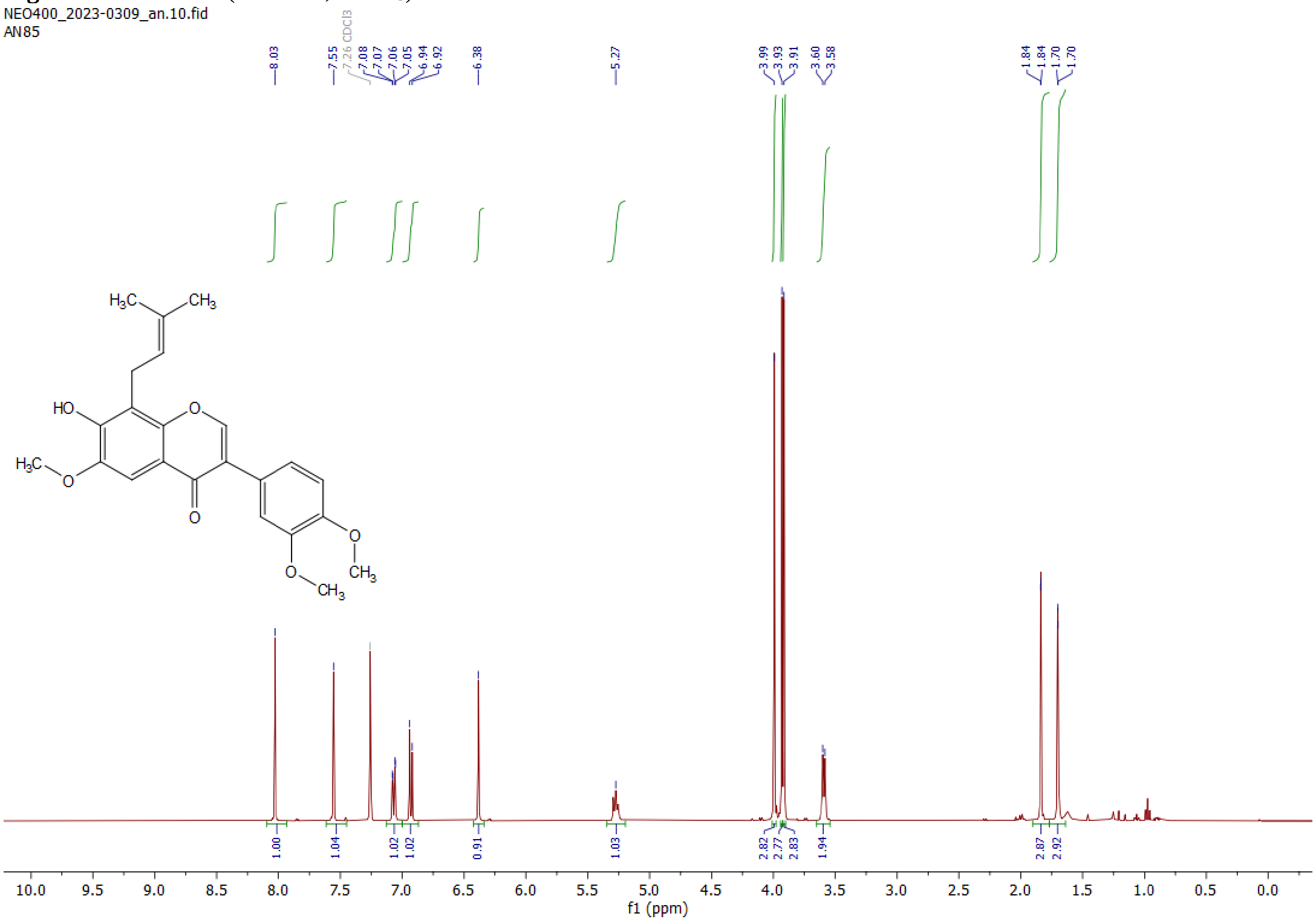

Figure S57: $^{13}C\{^{1}H\}$ NMR (101 MHz, $CDCl_3$) of **21c**

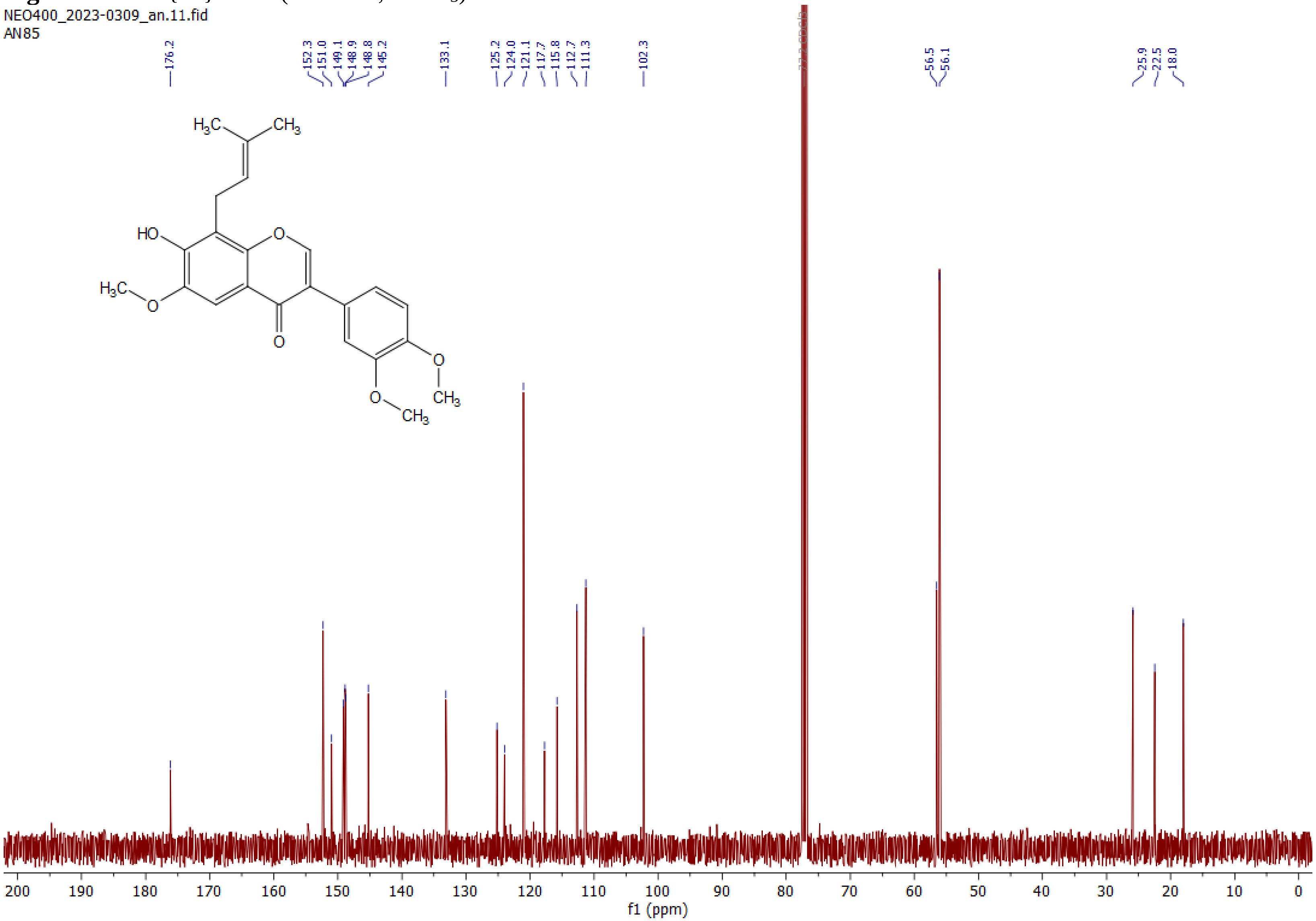

Figure S58: ^{1}H NMR (400 MHz, $CDCl_3$) of **21d**

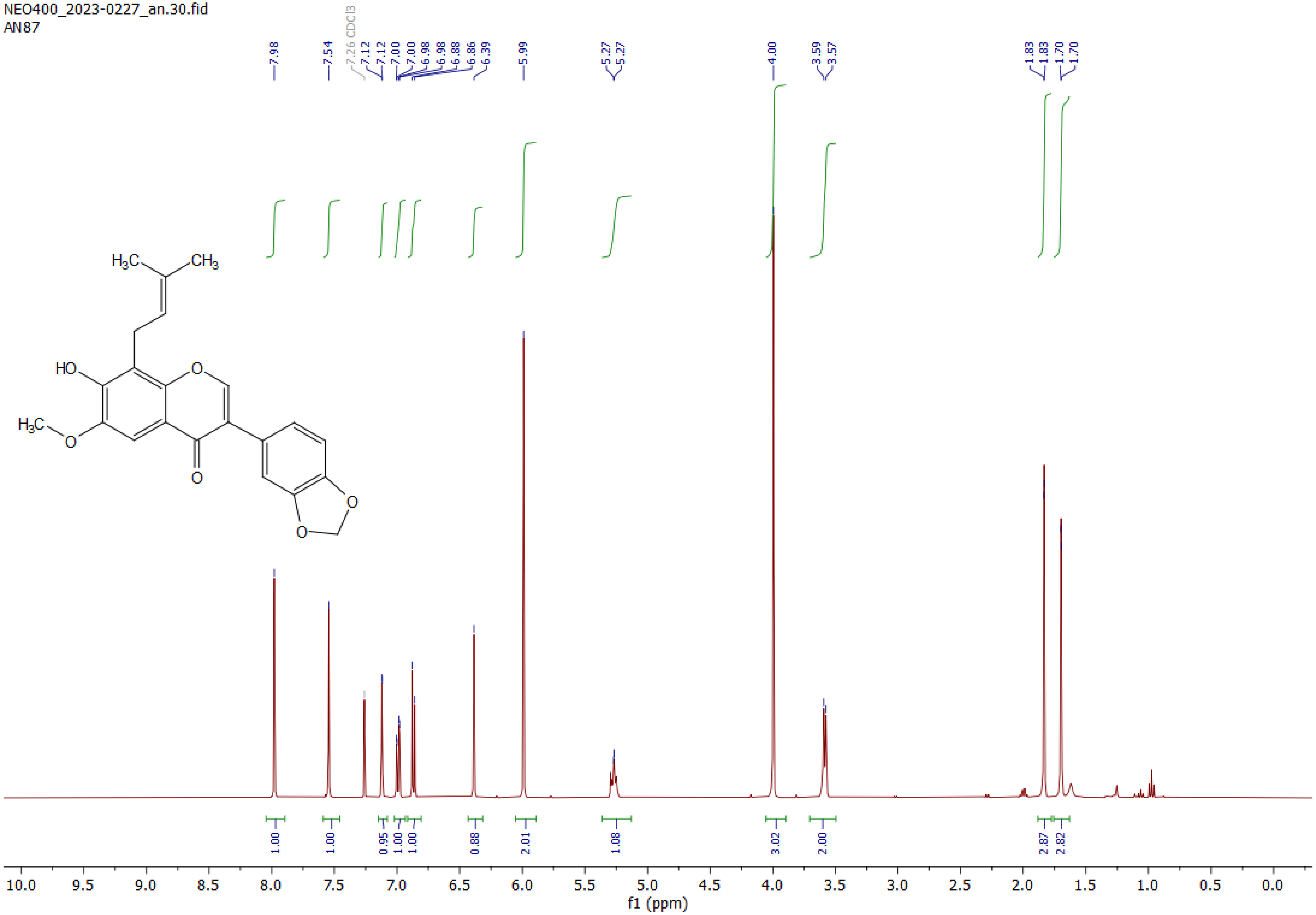

Figure S59: $^{13}C\{^1H\}$ NMR (101 MHz, $CDCl_3$) of **21d**

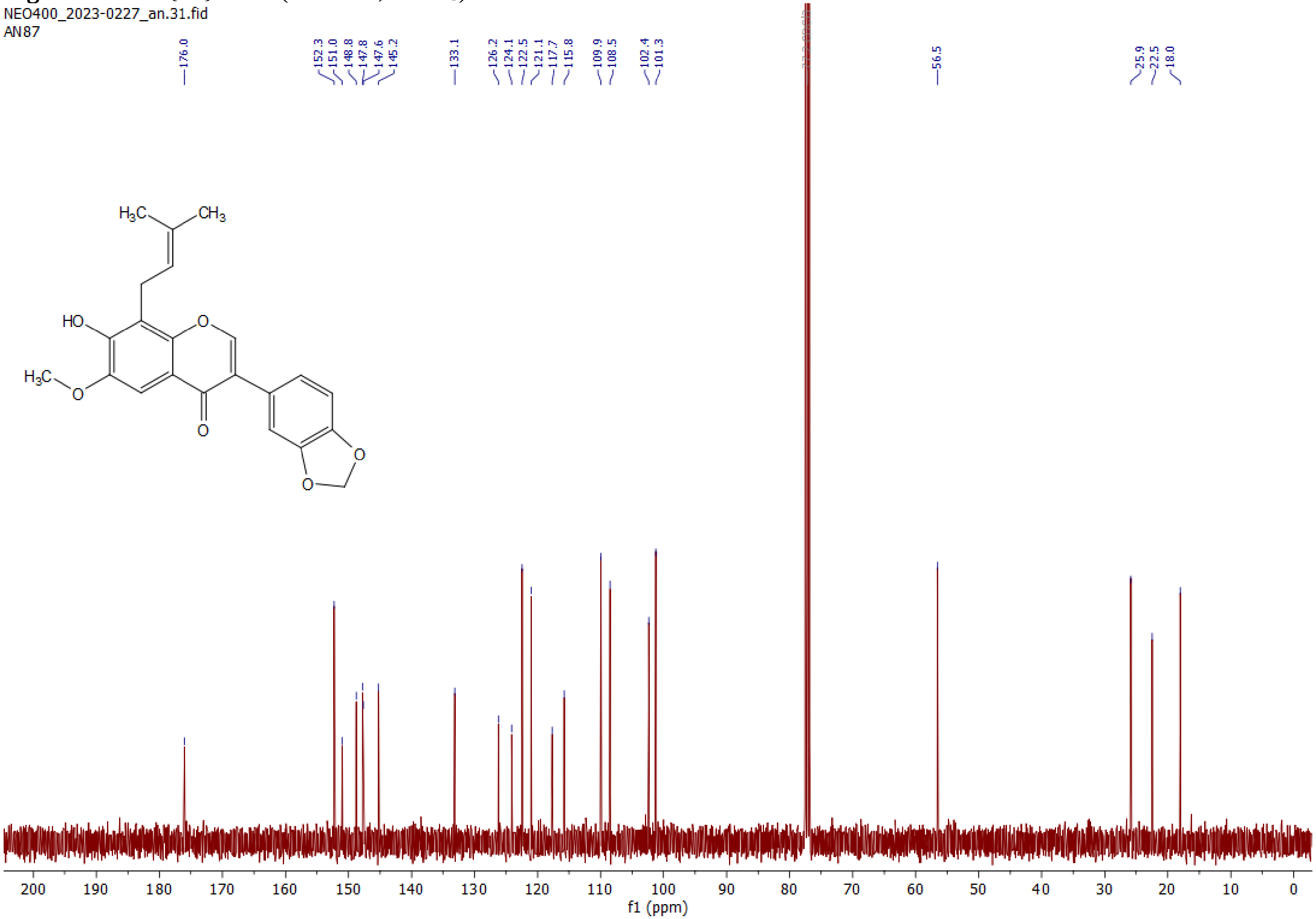

Figure S60: H,H-COSY (400 MHz, $CDCl_3$) of **21d**

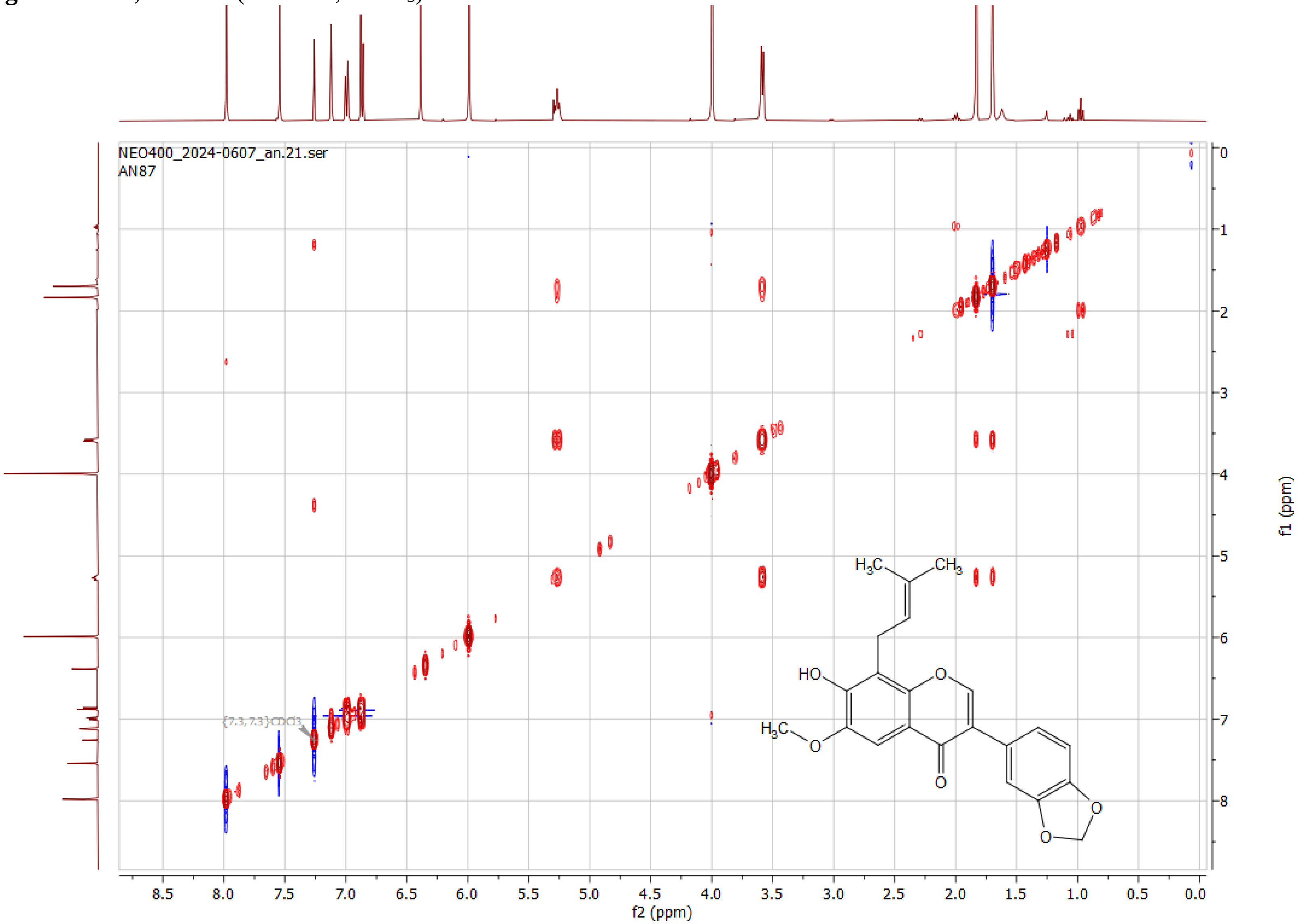

Figure S61: HSQC (400/101 MHz, $CDCl_3$) of **21d**

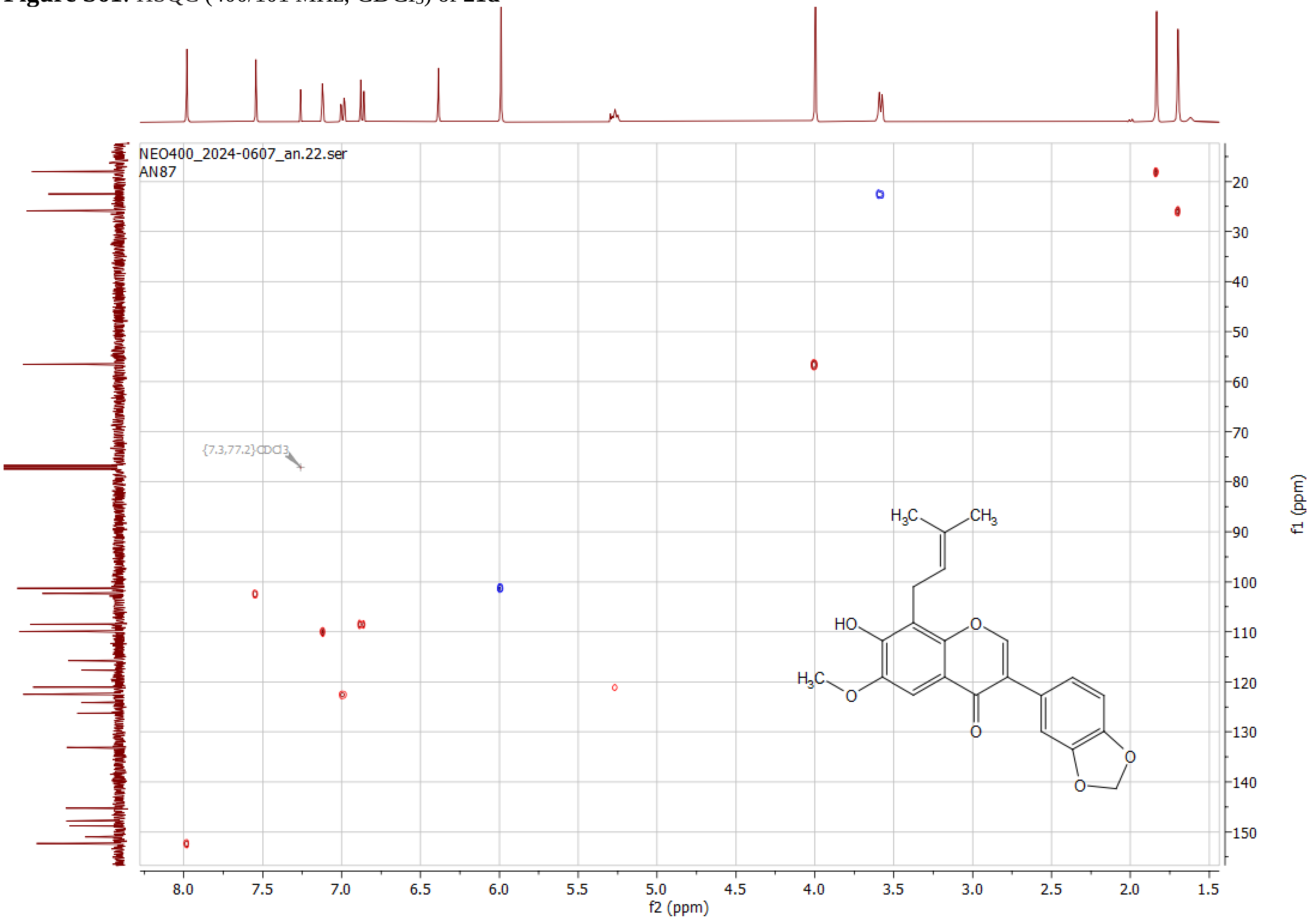

Figure S62: HMBC (400/101 MHz, $CDCl_3$) of **21d**

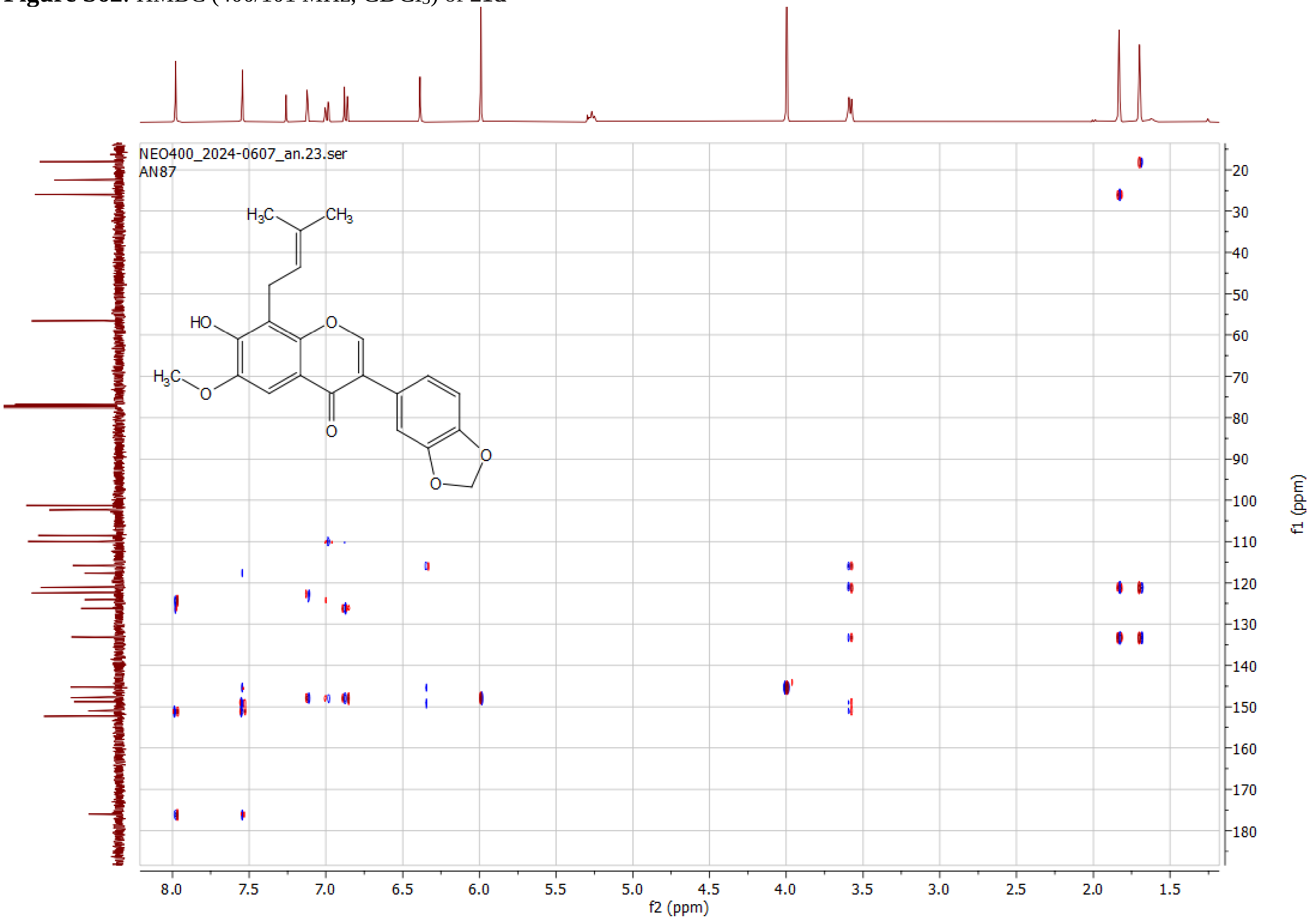

Figure S63: ^{1}H NMR (400 MHz, $CDCl_3$) of **21e**

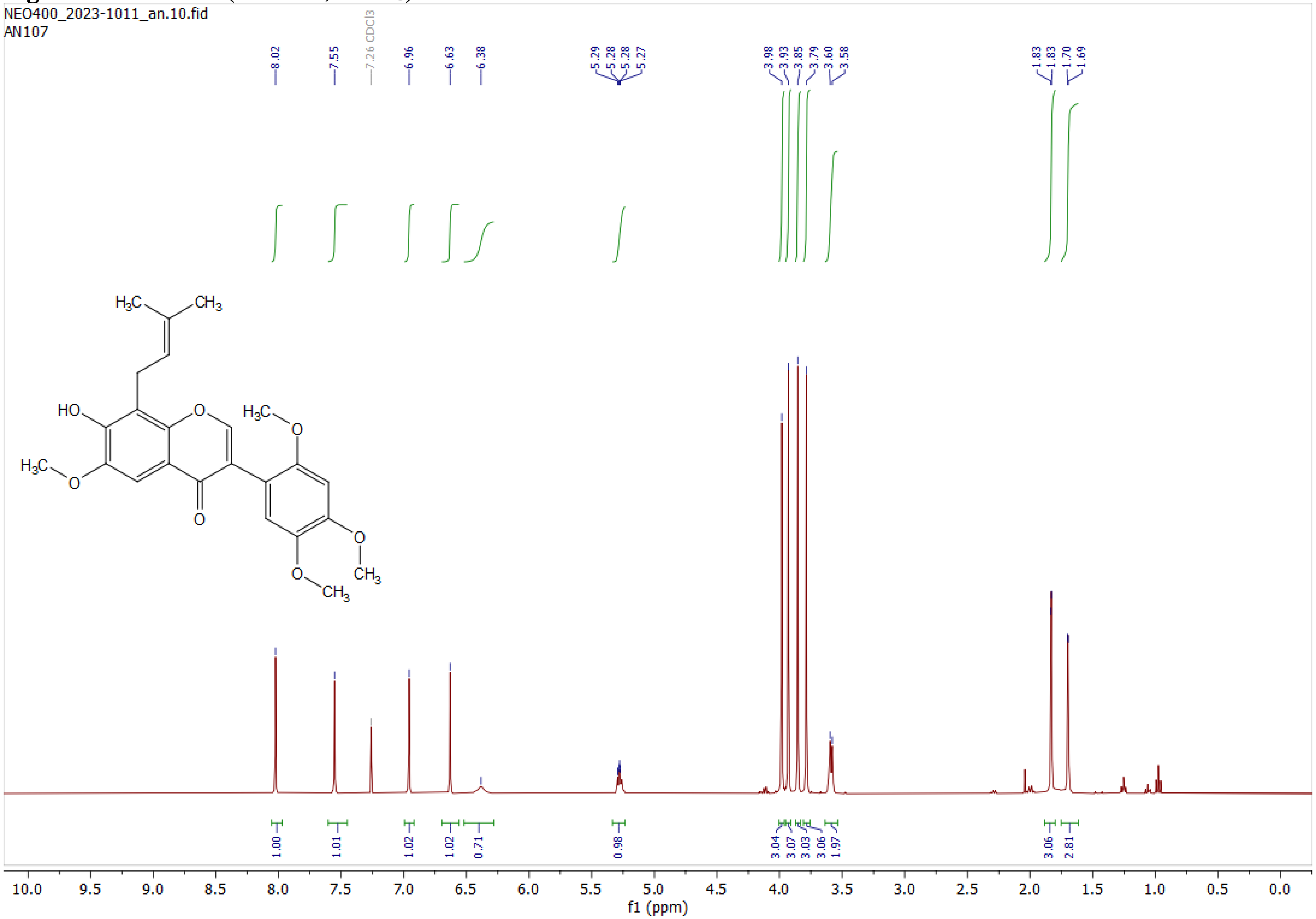

Figure S64: $^{13}C\{^{1}H\}$ NMR (101 MHz, $CDCl_3$) of **21e**

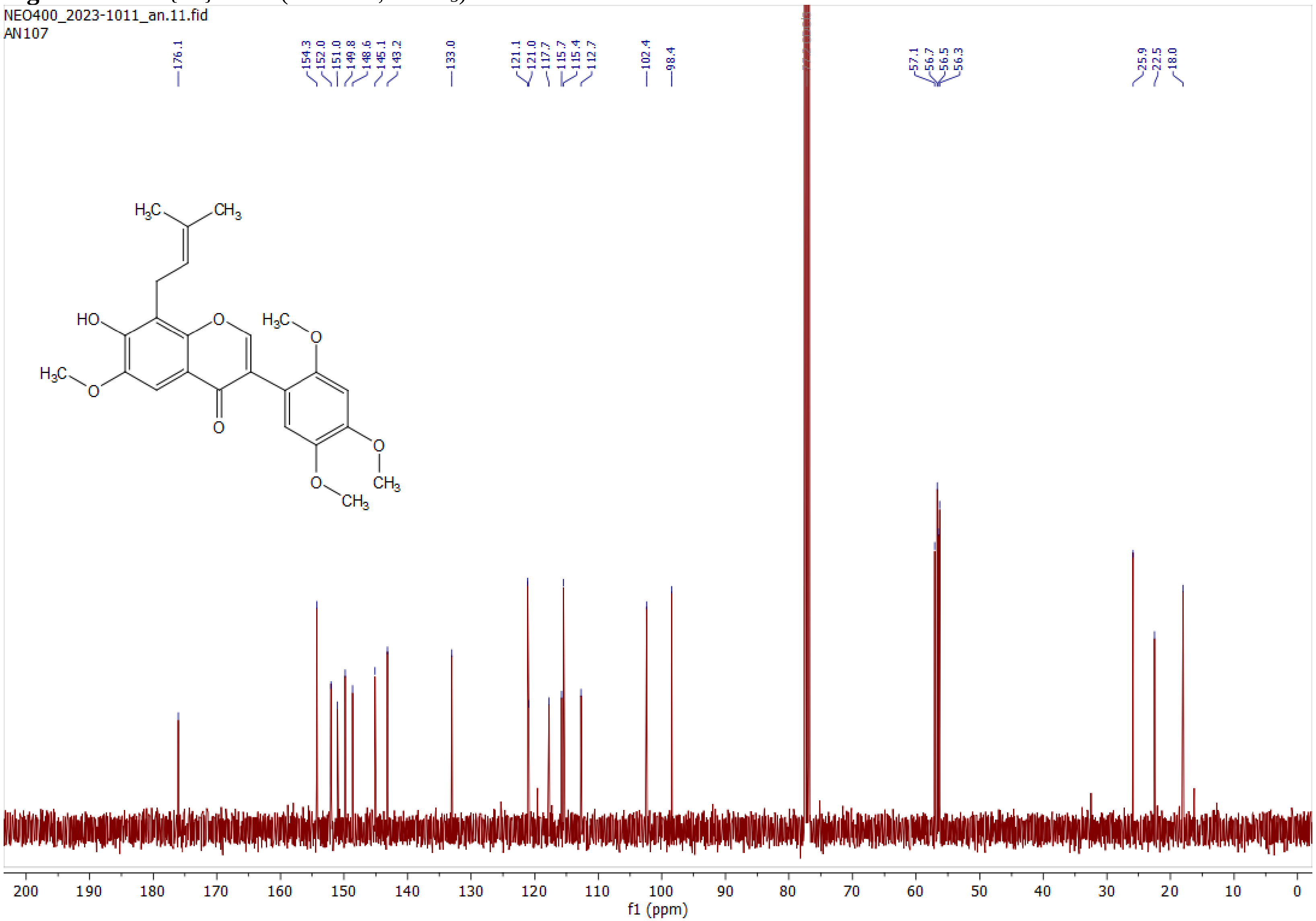

Figure S65: ^{1}H NMR (400 MHz, $CDCl_3$) of **21f**

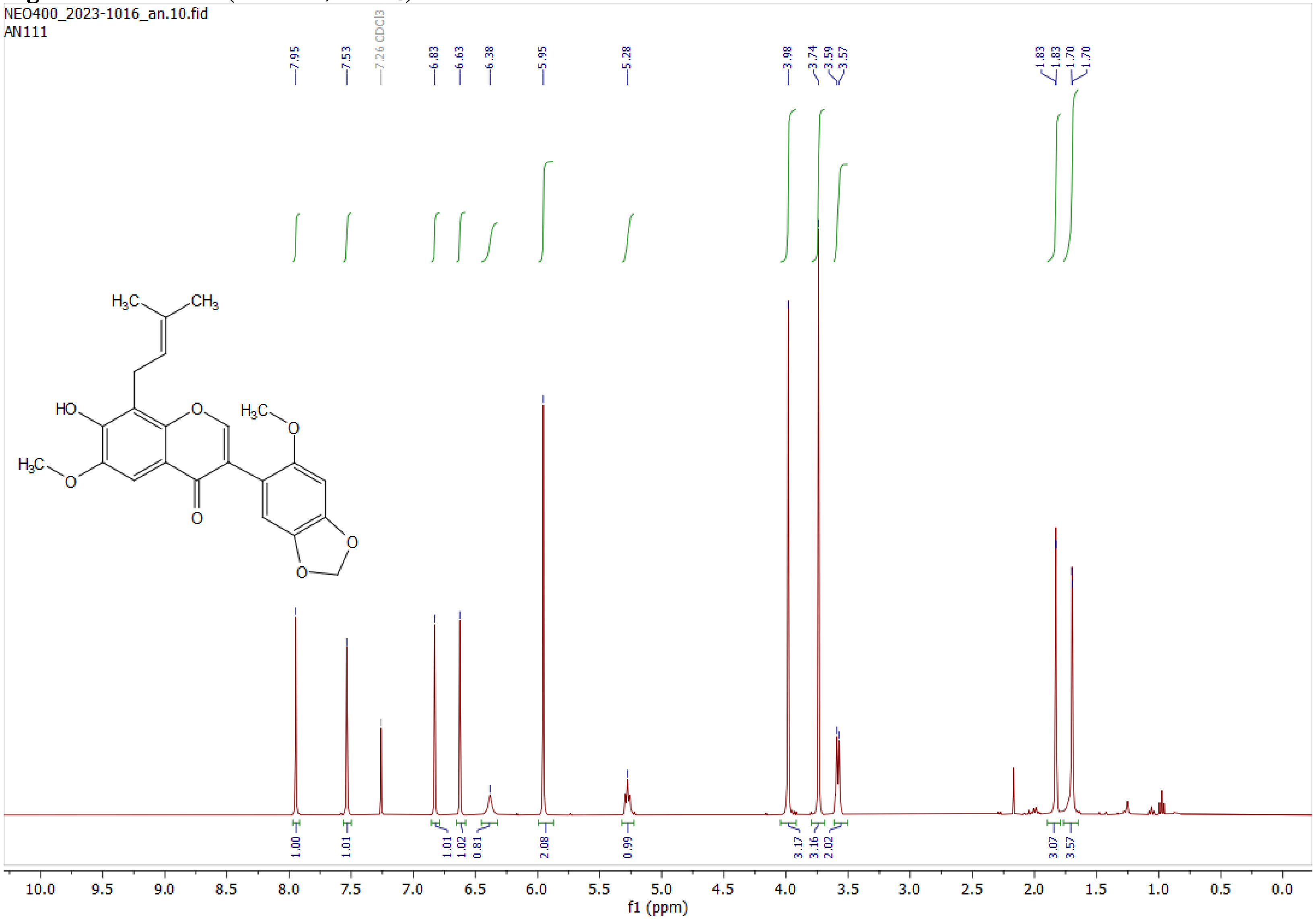

Figure S66: $^{13}C\{^1H\}$ NMR (101 MHz, $CDCl_3$) of **21f**

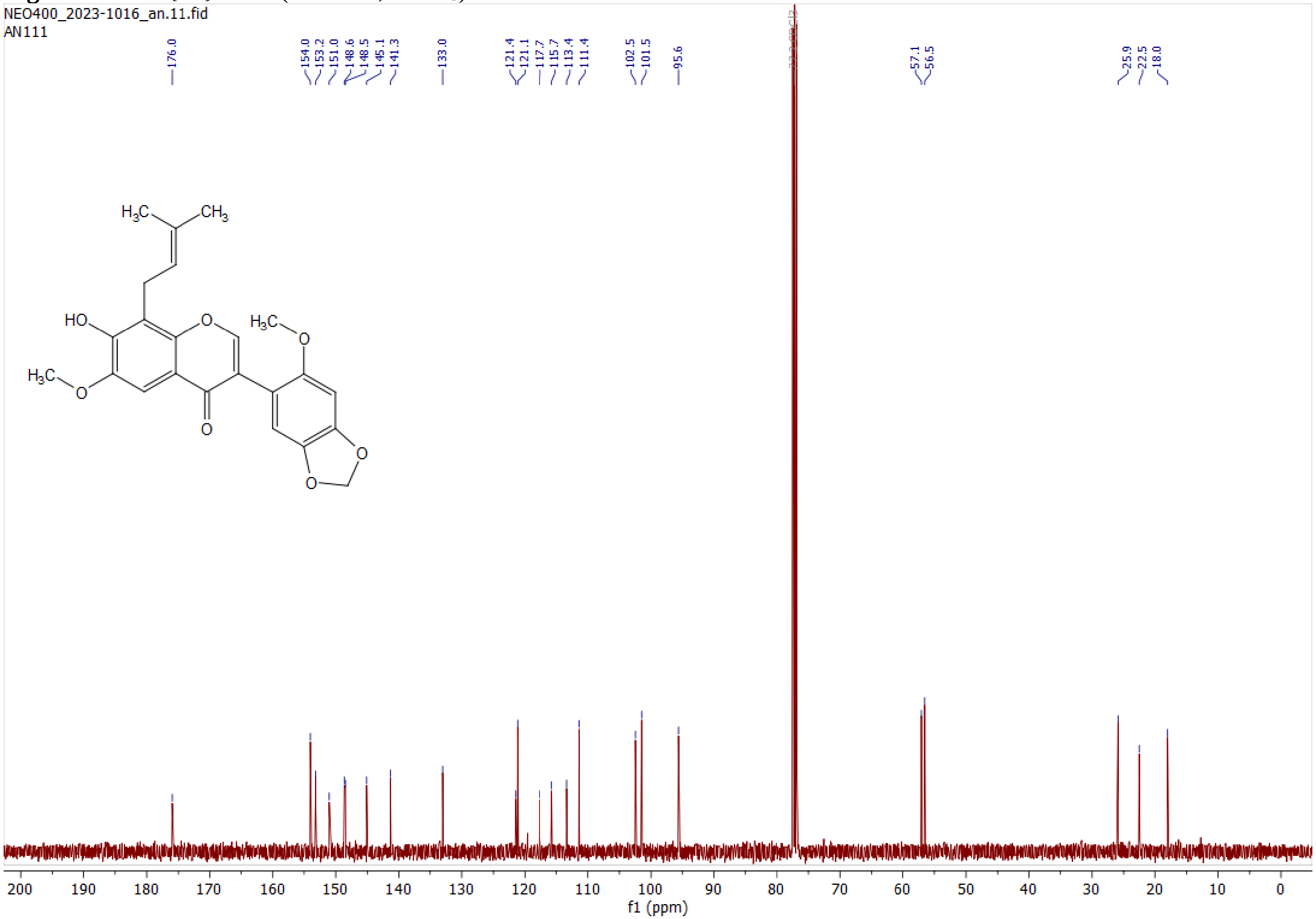

Figure S67: ^{1}H NMR (400 MHz, $CDCl_3$) of **22b**

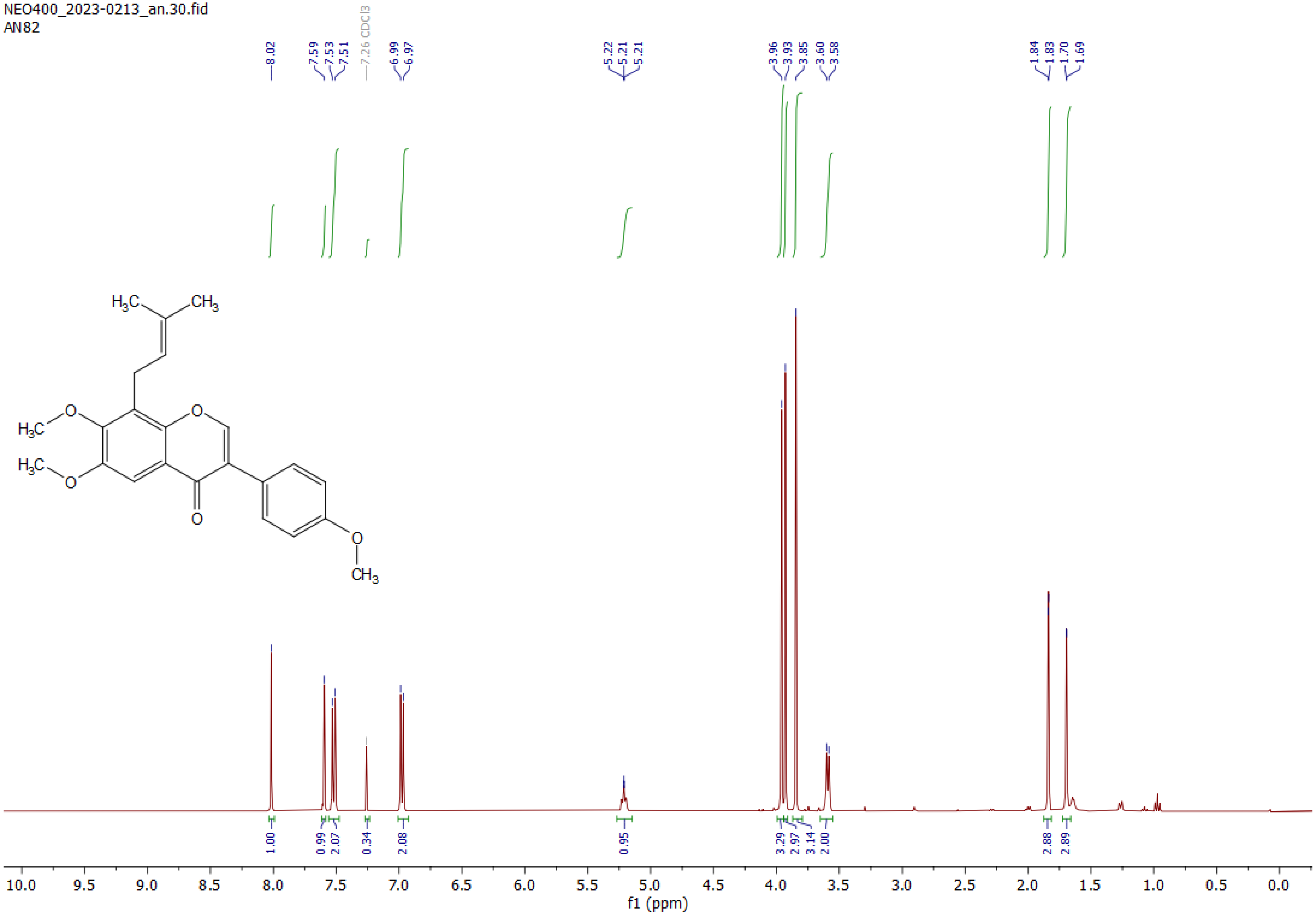

Figure S68: $^{13}C\{^1H\}$ NMR (101 MHz, $CDCl_3$) of **22b**

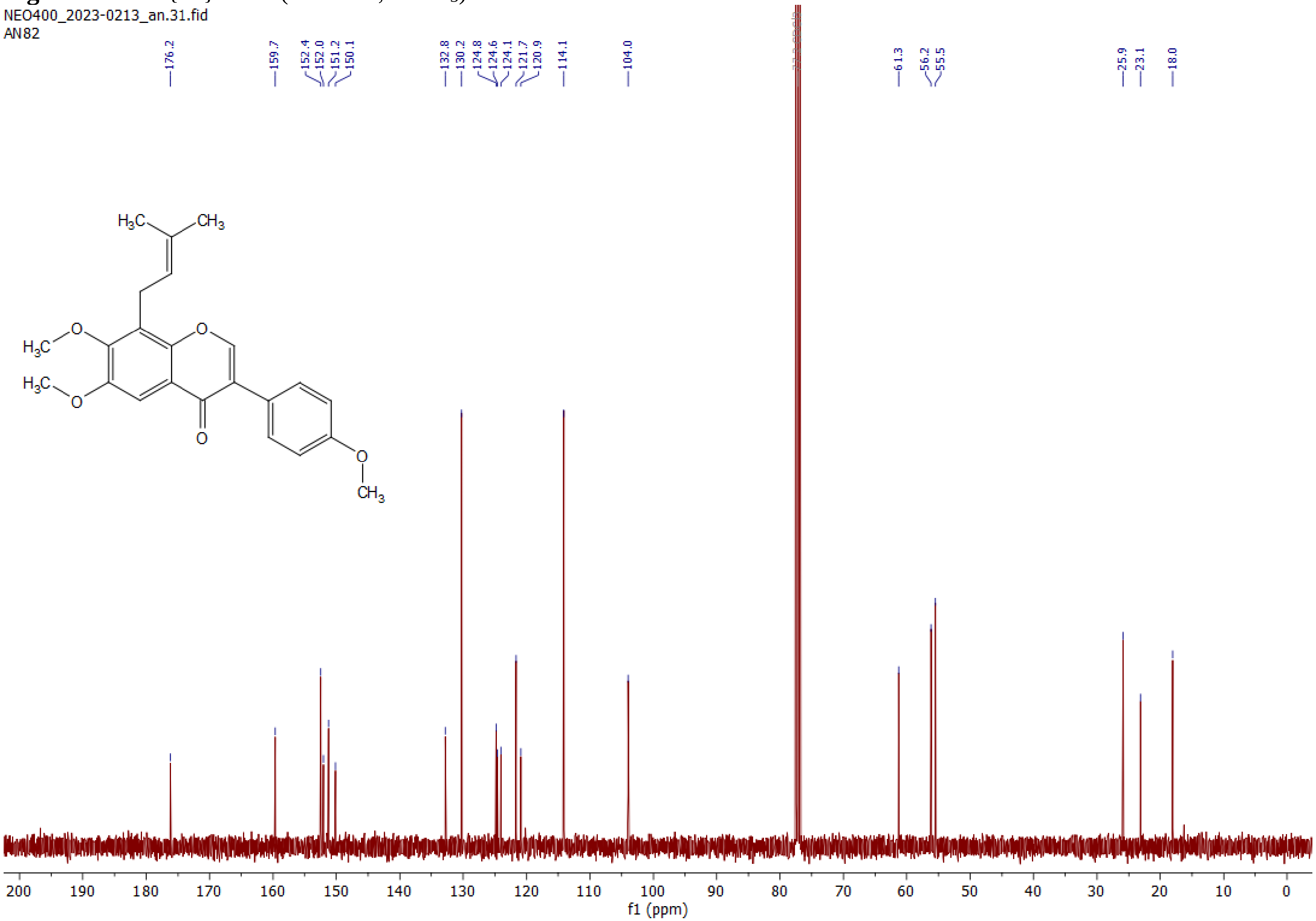

Figure S69: ^{1}H NMR (400 MHz, $CDCl_3$) of **22c**

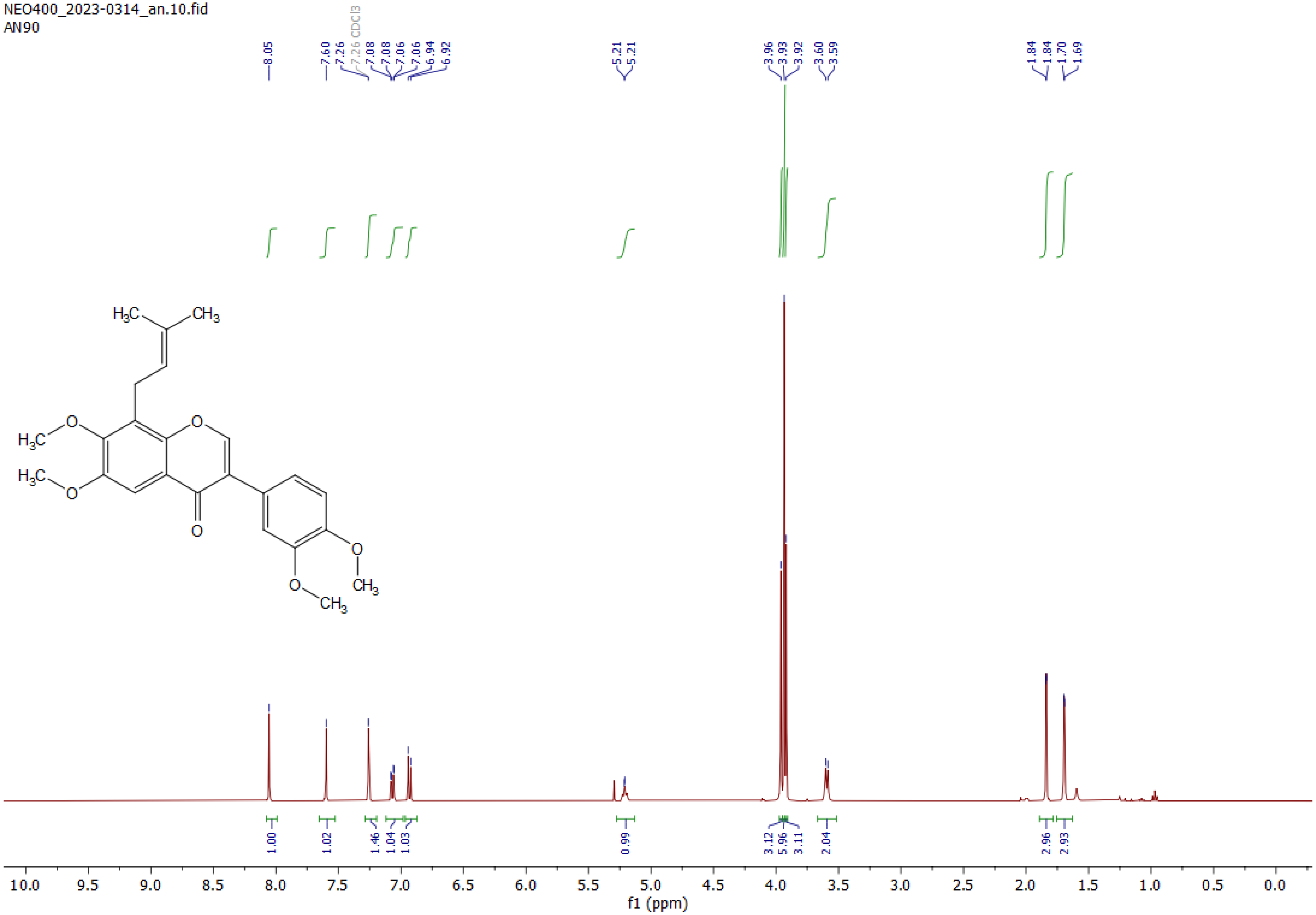

Figure S70: $^{13}C\{^{1}H\}$ NMR (101 MHz, $CDCl_3$) of **22c**

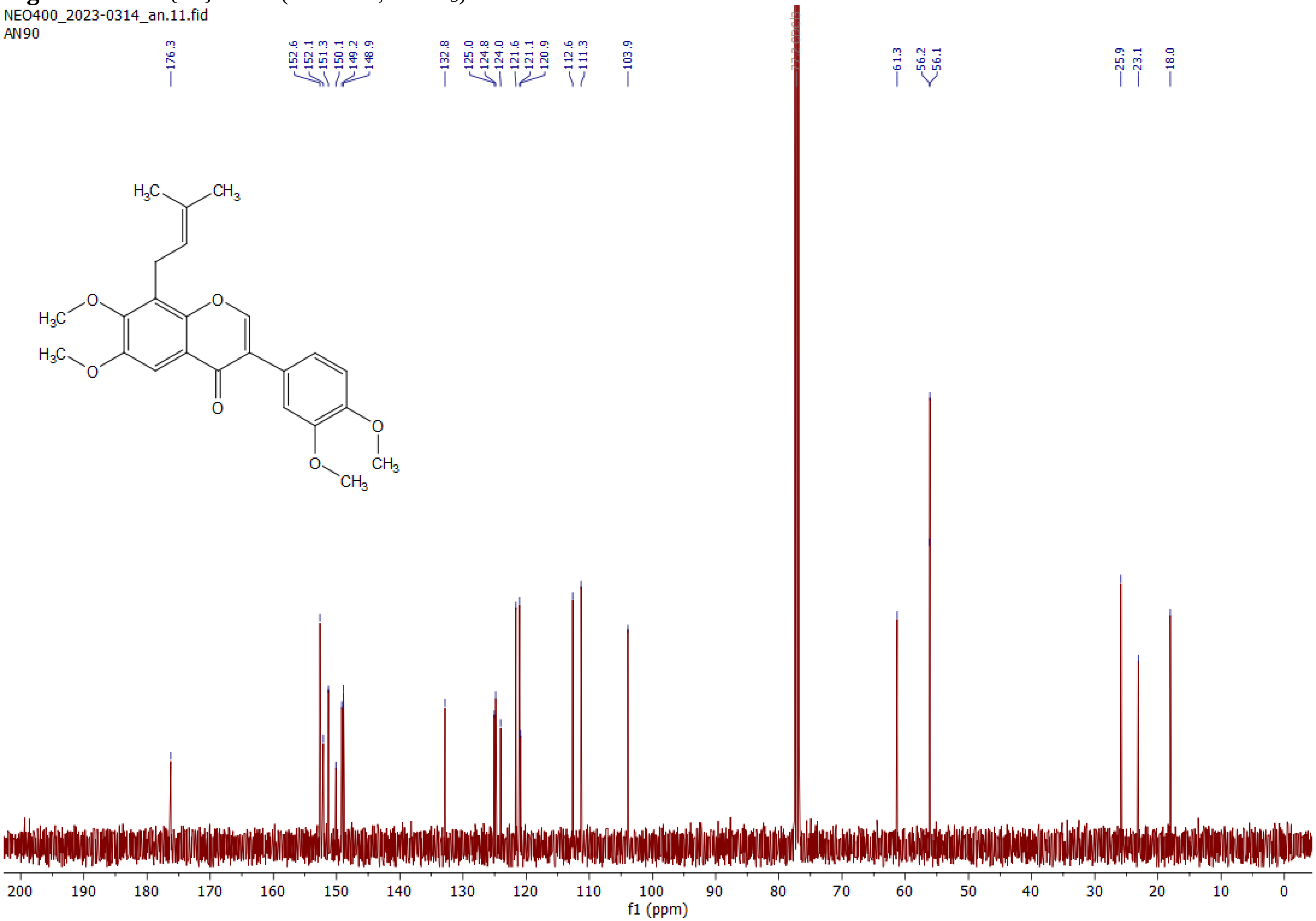

Figure S71: ^{1}H NMR (400 MHz, $CDCl_3$) of **22d**

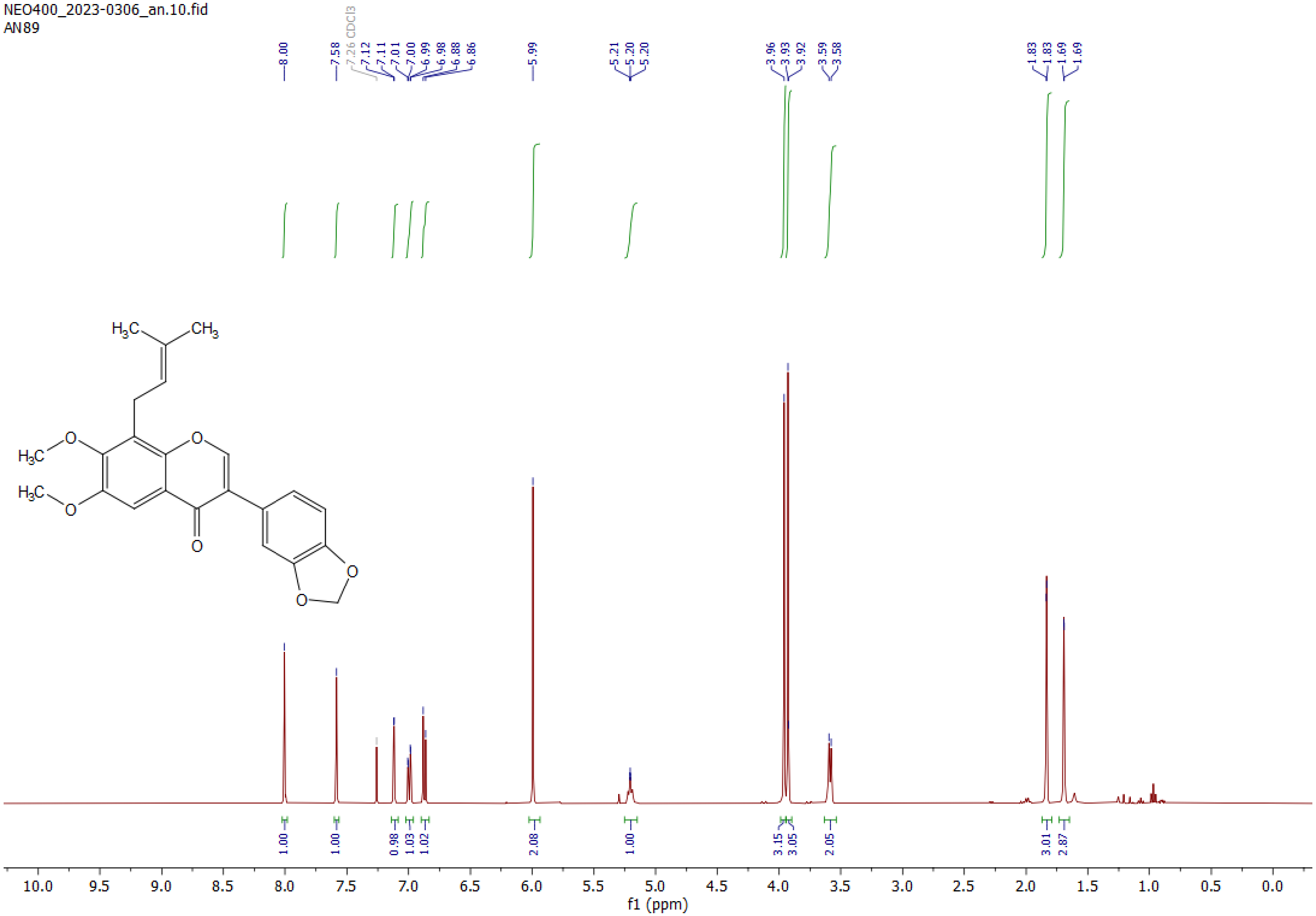

Figure S72: $^{13}C\{^{1}H\}$ NMR (101 MHz, $CDCl_3$) of **22d**

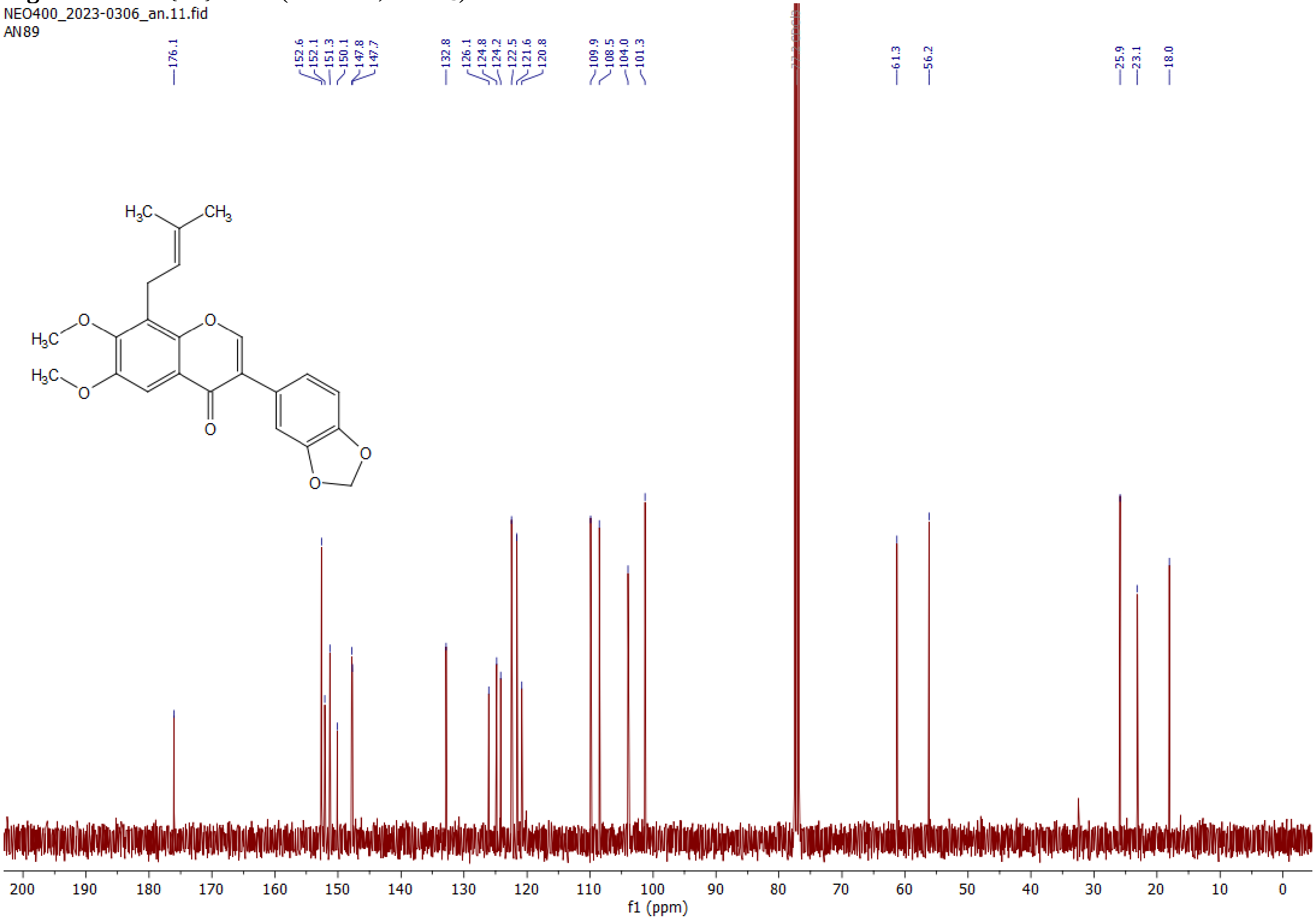

Figure S73: ^{1}H NMR (400 MHz, $CDCl_3$) of **22e**

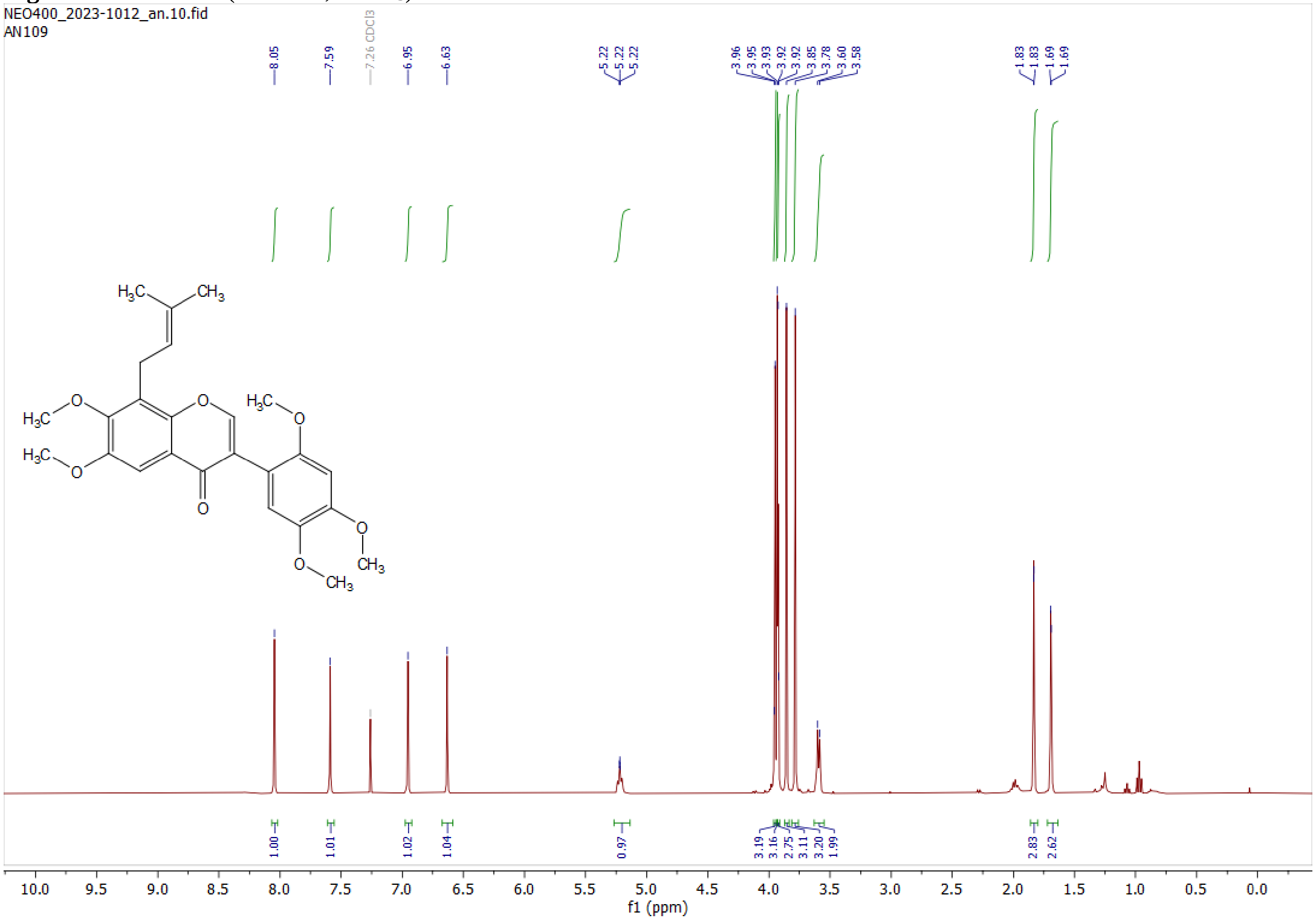

Figure S74: $^{13}C\{^{1}H\}$ NMR (101 MHz, $CDCl_3$) of **22e**

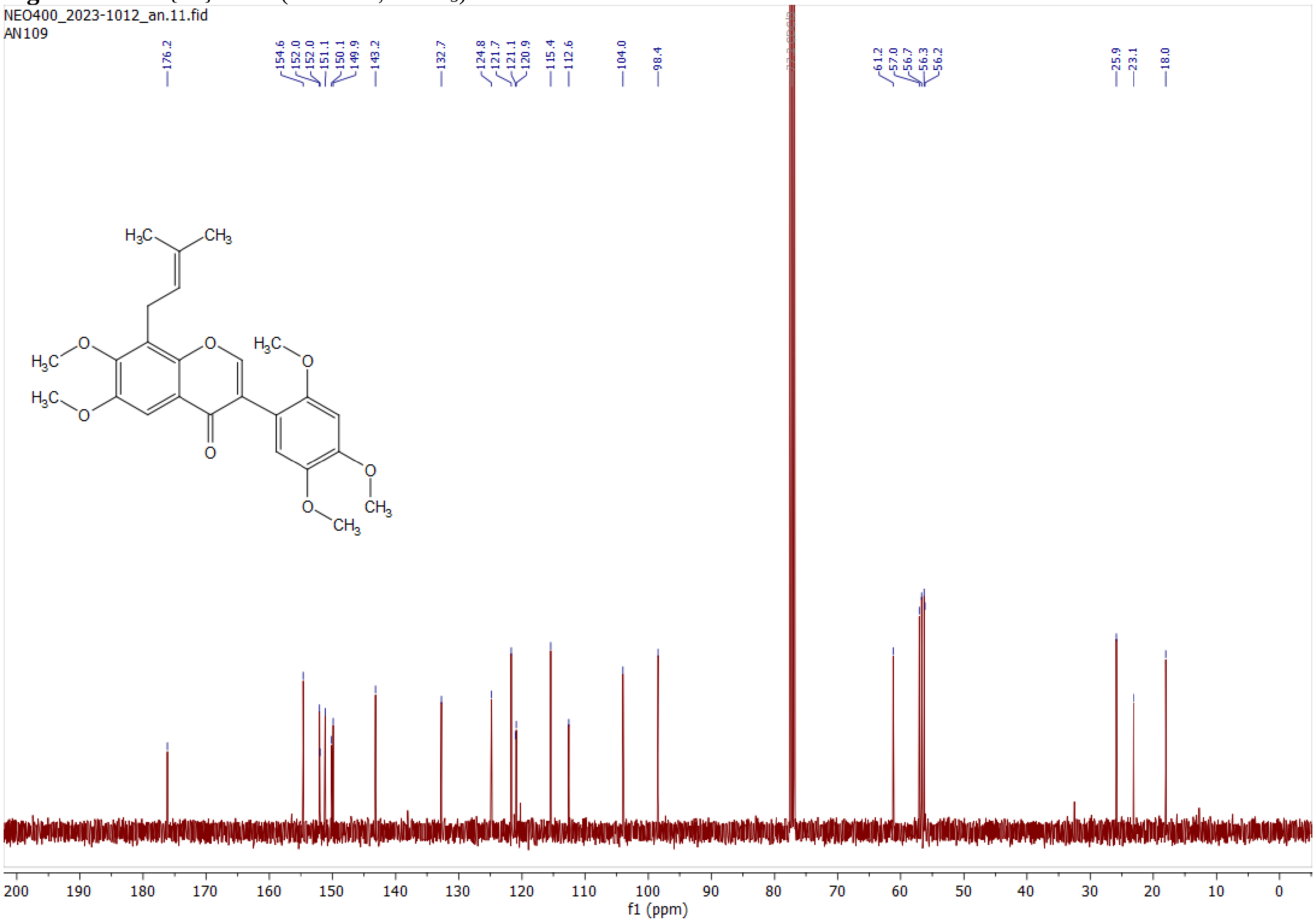

Figure S75: ^{1}H NMR (400 MHz, $CDCl_3$) of **22f**

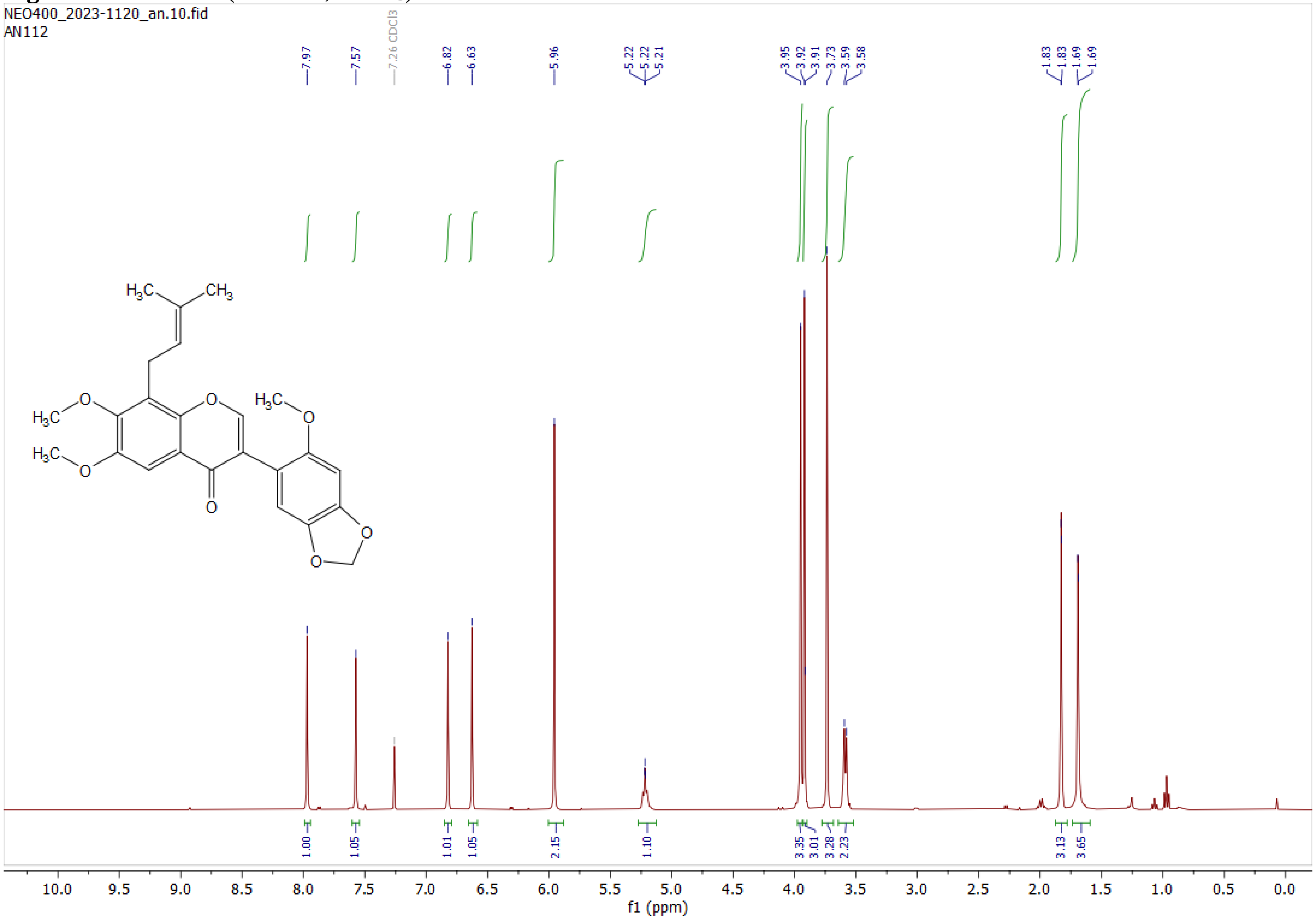

Figure S76: $^{13}C\{^{1}H\}$ NMR (101 MHz, $CDCl_3$) of **22f**

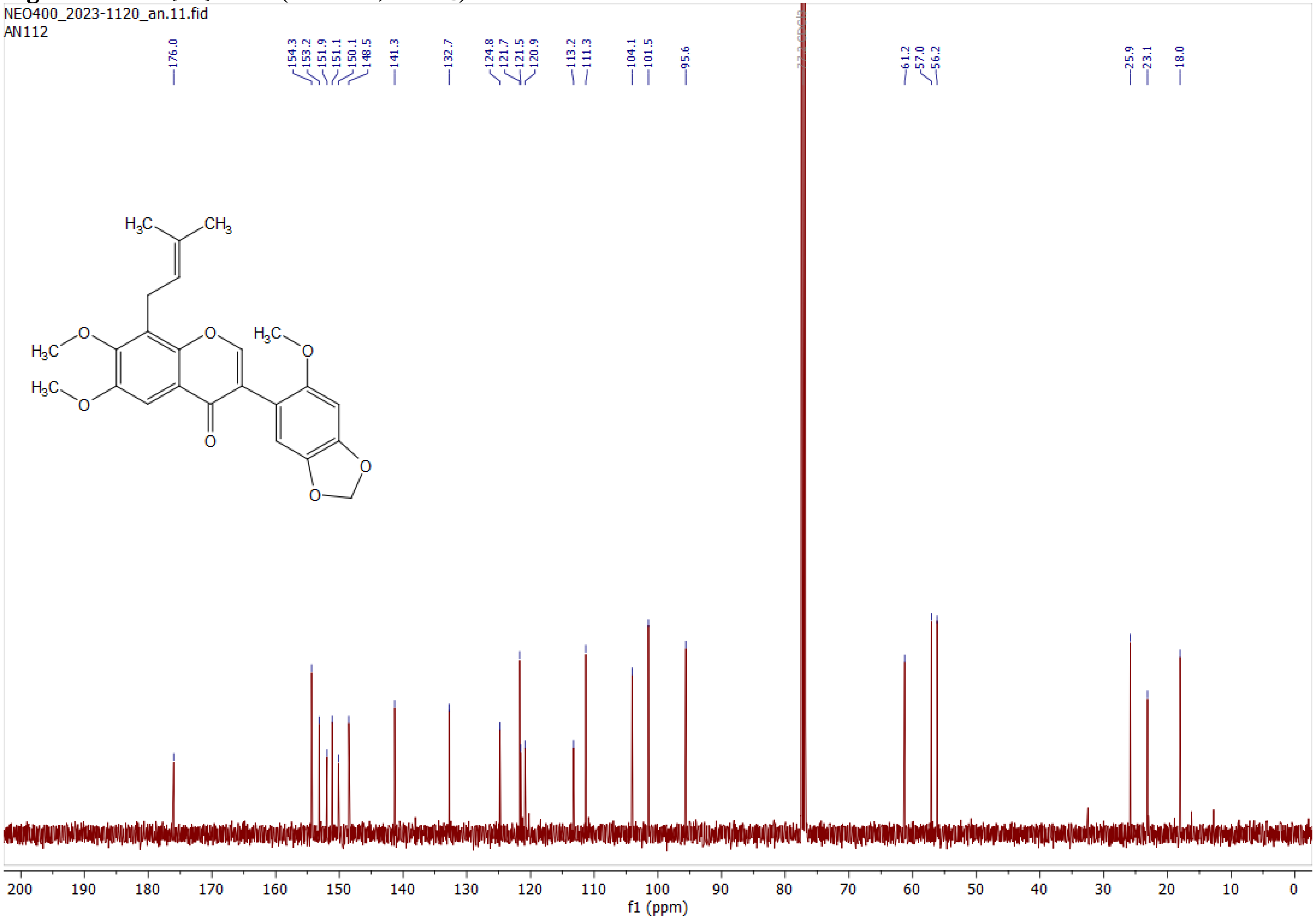

Figure S77: ^{1}H NMR (400 MHz, $CDCl_3$) of **23**

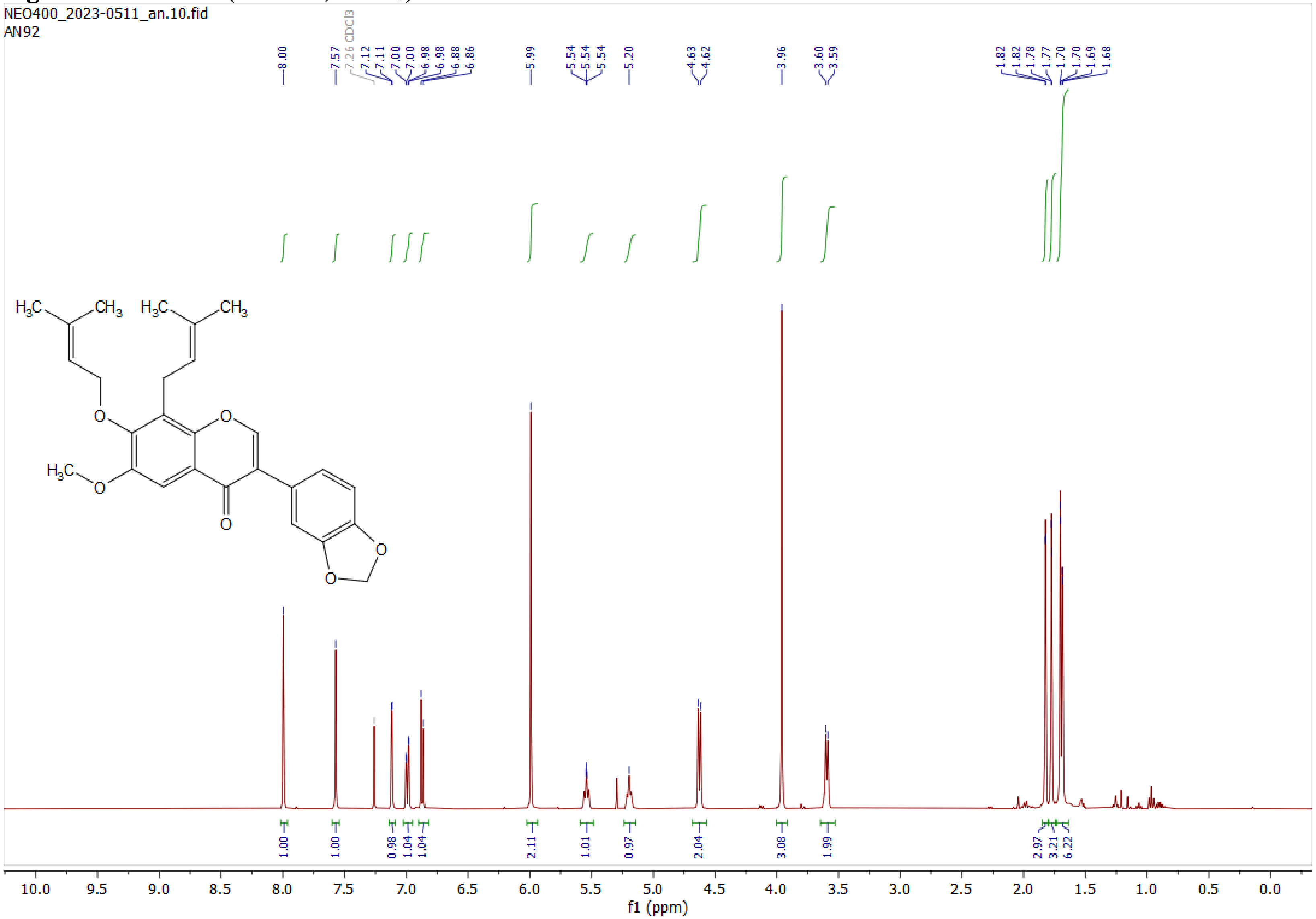

Figure S78: $^{13}C\{^{1}H\}$ NMR (101 MHz, $CDCl_3$) of **23**

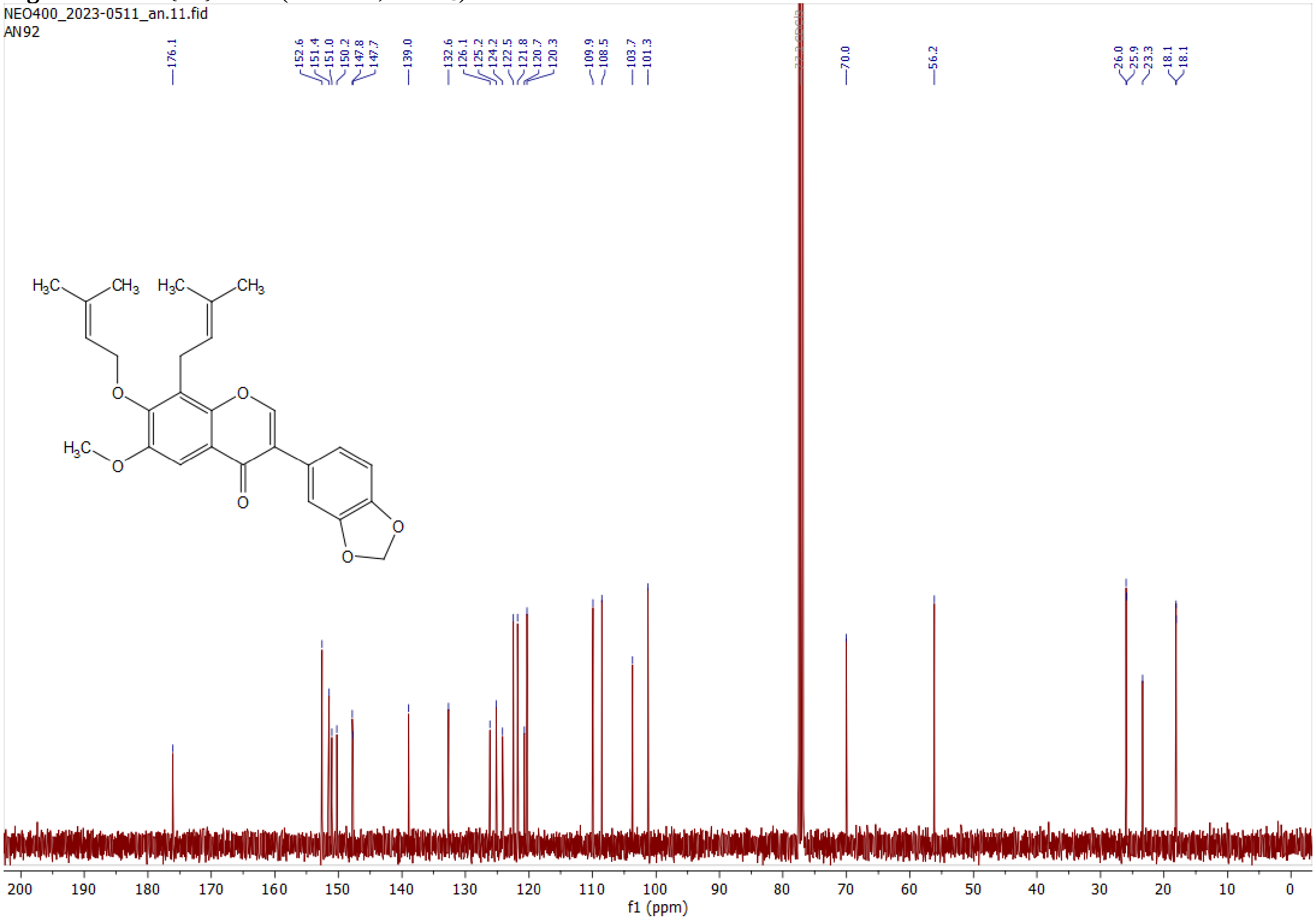

Table S4. Natural products synthesized in this study.

No	Trivial name	Natural source[ref]	Reported bioactivities[ref]
**21b**	Millesianin H	*Millettia dielsiana* Harms[13]	Inactive as NO production inhibitor[13]
**21c**	Predurallone	*M. dura*[14]	none
**21d**	Predurmillone	*M. ferruginea*[15]<br>*M. dielsiana* Harms[13]	Inactive as NO production inhibitor[13]
**21e**	Millesianin I[13]<br>Pachyloisoflavone A[16]	*M. dielsiana* Harms[13]<br>*M. pachyloba*[16]	Moderately active NO production inhibitor[13]
**21f**	Millesianin D	*M. dielsiana* Harms[17]<br>*M. pachyloba* Drake[18]<br>*M. dielsiana* Harms[13]	Moderately active NO production inhibitor[13]
**22b**	Pachyvone A	*M. pachyloba* Drake[19]	Inactive against HeLa and MCF-7 cell lines.[19]
**22d**	Pachyvone B	*M. ferruginea*[20]<br>*M. pachyloba* Drake.[19]	Inactive against HeLa and MCF-7 cell lines.[19]
**22e**	Placoisoflavone A[21]<br>Pachyvone C[19]	*Placolobium vietnamense* N.D.Khoi&Yakovlev[21]<br>*M. pachyloba* Drake.[19]	Active against human HepG2 cells (IC_{50} = 8.0 μM)[21]<br>Cytotoxicity against HeLa (IC_{50} = 7.9 μM) and HCT-116 (IC_{50} = 8.6 μM).[19]
**22f**	8-Prenylmilldurone	*M. oblata* ssp. *teitensis*[22]<br>*M. pachyloba* Drake[19]	Inactive against MDA-MB-231 human breast cancer cell lines.[22]<br>Cytotoxicity against HeLa (IC_{50} = 14.6 μM)[19]

Figure S79: Structures of natural products synthesized in this work and numbering scheme for the example Millesianin H.

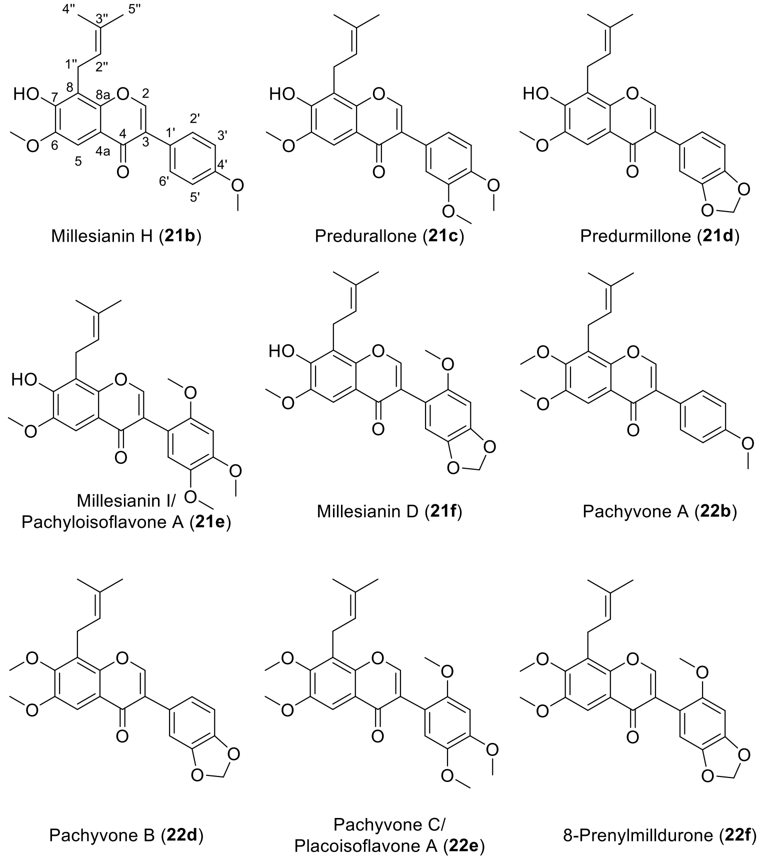

Table S5. ^{13}C NMR (100 MHz) and ^{1}H NMR (400 MHz) Data of the Synthesized Compound **21b** and comparison with Millesianin H, in $CDCl_3$

Position	**21b** (synthetic, this work)		Millesianin H[13] (from natural source)	
	δ_C	δ_H (*J in* Hz)	δ_C	δ_H (*J in* Hz)
2	152.1	7.99, s	152.0	7.99, s
3	124.8		124.6	
4	176.1		176.0	
4a	117.7		117.6	
5	102.4	7.55, s	102.3	7.55, s
6	145.2		145.1	
7	148.7		148.6	
8	115.7		115.6	
8a	151.0		150.8	
1′	124.0		123.8	
2′	114.1	6.97, d (8.7)	113.9	6.98, d (8.4)
3′	130.3	7.52, d (8.7)	130.1	7.50, d (8.4)
4′	159.6		159.5	
5′	130.3	7.52, d (8.7)	130.1	7.50, d (8.4)
6′	114.1	6.97, d (8.7)	113.9	6.98, d (8.4)
1″	22.5	3.59, d (7.2)	22.3	3.59, d (7.2)
2″	121.1	5.27, tm (7.2)	121.0	5.28, tm (7.2)
3″	133.1		132.8	
4″	18.0	1.84, s	17.9	1.84, s
5″	25.9	1.70, s	25.7	1.69, s
7-OH		6.38, s		6.38, s
6-OCH_3	55.5	3.84, s	55.3	3.85, s
4′-OCH_3	56.5	3.99, s	56.4	3.99, s

Table S6. ^{13}C NMR (100 MHz) and ^{1}H NMR (400 MHz) Data of the Synthesized Compounds **21c** and **21d** and comparison with Predurallone and Predurmillone, respectively, in $CDCl_3$

Position	**21c** (synthetic, this work)		**21d** (synthetic, this work)		Predurallone[14] (from natural source)		Predurmillone[15] (from natural source)	
	δ_C	δ_H (*J in* Hz)	δ_C	δ_H (*J in* Hz)	δ_C	δ_H (*J in* Hz)	δ_C	δ_H (*J in* Hz)
2	152.3	8.03, s	152.3	7.98, s	152.3	8.02, s	NA	7.95
3	124.0		124.1		123.9		NA	
4	176.2		176.0		176.2		NA	
4a	117.7		117.7		117.6		NA	
5	102.3	7.56, s	102.4	7.54, s	102.3	7.54, s	NA	7.53
6	145.2		145.2		145.3		NA	
7	148.9		148.8		148.9		NA	
8	115.8		115.8		115.8		NA	
8a	151.0		151.0		151.0		NA	
1′	125.2		126.2		125.2		NA	
2′	112.7	7.25, d (2.1)	109.9	7.12, d (1.7)	112.7	7.26, d	NA	7.10
3′	149.1		147.8		149.1		NA	
4′	148.8		147.6		148.9		NA	
5′	111.3	6.93, d (8.3)	108.5	6.87, d (8.0)	111.3	6.91, d	NA	6.85
6′	121.1	7.07, dd (8.3, 2.1)	122.5	6.99, dd (8.0, 1.7)	121.1	7.06, dd	NA	6.97
1″	22.5	3.59, d (7.1)	22.5	3.58, d (7.1)	22.4	3.58, d	NA	3.51
2″	121.1	5.27, tm (7.1)	121.1	5.27, tm (7.1)	121.1	5.27, t	NA	5.25
3″	133.1		133.1		133.0		NA	
4″	18.0	1.84, s	18.0	1.83, s	18.0	1.83, s	NA	1.80
5″	25.9	1.70, s	25.9	1.70, s	25.8	1.69, s	NA	1.68
7-OH		6.38, s		6.39, s		6.61, br s	NA	6.35
6-OCH_3	56.5	3.99, s	56.5	4.00, s	56.4	3.94, s	NA	4.00
3′-OCH_3	56.1	3.93, s			56.0	3.92, s		
4′-OCH_3	56.1	3.91, s			56.0	3.89, s		
-OCH_2-			101.3	5.99, s				6.00

Signal assignment for **21d** is based on 1D and 2D NMR; NA: Not available

Table S7. ^{13}C NMR (100 MHz) and ^{1}H NMR (400 MHz) Data of the Synthesized Compounds **21e** and **21f** and Comparison with Millesianin I (7-Methoxypredurmillone) and Millesianin D, respectively, in $CDCl_3$

Position	**21e** (synthetic, this work)		**21f** (synthetic, this work)		Millesianin I[13] (from natural source)		Millesianin D[17] (from natural source)	
	δ_C	δ_H (*J in* Hz)	δ_C	δ_H (*J in* Hz)	δ_C	δ_H (*J in* Hz)	δ_C	δ_H (*J in* Hz)
2	154.3	8.02, s	154.0	7.95, s	154.1	8.03, s	153.9	7.95, s
3	121.0		121.4		120.9		121.3	
4	176.1		176.0		176.0		175.9	
4a	117.7		117.7		117.6		117.3	
5	102.4	7.55, s	102.5	7.53, s	102.3	7.55, s	102.3	7.54, s
6	145.1		145.1		145.0		144.9	
7	148.6		148.6		148.5		148.5	
8	115.7		115.7		115.6		115.6	
8a	151.0		151.0		150.9		150.9	
1′	112.7		113.4		112.6		113.2	
2′	152.0		153.2		151.9		153.0	
3′	98.4	6.63, s	95.6	6.63, s	98.4	6.64, s	95.5	6.63, s
4′	149.8		148.5		149.7		148.3	
5′	143.2		141.3		143.1		141.2	
6′	115.4	6.96, s	111.4	6.83, s	115.4	6.96, s	111.2	6.83, s
1″	22.5	3.59, d (6.2)	22.5	3.58, d (7.1)	22.8	3.59, d (7.2)	22.3	3.58, d (7.2)
2″	121.1	5.28, tm (6.2)	121.1	5.28, tm (7.1)	121.0	5.28, t (7.2)	121.0	5.28, t (7.2)
3″	133.0		133.0		132.9		132.9	
4″	18.0	1.83, s	18.0	1.83, s	17.9	1.84, s	17.9	1.83, s
5″	25.9	1.70, s	25.9	1.70, s	25.8	1.70, s	25.7	1.70, s
7-OH		6.38, s		6.38, s		6.35, s		6.32, s
6-OCH_3	56.5	3.98, s	56.5	3.98, s	56.4	3.99, s	56.4	4.00, s
2′-OCH_3	57.1	3.79, s	57.1	3.74, s	57.0	3.79, s	56.9	3.74, s
4′-OCH_3	56.3	3.93, s			56.2	3.93, s		
5′-OCH_3	56.7	3.85, s			56.6	3.86, s		
-OCH_2-			101.5	5.95, s			101.3	5.96, s

Table S8. ^{13}C NMR (100 MHz) and ^{1}H NMR (400 MHz) Data of the Synthesized Compounds **22b** and **22d** and Comparison with Pachyvone A and Pachyvone B, respectively in $CDCl_3$

Position	**22b** (synthetic, this work)		**22d** (synthetic, this work)		Pachyvone A[19] (from natural source)		Pachyvone B[20] (from natural source)	
	δ_C	δ_H (*J in* Hz)	δ_C	δ_H (*J in* Hz)	δ_C	δ_H (*J in* Hz)	δ_C	δ_H (*J in* Hz)
2	152.4	8.02, s	152.6	8.00, s	152.3	8.02, s	152.5	8.01, s
3	124.1		124.2		123.9		124.1	
4	176.2		176.1		176.1		175.9	
4a	120.9		120.8		120.7		120.7	
5	104.0	7.59, s	104.0	7.58, s	103.8	7.60, s	103.8	7.59, s
6	151.2		151.3		151.1		151.1	
7	152.0		152.1		151.9		151.9	
8	124.8		124.8		124.7		124.7	
8a	150.1		150.1		150.0		149.9	
1′	124.6		126.1		124.5		125.9	
2′	130.2	7.52, d (8.8)	109.9	7.12, d (1.7)	130.1	7.52, d (8.8)	109.8	7.12, d (1.8)
3′	114.1	6.98, d (8.8)	147.7		113.9	6.98, d (8.8)	147.7	
4′	159.7		147.8		159.5		147.6	
5′	114.1	6.98, d (8.8)	108.5	6.87, d (8.0)	113.9	6.98, d (8.8)	108.4	6.88, d (8.0)
6′	130.2	7.52, d (8.8)	122.5	6.99, dd (8.0, 1.7)	130.1	7.52, d (8.8)	122.3	6.99, dd (8.0, 1.8)
1″	23.1	3.59, d (7.0)	23.1	3.59, d (7.0)	22.9	3.59, d (7.2)	22.9	3.59, d (7.2)
2″	121.7	5.21, tm (7.0)	121.6	5.20, tm (7.0)	121.5	5.21, t (7.2)	121.5	5.21, m
3″	132.8		132.8		132.7		132.7	
4″	18.0	1.84, s	18.0	1.83, s	17.9	1.84, s	17.9	1.83, s
5″	25.9	1.69, s	25.9	1.69, s	25.8	1.69, s	25.8	1.69, s
6-OCH_3	56.2	3.96, s	56.2	3.96, s	56.0	3.96, s	56.0	3.96, s
7-OCH_3	61.3	3.93, s	61.3	3.93, s	61.1	3.93, s	61.1	3.93, s
4′-OCH_3	55.5	3.85, s			55.3	3.84, s		
-OCH_2-			101.3	5.99, s			101.2	5.99, s

Table S9. ^{13}C NMR (100 MHz) and ^{1}H NMR (400 MHz) Data of the Synthesized Compounds **22e** and **22f** and Comparison with Pachyvone C (alternate name: Placoisoflavone A) and 8-Prenylmilldurone, respectively in $CDCl_3$

Position	**22e** (synthetic, this work)		**22f** (synthetic, this work)		Pachyvone C[21] (Placoisoflavone A) (from natural source)		8-Prenylmilldrone[22] (from natural source)	
	δ_C	δ_H (*J in* Hz)	δ_C	δ_H (*J in* Hz)	δ_C	δ_H (*J in* Hz)	δ_C	δ_H (*J in* Hz)
2	154.6	8.05, s	154.3	7.97, s	154.5	8.04, s	154.2	7.97, s
3	120.9		121.5		120.8		121.4	
4	176.2		176.0		176.0		175.9	
4a	121.1		120.9		120.9		120.8	
5	104.0	7.59, s	104.1	7.57, s	103.9	7.59, s	103.9	7.57, s
6	152.0		151.1		151.9		150.9	
7	152.0		151.9		152.0		151.8	
8	124.8		124.8		124.7		124.7	
8a	149.9		150.1		149.8		150.0	
1′	112.6		113.2		112.6		113.1	
2′	151.1		153.2		151.0		153.0	
3′	98.4	6.63, s	95.6	6.63, s	98.5	6.64, s	95.5	6.63, s
4′	150.1		148.5		150.0		148.4	
5′	143.2		141.3		143.1		141.2	
6′	115.4	6.95, s	111.3	6.82, s	115.4	6.96, s	111.2	6.82, s
1″	23.1	3.59, d (6.9)	23.1	3.59, d (7.0)	23.0	3.60, d (7.0)	23.0	3.59, d (7.2)
2″	121.7	5.22, tm (6.9)	121.7	5.22, tm (7.0)	121.6	5.23, t (7.0)	121.6	5.22, m
3″	132.7		132.7		132.6		132.6	
4″	18.0	1.83, s	18.0	1.83, s	17.9	1.84, s	17.9	1.83, s
5″	25.9	1.69, s	25.9	1.69, s	25.7	1.70, s	25.8	1.69, s
6-OCH_3	61.2	3.93, s	56.2	3.95, s	61.1	3.93, s	56.0	3.95, s
7-OCH_3	57.0	3.78, s	61.2	3.92, s	56.9	3.79, s	61.2	3.92, s
2′-OCH_3	56.2	3.95, s	57.0	3.74, s	56.0	3.95, s	56.9	3.74, s
4′-OCH_3	56.7	3.85, s			56.6	3.86, s		
5′-OCH_3	56.3	3.92, s			56.2	3.93, s		
-OCH_2-			101.5	5.96, s			101.4	5.97, s

D References

(1) STOE & Cie GmbH (2018) X-Area. software package for collecting single-crystal data on STOE area-detector diffractometers, for image processing, for the correction and scaling of reflection intensities and for outlier rejection. STOE & Cie GmbH, Darmstadt.

(2) Sheldrick, G. Crystal structure refinement with SHELXL. *Acta Cryst. C* **2015**, *C71* (1), 3-8. DOI: doi:10.1107/S2053229614024218. Sheldrick, G. A short history of SHELX. *Acta Cryst. A* **2008**, *A64* (1), 112-122. DOI: doi:10.1107/S0108767307043930.

(3) Kratzert, D. FinalCif. Version V113; online available at: https://dkratzert.de/finalcif.html (last accession date: 2023-03-09).

(4) Macrae, C. F.; Sovago, I.; Cottrell, S. J.; Galek, P. T. A.; McCabe, P.; Pidcock, E.; Platings, M.; Shields, G. P.; Stevens, J. S.; Towler, M.; et al. Mercury 4.0: from visualization to analysis, design and prediction. *J. Appl. Cryst.* **2020**, *53* (1), 226-235. DOI: doi:10.1107/S1600576719014092.

(5) Pedersen, D. S.; Rosenbohm, C. Dry Column Vacuum Chromatography. *Synthesis* **2001**, (16), 2431-2434. DOI: 10.1055/s-2001-18722.

(6) Lang'at-Thoruwa, C.; Song, T. T.; Hu, J.; Simons, A. L.; Murphy, P. A. A Simple Synthesis of 7,4‘-Dihydroxy-6-methoxyisoflavone, Glycitein, the Third Soybean Isoflavone. *J. Nat. Prod.* **2003**, *66* (1), 149-151. DOI: 10.1021/np020320r.

(7) Mendieta-Moctezuma, A.; Rugerio-Escalona, C.; Villa-Ruano, N.; Gutierrez, R. U.; Jiménez-Montejo, F. E.; Fragoso-Vázquez, M. J.; Correa-Basurto, J.; Cruz-López, M. C.; Delgado, F.; Tamariz, J. Synthesis and biological evaluation of novel chromonyl enaminones as α-glucosidase inhibitors. *Med. Chem. Res.* **2019**, *28* (6), 831-848. DOI: 10.1007/s00044-019-02320-w.

(8) Brzaszcz, M.; Kloc, K.; Maposah, M.; Mlochowski, J. Selenium(IV) Oxide Catalyzed Oxidation of Aldehydes to Carboxylic Acids with Hydrogen Peroxide. *Synth. Commun.* **2000**, *30* (24), 4425-4434. DOI: 10.1080/00397910008087069.

(9) Alves, A. P. L.; Júnior, J. A. B. C.; Slana, G. B. A.; Cardoso, J. N.; Wang, Q.; Lopes, R. S. C.; Lopes, C. C. Synthesis of 1,2,4-Trimethoxybenzene and Its Selective Functionalization at C-3 by Directed Metalation. *Synth. Commun.* **2009**, *39* (20), 3693-3709. DOI: 10.1080/00397910902805598.

(10) Tajik, H.; Mohammadpoor-Baltork, I.; Albadi, J. Bromination of Some Aromatic Compounds with Potassium Bromide in the Presence of Benzyltriphenylphosphonium Peroxodisulfate. *Synth. Commun.* **2007**, *37* (2), 323-328. DOI: 10.1080/00397910601033906.

(11) Burns, M. J.; Fairlamb, I. J. S.; Kapdi, A. R.; Sehnal, P.; Taylor, R. J. K. Simple Palladium(II) Precatalyst for Suzuki−Miyaura Couplings: Efficient Reactions of Benzylic, Aryl, Heteroaryl, and Vinyl Coupling Partners. *Org. Lett.* **2007**, *9* (26), 5397-5400. DOI: 10.1021/ol702291r.

(12) Novák, Z.; Timári, G.; Kotschy, A. The first total synthesis of Cicerfuran utilizing a one-pot synthesis of hydroxylated benzofurans. *Tetrahedron* **2003**, *59* (38), 7509-7513. DOI: https://doi.org/10.1016/S0040-4020(03)01170-0.

(13) Ye, H.; Wu, W.; Liu, Z.; Xie, C.; Tang, M.; Li, S.; Yang, J.; Tang, H.; Chen, K.; Long, C.; et al. Bioactivity-guided isolation of anti-inflammation flavonoids from the stems of *Millettia dielsiana* Harms. *Fitoterapia* **2014**, *95*, 154-159. DOI: https://doi.org/10.1016/j.fitote.2014.03.008.
(14) Yenesew, A.; Midiwo, J. O.; Waterman, P. G. Four isoflavones from seed pods of *Millettia dura*. *Phytochemistry* **1996**, *41* (3), 951-955. DOI: https://doi.org/10.1016/0031-9422(95)00662-1.
(15) Dagne, E.; Bekele, A. C-prenylated isoflavones from *Millettia ferruginea*. *Phytochemistry* **1990**, *29* (8), 2679-2682. DOI: https://doi.org/10.1016/0031-9422(90)85212-X.
(16) Na, Z.; Fan, Q.-F.; Song, Q.-S.; Hu, H.-B. Three new flavonoids from *Millettia pachyloba*. *Phytochemistry Lett.* **2017**, *19*, 215-219. DOI: https://doi.org/10.1016/j.phytol.2017.02.002.
(17) Gong, T.; Wang, D.-X.; Chen, R.-y.; Liu, P.; Yu, D.-Q. Novel Benzil and Isoflavone Derivatives from *Millettia dielsiana*. *Planta Med.* **2009**, *75* (03), 236-242. DOI: 10.1055/s-0028-1112203.
(18) Na, Z.; Song, Q.; Fan, Q. New Xanthone from Millettia pachyloba Drake. *Rec. Nat. Prod.* **2019**, *13* (5), 385-389.
(19) Yan, W.; Yang, J.; Tang, H.; Xue, L.; Chen, K.; Wang, L.; Zhao, M.; Tang, M.; Peng, A.; Long, C.; et al. Flavonoids from the stems of *Millettia pachyloba* Drake mediate cytotoxic activity through apoptosis and autophagy in cancer cells. *J. Adv. Res.* **2019**, *20*, 117-127. DOI: https://doi.org/10.1016/j.jare.2019.06.002.
(20) Deyou, T.; Jang, Y. P. A new prenylated isoflavone from the seeds of *Millettia ferruginea* ssp. *ferruginea*. *S. Afr. J. Bot.* **2018**, *117*, 155-157. DOI: https://doi.org/10.1016/j.sajb.2018.05.004.
(21) Do, L. T. M.; Huynh, T. T. N.; Tran, Q. H. N.; Nguyen, H. T. M.; Nguyen, T. T. A.; Nguyen, T. T. N.; Nguyen, P. H. H.; Sichaem, J. Placoisoflavones A and B, two new cytotoxic isoflavonoids from *Placolobium vietnamense* N.D.Khôi & Yakovlev. *Nat. Prod. Res.* **2024**, *38* (1), 112-118. DOI: 10.1080/14786419.2022.2110092.
(22) Deyou, T.; Marco, M.; Heydenreich, M.; Pan, F.; Gruhonjic, A.; Fitzpatrick, P. A.; Koch, A.; Derese, S.; Pelletier, J.; Rissanen, K.; et al. Isoflavones and Rotenoids from the Leaves of *Millettia oblata* ssp. *teitensis*. *J. Nat. Prod.* **2017**, *80* (7), 2060-2066. DOI: 10.1021/acs.jnatprod.7b00255.
